# Formation of Nucleophilic Allylboranes from Molecular Hydrogen and Allenes Catalyzed by a Pyridonate Borane that Displays Frustrated Lewis Pair Reactivity

**DOI:** 10.1002/anie.202011790

**Published:** 2020-10-22

**Authors:** Max Hasenbeck, Sebastian Ahles, Arthur Averdunk, Jonathan Becker, Urs Gellrich

**Affiliations:** ^1^ Institut für Organische Chemie Justus-Liebig-Universität Gießen Heinrich-Buff-Ring 17 35392 Gießen Germany; ^2^ Institut für Anorganische und Analytische Chemie Justus-Liebig-Universität Gießen Heinrich-Buff-Ring 17 35392 Gießen Germany

**Keywords:** allylboranes, boron–ligand cooperation, DFT computations, frustrated Lewis pairs, hydrogen activation

## Abstract

Here we report the in situ generation of nucleophilic allylboranes from H_2_ and allenes mediated by a pyridonate borane that displays frustrated‐Lewis‐pair reactivity. Experimental and computational mechanistic investigations reveal that upon H_2_ activation, the covalently bound pyridonate substituent becomes a datively bound pyridone ligand. Dissociation of the formed pyridone borane complex liberates Piers borane and enables a hydroboration of the allene. The allylboranes generated in this way are reactive towards nitriles. A catalytic protocol for the formation of allylboranes from H_2_ and allenes and the allylation of nitriles has been devised. This catalytic reaction is a conceptually new way to use molecular H_2_ in organic synthesis.

## Graphical abstract

The in situ formation of nucleophilic allylboranes from H_2_ and allenes is reported. The reaction is catalyzed by pyridonate borane that displays frustrated‐Lewis‐pair reactivity. The allylboranes that are generated in this way are reactive towards nitriles. Mechanistic investigations show that a change in the binding mode of the pyridonate substituent upon hydrogen activation is essential for the formation of the allylboranes.

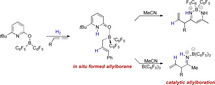

## Introduction

**Scheme 1:**
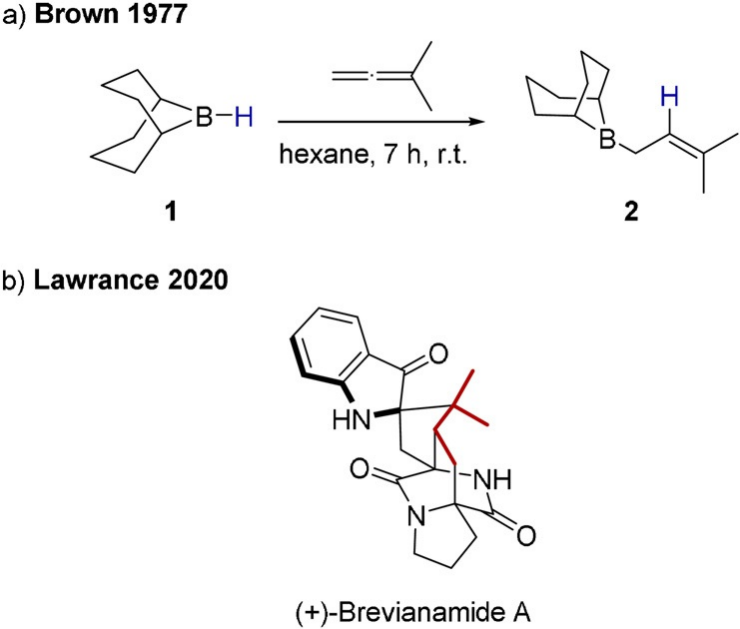
a) Formation of B‐3,3‐dimethallyl‐9‐BBN **2** by hydroboration of dimethylallene reported by Brown. b) Structure of Brevianamide A with the fragment originating from an allylborane highlighted.

Allylboranes are a prevalent class of C‐nucleophiles.[^1^, ^2^, ^3^] Classic ways for their preparation include the addition of nucleophilic allyl Grignard or allyl lithium compounds to electrophilic boron methoxides.^[4]^ However, already in 1977 Kramer and Brown reported the formation of nucleophilic allylboranes upon hydroboration of allenes by 9‐borabicyclo[3.3.1]nonane (9‐BBN, **1**) (Scheme 1).^[5]^ The allylborane **2** prepared in this way was used in the first total synthesis of Brevianamide A, a highly challenging target for organic synthesis, reported earlier this year. This demonstrates the ongoing importance of this class of nucleophiles for organic synthesis.^[6]^

**Scheme 2:**
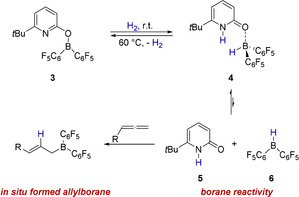
Envisioned in situ formation of a nucleophilic allylborane upon hydrogen activation by **3** and hydroboration of an allene.

We recently reported reversible H_2_ activation by the pyridonate borane **3** that can be described as an intramolecular frustrated Lewis pair (FLP).[^7^, ^8^, ^9^] A distinguishing feature of this system is that the H_2_ activation is associated with a transition of the covalently bound pyridonate substituent to a datively bound pyridone ligand (Scheme 2).

Piers borane **6** has been shown to display the typical reactivity of a trivalent borane, for example, the hydroboration of unsaturated double bonds.^[12]^ We, therefore, envisioned that Piers borane **6**, formed in situ upon H_2_ activation by **3** and dissociation of **4**, is able to hydroborate an allene, yielding a nucleophilic allylborane.^[13]^ The formation of a nucleophilic allylborane from H_2_ and an allene would be an atom economic and conceptually new way to use molecular hydrogen for the formation of a reactive organic intermediate. Furthermore, a reaction sequence consisting of allylation of an electrophile by the allylborane formed in this way, followed by a protodeborylation mediated by the pyridone **5**, would regenerate the pyridonate borane **3**. That would enable us to realize an allylation that requires only catalytic amounts of the pyridonate borane **3**.

## Results and Discussion

**Scheme 3:**
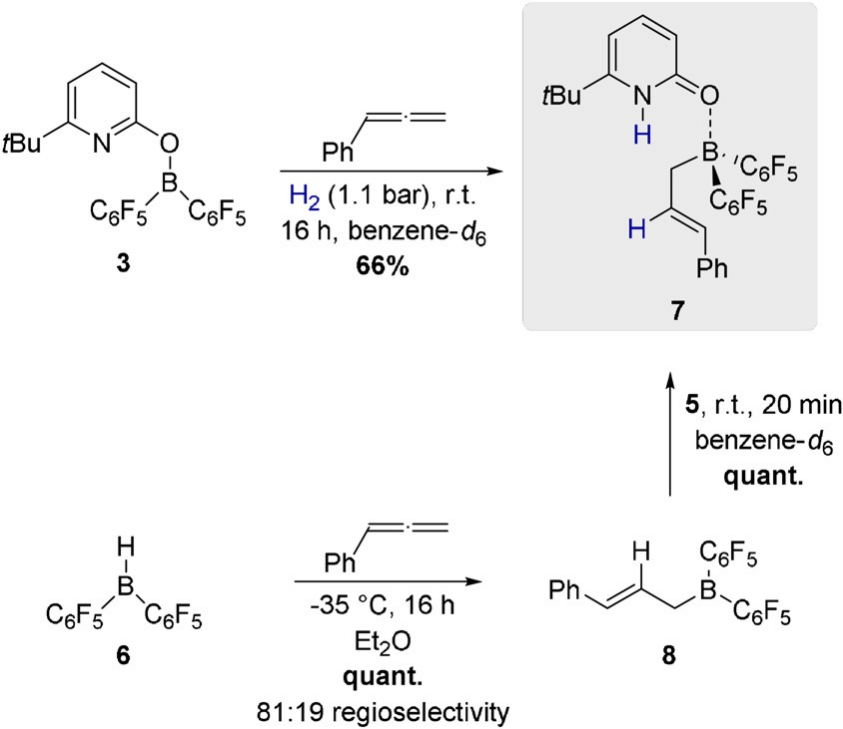
Formation of the pyridone allylborane complex **7** upon hydrogen activation by **3** and the stepwise formation of **7** upon hydroboration of phenylallene by Piers borane **6** and addition of the pyridone **5**. Yields were determined by ^1^H NMR with 1,3,5‐trimethoxybenzene as internal standard.

To prove the working hypothesis, phenylallene was added to a solution of **3** in benzene and the reaction mixture was exposed to H_2_ (1.1 bar). This led to the formation of a new borane complex that was assigned to be the pyridone allylborane complex **7** in 66 % yield (Scheme 3). To substantiate the assumed reaction sequence involving a hydroboration and re‐coordination of the pyridone **5** to the formed allylborane, Piers borane **6** was reacted with phenylallene (Scheme 3). At −35 °C in diethylether, phenyallene was quantitatively hydroborated and the allylborane **8** was obtained with a regioselectivity of 81:19. In the next step, the pyridone **5** was added at r.t. This yielded the pyridone allylborane complex **7** in quantitative yield.

**Scheme 4:**
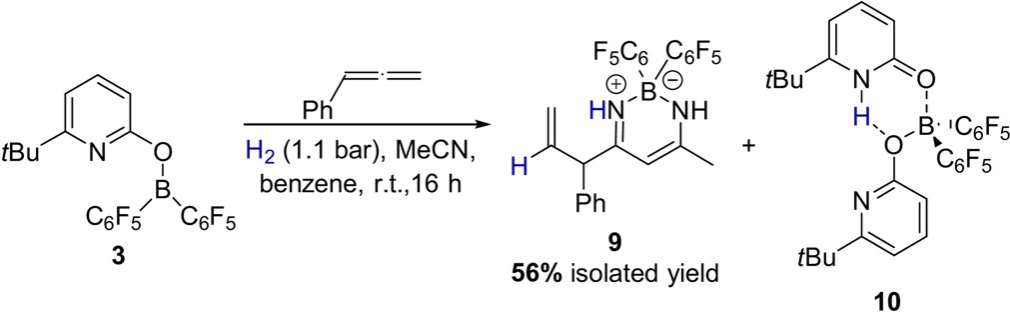
Formation of the β‐diketiminate borane complex **9** upon the reaction of **3** with acetonitrile and phenylallene under hydrogen pressure.

Thus, we were able to demonstrate that it is indeed possible to form an allylborane from hydrogen and phenylallene. In the next stage of this research project, we aimed to examine if the allylboranes generated in this way could be used as nucleophiles for an allylation reaction. A prerequisite for the use of **3** for an allylation is that the pyridonate borane **3** is able to activate hydrogen in the presence of a Lewis basic electrophile. To elucidate if this condition is met and if **7** serves as a source for nucleophilic allylboranes, **3** was reacted with phenylallene and one equivalent acetonitrile under moderated H_2_ pressure (1.1 bar). However, this reaction did not yield the expected allylimine, but the β‐diketiminate borane complex **9** together with the bispyridone complex **10** (Scheme 4).

**Figure 1:**
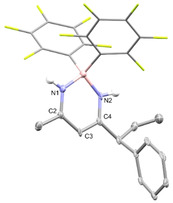
Molecular structure of **9** derived from SCXRD (50 % probability ellipsoids, all hydrogens attached to carbons are omitted and C_6_F_5_ rings are shown in stick representation for clarity). Selected bond lengths and angles: N1‐B: 1.528(17) Å, N1‐C2 1.336(17) Å, C2‐C3 1.403(19) Å, C3‐C4 1.390(19) Å, N2‐C4: 1.315(17) Å, N2‐B: 1.539(17) Å, C2‐N1‐B: 125.5(2)°, C4‐N2‐B: 126.7(2)°.

The β‐diketiminate borane complex **9** that proved to be air stable was isolated by silica gel column chromatography in 56 % yield and fully characterized by NMR and ESI‐MS. The structural assignment is further supported by single‐crystal X‐ray diffraction (SCXRD, Figure 1). The β‐diketiminate borane complex **9** is reminiscent to the core structure of BODIPY dies.^[15]^ Indeed, **9** is a fluorophore that absorbs at *λ*_max_=359 nm and emits at *λ*_max_(fluorescence)=409 nm with a molar attenuation coefficient of ϵ_359_=6928 cm^−1^ M^−1^ (see SI for full spectra).

**Scheme 5:**
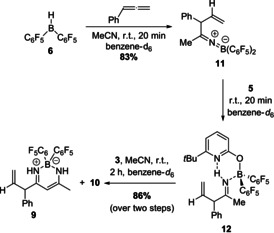
Stepwise formation of the β‐diketiminate borane complex **9** from Piers borane **6**. Yields were determined by ^1^H NMR with 1,3,5‐trimethoxybenzene as internal standard.

The β‐diketiminate borane complex **9** presumably originates from a nucleophilic attack of the enamine tautomer of an intermediately formed allylimine to acetonitrile. To verify this hypothesis and to check if an allylimine was formed as intermediate *en route* to **9**, the allylborane **8**, in situ generated by hydroboration of phenylallene, was reacted with one equivalent acetonitrile. This reaction furnished the ketiminoborane **11**, which is the allylation product of acetonitrile, in 83 % yield. The formation of **11** demonstrates that allylborane **8** is indeed nucleophilic (Scheme 5).

**Figure 2:**
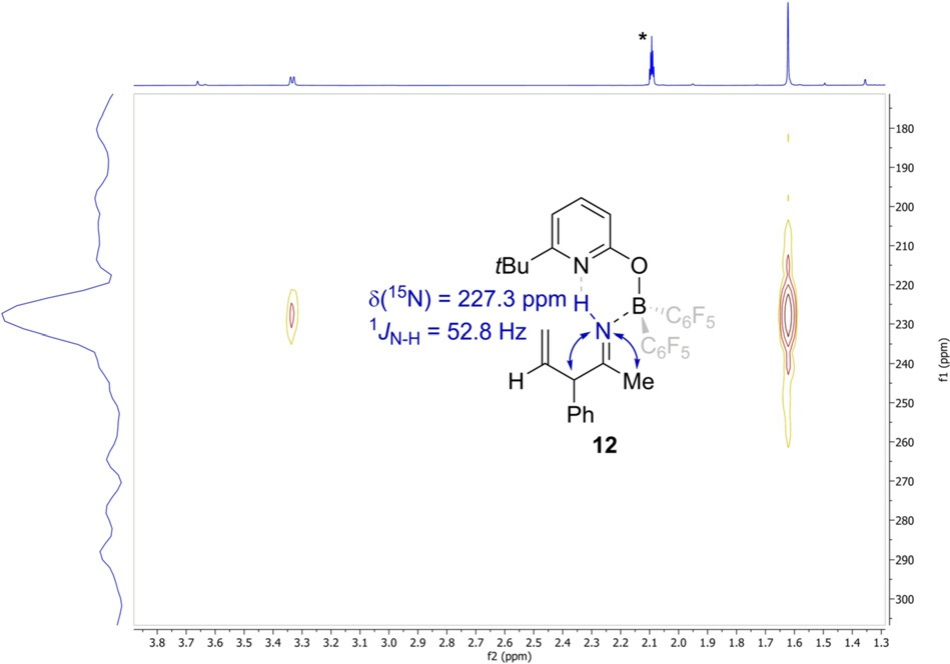
^15^N‐^1^H HMBC NMR spectrum of the allylimine complex **12** (600 MHz, [D_8_]toluene). The blue arrows indicate the observed ^15^N‐^1^H correlations. The signal marked with a star can be assigned to a solvent residue peak from [D_8_]toluene.

The ^11^B NMR shift of 21.4 ppm and the C=N stretching vibration of 1864 cm^−1^ indicate that **11** is present as monomeric ketiminoborane with linearity at nitrogen.^[14]^ Next, pyridone **5** was added to the ketiminoborane **11**. This sequence yielded the allylimine complex **12** (Scheme 5). The NH signal of **12** is found at 10.90 ppm, indicating an N−H⋅⋅⋅N hydrogen bond. A ^15^N‐^1^H HSQC NMR experiment shows that the proton is bound to a nitrogen with a shift of 227.3 ppm. Additional ^15^N‐^1^H HMBC NMR experiments revealed that this nitrogen couples to the methyl group and to the CHPh group of the allylimine **12** (Figure 2, for full spectra see SI).

**Scheme 6:**
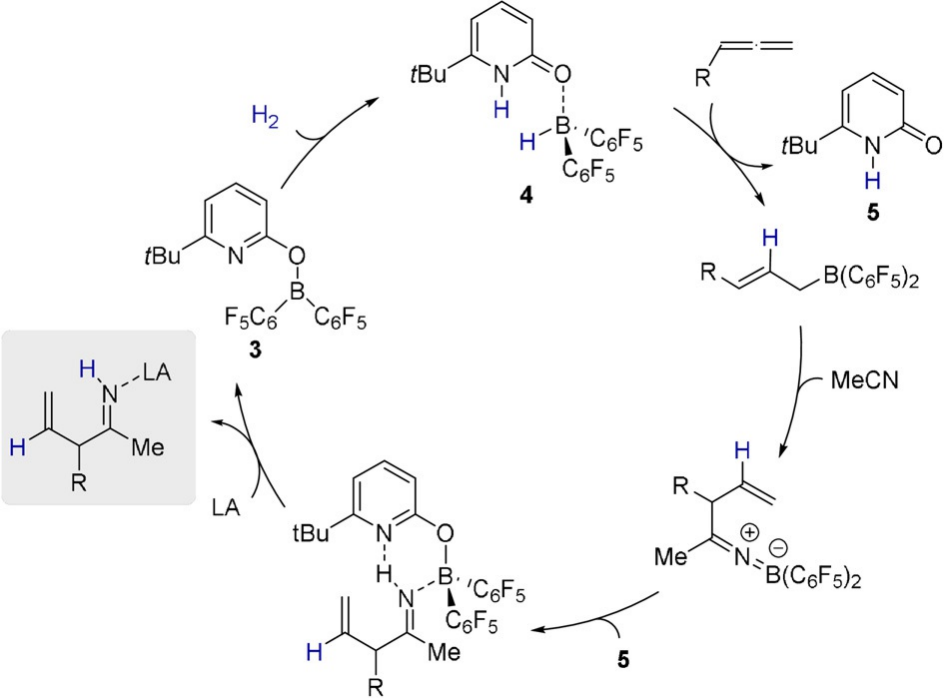
Envisioned catalytic cycle for the allylation of nitriles using an additional Lewis acid that captures the allylimine (grey box) (LA=Lewis acid).

Thus, the NMR experiment confirms the proton transfer from the pyridon **5** to the nitrogen of the ketiminoborane **11** and formation of an allylimine. The addition of one further equivalent acetonitrile and pyridonate borane **3** led to the clean formation of the β‐diketiminate borane complex **9** in 86 % yield (Scheme 5). This experiment strongly supports the assumption that upon the reaction of **3** with phenylallene and acetonitrile under H_2_ pressure, which leads to the formation of **9**, the desired allylimine was indeed formed intermediately (Scheme 4). Therefore, the formation of **9** from phenylallene and acetonitrile proves that **3** is not only able to activate dihydrogen in the presence of acetonitrile, but also mediates the subsequent allylation, leading to an allylimine. However, the B(C_6_F_5_)_2_ fragment is irreversibly bound in **9** which inhibits any further catalytic reactivity. We envisioned that the addition of a strong Lewis acid would enable us to capture the intermediately formed allylimine prior to the β‐diketiminate borane complex formation (Scheme 6).

**Scheme 7:**
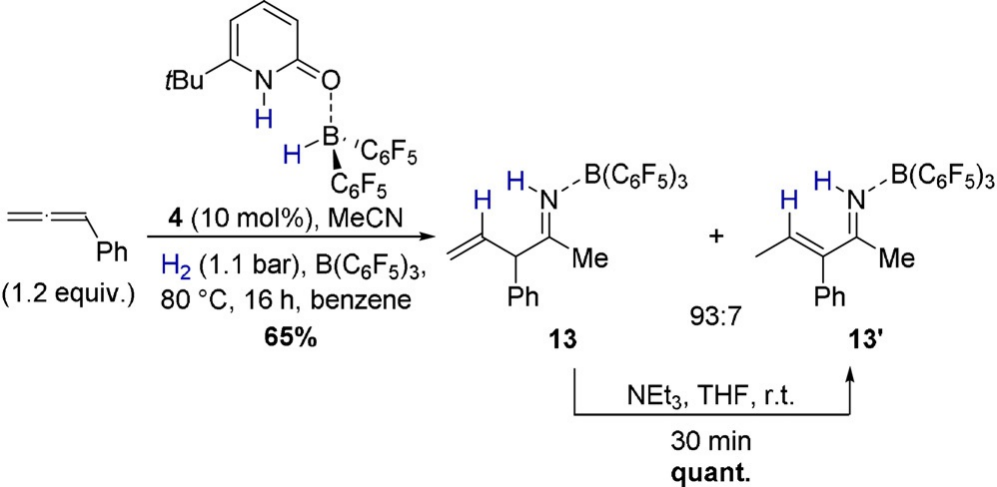
Formation of the allylimine borane complex **13** catalyzed by **4**, and its isomerization to the vinylimine **13′**. One equivalent acetonitrile and B(C_6_F_5_)_3_ were used.

As the liberation of the allylimine from **12** would furthermore regenerate the pyridonate borane **3**, a catalytic allylation can be envisioned. Indeed, upon addition of B(C_6_F_5_)_3_ the formation of the allylimine borane complex **13** from phenylallene, acetonitrile, and dihydrogen in presence of catalytic amounts of the pyridone borane complex **4** (10 mol %) was observed (Scheme 7).

**Figure 3:**
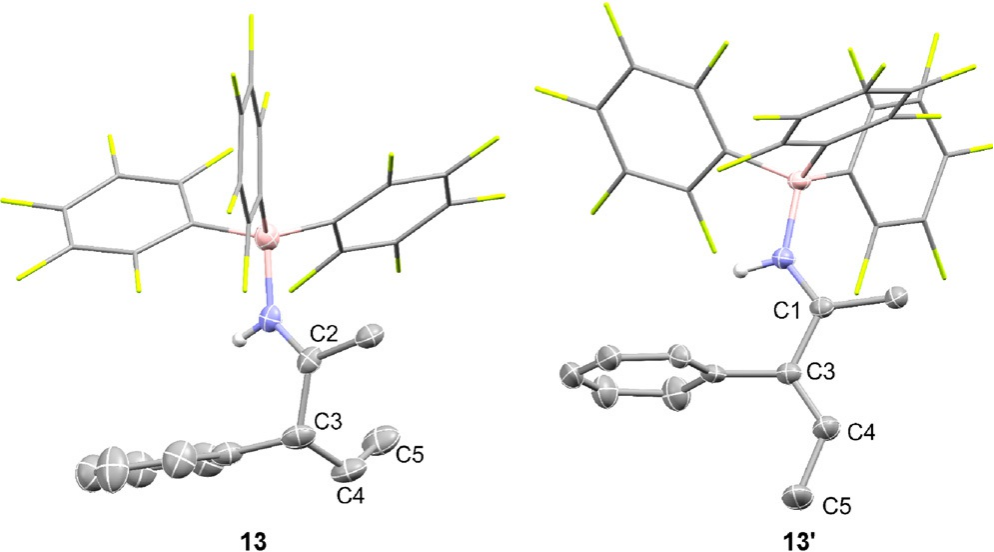
Molecular structure of **13** and **13′** derived from SCXRD (50 % probability ellipsoids, all hydrogens attached to carbons are omitted and C_6_F_5_ rings are shown in stick representation for clarity). Selected bond lengths and angles: **13**: C2‐C3 1.528(8) Å, C3‐C4 1.514(9) Å, C4‐C5 1.310(5) Å, N1‐C2‐C3: 121.7(8)°, C3‐C4‐C5: 126.2(4)°; **13′**: C1‐C3 1.4744(17) Å, C3‐C4 1.3408(18) Å, C4‐C5 1.497(2), N1‐C1‐C3: 118.07(11)°, C3‐C4‐C5: 124.67(14)°.

The allylimine borane complex **13** proved to be air‐stable and was isolated in 65 % yield. However, on silica gel **13** isomerizes to the vinylimine **13′** as proven by two‐dimensional TLC (see SI). Thus, the two isomers, fully characterized by NMR and ESI‐MS, were obtained in a 93:7 ratio after column chromatography. The molecular structure of **13** in the solid‐state was further analyzed by SCXRD (Figure 3). The allylimine borane complex **13** can be quantitatively isomerized to the vinylamine **13′** by addition of triethylamine at r.t. within minutes, indicating that **13′** is the thermodynamically more stable isomer. The molecular structure of **13′** derived from SCXRD confirms the *E*‐configuration of the double bond.

**Scheme 8:**
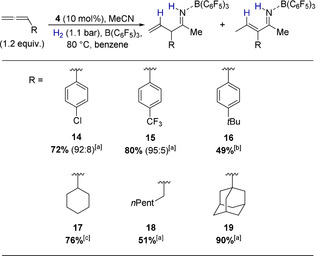
Allylation of acetonitrile catalysed by **4** with different allenes. One equivalent acetonitrile and B(C_6_F_5_)_3_ were used. All yields are isolated yields. The number in parenthesis indicate the ratio of allylimine to vinylimine. [a] Reaction time 5 days. [b] Reaction time 16 hours. [c] Reaction time 7 days.

To prove that the catalytic activity of **4** is more general, we explored next the scope and limitations of this novel allylation at the example of six different allenes (Scheme 8). Electron withdrawing groups are tolerated, but longer reaction times are required to obtain good yields. In the case of *tert*‐butyl phenyl substituted allene the product **16** is only obtained in moderate yields. However, an NMR analysis of the crude reaction mixture showed that the allene is consumed after 16 h reaction time, indicating that side reactions lower the yield in this case. Aliphatic allenes are suitable substrates, but again longer reaction times are required. The formation of the adamantly substituted allylimine **19** in excellent yield further shows that sterically demanding substituents are tolerated. The products **17**, **18**, and **19** did not isomerize upon purification by silica gel chromatography. However, we demonstrated at the example of **18** that the allylimines with aliphatic substituents can be isomerized to the respective vinylimines by the addition of a base. Treatment of **18** with NEt_3_ at r.t. for 30 minutes allowed us to isolate the corresponding vinylimine in 95 % yield (see SI).

**Figure 4:**
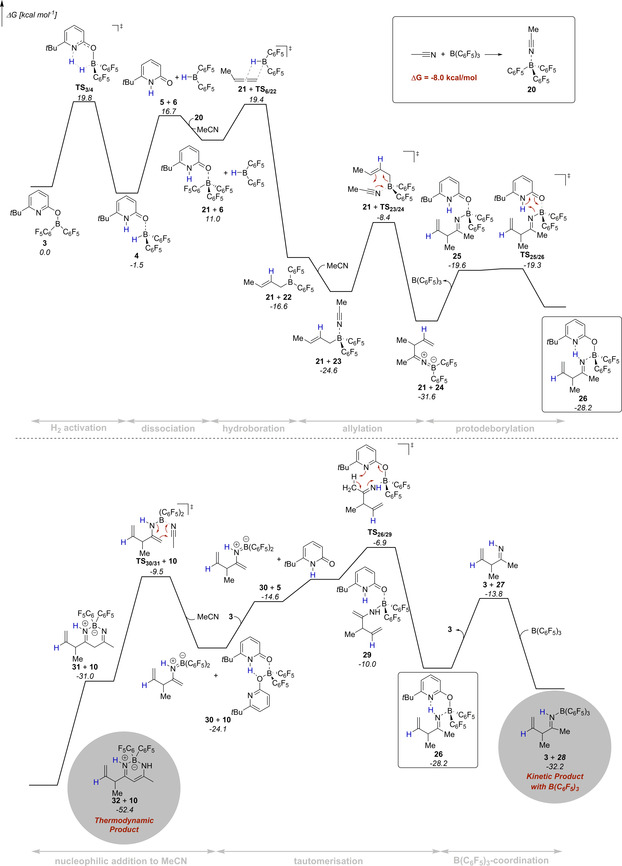
Potential energy surface for the formation of the β‐diketiminate borane complex **32** and allylimine borane complex **28** computed at revDSD‐PBEP86‐D4/def2‐QZVPP//PBEh‐3c. The SMD model for benzene was used to implicitly account for solvent effects.

The mechanism of this novel catalytic transformation was further addressed by dispersion‐corrected Density Functional Theory (DFT) computations at the revDSD‐PBEP86‐D4/def2‐QZVPP//PBEh‐3c level (Figure 4).[^16^, ^17^, ^18^] The revDSD‐PBEP86‐D4 functional is one of the best DFT methods for ground‐state thermochemistry and kinetics as shown by benchmark computations using the GMTKN55 database.[^16a^, ^19^] The SMD model for benzene was used to implicitly account for solvent effects.^[20]^ We used 1,2‐butadiene as model substrate and further assumed, that under the reaction conditions complex **20** forms upon coordination of acetonitrile to B(C_6_F_5_)_3_. In agreement with previous investigations, the computations reveal that the catalytic cycle commences with hydrogen activation by the pyridonate borane **3**. The pyridone borane complex **4** that is formed in this way dissociates to liberate Piers borane **6**. As a stoichiometric amount of B(C_6_F_5_)_3_ is present, we considered that the pyridone **5** coordinates to the B(C_6_F_5_)_3_, which overall renders the liberation of Piers borane **6** thermodynamically more favorable. The hydroboration of the terminal allene requires passing a moderate barrier of 8.4 kcal mol^−1^ and is according to the computations exergonic by 27.6 kcal mol^−1^.

**Scheme 9:**
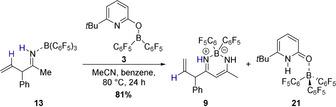
Conversion of the allylimine B(C_6_F_5_)_3_ complex **13** to the β‐diketiminate borane complex **9**, which is the thermodynamic product of the allylation, catalyzed by **3**. Yields were determined by ^1^H NMR with 1,3,5‐trimethoxybenzene as internal standard.

Coordination of acetonitrile to the allylborane **22** precedes the intramolecular allylation via a cyclic six‐membered transition state with a barrier of 16.2 kcal mol^−1^. The computed structure of the ketiminoborane **24** shows a C=N=B angle of 178.7°, which agrees with the linearity at nitrogen deduced from the experimental C=N stretching vibration of **11**. Upon dissociation of pyridone B(C_6_F_5_)_3_ complex **21** and recoordination of **5**, a virtually barrier‐less intramolecular proton transfer yields the allylimine complex **26**. Based on our experimental findings, we propose that the allylimine complex **26** is the common intermediate for the formation of the β‐diketiminate borane complex **32** and the allylimine B(C_6_F_5_)_3_ complex **28**. In the absence of B(C_6_F_5_)_3_, an intramolecular proton transfer from the methyl group of the allylimine to the pyridine nitrogen that requires a Gibbs free activation energy of 21.3 kcal mol^−1^ yields the pyridone enamine borane complex **29**. Dissociation of this complex, thermodynamically favored by formation of the bispyridone complex **10**, enables the nucleophilic addition of the enamine to acetonitrile via the six‐membered transition state **TS_30/31_**. While the C−C bond formation is already exergonic, the tautomerization to the diketiminate borane complex **32** provides a further decisive driving force to the reaction. In the presence of B(C_6_F_5_)_3_, the kinetically favored pathway commences with the dissociation of the allylimine complex **26** that is endergonic by 14.4 kcal mol^−1^. Coordination of the B(C_6_F_5_)_3_ to the free allylimine **27** yields the allylimine B(C_6_F_5_)_3_ complex **28**. The coordination of B(C_6_F_5_)_3_ to the allyimine **27** imparts a barrier of 25.3 kcal mol^−1^ for the tautomerization to the pyridone enamine borane complex **29** and therefore suppresses the formation of the β‐diketiminate borane complex **32**. However, according to the computations, the allylimine B(C_6_F_5_)_3_ complex **28** must be regarded as the kinetic product while the β‐diketiminate borane complex **32** is the thermodynamic product of the initial allylation. To verify this computational result experimentally, the isolated allylimine B(C_6_F_5_)_3_ complex **13** was reacted with one additional equivalent MeCN and the pyridonate borane **3** at an elevated temperature of 80 °C (Scheme 9). Within 24 h, the formation of the β‐diketiminate borane complex **9** in 81 % yield was observed.

This result confirms, in agreement with computations, that the β‐diketiminate borane complex **9** is the thermodynamic more stable product.

## Conclusion

In summary, we have documented the formation of allylboranes from molecular hydrogen and allenes mediated by a pyridonate borane. The stochiometric reaction of these in situ generated allylboranes with acetonitrile leads to a β‐diketiminate borane complex. By using an additional Lewis acid, we were able to develop a method for the allylation of nitriles that requires only catalytic amounts of the pyridonate borane. Mechanistic investigations reveal that the change in the binding mode of the pyridonate substituent in course of the hydrogen activation is vital for the formation of the allylborane. The results presented herein might stimulate the development of metal‐free, atom‐economic catalytic allylations.

## Conflict of interest

The authors declare no conflict of interest.

## Supporting information

As a service to our authors and readers, this journal provides supporting information supplied by the authors. Such materials are peer reviewed and may be re‐organized for online delivery, but are not copy‐edited or typeset. Technical support issues arising from supporting information (other than missing files) should be addressed to the authors.

Supplementary
