# Supplementary material for: Formation of Nucleophilic Allylboranes from Molecular Hydrogen and Allenes Catalyzed by a Pyridonate Borane that Displays Frustrated Lewis Pair Reactivity
Source: Angew Chem Int Ed Engl. 2020 Oct 22;59(52):23885–91. doi: 10.1002/anie.202011790 (PMC7814673; doi:10.1002/anie.202011790)
Supplement: Supplementary file 1 — Supplementary [file ANIE-59-23885-s001.pdf]

Angewandte Chemie
Eine Zeitschrift der Gesellschaft Deutscher Chemiker

Supporting Information

Formation of Nucleophilic Allylboranes from Molecular Hydrogen and Allenes Catalyzed by a Pyridonate Borane that Displays Frustrated Lewis Pair Reactivity

*Max Hasenbeck, Sebastian Ahles, Arthur Averdunk, Jonathan Becker, and Urs Gellrich**

anie_202011790_sm_miscellaneous_information.pdf

Supplementary information

1 Table of contents

2 General Information 4

3 Synthesis and characterization of mechanistically relevant intermediates 6

3.1 Synthesis and characterization of cinnamylbis(perfluorophenyl)borane pyridone complex **7** 6

3.2 Synthesis and characterization of cinnamylbis(perfluorophenyl)borane **8** 10

3.2.1 NMR spectra of cinnamylbis(perfluorophenyl)borane **8** 11

3.3 Synthesis and characterization of cinnamylbis(perfluorophenyl)borane pyridone complex 7 from cinnamylbis(perfluorophenyl)borane **8** 23

3.3.1 NMR spectra of cinnamylbis(perfluorophenyl)borane pyridone complex **7** 24

3.4 Synthesis and characterization of β-diketiminate borane complex **9** 36

3.4.1 NMR spectra of β-diketiminate borane complex **9** 37

3.4.2 UV-Vis and fluorescence spectrum of β-diketiminate borane complex **9** 44

3.5 Synthesis and characterization of ketiminoborane **11** 45

3.5.1 IR spectrum of ketiminoborane **11** 46

3.5.2 NMR spectra of ketiminoborane **11** 47

3.6 Synthesis and characterization of ketiminoborane pyridone complex **12** 55

3.6.1 NMR spectra of ketiminoborane pyridone complex **12** 56

4 Synthesis of allenes 67

4.1.1 NMR spectra of (1-adamantyl)allene 69

5 Catalytic allylation of Nitriles 74

5.1 General Procedure for the catalysis experiments 74

5.2 General Procedure for the rearrangement of the allylimine borane complex to the vinylimine borane complex 74

5.2.1 2D TLC experiment of the isomerization of **13** to **13’** 75

5.3 Synthesis and characterization of allylimine borane complexes and the corresponding vinylimine borane complexes 77

5.3.1 3-Phenylpent-4-en-2-imine tris(perfluorophenyl)borane complex **13** 77

5.3.2 (*E*)-3-Phenylpent-3-en-2-imine tris(perfluorophenyl)borane complex **13’** 77

5.3.3 3-(4-(chloro)phenyl)pent-4-en-2-imine tris(perfluorophenyl)borane complex **14** 78

5.3.4 (*E*)-3-(4-chlorophenyl)pent-3-en-2-imine tris(perfluorophenyl)borane complex **14’** ... 79

5.3.5 3-(4-(Trifluoromethyl)phenyl)pent-4-en-2-imine tris(perfluorophenyl)borane complex **15** 80

5.3.6 (*E*)-3-(4-(trifluoromethyl)phenyl)pent-3-en-2-imine tris(perfluorophenyl)borane complex **15’** 80
5.3.7 3-(4-(tert-Butyl)phenyl)pent-4-en-2-imine tris(perfluorophenyl)borane complex **16**.. 81
5.3.8 (*E*)-3-(4-(tert-butyl)phenyl)pent-3-en-2-imine tris(perfluorophenyl)borane complex **16’** 82
5.3.9 3-Cyclohexylpent-4-en-2-imine tris(perfluorophenyl)borane complex **17** 82
5.3.10 3-vinylnonan-2-imine tris(perfluorophenyl)borane complex **18** 83
5.3.11 (*E*)-3-ethylidenenonan-2-imine tris(perfluorophenyl)borane complex **18’** 84
5.3.12 3-(1-Adamantyl)pent-4-en-2-imine tris(perfluorophenyl)borane complex **19** 84
5.4 Additional NMR spectra of catalysis products 86
5.4.1 3-phenylpent-4-en-2-imine tris(perfluorophenyl)borane complex **13** 86
5.4.2 (*E*)-3-phenylpent-3-en-2-imine tris(perfluorophenyl)borane complex **13’**........ 95
5.4.3 3-(4-(chloro)phenyl)pent-4-en-2-imine tris(perfluorophenyl)borane complex **14**..... 105
5.4.4 (*E*)-3-(4-chlorophenyl)pent-3-en-2-imine tris(perfluorophenyl)borane complex **14’**. 112
5.4.5 3-(4-(Trifluoromethyl)phenyl)pent-4-en-2-imine tris(perfluorophenyl)borane complex **15** 119
5.4.6 (*E*)-3-(4-(trifluoromethyl)phenyl)pent-3-en-2-imine tris(perfluorophenyl)borane complex **15’** 126
5.4.7 3-(4-(tert-butyl)phenyl)pent-4-en-2-imine tris(perfluorophenyl)borane complex **16** 133
5.4.8 (*E*)-3-(4-(tert-butyl)phenyl)pent-3-en-2-imine tris(perfluorophenyl)borane complex **16’** 140
5.4.9 3-cyclohexylpent-4-en-2-imine tris(perfluorophenyl)borane complex **17**........ 147
5.4.10 3-vinylnonan-2-imine tris(perfluorophenyl)borane complex **18** 154
5.4.11 (*E*)-3-ethylidenenonan-2-imine tris(perfluorophenyl)borane complex **18’** 161
5.4.12 3-(adamantan-1-yl)pent-4-en-2-imine tris(perfluorophenyl)borane complex **19** 168
6 Mechanistic investigations 175
6.1 Consecutive H_2 activation and hydroboration of phenylallene starting from pyridonate borane **3** 175
6.2 Stochiometric synthesis of β-diketiminate borane complex **9** from pyridonate borane **3**, phenylallene, and acetonitrile........ 183
6.3 Stochiometric synthesis of β-diketiminate borane complex **9** from ketiminoborane pyridone complex **12**, pyridonate borane **3** and acetonitrile........ 187
6.4 Transformation of the kinetic to the thermodynamic product........ 196
7 X-ray analysis........ 200
7.1 Crystallographic data collection and processing........ 200
7.2 Allylimin trisperfluorphenyl borane complex **13**........ 200
7.3 β-diketiminate borane complex **9** 211

7.4 Vinylimin borane complex **13’** ... 224
8 Computational details ... 234
9 References ... 263

2 General Information

All manipulations with air and moisture sensitive compounds were carried out under a nitrogen atmosphere using standard Schlenk techniques or in a nitrogen filled glovebox (MBraun UNILAB plus).

6-*tert*-butyl-2-pyridone **5** was synthesized from 3,3'-dimethyl-2-butanone, according to a literature known procedure.[1]

$B(C_6F_5)_3$ was synthesized from boron trifluoride etherate, according to literature procedures.[2]

Piers Borane **6** was synthesized from $B(C_6F_5)_3$ according to a literature procedure.[3]

All dry, non-deuterated solvents were if commercially available purchased by Acros Organics or Sigma Aldrich in a sealed bottle with a septum and stored (except for benzene) over molecular sieves. Deuterated solvents were distilled under inert conditions and kept in a glovebox over molecular sieves.

Cyclohexylallene was commercially purchased from Sigma Aldrich, subject to three pump-thaw cycles, and stored in a glovebox over molecular sieves.

Reaction under microwave irradiation were performed in a Discover SP microwave from CEM.

Flash column chromatography was carried out with Silica 60 M (0.04–0.063 mm) from Macherey-Nagel GmbH&Co. KG. Thin layer chromatography was carried out on Polygram®SIL G/UV254 from Macherey-Nagel GmbH&Co. KG. Solvents for column chromatography were distilled bulb to bulb prior to use.

NMR spectra were recorded on Bruker Avance II 200 MHz, Bruker Avance III HD 400 MHz, Bruker Avance II 400 MHz, and Bruker Avance III HD 600 MHz spectrometers. ^{1}H and ^{13}C NMR chemical shifts are referenced to residual solvent resonance peaks. ^{15}N spectra are referenced to NH_3 (0 ppm).

IR spectra were recorded on a Bruker Vertex 70 FT-IR spectrometer.

Mass spectra were recorded on an ESI-MS- Bruker Mikro-TOF mass spectrometer. Samples were dissolved in methanol. In positive ion detection mode the capillary current was set to 4500 V with the end plate offset of –500 V, and in negative mode the capillary current was set to 3000 V with the end plate offset of 500 V.

UV-Vis spectra were recorded with a SPECORD® 200 PLUS spectrophotometer equipped with two automatic eight-fold cell changers and a Peltier element thermostat system (0.1 °C accuracy) by Analytik Jena. The system was operated with the ASpect UV software by Analytik Jena. The sample solutions were measured in QS High Precision Cells made of Quartz Suprasil® by Hellma Analytics with a light path of 10 mm. Cyclohexane for UV-Vis spectroscopy was purchased from Merck (Uvasol®quality).

Fluorescence spectra were recorded with a FP-8300 fluorescence spectrophotometer from Jasco. The sample solutions were measured in QS High Precision Cells made of Quartz Suprasil® by Hellma Analytics with a light path of 10×10 mm. Cyclohexane for fluorescence spectroscopy was purchased from Merck (Uvasol®quality). The system was operated with SpectraManager V2 software by Jasco. The following parameters were used:

Ex bandwidth: 2.5 nm
Em bandwidth: 2.5 nm

Response:	0.5 sec
Sensitivity:	Medium
Data interval:	1 nm
Ex wavelength:	359.0 nm
Scan speed:	500 nm/min

3 Synthesis and characterization of mechanistically relevant intermediates

3.1 Synthesis and characterization of cinnamylbis(perfluorophenyl)borane pyridone complex 7

A stock solution of pyridone **5** and Piers borane **6** (300 µL) (0.1 M pyridone **5** and 0.11 M Piers borane **6** in benzene-d_6) was transferred to a 60 mL flask with J. Young valve. Benzene was added (1.0 mL). The flask was taken out of the glovebox and subject to three freeze-pump-thaw cycles. Then, the reaction was heated under passive vacuum to 60 °C overnight. The next day phenylallene (3.8 µL, 0.03 mmol) was added inside the glovebox. The flask was again subject to three freeze-pump-thaw cycles, pressurized with H_2 (1.1 bar) and stirred overnight at room temperature The solvent was evaporated under high vacuum and 1,3,5-trimethoxybenzene (0.09 mmol, 50 µL of a 0.18 M stock solution in benzene-d_6) was added and a ^{1}H and ^{11}B NMR spectrum were measured (Figure SI 1, Figure SI 2, Figure SI 3, Figure SI 4).

The allylborane pyridone complex **7** is the main product with an *in situ* yield of 66%.

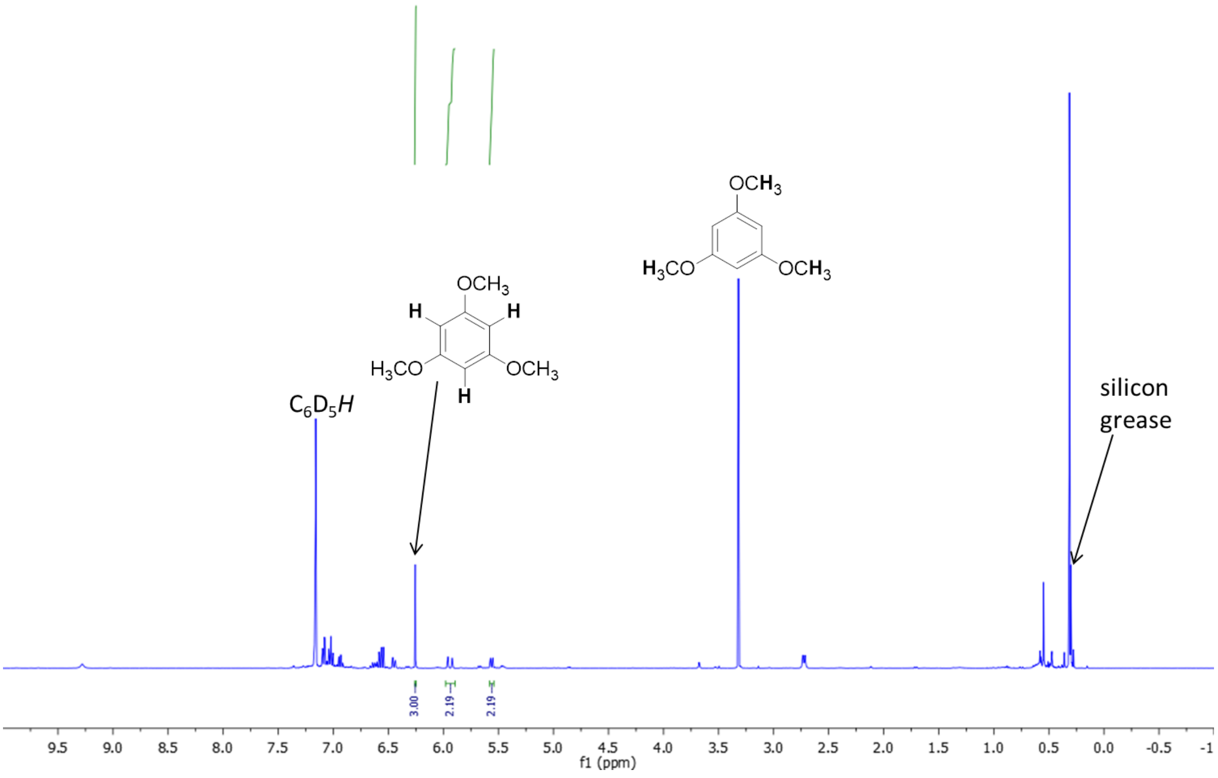

Figure SI 1: ^{1}H NMR spectrum of a reaction mixture of pyridonate borane **3** and phenylallene after 1 d at room temperature under H_2 pressure (1.1 bar) in a 60 mL flask with J. Young valve and 1,3,5-trimethoxybenzene as an internal standard for quantification (400 MHz, benzene-d_6).

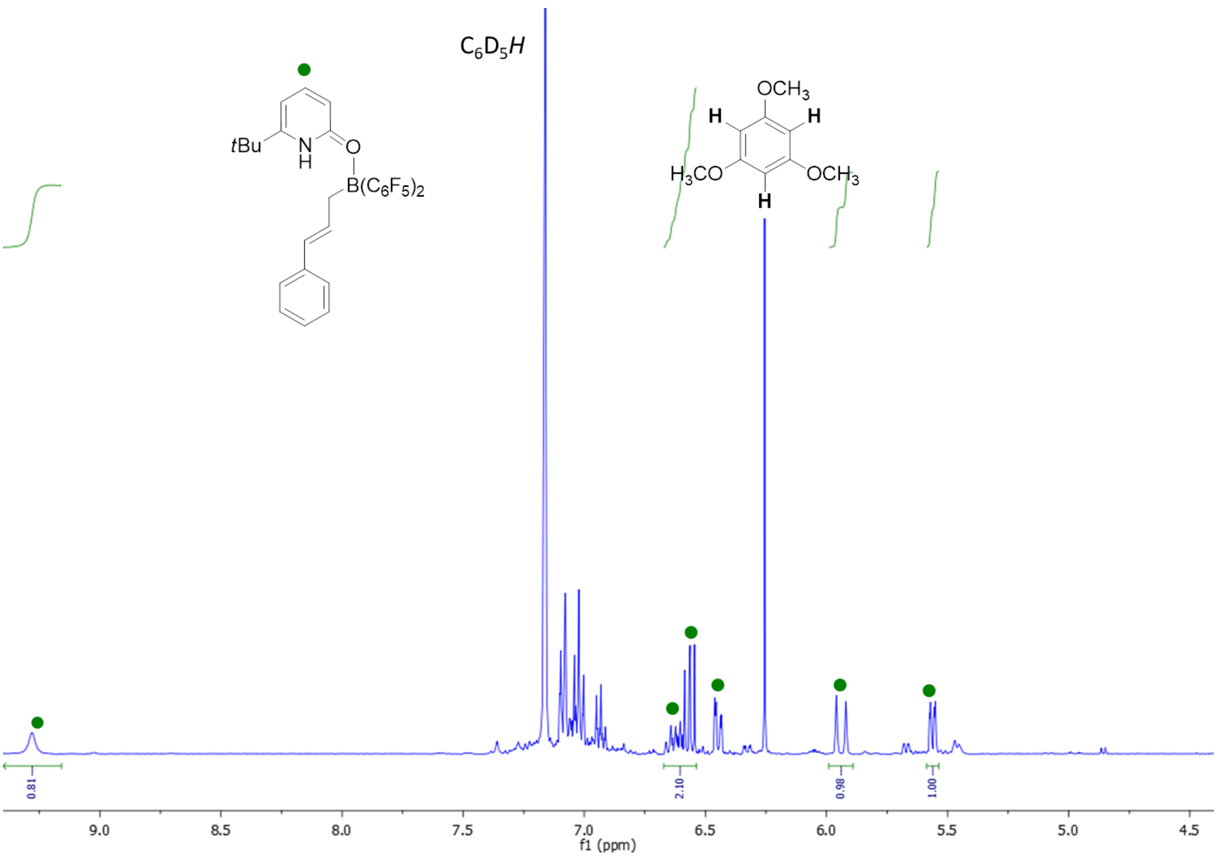

Figure SI 2: Low field excerpt ^{1}H NMR spectrum of a reaction mixture of pyridonate borane **3** and phenylallene after 1 d at room temperature under H_2 pressure (1.1 bar) in a 60 mL flask with J. Young valve and 1,3,5-trimethoxybenzene as an internal standard for quantification (400 MHz, benzene-d_6).

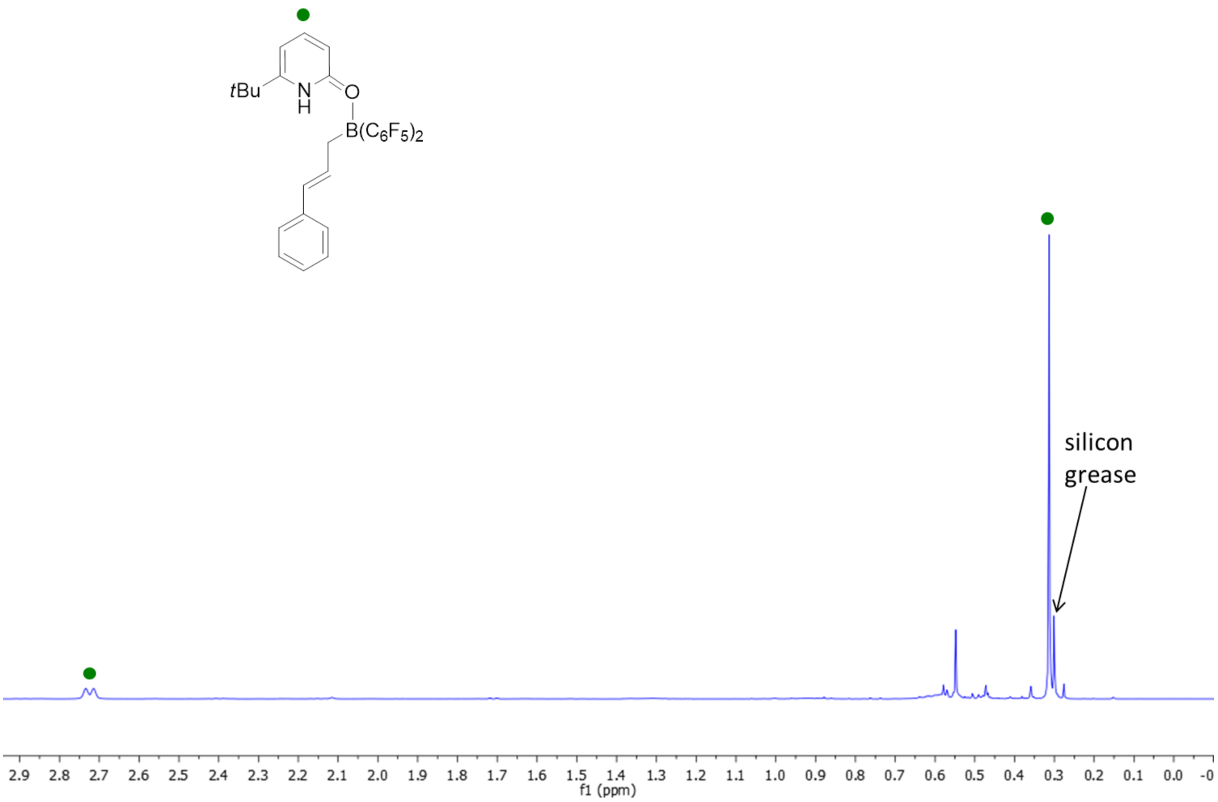

Figure SI 3: High field excerpt ^{1}H NMR spectrum of a reaction mixture of pyridonate borane **3** and phenylallene after 1 d at room temperature under H_2 pressure (1.1 bar) in a 60 mL flask with J. Young valve and 1,3,5-trimethoxybenzene as an internal standard for quantification (400 MHz, benzene-d_6).

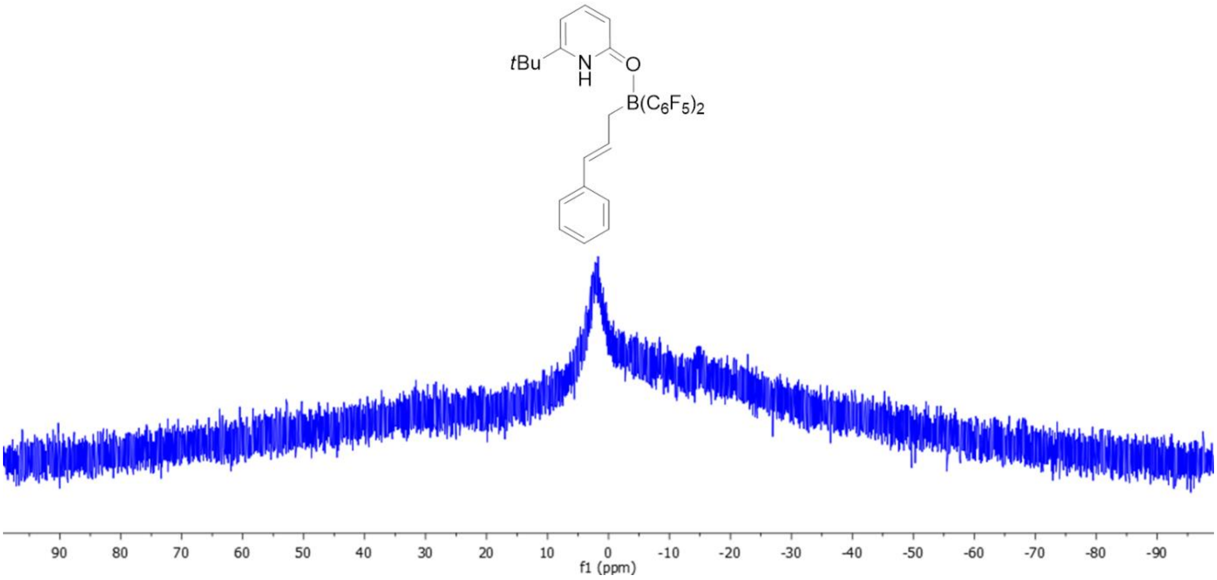

Figure SI 4: ^{11}B NMR spectrum of a reaction mixture of pyridonate borane **3** and phenylallene after 1 d at room temperature under H_2 pressure (1.1 bar) in a 60 mL flask with J. Young valve and 1,3,5-trimethoxybenzene as an internal standard for quantification (128 MHz, benzene-d_6).

To verify formation of **7** and for full characterization, **7** was synthesized stepwise by hydroboration of phenylallene with Piers borane **6** and subsequent addition of pyridone **5** (chapter 3.3).

3.2 Synthesis and characterization of cinnamylbis(perfluorophenyl)borane 8

The reaction was performed in a nitrogen filled glovebox. Piers borane **6** (17.3 mg, 0.05 mmol, 1.00 equiv.) was dissolved in dry diethylether (1.00 mL) and was kept at –35 °C for one hour. In a separate vial phenylallene (6.4 µL, 0.05 mmol, 1.00 equiv.) was dissolved in dry diethylether (0.50 mL) and kept at –35 °C for one hour. Both solutions were mixed and kept at –35 °C for 6 d. The diethylether was removed by letting the open reaction vessel stand at room temperature for 3 h. The residue was dissolved in benzene-d_6 (0.40 mL) and transferred to an NMR tube with J. Young valve and rinsed two times with benzene-d_6 (0.10 mL).

After the characterization was complete the amount of cinnamylbis(perfluorophenyl)borane **8** was quantified *in situ* by adding a stock solution of 1,3,5-trimethoxybenzene (50 µL of a 0.5 M solution in benzene-d_6, 0.025 mmol) (Figure SI 16). The yield determined by qNMR was 81%.

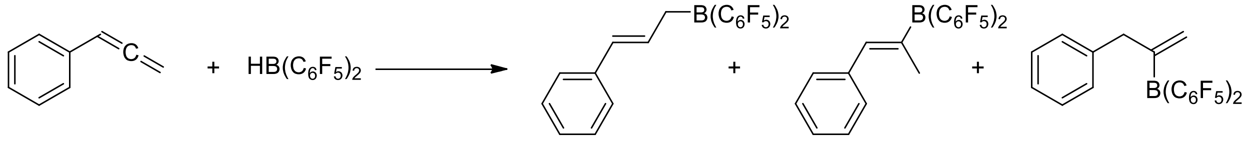

Scheme SI 1: Quantification of the regioisomers of the hydroboration of phenylallene with Piers borane **6**.

^{1}H NMR (400 MHz, benzene-d_6) δ 7.23 – 7.18 (m, 2H, Ph-*H*), 7.09 – 7.04 (m, 2H, Ph-*H*), 7.02 – 6.95 (m, 1H, Ph-*H*), 6.37 (d, *J* = 15.7 Hz, 1H, bn-*H*), 6.09 (dt, *J* = 15.7, 7.7 Hz, 1H, *H*C-CH$_2$B), 2.77 (d, *J* = 7.7 Hz, 2H, C*H*$_2$B).

Remark: Although **8** is the main species two other regioisomers were tentatively assigned by comparing to the reported signals by Erker *et al.*[4] The ratio of cinnamylbis(perfluorophenyl)borane **8** to the other two identifiable regioisomers is 17:4 (Figure SI 6 and Figure SI 7).

^{13}C{^{1}H} NMR (101 MHz, benzene-d_6) δ 137.8 (Ph-*C*$_q$), 135.6 (bn-*C*), 129.0 (Ph-*C*), 127.9 (Ph-*C*), 126.3 (Ph-*C*), 121.6 (C=*C*H-CH$_2$B) 34.8 (*C*H$_2$B).

Remark: The ^{13}C{^{1}H} NMR spectrum shows broad signals with low intensity at 149.0, 146.5, 144.6, 142.1 138.9, 136.4 ppm which can be tentatively assigned to the pentafluorophenyl groups of the different hydroboration products.

^{11}B NMR (128 MHz, benzene-d_6) δ 57.6.

^{19}F NMR (377 MHz, benzene-d_6) δ -129.81 (dt, *J* = 22.2, 6.2 Hz, o-*F*), -147.75 (t, *J* = 17.7 Hz, p-*F*), -161.22 (td, *J* = 22.1, 7.7 Hz, m-*F*).

3.2.1 NMR spectra of cinnamylbis(perfluorophenyl)borane 8

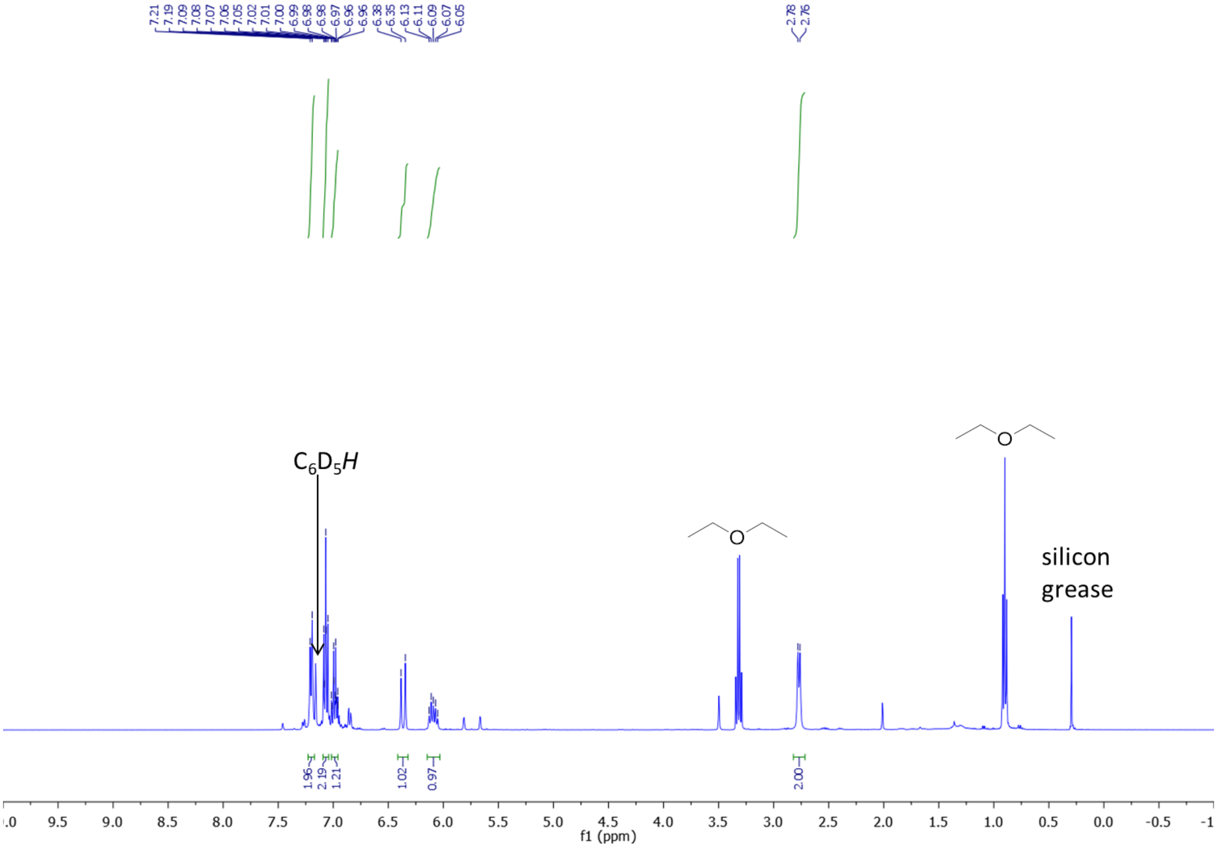

Figure SI 5: ^{1}H NMR of the reaction mixture containing allylborane **8** as main species and other regioisomers of the hydroboration of phenylallene with Piers borane (400 MHz, benzene-d_6).

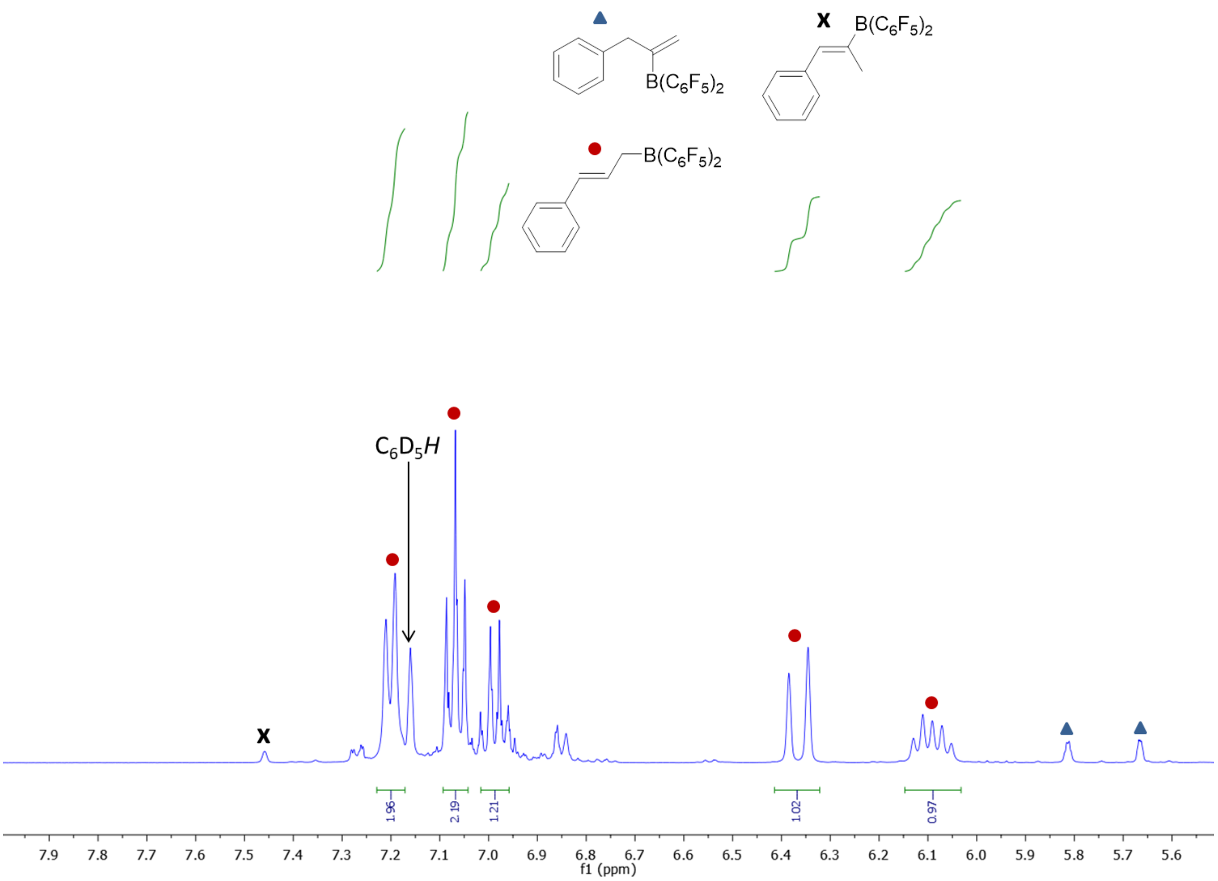

Figure SI 6: Low field excerpt of the ^{1}H NMR of the reaction mixture containing allylborane **8** as main species (red dot) and other regioisomers (blue up-pointing triangle and black cross) of the hydroboration of phenylallene with Piers borane (400 MHz, benzene-d_6).

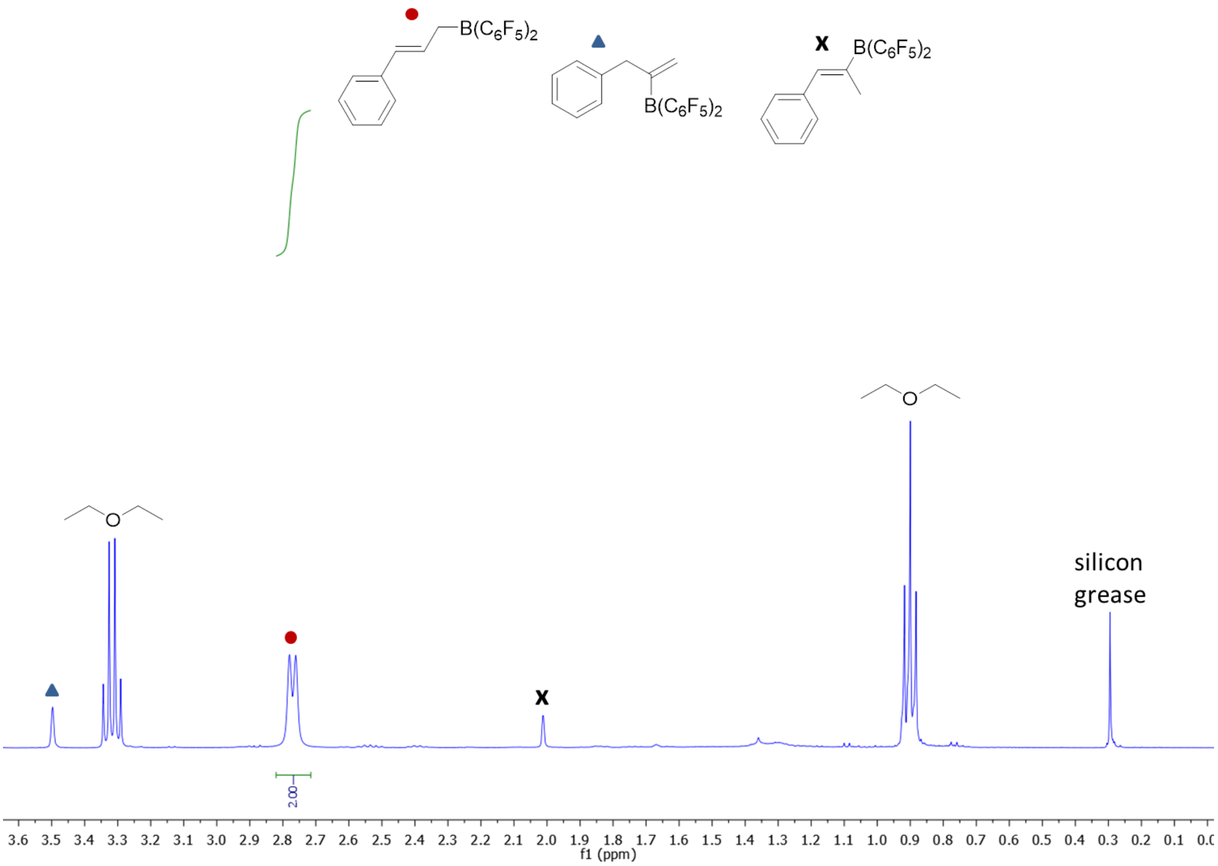

Figure SI 7: High field excerpt of the ^{1}H NMR of the reaction mixture containing allylborane **8** as main species (red dot) and other regioisomers (blue up-pointing triangle and black cross) of the hydroboration of phenylallene with Piers borane (400 MHz, benzene-d_6).

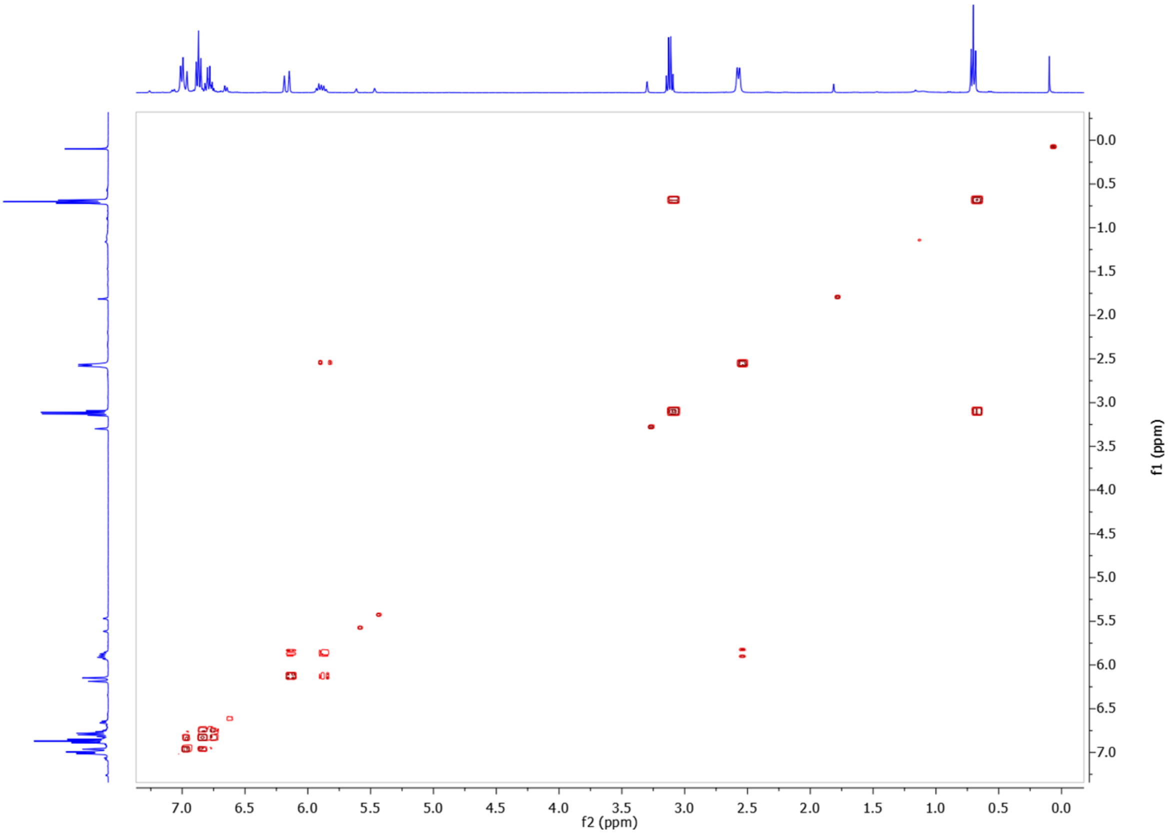

Figure SI 8: COSY NMR of the reaction mixture containing allylborane **8** as main species and other regioisomers of the hydroboration of phenylallene with Piers borane (400 MHz, benzene-*d*$_6$).

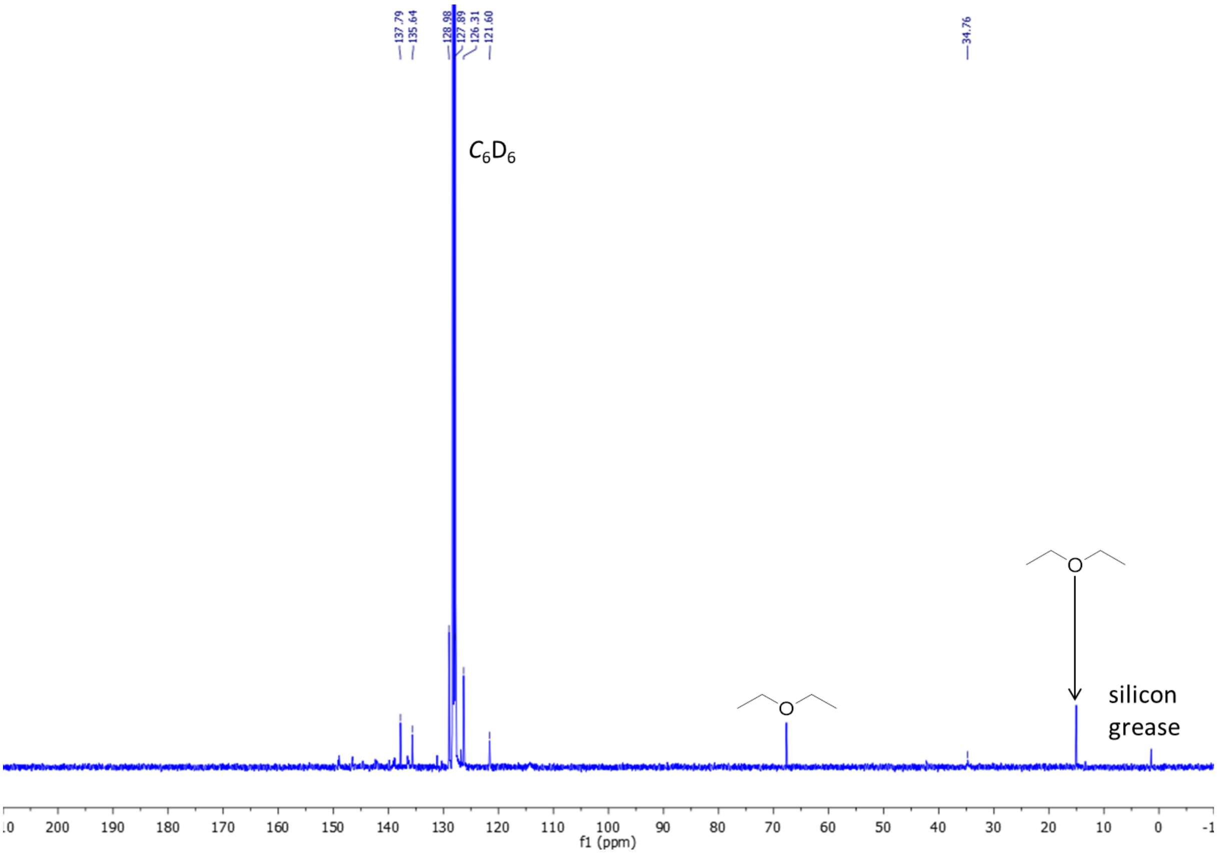

Figure SI 9: $^{13}C\{^{1}H\}$ NMR spectrum of the reaction mixture containing allylborane **8** as main species and other regioisomers of the hydroboration of phenylallene with Piers borane (101 MHz, benzene-d_6).

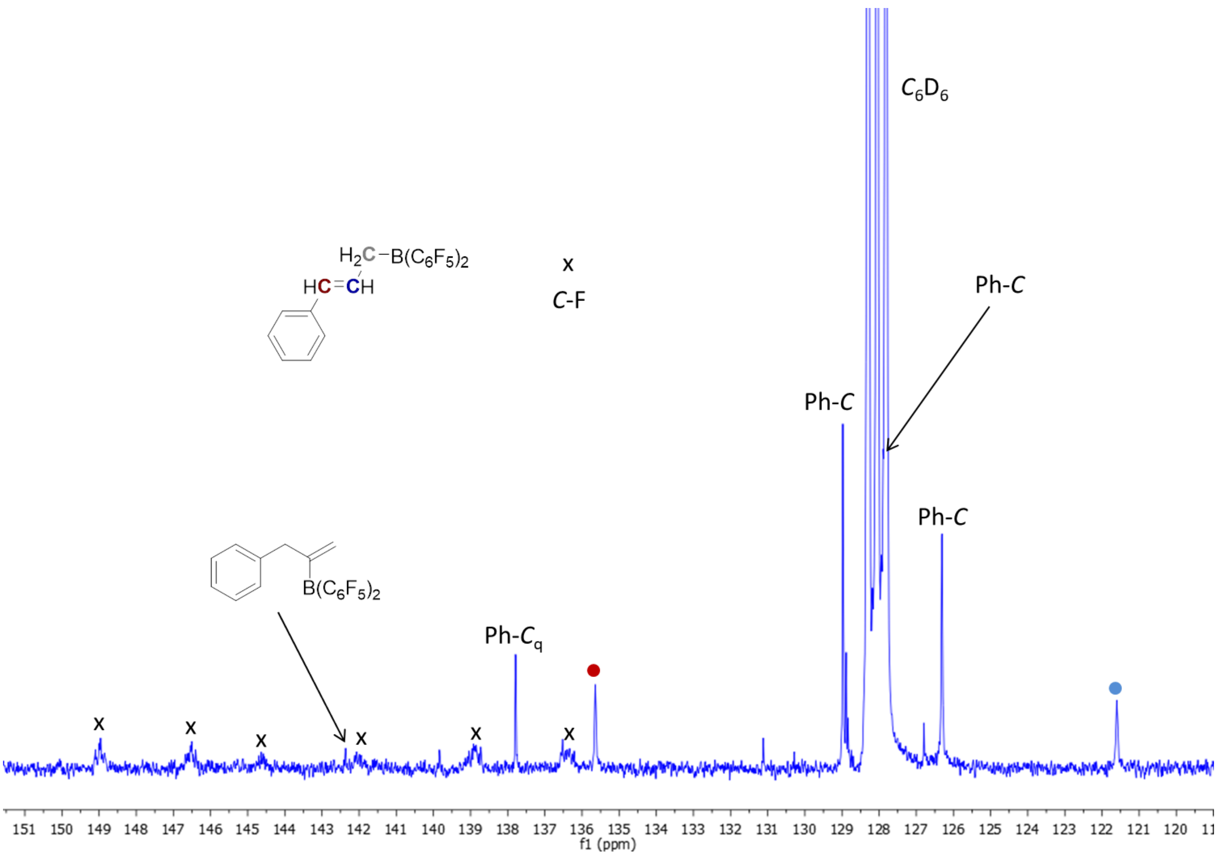

Figure SI 10: Low field excerpt of the $^{13}C\{^{1}H\}$ NMR spectrum of the reaction mixture containing allylborane **8** (marked peaks) as main species and other regioisomers of the hydroboration of phenylallene with Piers borane (101 MHz, benzene-d_6).

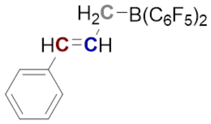

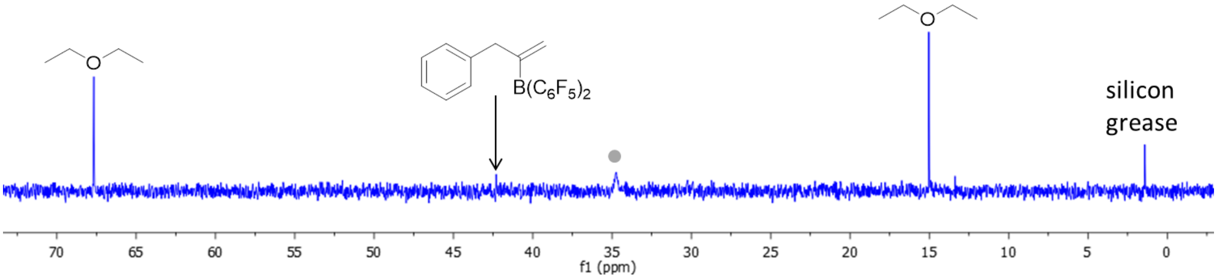

Figure SI 11: High field excerpt of the $^{13}C\{^{1}H\}$ NMR spectrum of the reaction mixture containing allylborane **8** as main species and other regioisomers of the hydroboration of phenylallene with Piers borane (101 MHz, benzene-d_6).

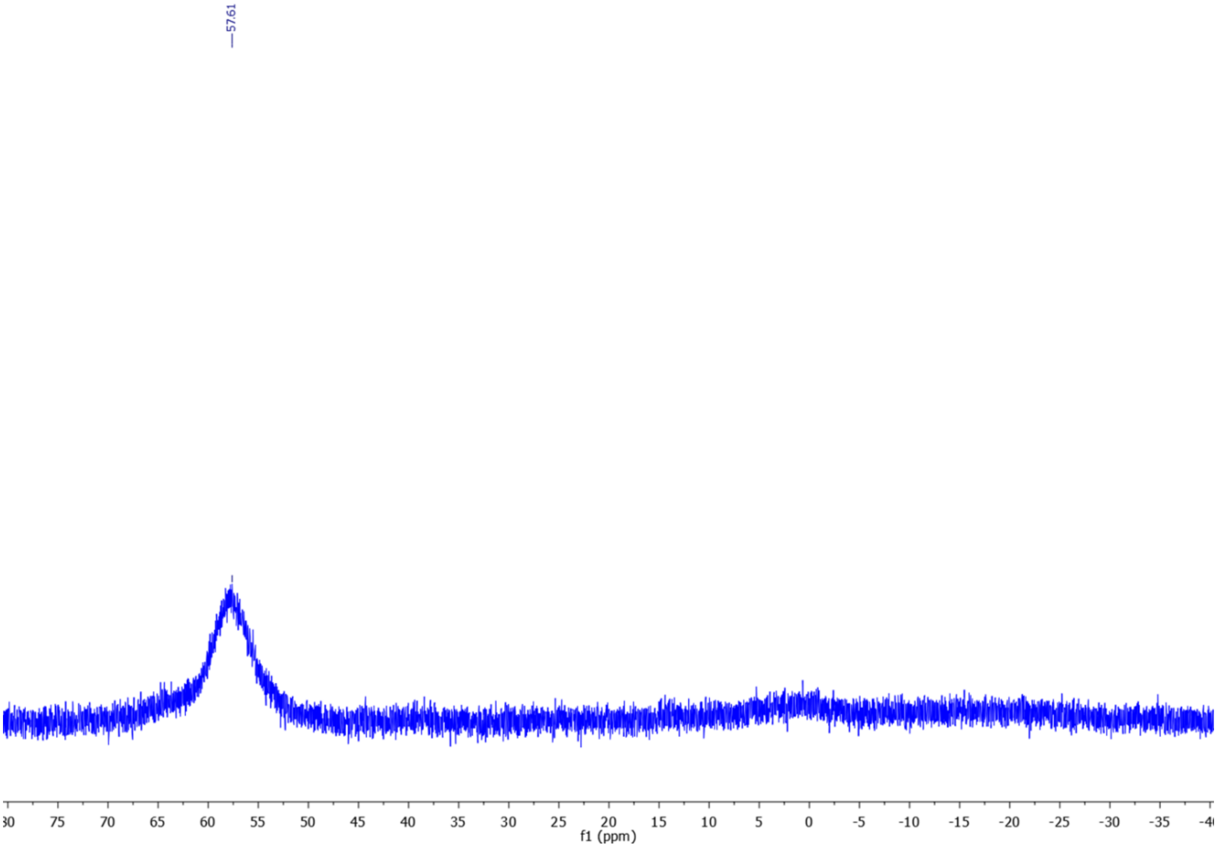

Figure SI 12: ^{11}B NMR spectrum of the reaction mixture containing allylborane **8** as main species and other regioisomers of the hydroboration of phenylallene with Piers borane (128 MHz, benzene-d_6).

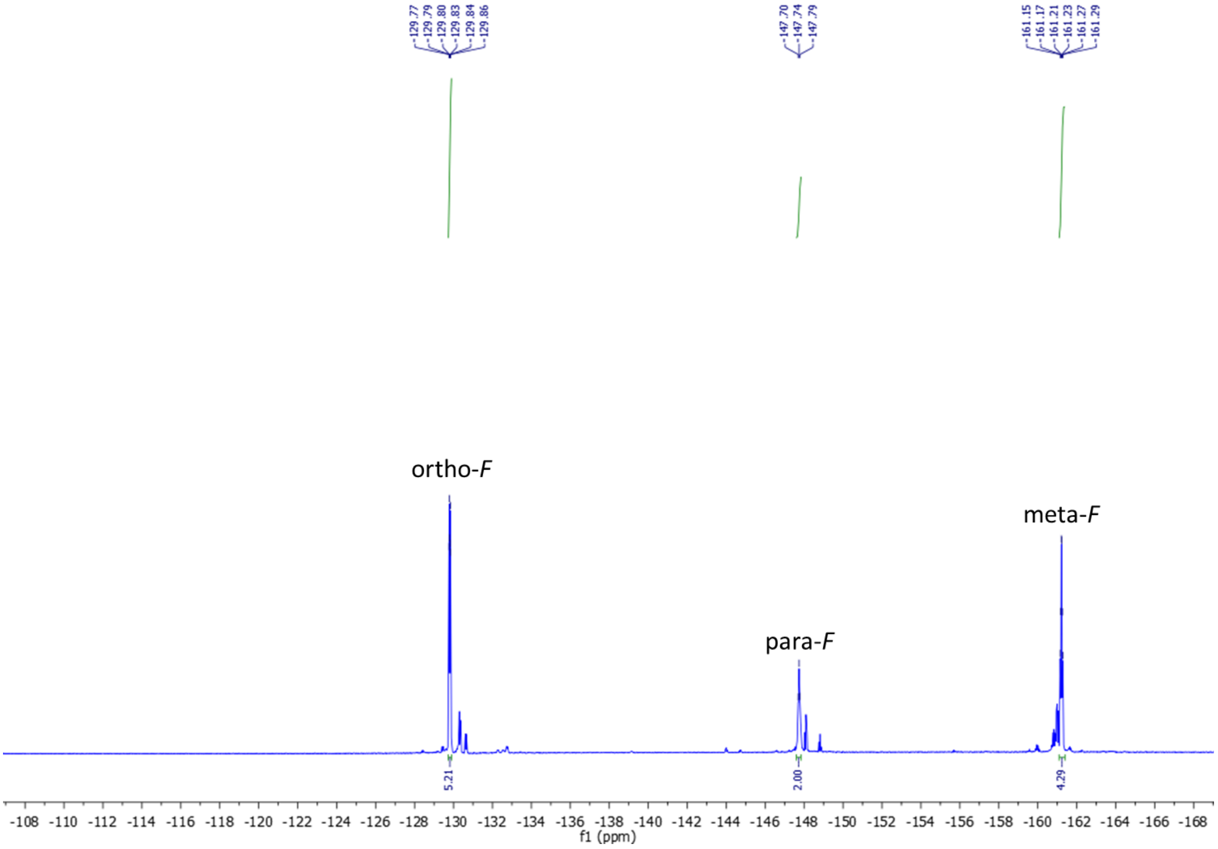

Figure SI 13: ^{19}F NMR spectrum of the reaction mixture containing allylborane **8** as main species and other regioisomers of the hydroboration of phenylallene with Piers borane (377 MHz, benzene-d_6).

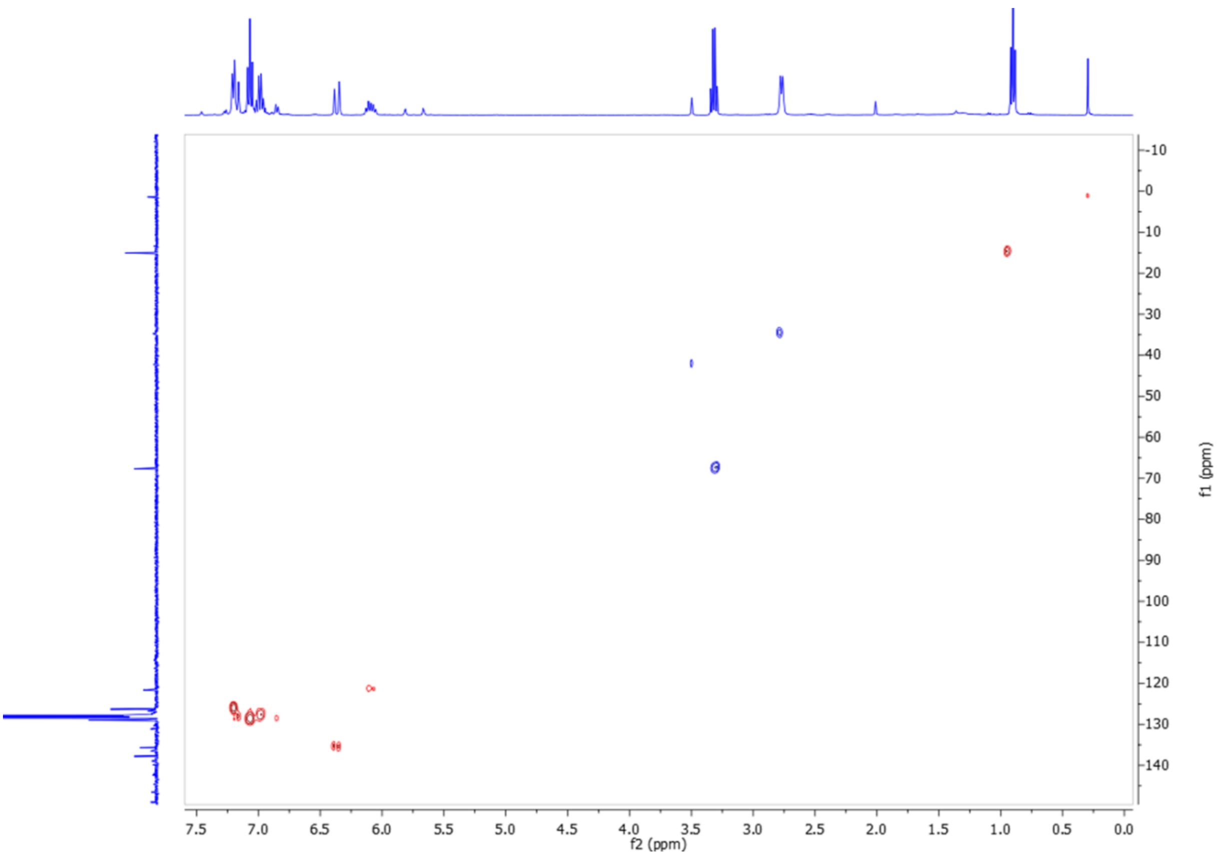

Figure SI 14: HSQC NMR spectrum of the reaction mixture containing allylborane **8** as main species and other regioisomers of the hydroboration of phenylallene with Piers borane (101 MHz, benzene-d_6).

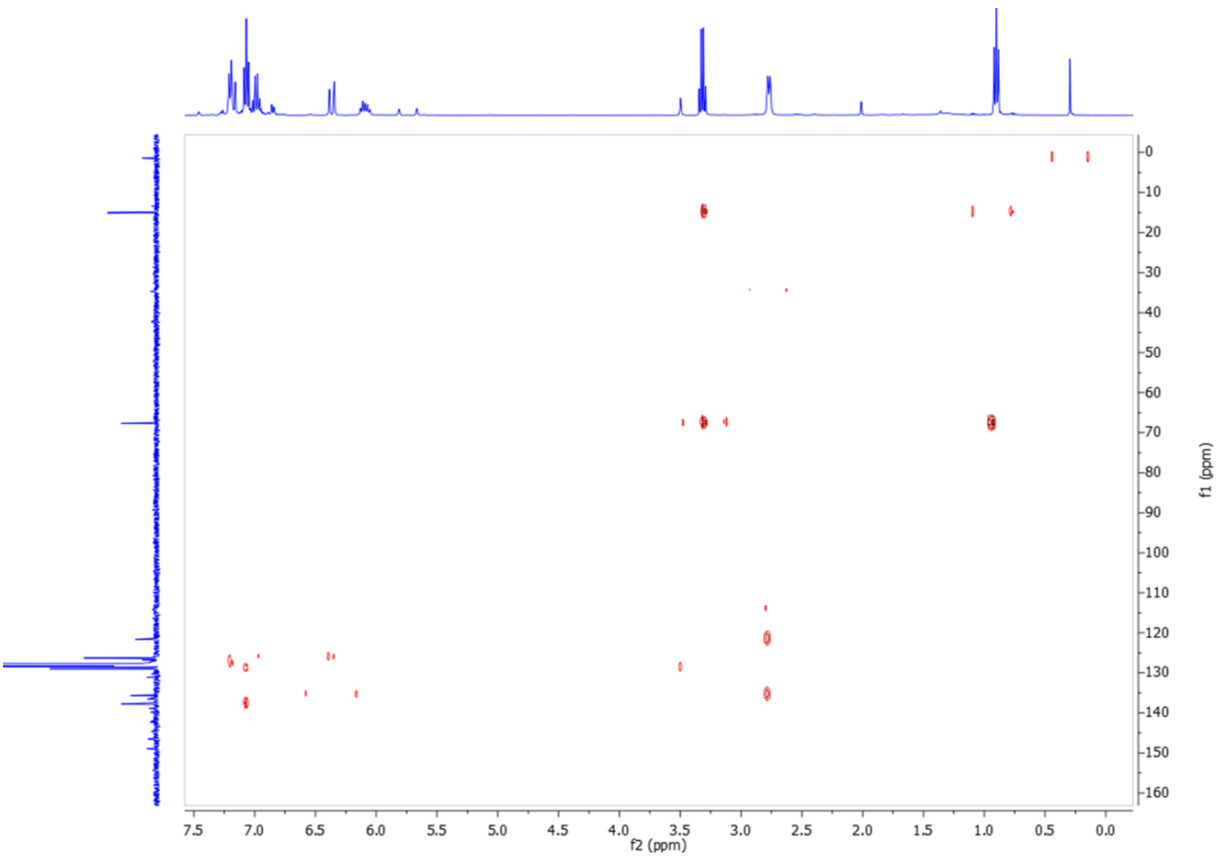

Figure SI 15: HMBC NMR spectrum of the reaction mixture containing allylborane **8** as main species and other regioisomers of the hydroboration of phenylallene with Piers borane (101 MHz, benzene-d_6).

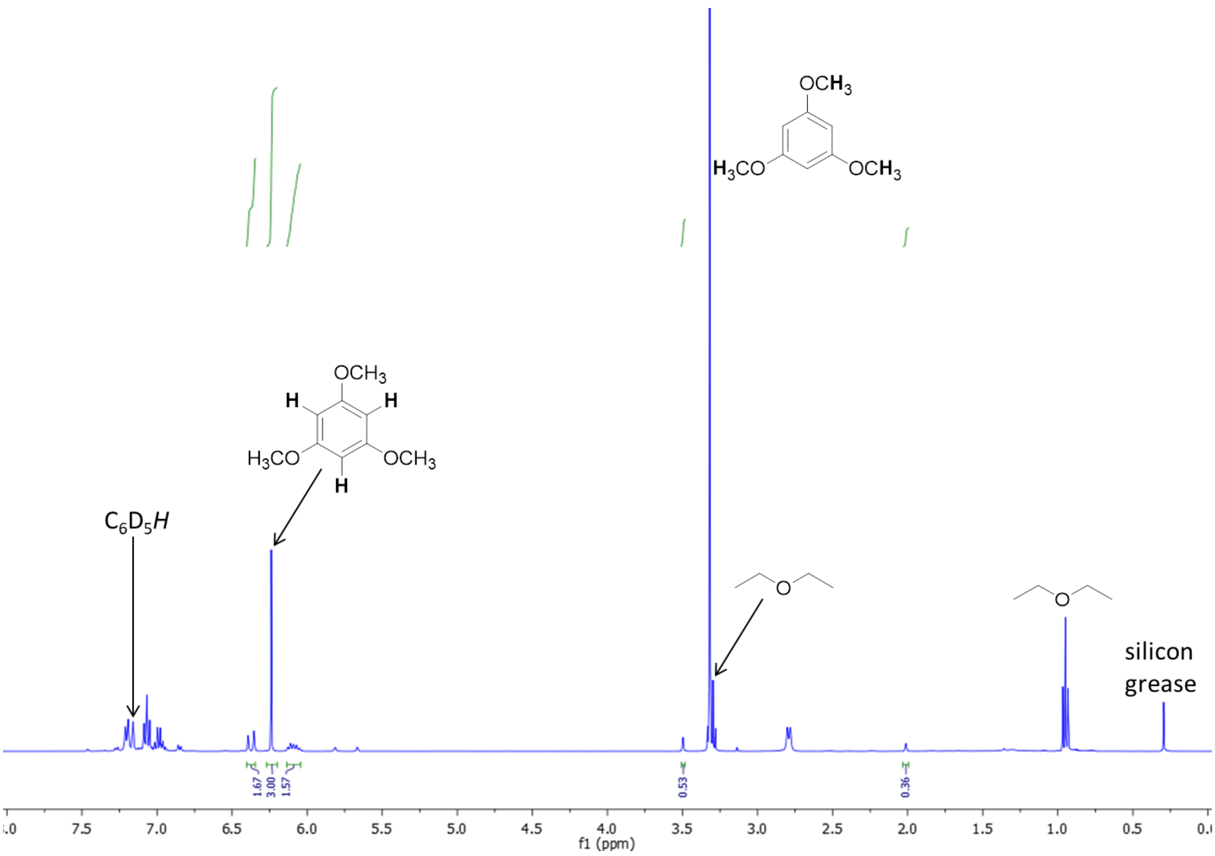

Figure SI 16: ^{1}H NMR of the reaction mixture containing allylborane **8** as main species and other regioisomers of the hydroboration of phenylallene with Piers borane with 1,3,5-trimethoxybenzene (50 µL of a 0.5 M solution in benzene-d_6, 0.025 mmol) (400 MHz, benzene-d_6).

3.3 Synthesis and characterization of cinnamylbis(perfluorophenyl)borane pyridone complex 7 from cinnamylbis(perfluorophenyl)borane 8

Cinnamylbis(perfluorophenyl)borane **8** (23.1 mg, 0.05 mmol) was synthesized as described in chapter 3.2. Afterwards pyridone **5** (7.6 mg, 0.05 mmol) was added.

After the characterization was complete the amount of the cinnamylbis(perfluorophenyl)borane pyridone complex **7** was quantified *in situ* by adding a stock solution of 1,3,5-trimethoxybenzene (50 µL of a 0.5 M solution in benzene-d_6, 0.025 mmol) (Figure SI 28).The yield determined by qNMR was 81% over two steps or quantitative starting from allylborane **8**.

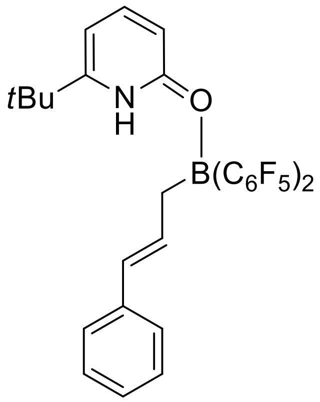

^{1}H NMR (400 MHz, benzene-d_6) δ 9.30 (s, 1H, N*H*), 7.10 – 7.01 (m, 4H, Ph-*H*), 6.96 – 6.90 (m, 1H, Ph-*H*), 6.66 – 6.56 (m, 2H, $HC_{sp2}CH_2B$), 6.46 (ddd, *J* = 9.0, 2.2, 0.9 Hz, 1H, Py-*H*), 5.93 (d, *J* = 15.7 Hz, bn-$C_{sp2}H$), 5.59 (ddd, *J* = 7.5, 2.1, 1.0 Hz, 1H, Py-*H*), 2.71 (d, *J* = 7.8 Hz, 2H, H_2CB), 0.33 (s, 9H, $C(CH_3)_3$).

^{13}C{^{1}H} NMR (101 MHz, benzene-d_6) δ 161.5 (Py-*C*), 157.7 (Py-*C*), 145.7 (Py-*C*), 139.2 (Ph-C_q), 133.9 (bn-CH*C*HCH$_2$B), 128.8 (bn-*C*), 128.7 (Ph-*C*), 126.4 (Ph-*C*), 125.7 (Ph-*C*), 114.4 (Py-*C*), 109.3 (Py-*C*), 34.4 ($C(CH_3)_3$), 30.2 (CH_2B), 27.7 ($C(CH_3)$)

^{11}B NMR (128 MHz, benzene-d_6) δ 2.22.

^{19}F NMR (377 MHz, benzene-d_6) δ -133.59 (dd, *J* = 24.8, 8.9 Hz, o-*F*), -158.30 (t, *J* = 20.6 Hz, p-*F*), -163.77 (ddd, *J* = 24.9, 20.4, 9.3 Hz, m-*F*).

3.3.1 NMR spectra of cinnamylbis(perfluorophenyl)borane pyridone complex 7

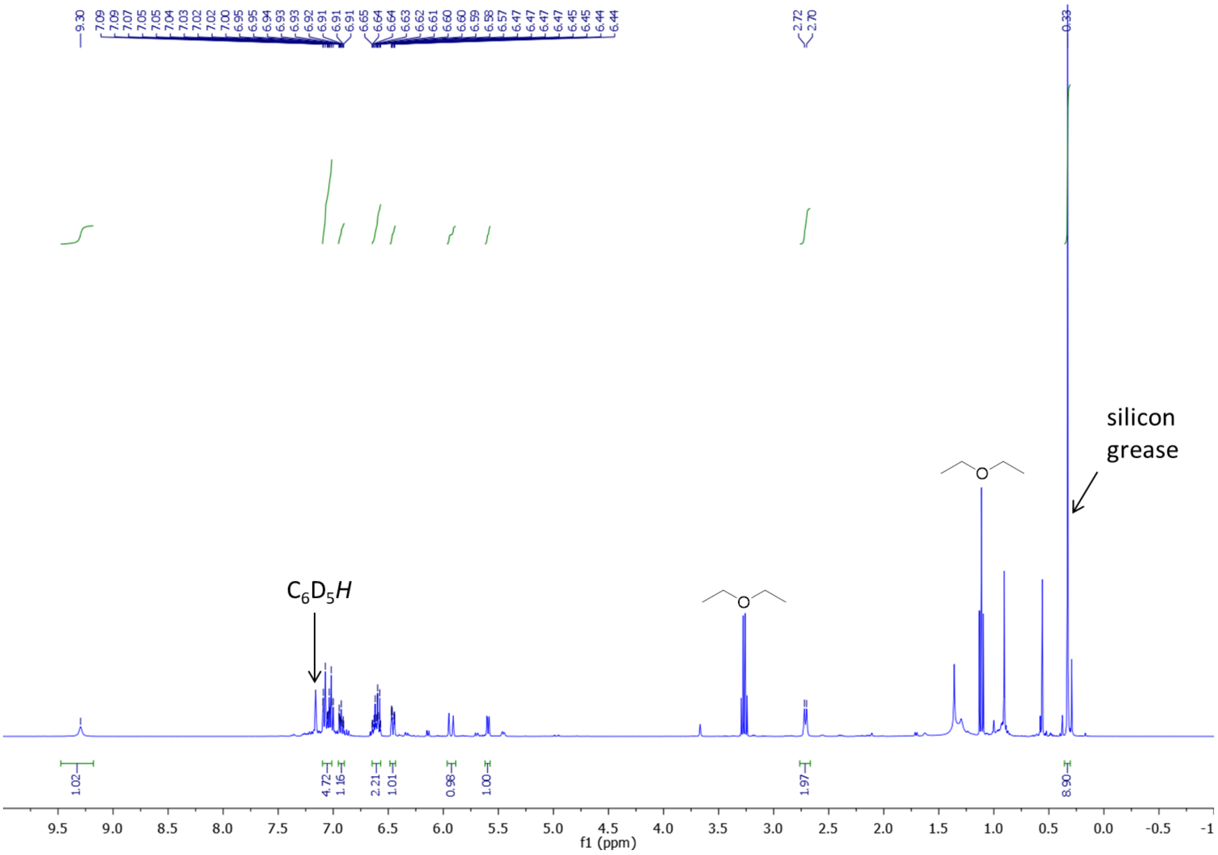

Figure SI 17: ^{1}H NMR spectrum of the cinnamylbis(perfluorophenyl)borane pyridone complex **7** (400 MHz, benzene-d_6).

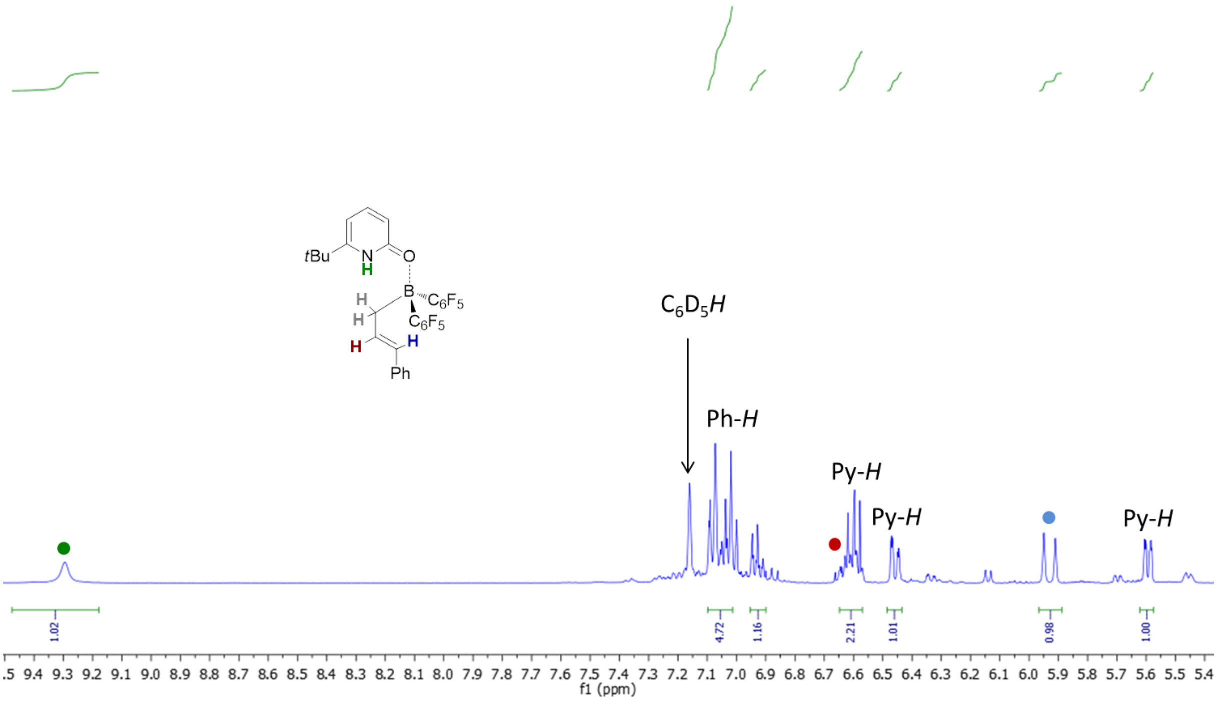

Figure SI 18: Low field excerpt of the ^{1}H NMR spectrum of the cinnamylbis(perfluorophenyl)borane pyridone complex **7** (400 MHz, benzene-d_6).

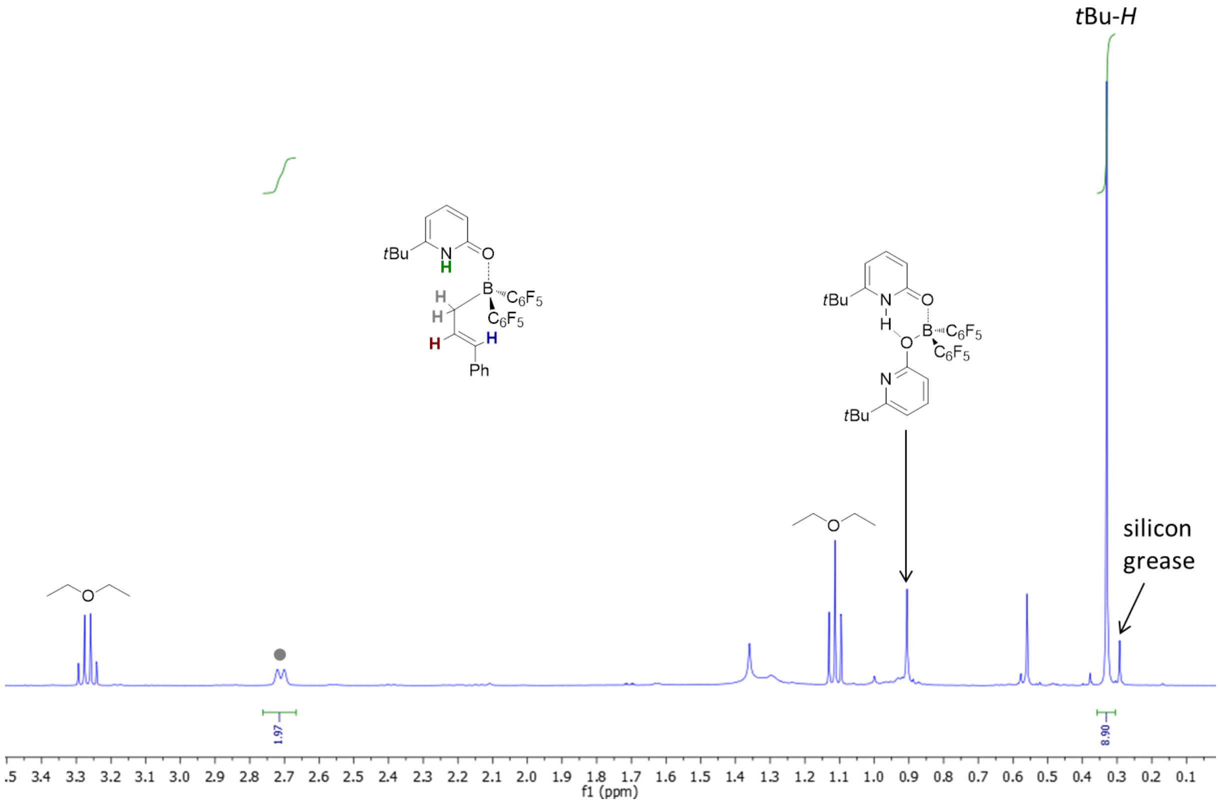

Figure SI 19: High field excerpt of the ^{1}H NMR spectrum of the cinnamylbis(perfluorophenyl)borane pyridone complex **7** (400 MHz, benzene-d_6).

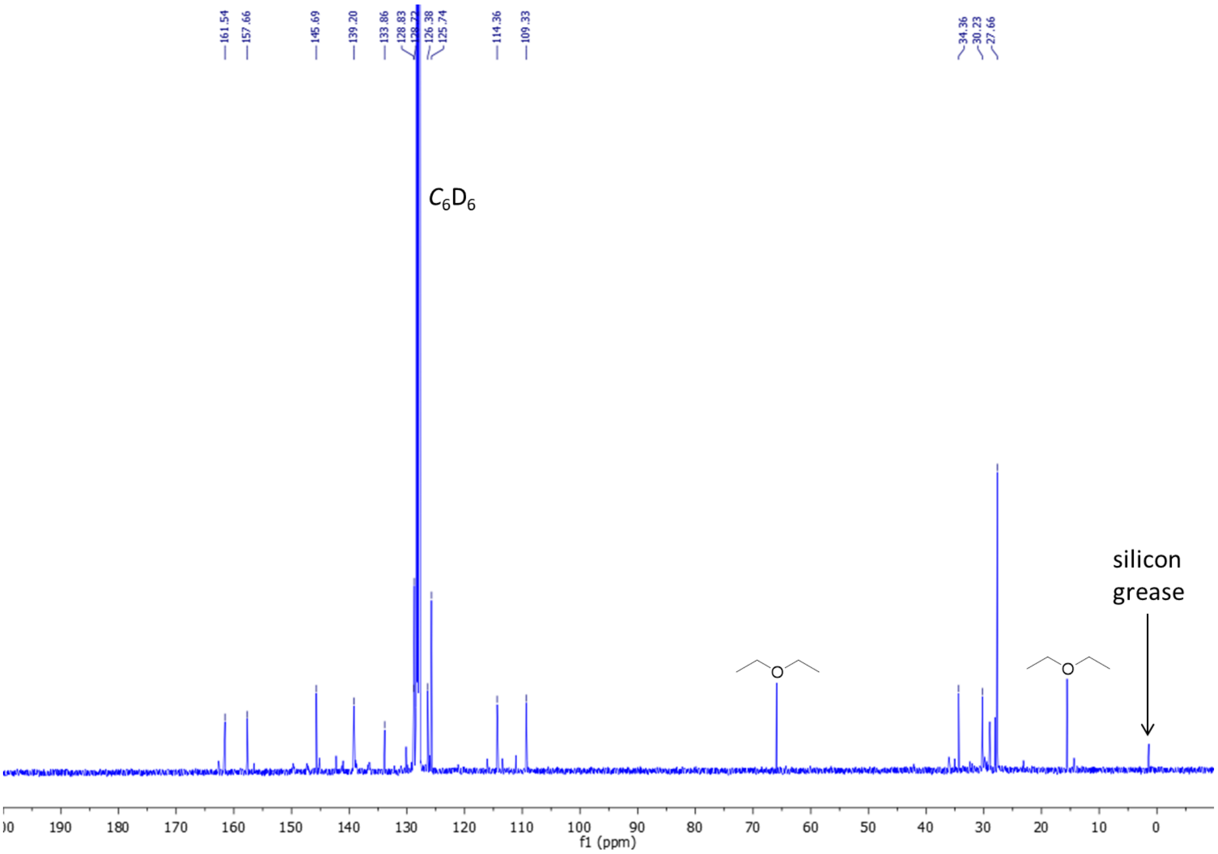

Figure SI 20: $^{13}C\{^{1}H\}$ NMR spectrum of the cinnamylbis(perfluorophenyl)borane pyridone complex **7** (101 MHz, benzene-d_6).

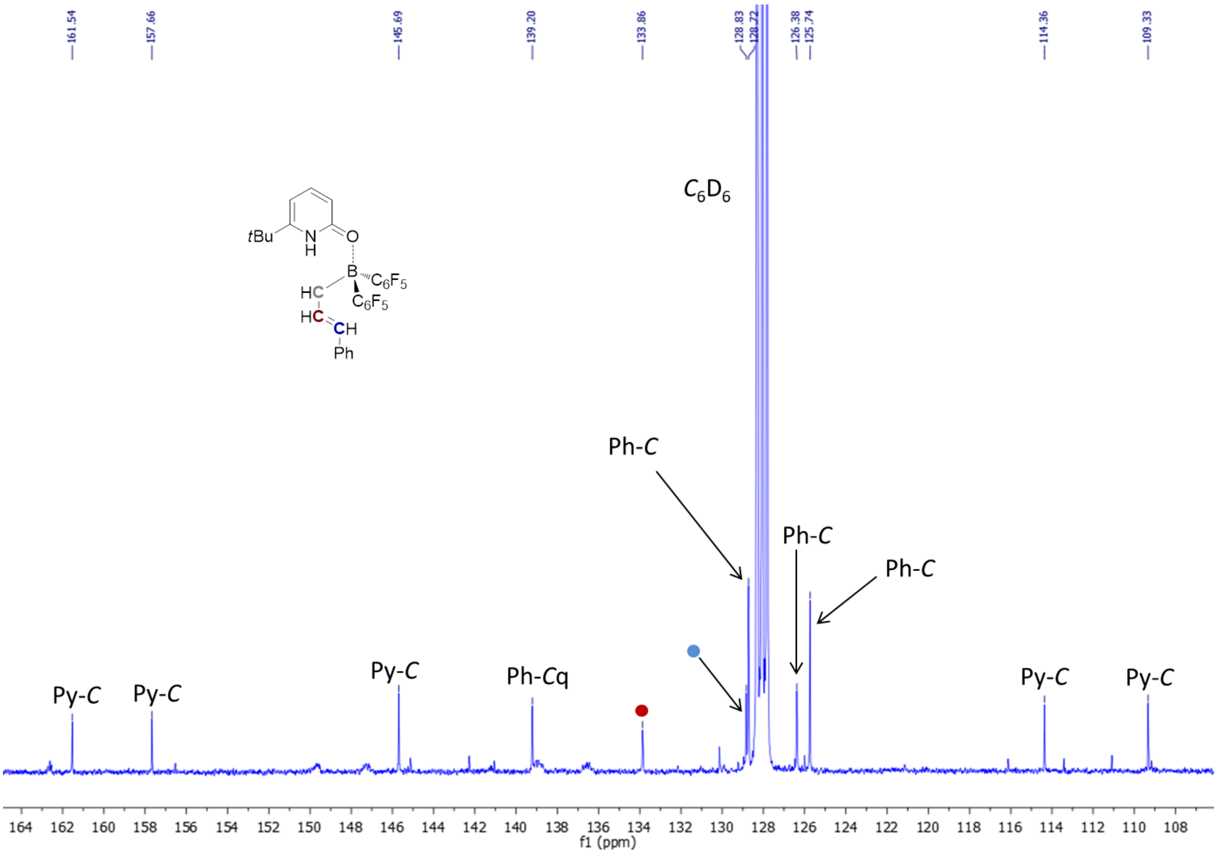

Figure SI 21: Low field excerpt of the $^{13}C\{^{1}H\}$ NMR spectrum of the cinnamylbis(perfluorophenyl)borane pyridone complex **7** (101 MHz, benzene-d_6).

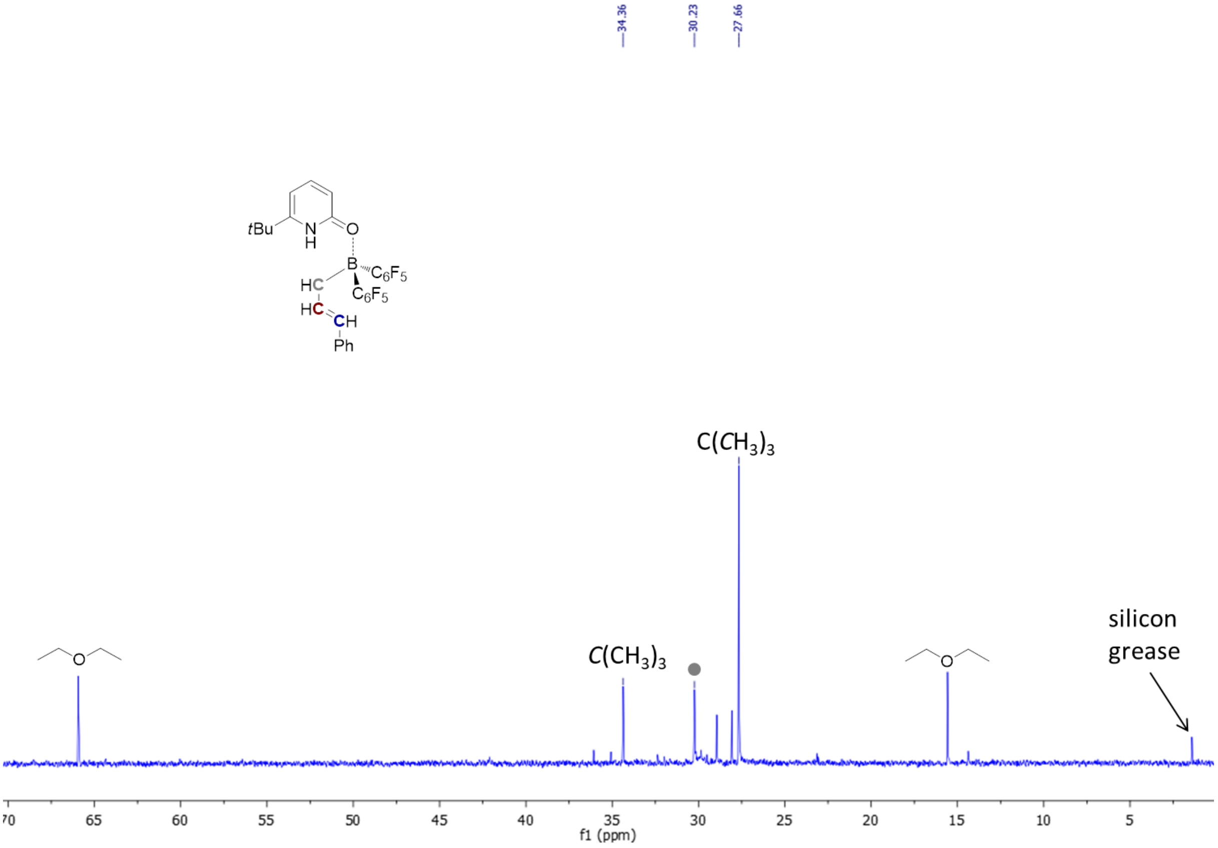

Figure SI 22: High field excerpt of the $^{13}C\{^{1}H\}$ NMR spectrum of the cinnamylbis(perfluorophenyl)borane pyridone complex **7** (101 MHz, benzene-*d*$_6$).

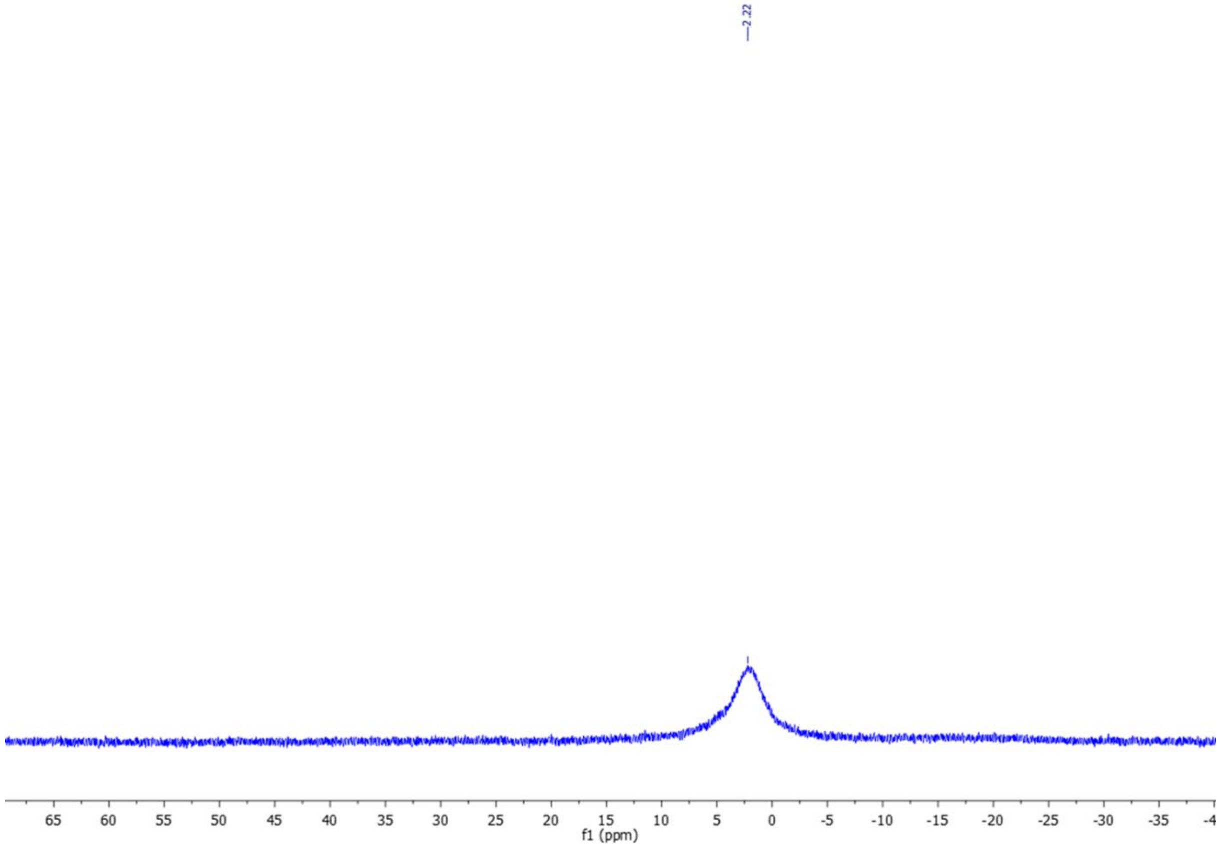

Figure SI 23: ^{11}B NMR spectrum of the cinnamylbis(perfluorophenyl)borane pyridone complex **7** (128 MHz, benzene-d_6).

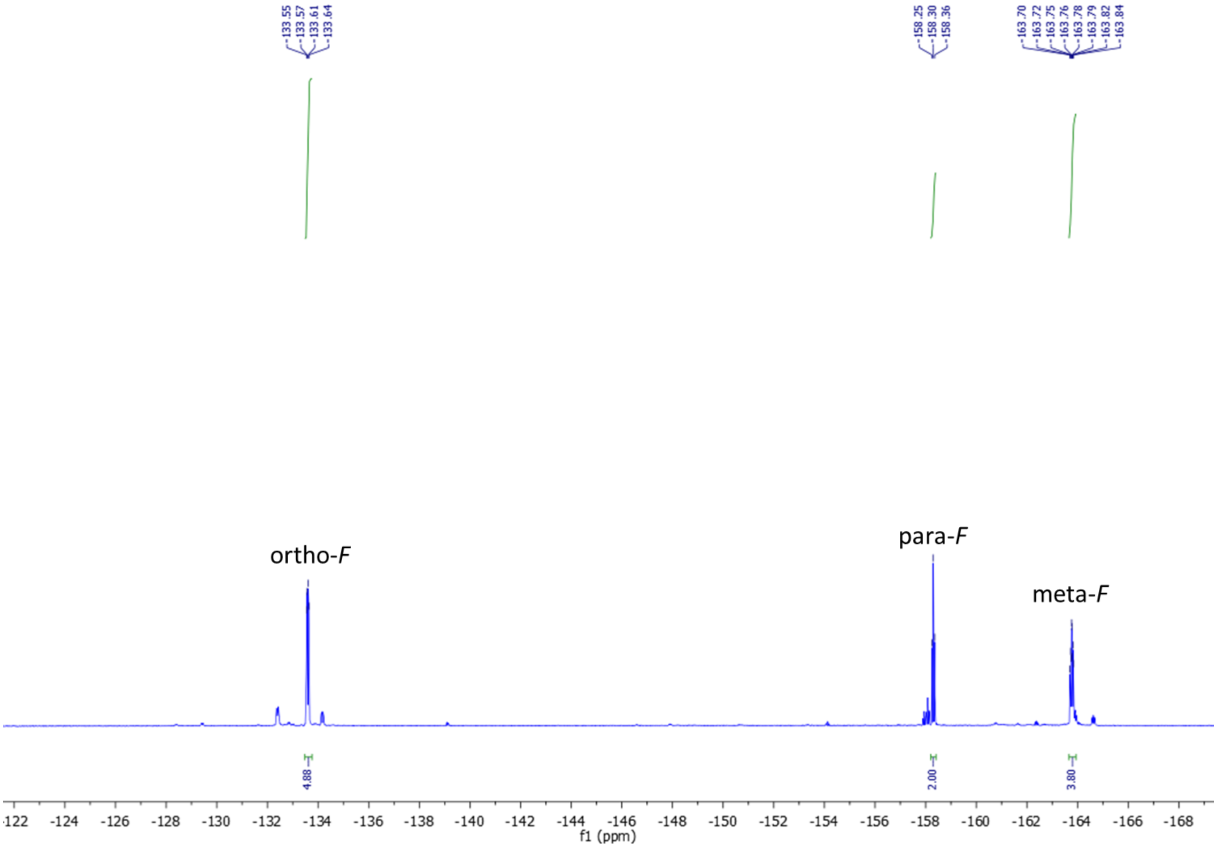

Figure SI 24: ^{19}F NMR spectrum of the cinnamylbis(perfluorophenyl)borane pyridone complex **7** (377 MHz, benzene-d_6).

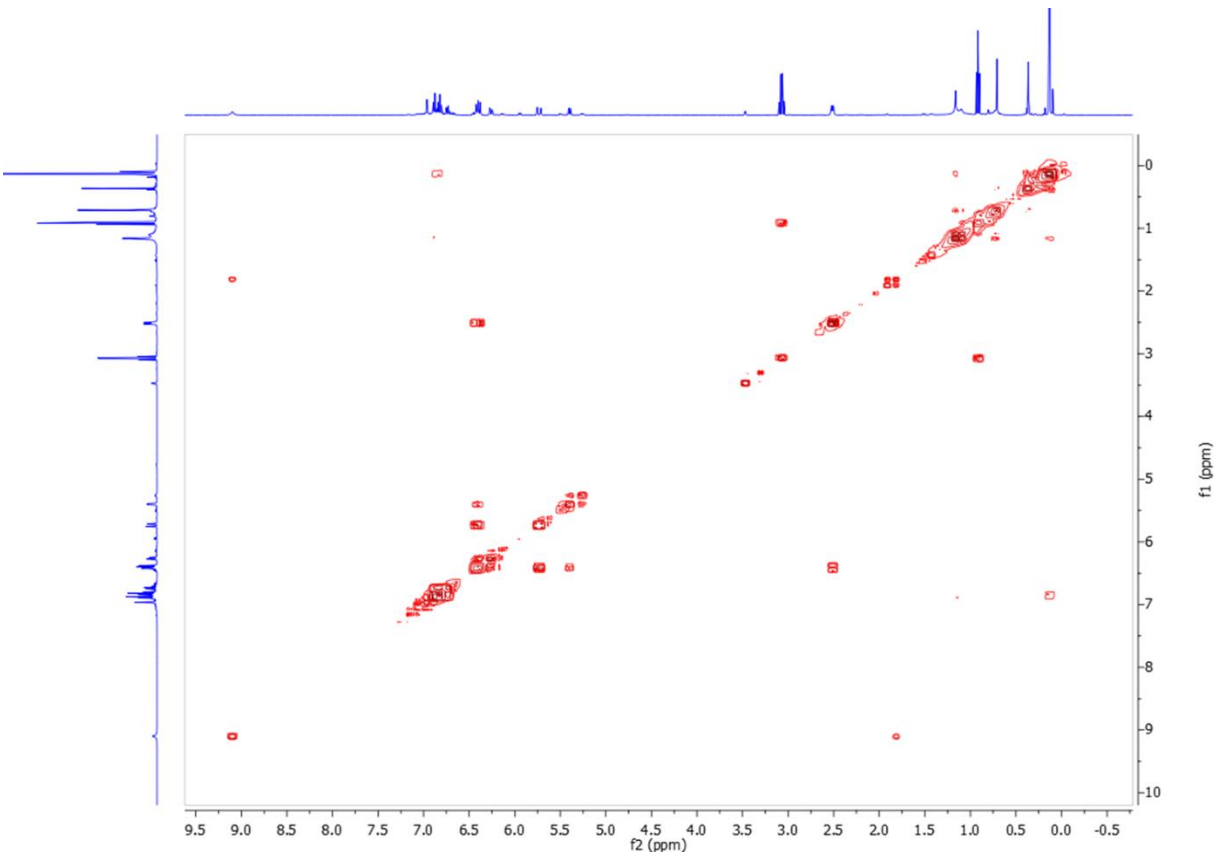

Figure SI 25: COSY NMR spectrum of the cinnamylbis(perfluorophenyl)borane pyridone complex **7** (400 MHz, benzene-d_6).

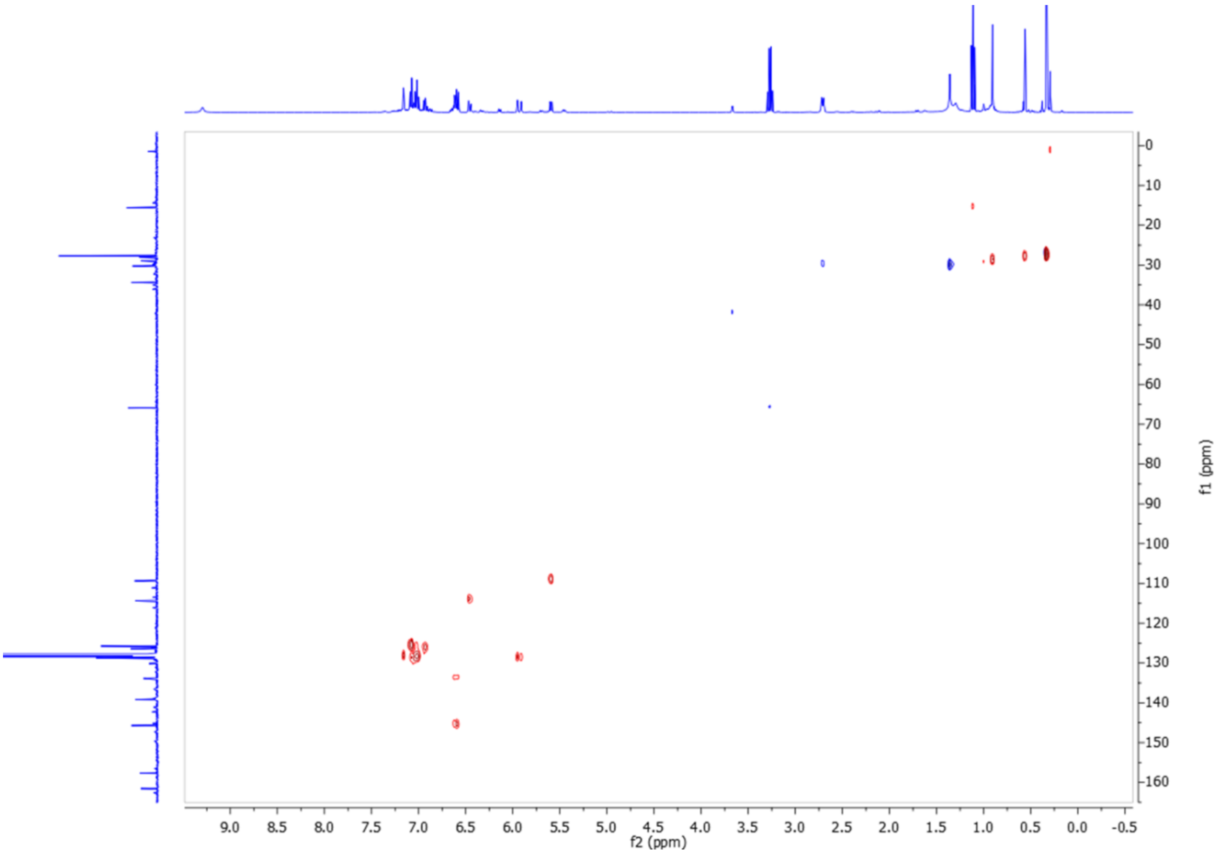

Figure SI 26: HSQC NMR spectrum of the cinnamylbis(perfluorophenyl)borane pyridone complex **7** (101 MHz, benzene-d_6).

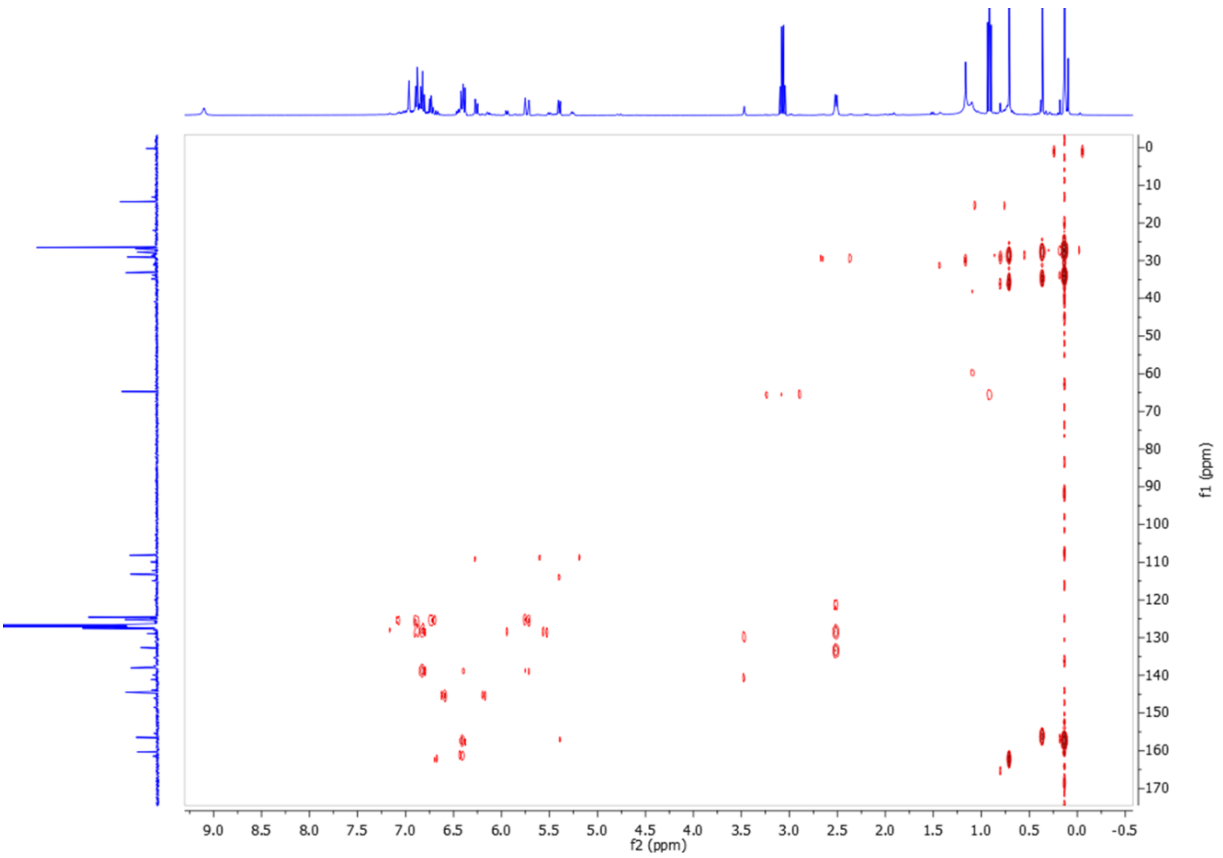

Figure SI 27: HMBC NMR spectrum of the cinnamylbis(perfluorophenyl)borane pyridone complex **7** (101 MHz, benzene-d_6).

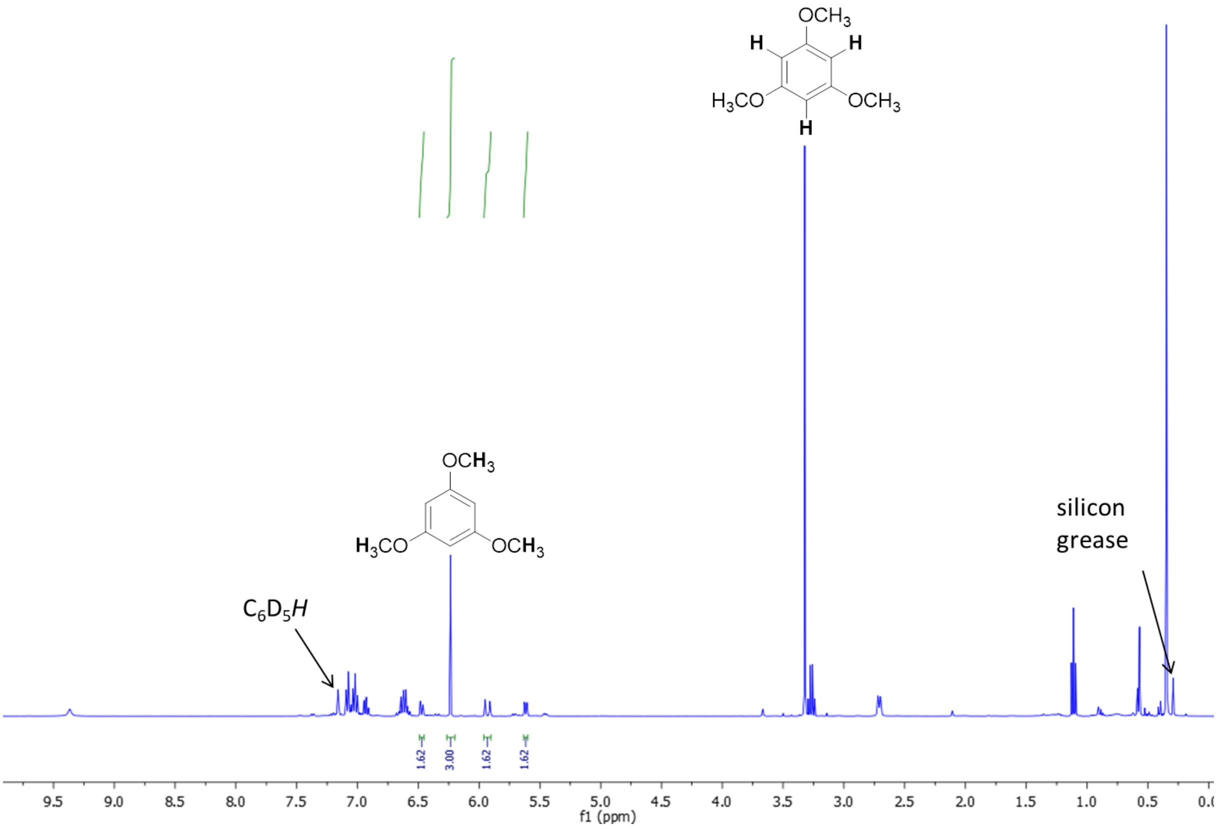

Figure SI 28: ^{1}H NMR spectrum of the cinnamylbis(perfluorophenyl)borane pyridone complex **7** with 1,3,5-trimethoxybenzene (50 µL of a 0.5 M solution in benzene-d_6) (400 MHz, benzene-d_6).

3.4 Synthesis and characterization of β-diketiminate borane complex 9

Piers borane **6** (100.0 mg, 0.29 mmol, 2.00 equiv.) and 6-*tert*-butyl-2-pyridone **5** (43.9 mg, 0.29 mmol, 2.00 equiv.) were dissolved in dry benzene (3.00 mL) in a Schlenk tube with J. Young valve, subject to three freeze-pump-thaw cycles, and heated overnight to 60 °C under passive vacuum. Phenylallene (37.0 µL, 0.29 mmol, 2.00 equiv.) and acetonitrile (15.3 µL, 0.29 mmol, 2.00 equiv.) were added. The tube was subject to three freeze-pump-thawed cycles, pressurized with H_2 (1.1 bar), and stirred overnight at room temperature. The solvent was evaporated and the crude product was purified by flash column chromatography (about 15 g silica, *n*-hexane/EtOAc, 96.5 : 3.5, $R_f \approx 0.3$). The product was obtained as yellow oil in 56% yield (44.1 mg, 0.081 mmol).

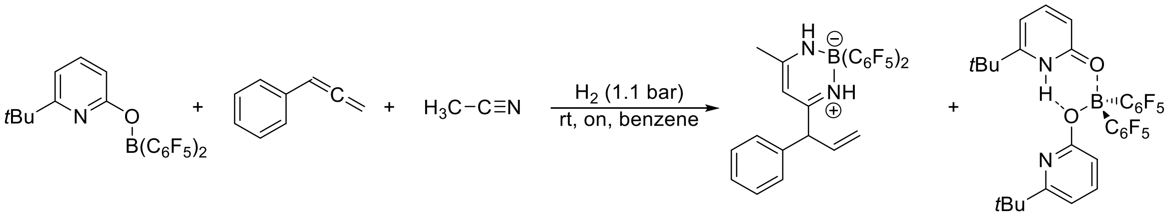

Scheme SI 2: Synthesis of ß-diketiminate borane complex **9** starting from pyridonate borane **3**.

^{1}H NMR (600 MHz, benzene-*d*$_6$) δ 7.11 – 7.07 (m, 2H, Ar-*H*), 7.04 – 7.00 (m, 1H, Ar-*H*), 6.99-6.96 (m, 2H, Ar-*H*), 6.68 (br, 1H, N*H*), 5.95 (br, 1H, N*H*), 5.82 (ddd, *J* = 17.1, 10.2, 7.6 Hz, 1H, C_{sp2}-*H*), 5.08 (d, *J* = 10.2 Hz, 1H, C_{sp2}-*H*), 4.95 (dt, *J* = 17.1, 1.1 Hz, 1H, C_{sp2}-*H*), 4.39 (t, *J* = 2.0 Hz, 1H, C_{sp2}-*H*), 3.89 (d, *J* = 7.6 Hz, 1H, bn-*H*), 1.23 (s, 3H, C*H*$_3$).

^{13}C{^{1}H} NMR (101 MHz, benzene-*d*$_6$) δ 169.2 (C_{imine}), 166.7 (C_{imine}), 138.1 (Ar-C_q), 135.5 (C_{sp2}), 128.7 (Ar-*C*), 128.2 (Ar-*C*), 127.9 (Ar-*C*), 120.2 (C_{sp2}), 90.8 (C_{sp2}), 55.9 (C_{sp3}), 22.8 (CH_3).

Remark: The ^{13}C{^{1}H} NMR spectrum shows broad signals with low intensity at 149.0, 146.6, 141.3, 138.8, 136.3 ppm which can be tentatively assigned to the pentafluorophenyl groups.

^{11}B NMR (128 MHz, benzene-*d*$_6$) δ -3.6.

^{19}F NMR (377 MHz, benzene-*d*$_6$) δ -139.36 – -139.51 (m, *o*-F), -157.79 (t, *J* = 20.7 Hz, *p*-F), -162.88 – -163.11 (m, *m*-F).

HRMS (ESI) m/z [M+H$^+$] calc. for $C_{25}H_{16}BF_{10}N_2^+$: 545.1241; found: 545.1217.

3.4.1 NMR spectra of β-diketiminate borane complex 9

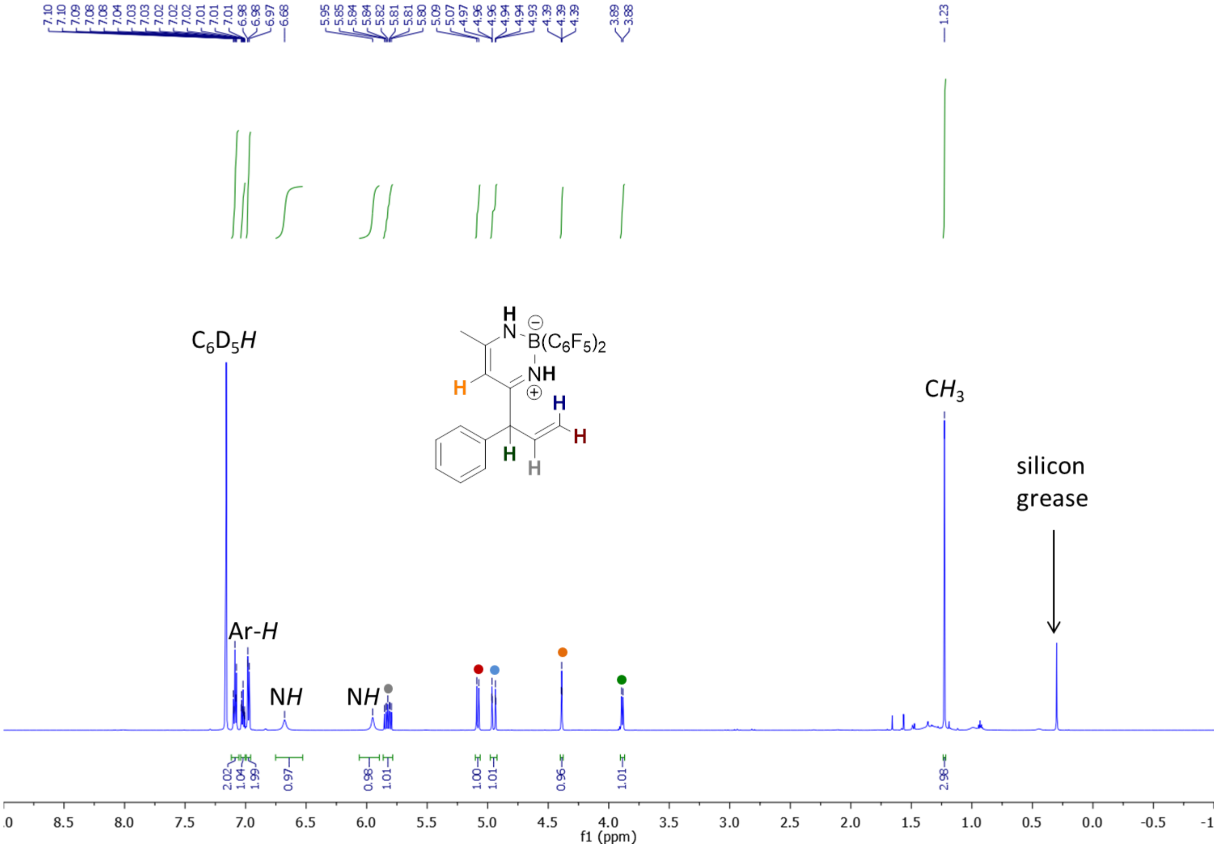

Figure SI 29: ^{1}H NMR spectrum of β-diketiminate borane complex **9** (600 MHz, benzene-d_6).

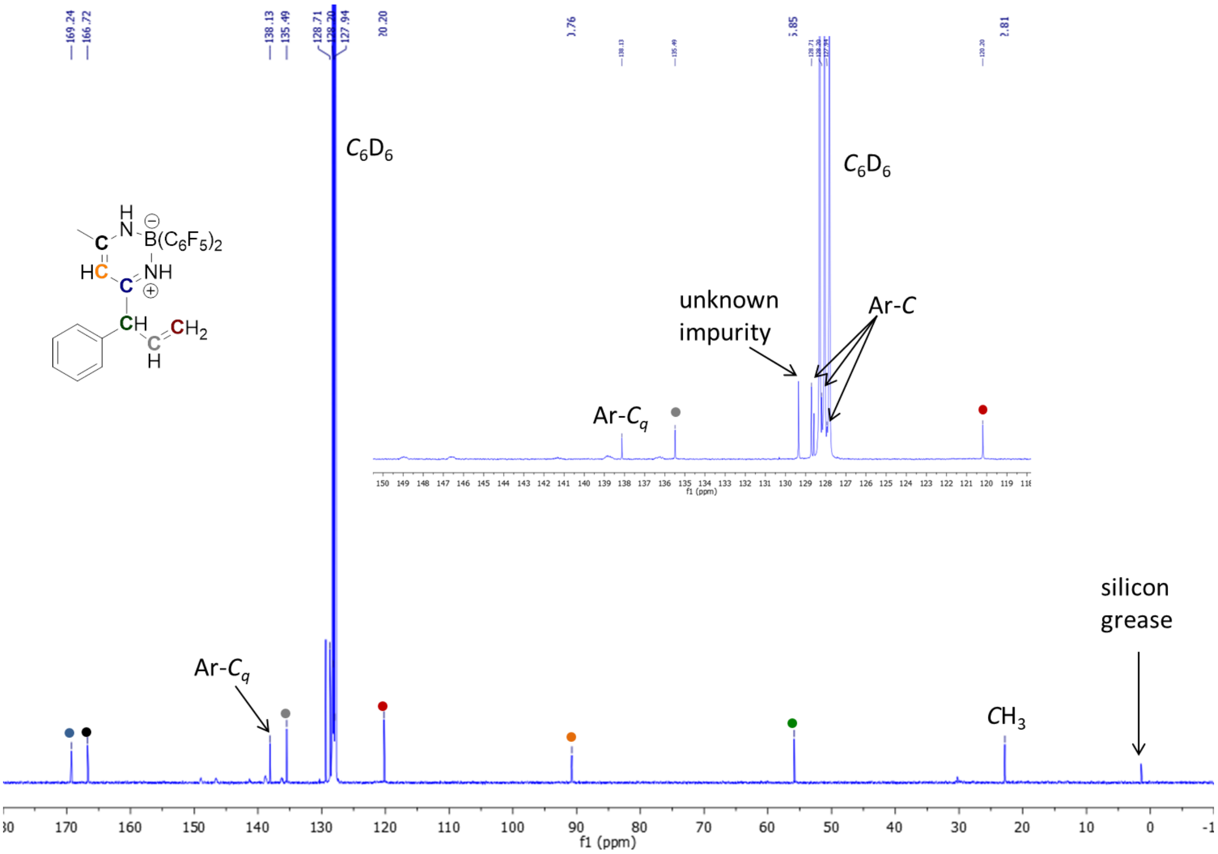

Figure SI 30: $^{13}C\{^1H\}$ NMR spectrum of β-diketiminate borane complex **9** (101 MHz, benzene-d_6).

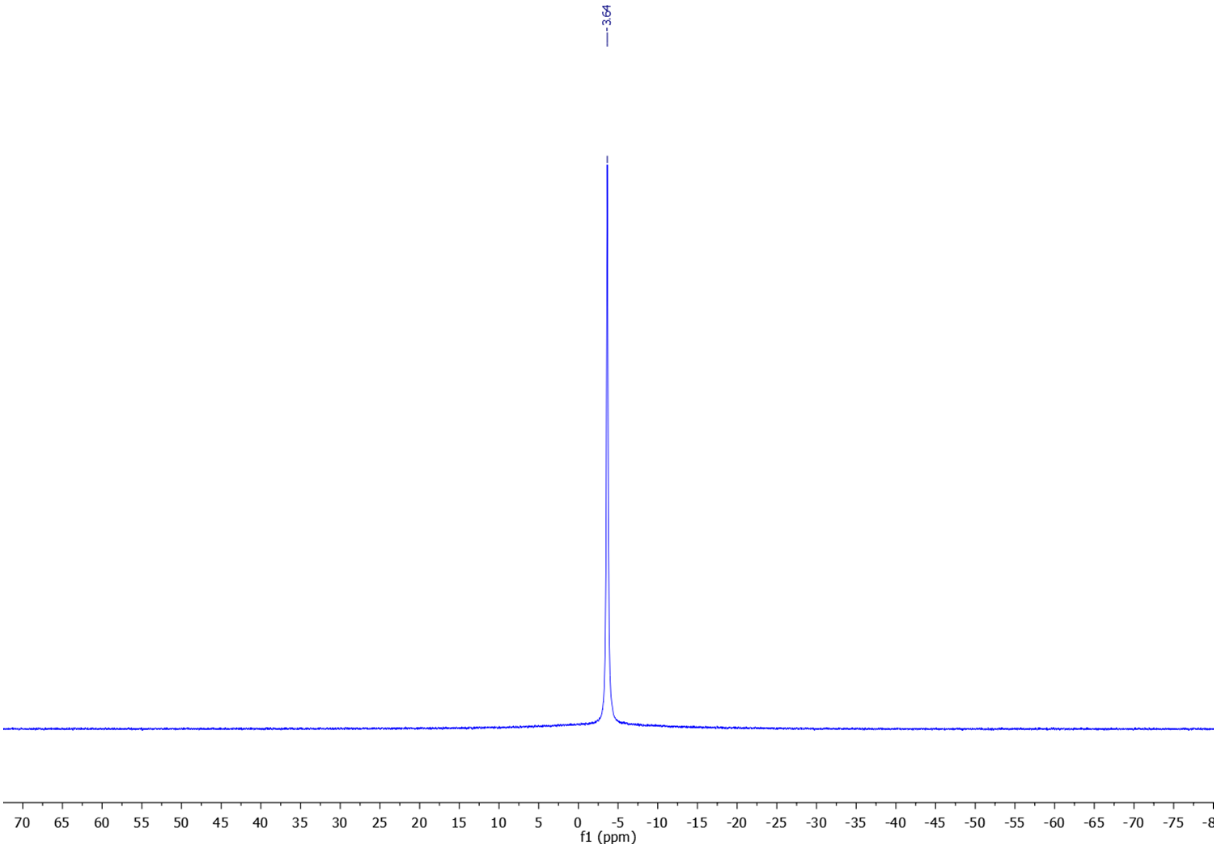

Figure SI 31: ^{11}B NMR spectrum of β-diketiminate borane complex **9** (128 MHz, benzene-d_6).

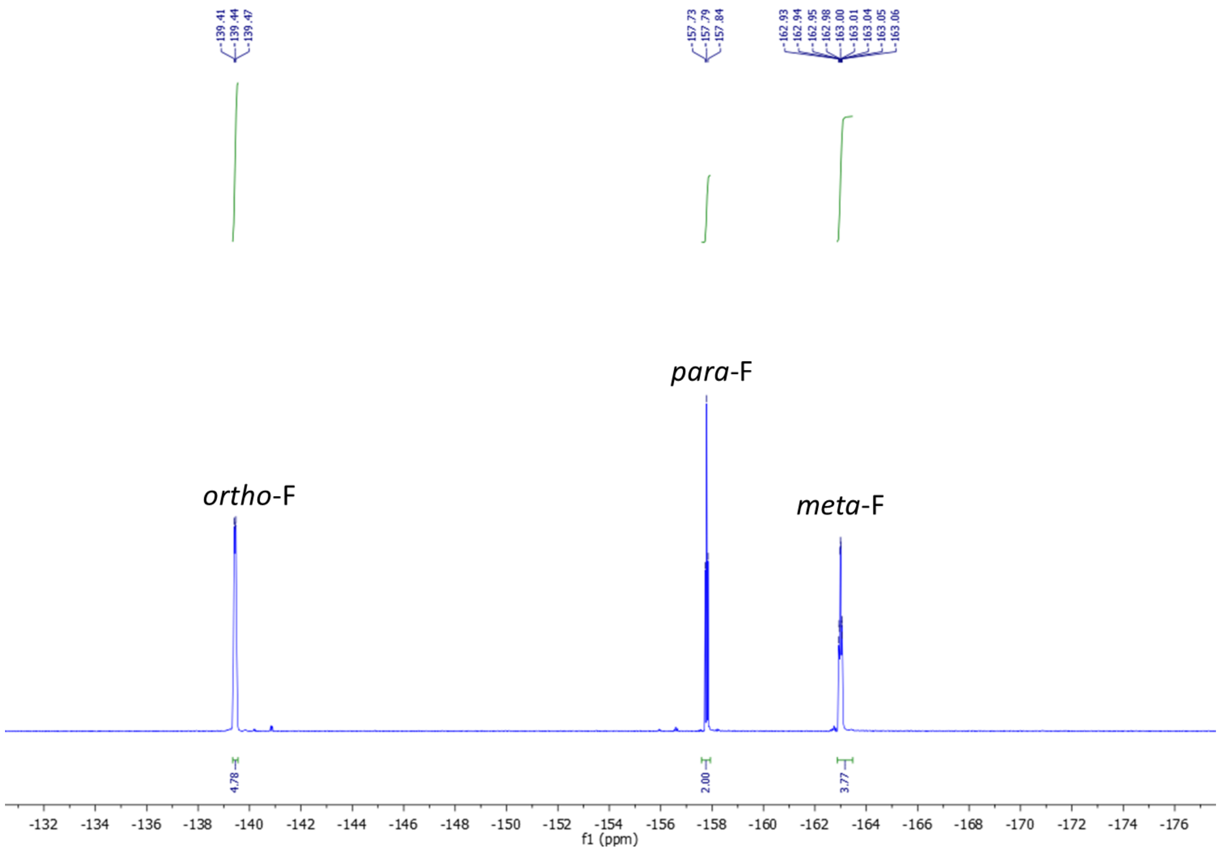

Figure SI 32: ^{19}F NMR spectrum of β-diketiminate borane complex **9** (377 MHz, benzene-d_6).

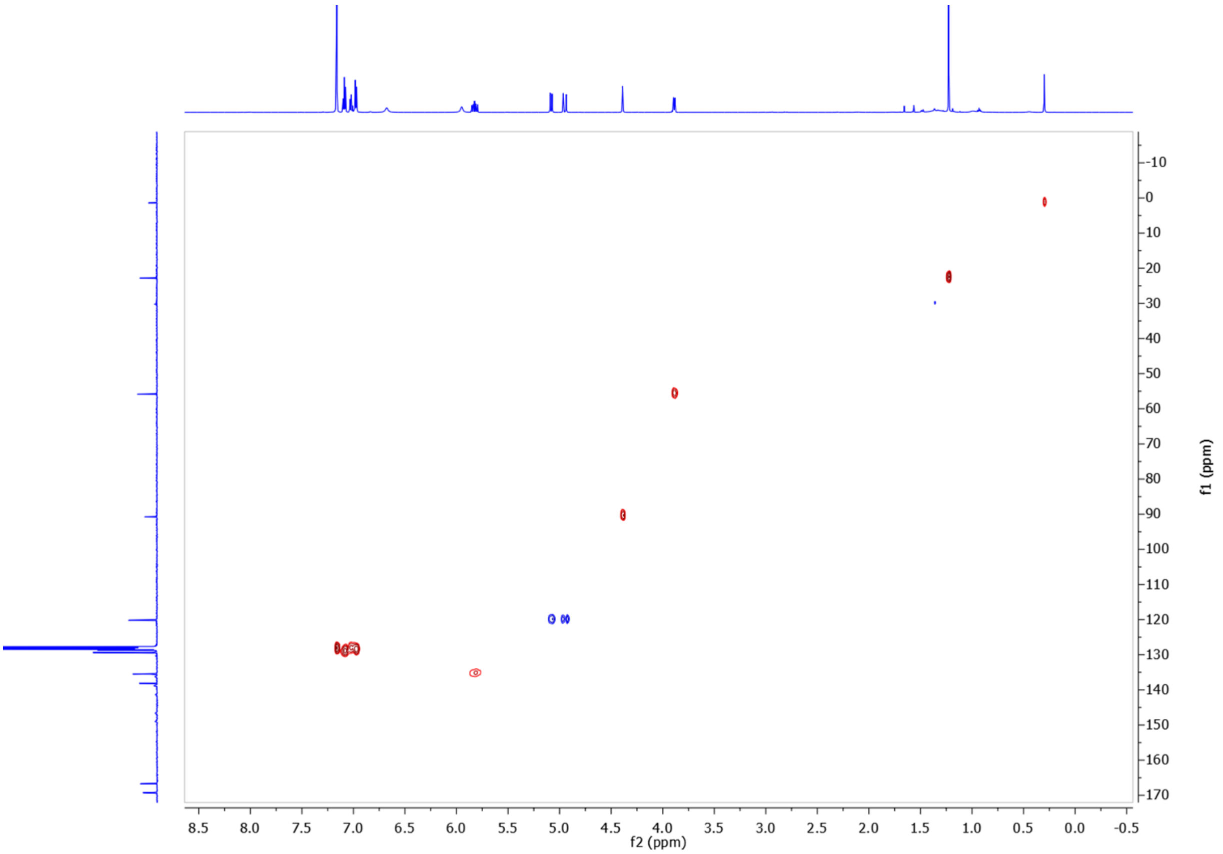

Figure SI 33: HSQC NMR spectrum with low field excerpt of β-diketiminate borane complex **9** (101 MHz, benzene-d_6).

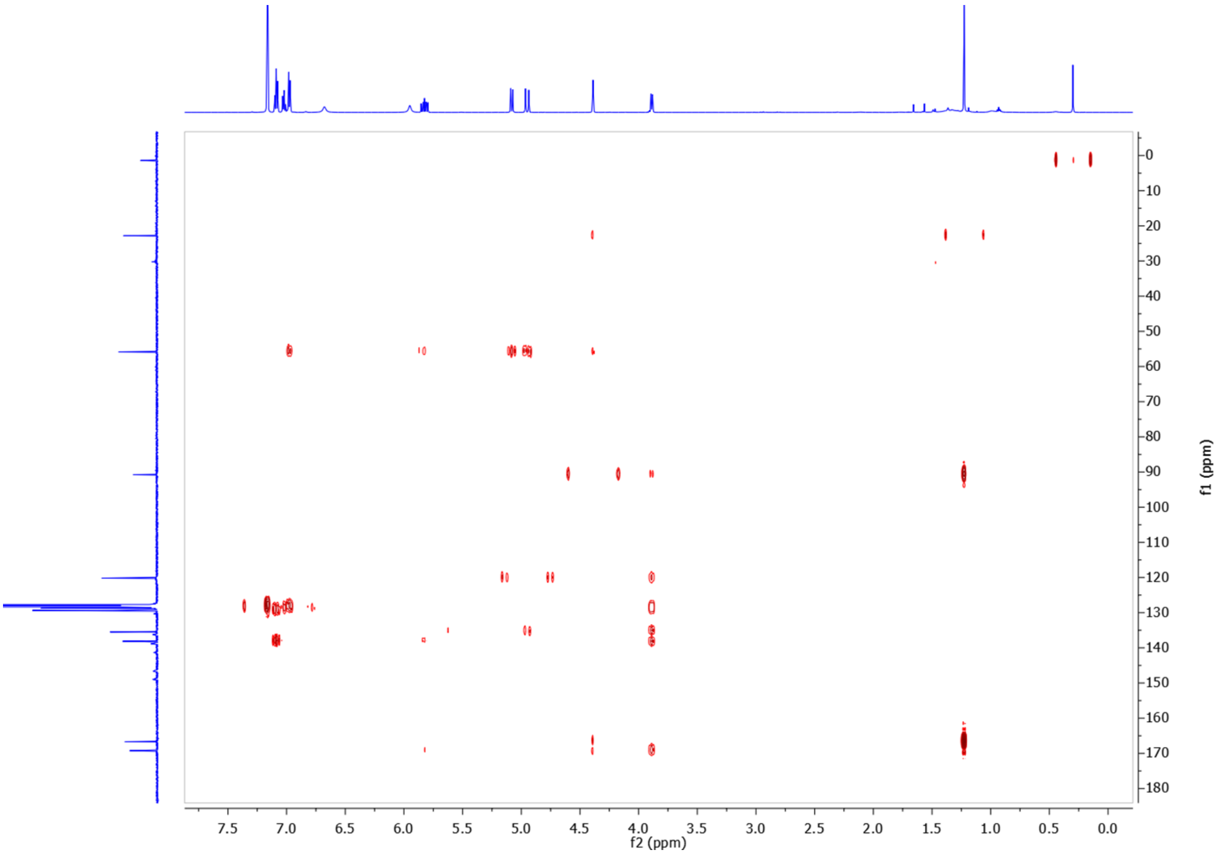

Figure SI 34: HMBC NMR spectrum of β-diketiminate borane complex **9** (101 MHz, benzene-d_6).

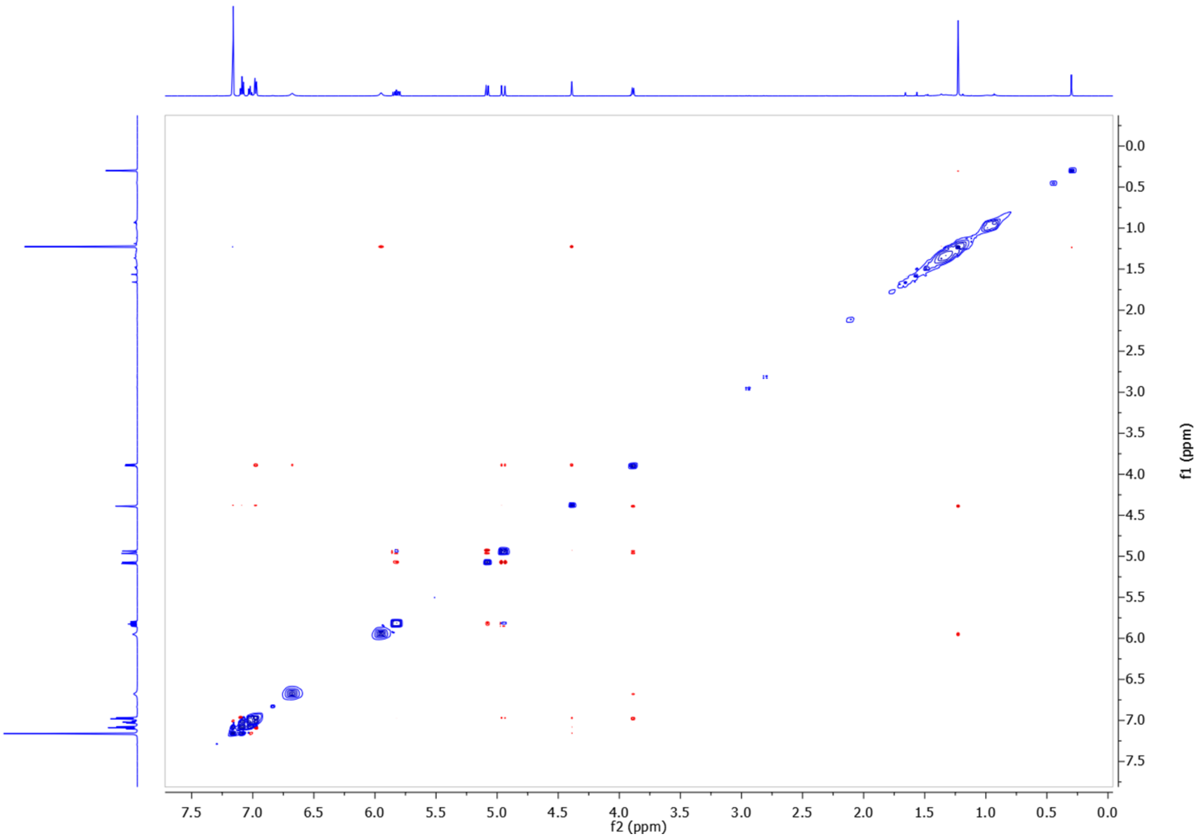

Figure SI 35: NOESY NMR spectrum of β-diketiminate borane complex **9** (600 MHz, benzene-d_6).

3.4.2 UV-Vis and fluorescence spectrum of β-diketiminate borane complex 9

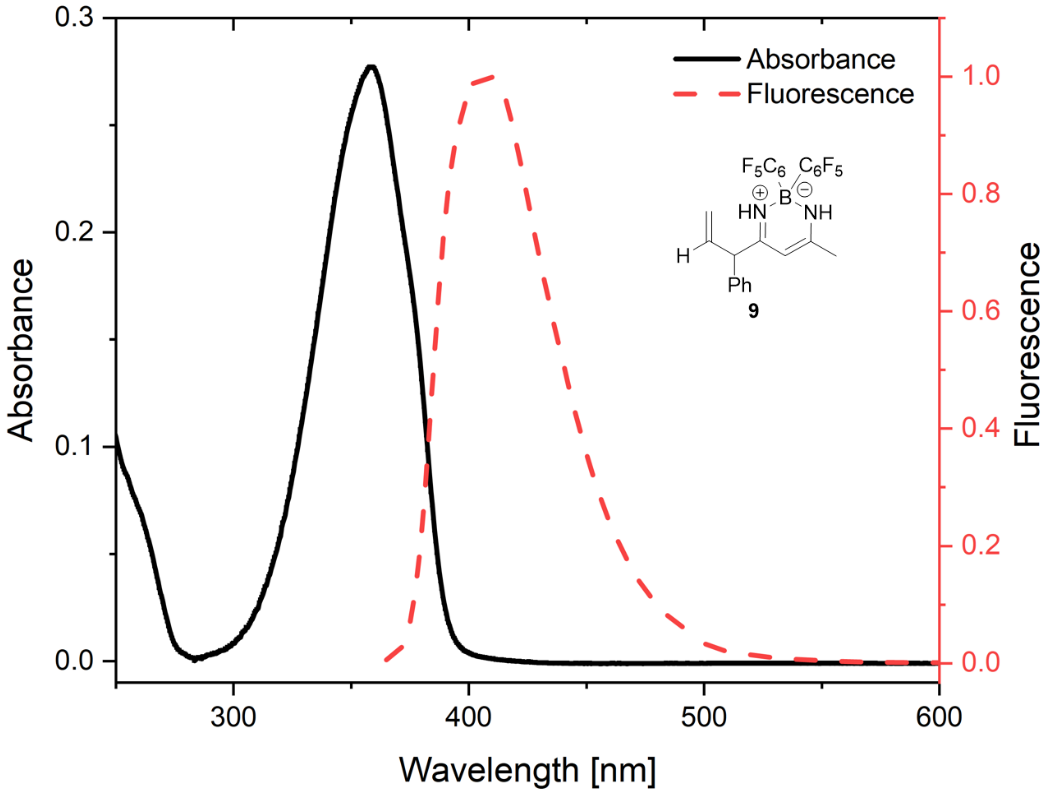

Figure SI 36: UV-Vis spectrum of **9** (black, solid line) (4×10^{-5} M in cyclohexane at 25 °C); fluorescence spectrum of **9** (red, dashed line) (1×10^{-6} M in cyclohexane at 23 °C, λ_{ex} = 359 nm).

A solution of β-diketiminate borane complex **9** in cyclohexane (4×10^{-5} M) has an absorbance maximum (λ_{max}) at 359 nm with a molar extinction coefficient (ε_{359}) of 6928 cm^{-1} M^{-1}. The fluorescence spectrum at an excitation wavelength (λ_{ex}) of 359 shows a maximum [λ_{max}(fluorescence)] at 409 nm. The Stokes shift is 50 nm.

3.5 Synthesis and characterization of ketiminoborane 11

Phenylallene (3.8 µL, 0.03 mmol, 1.00 equiv.) and acetonitrile (1.6 µL, 0.03 mmol, 1.00 equiv.) were dissolved in dry benzene-d_6 (0.40 mL). Piers borane **6** (10.4 mg, 0.03 mmol, 1.00 equiv.) was added. After 5 min at room temperature, the clear solution was transferred to an NMR tube with J. Young valve and rinsed two times with benzene-d_6 (0.10 mL).

The amount of ketimineborane **11** was quantified by an identical experiment in which 1,3,5-trimethoxybenzene was added (50 µL of a 0.5 M solution in benzene-d_6) (Figure SI 45). The yield by qNMR of ketimineborane **11** was 83%.

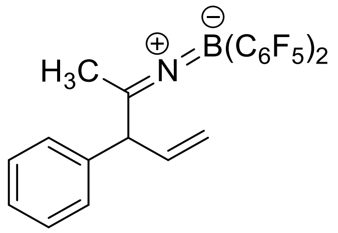

^{1}H NMR (400 MHz, benzene-d_6) δ 7.06 – 7.01 (m, 2H, Ar-*H*), 6.99 – 6.92 (m, 3H, Ar-*H*), 6.02 (ddd, *J* = 17.1, 10.2, 7.8 Hz, 1H, *H*C=CH$_2$), 5.04 (dt, *J* = 10.3, 1.1 Hz, 1H, HC=C*H*$_2$), 4.87 (dt, *J* = 17.0, 1.3 Hz, 1H, HC=C*H*$_2$), 3.86 (d, *J* = 7.9 Hz, 1H, bn-*H*), 1.71 (s, 1H,C*H*$_3$).

^{13}C{^{1}H} NMR (101 MHz, benzene-d_6) δ 161.3 (*C*=N), 138.4 (Ar-*C*$_q$), 135.5 (*C*$_{sp2}$=CH$_2$), 129.1 (Ar-*C*$_{sp2}$), 128.5 (Ar-*C*$_{sp2}$), 128.0 (Ar-*C*$_{sp2}$), 118.6 (C=*C*$_{sp2}$H$_2$), 59.7 (bn-*C*), 25.2 (*C*H$_3$)

Remark: The ^{13}C{^{1}H} NMR spectrum shows broad signals with low intensity at 149.6, 147.1, 138.9, 136.4 ppm which can be tentatively assigned to the pentafluorophenyl groups.

^{11}B NMR (128 MHz, benzene-d_6) δ 21.4.

^{19}F NMR (377 MHz, benzene-d_6) δ -132.43 (ddd, *J* = 60.9, 23.8, 9.6 Hz, *o*-F), -151.83 – -152.26 (m, *p*-F), -162.06 – -162.31 (m, *m*-F).

IR (ATR): 1867 (ν C=N) cm^{-1}

Although we were not able to identify the molecule ion *via* ESI mass spectrometry, we identified a potential fragment ion which we assigned to the structure below.

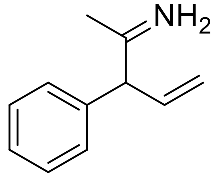

HRMS (ESI) m/z [M+H$^+$] calc. for $C_{11}H_{14}N^+$: 160.112; found: 160.111.

3.5.1 IR spectrum of ketiminoborane 11

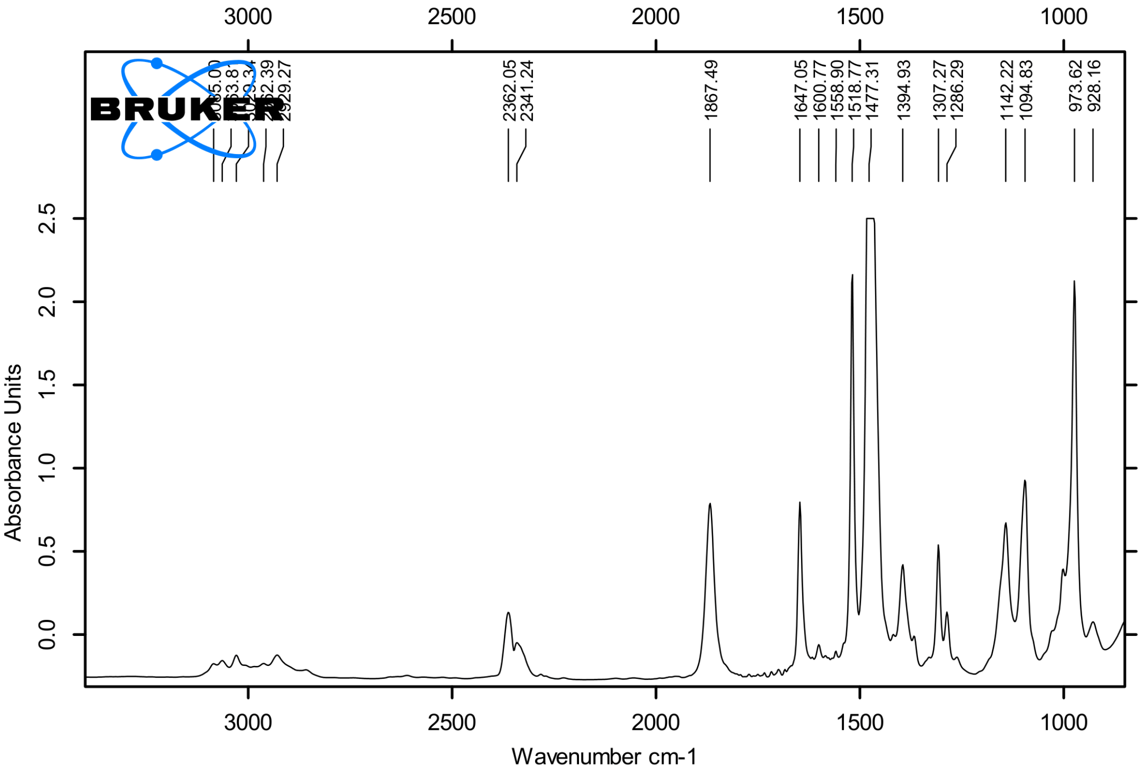

Figure SI 37: IR-spectrum of ketiminoborane **11.**

3.5.2 NMR spectra of ketiminoborane 11

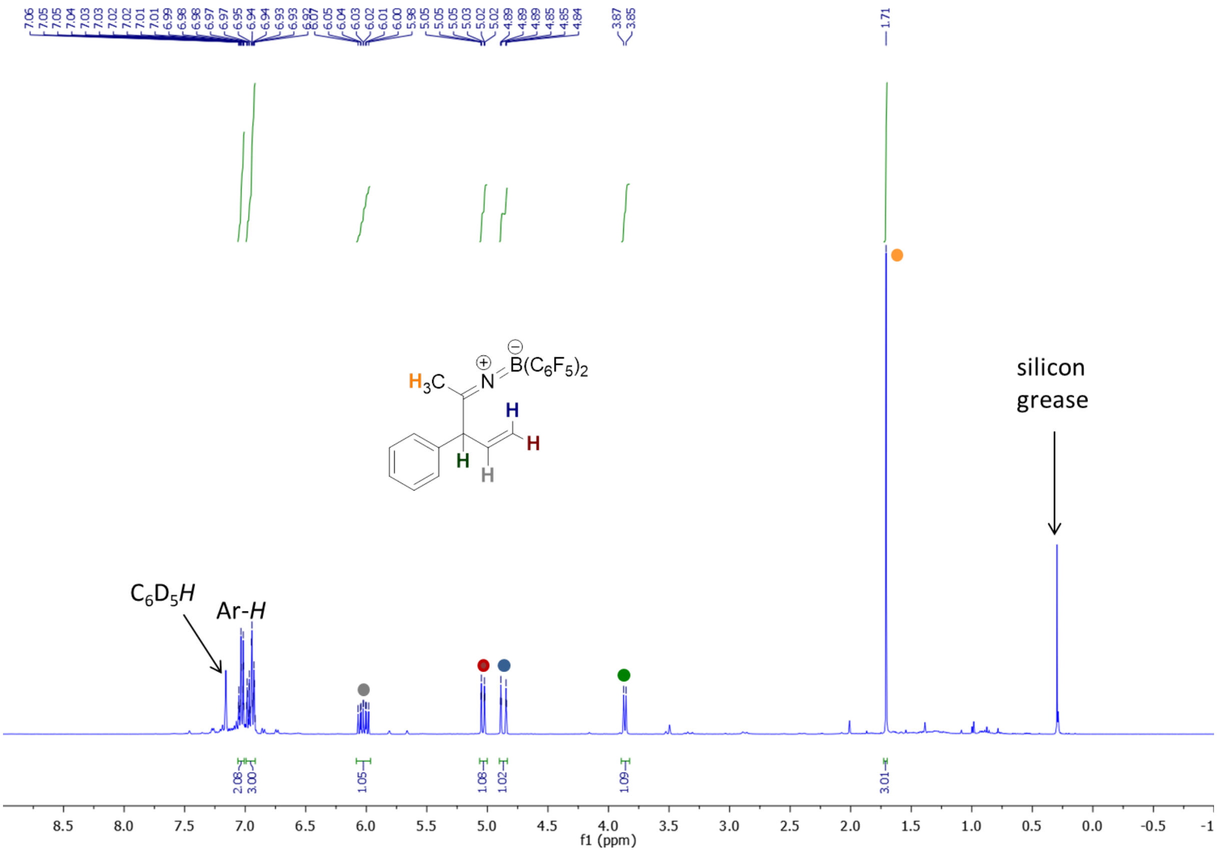

Figure SI 38: ^{1}H NMR spectrum of ketiminoborane **11** (400 MHz, benzene-d_6).

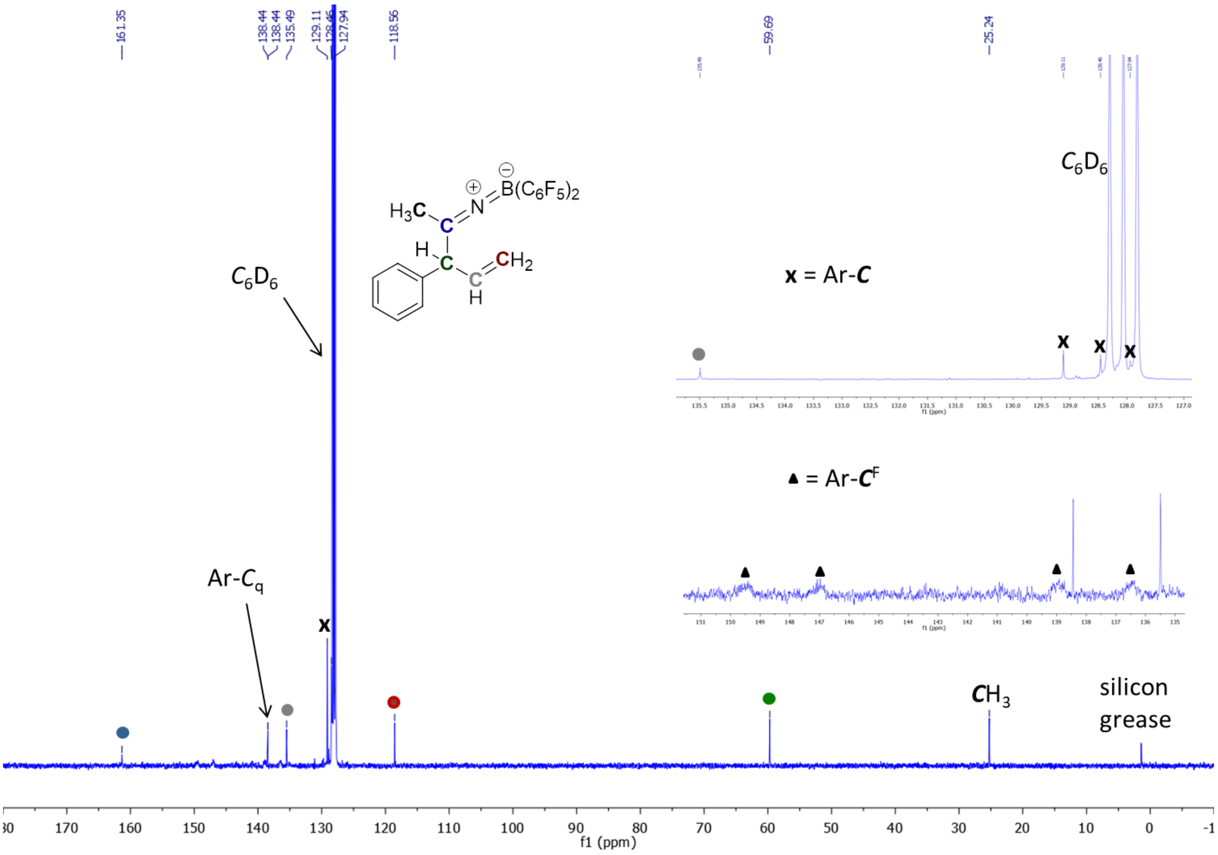

Figure SI 39: $^{13}C\{^1H\}$ NMR spectrum of ketiminoborane **11** (101 MHz, benzene-d_6).

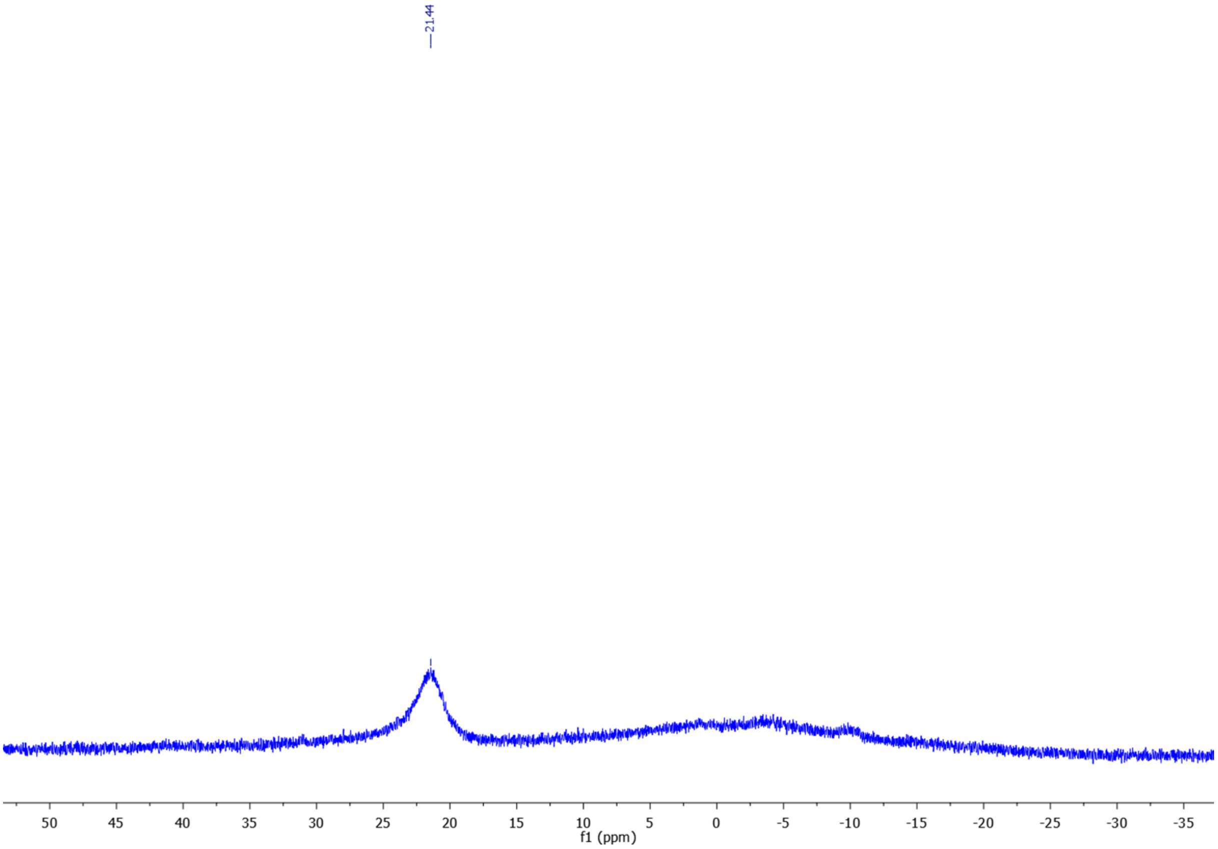

Figure SI 40: ^{11}B NMR spectrum of ketiminoborane **11** (128 MHz, benzene-d_6).

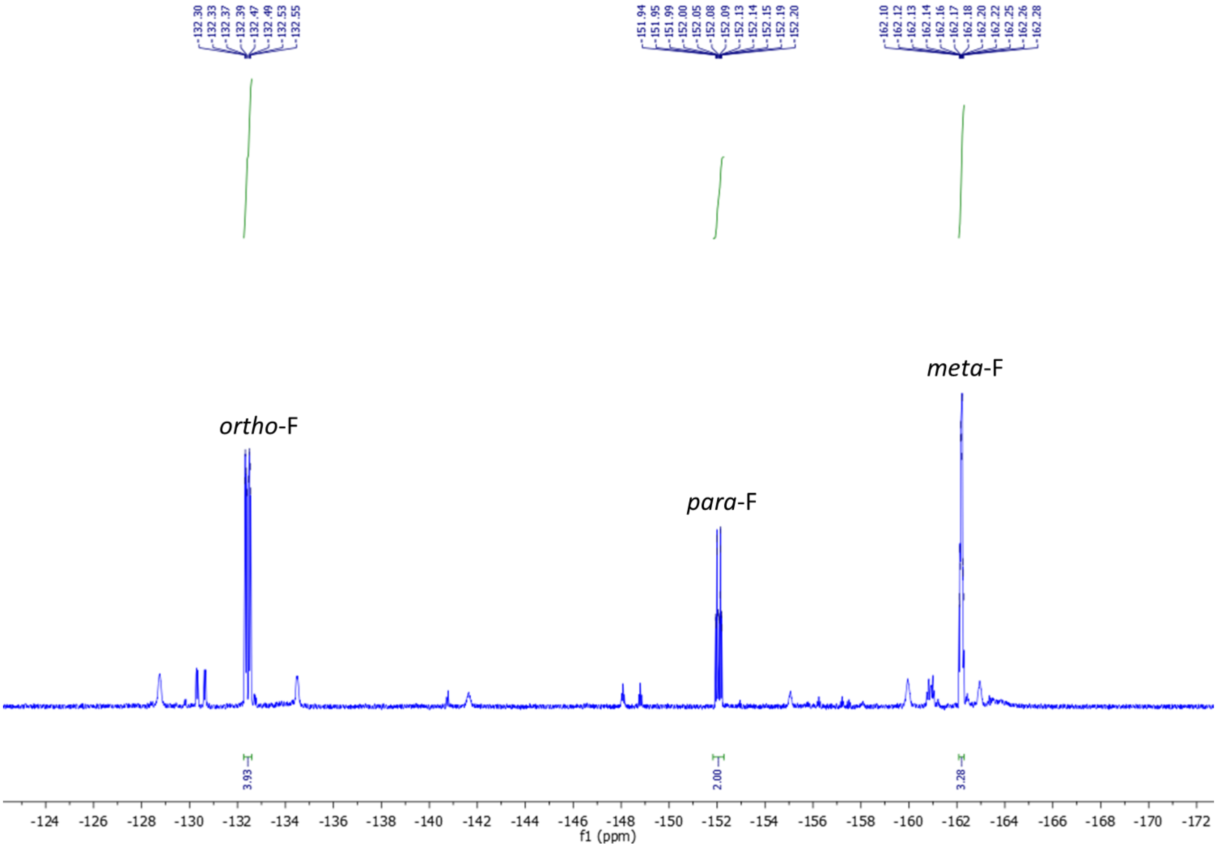

Figure SI 41: ^{19}F NMR spectrum of ketiminoborane **11** (377 MHz, benzene-d_6).

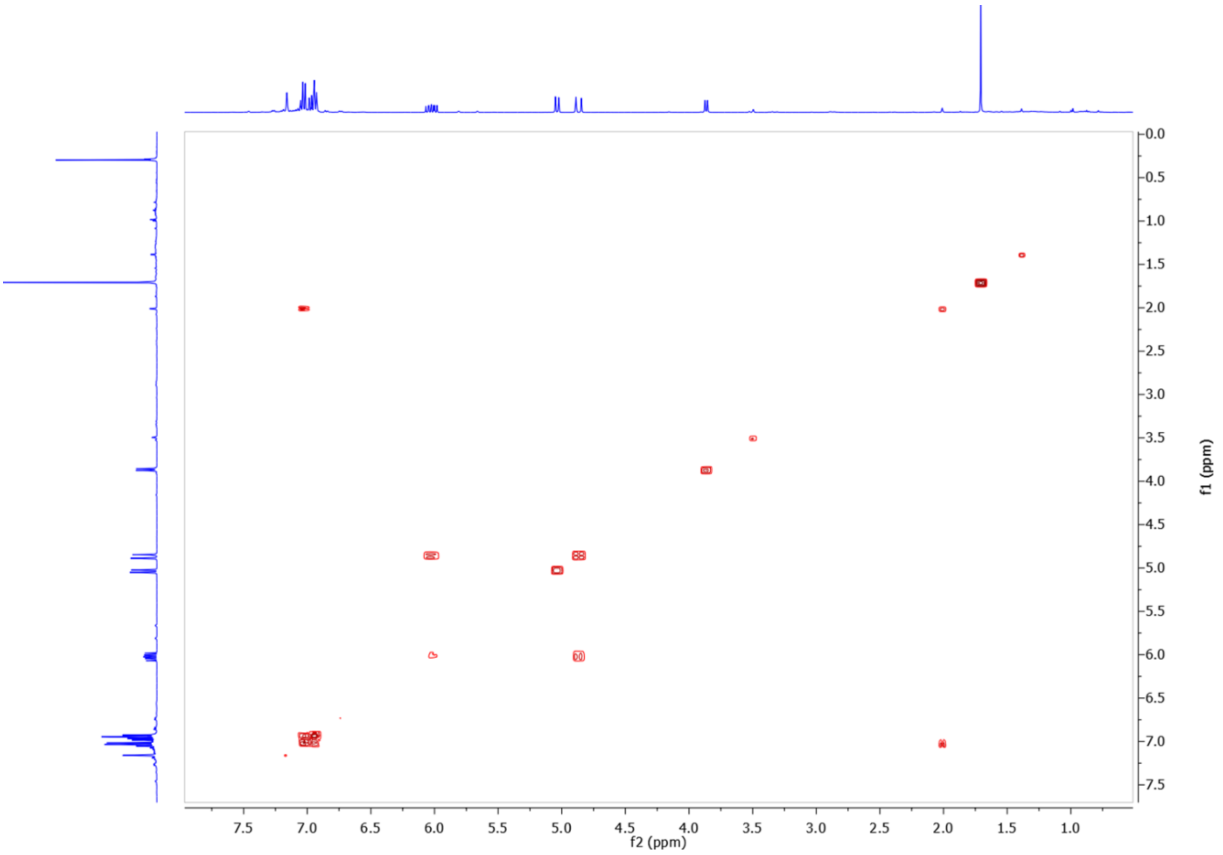

Figure SI 42: COSY NMR spectrum of ketiminoborane **11** (400 MHz, benzene-d_6).

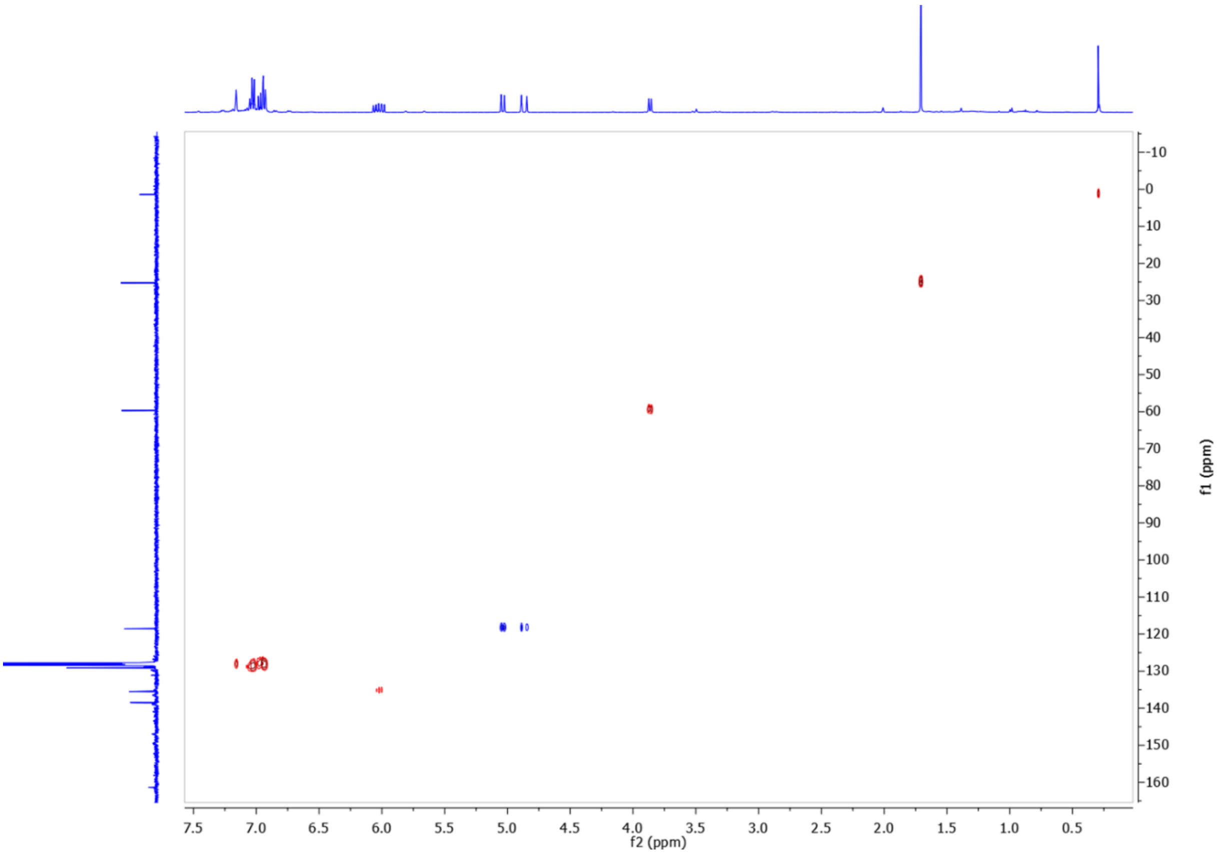

Figure SI 43: HSQC NMR spectrum of ketiminoborane **11** (128 MHz, benzene-d_6).

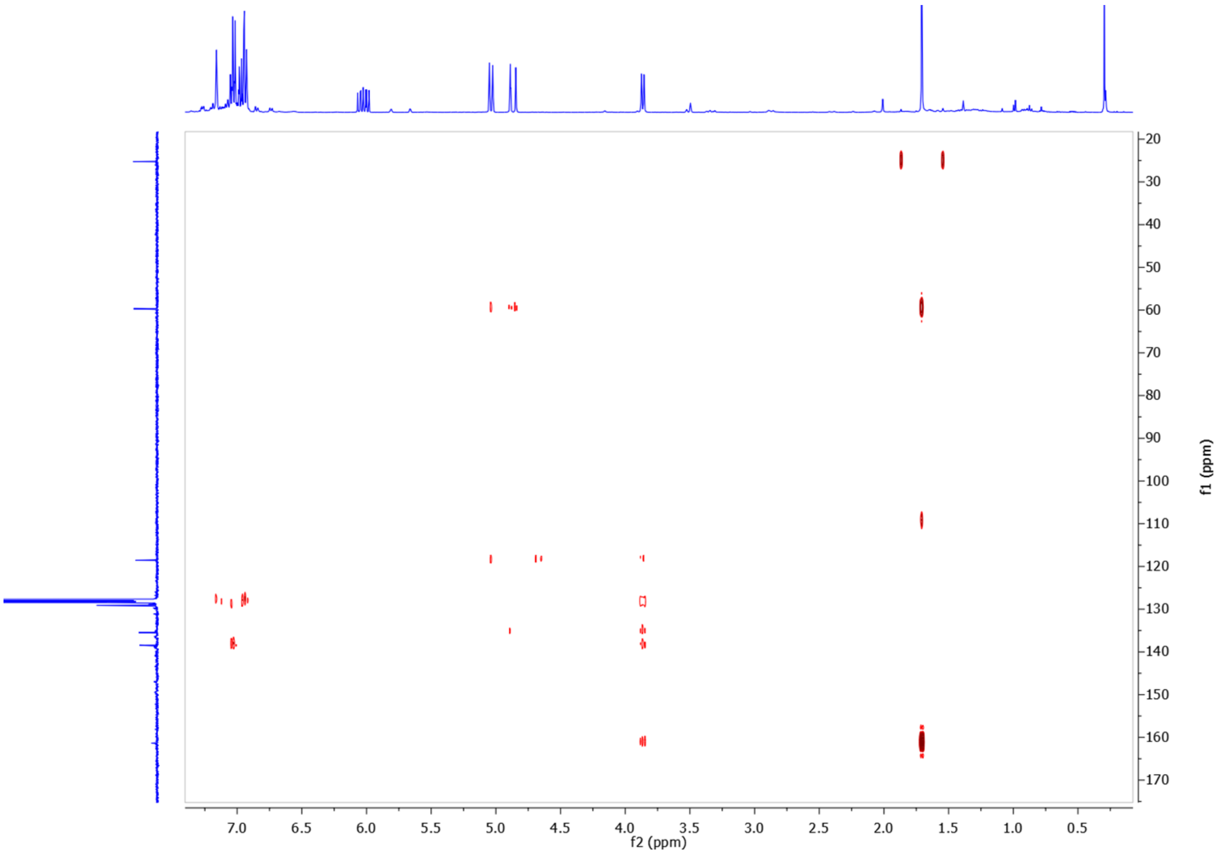

Figure SI 44: HMBC NMR spectrum of ketiminoborane **11** (128 MHz, benzene-d_6).

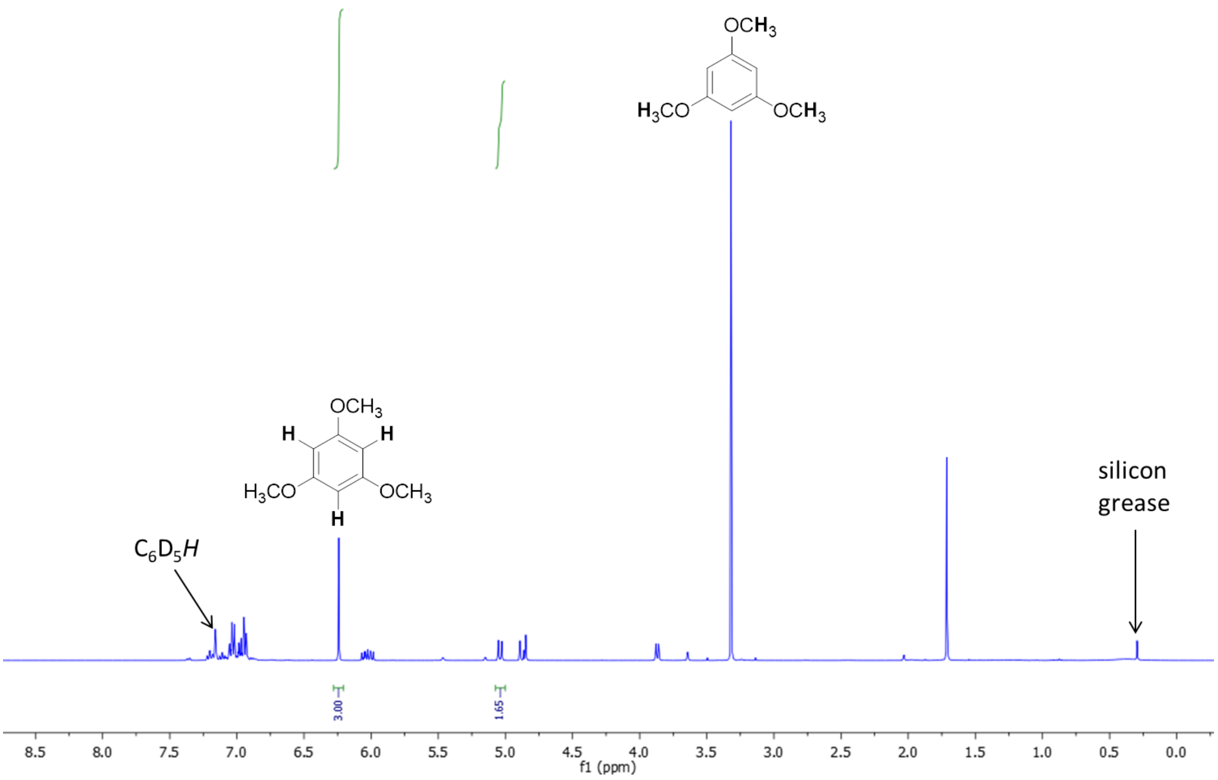

Figure SI 45: ^{1}H NMR spectrum of ketiminoborane **11** with 1,3,5-trimethoxybenzene (50 µL of a 0.5 M stock solution in benzene-d_6, 0.025 mmol) (400 MHz, benzene-d_6).

3.6 Synthesis and characterization of ketiminoborane pyridone complex 12

The reaction was set up in a nitrogen filled glovebox. Piers Borane **6** (34.6 mg, 100 µmol, 1.00 equiv.) was suspended in toluene-d_8 (2.0 mL). Then, acetonitrile (5.22 µL, 100 µmol, 1.00 equiv.) and phenylallene (12.8 µL, 100 µmol, 1.00 equiv.) were added. The suspension was shaken for 30 min, until it turned into a clear solution. It was kept at room temperature overnight. Afterwards it was cooled to –35 °C in a freezer. Then a –35 °C cold solution of 6-*tert*-butyl-2-pyridone **5** in toluene-d_8 (100.1 mg in 1.001 mL) (121 µL, 80.0 µmol, 0.80 equiv.) was added at –35 °C (the amount of pyridone **5**, was kept lower compared to ketamine borane **11**, to supress the formation of biscomplex **10**). An aliquot was transferred into a precooled NMR tube with J. Young valve. The NMR tube was frozen in liquid N_2 and transferred to an NMR spectrometer (600 MHz, sample holder cooled to –10 °C). Spectra were recorded at –10 °C. The main impurity is remaining phenylallene (marked with a black dot in the spectra).

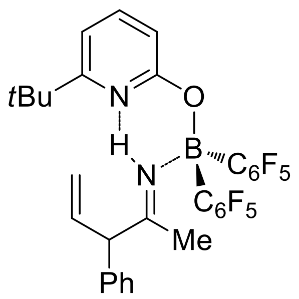

^{1}H NMR (600 MHz, toluene-d_8) δ 10.90 (s, 1H, N-*H*), 7.08 (dd, *J* = 8.2, 7.4 Hz, 1H, Py-*H*), 6.94 – 6.87 (m, 3H, *o*-Ar-*H*, *p*-Ar-*H*), 6.71 (d, *J* = 8.2 Hz, 1H, Py-*H*), 6.52 – 6.46 (m, 3H, *m*-Ar-*H*, Py-*H*), 5.60 (ddd, *J* = 17.2, 10.3, 6.8 Hz, 1H, *H*C=CH$_2$), 4.85 (d, *J* = 6.8 Hz, 1H, HC=C*H$_2$*), 4.71 (d, *J* = 17.2 Hz, 1H, HC=C*H$_2$*), 3.32 (d, *J* = 6.8 Hz, 1H, Ph-C*H*), 1.61 (s, 1H, C*H$_3$*), 1.05 (s, 3H, C(C*H$_3$*)$_3$).

^{13}C{^{1}H} NMR (151 MHz, toluene-d_8) δ 189.6 (*C*=NH), 166.4 (C_{Py}-C(CH$_3$)$_3$), 162.8 (C_{Py}-O), 139.3 (*p*-C_{Py}), 135.1 (Ph-C_q), 132.2 (H*C*=CH$_2$), 129.2 (*m*-C_{Ph}), 128.8 (*p*-C_{Ph}), 128.2 (*o*-C_{Ph}), 124.2 (HC=*C*H$_2$), 110.8 (*m*-C_{Py}), 59.5 (Ph-*C*H), 37.0 (*C*(CH$_3$)$_3$), 29.9 (C(*C*H$_3$)$_3$), 21.0 (*C*H$_3$).

^{11}B NMR (193 MHz, toluene-d_8) δ –0.36.

^{15}N NMR (61 MHz, toluene-d_8) δ 227.3 ($^1J_{NH}$ = 52.8 Hz).

Remark: The ^{19}F NMR spectrum (Figure SI 56) was measured from a different sample synthesized by a similar experiment where one equivalent of pyridone **5** (4.5 mg, 0.03 mmol) was added to a solution of ketiminoborane **11** (15.1 mg, 0.03 mmol) in benzene-d_6 (0.6 mL) at room temperature. Although ^{1}H, ^{13}C, ^{11}B and 2D NMR spectra show the formation of ketiminoborane pyridone complex **12** as main species, the corresponding ^{19}F NMR spectrum shows a complex reaction mixture where the assignment of the signals is not unambiguously possible.

3.6.1 NMR spectra of ketiminoborane pyridone complex 12

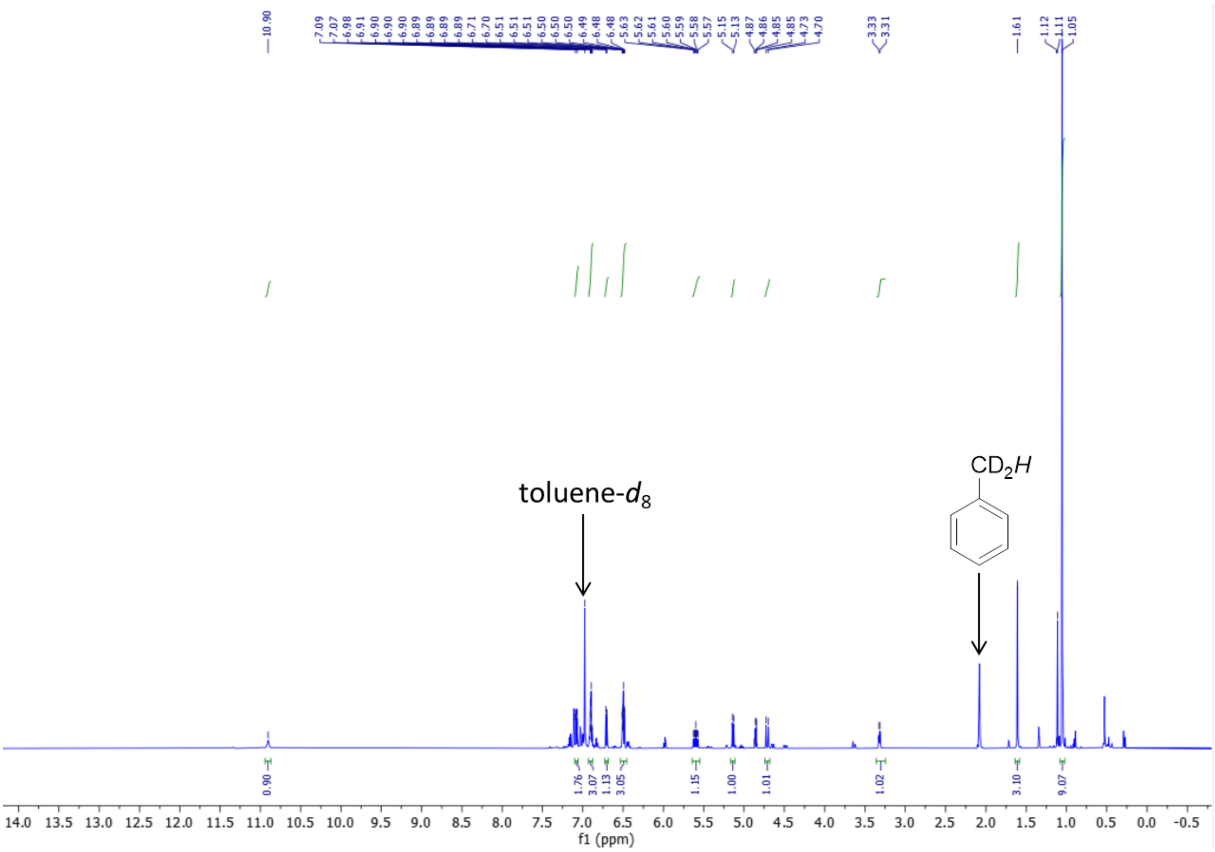

Figure SI 46: ^{1}H NMR spectrum of ketiminoborane pyridone complex **12** (600 MHz, toluene-d_8).

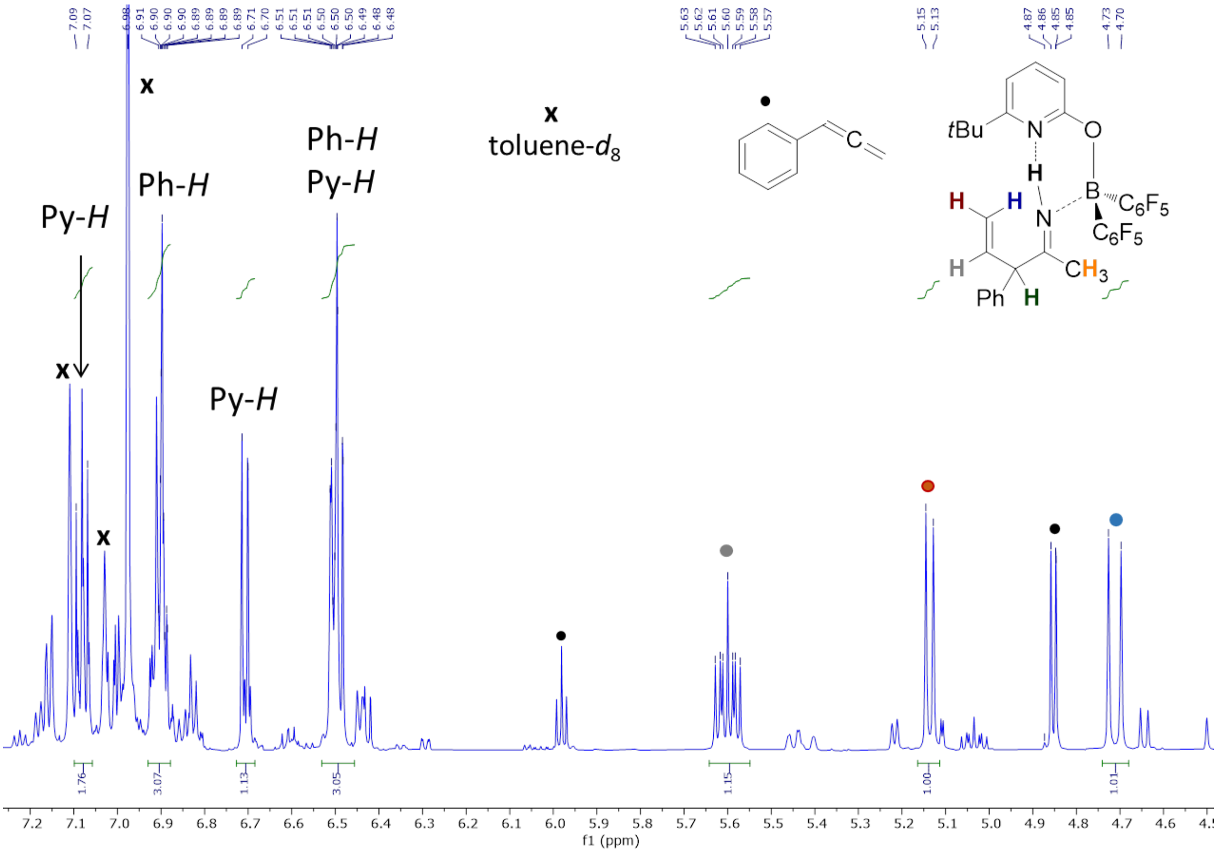

Figure SI 47: Low field excerpt of the ^{1}H NMR spectrum of ketiminoborane pyridone complex **12** (600 MHz, toluene-d_8).

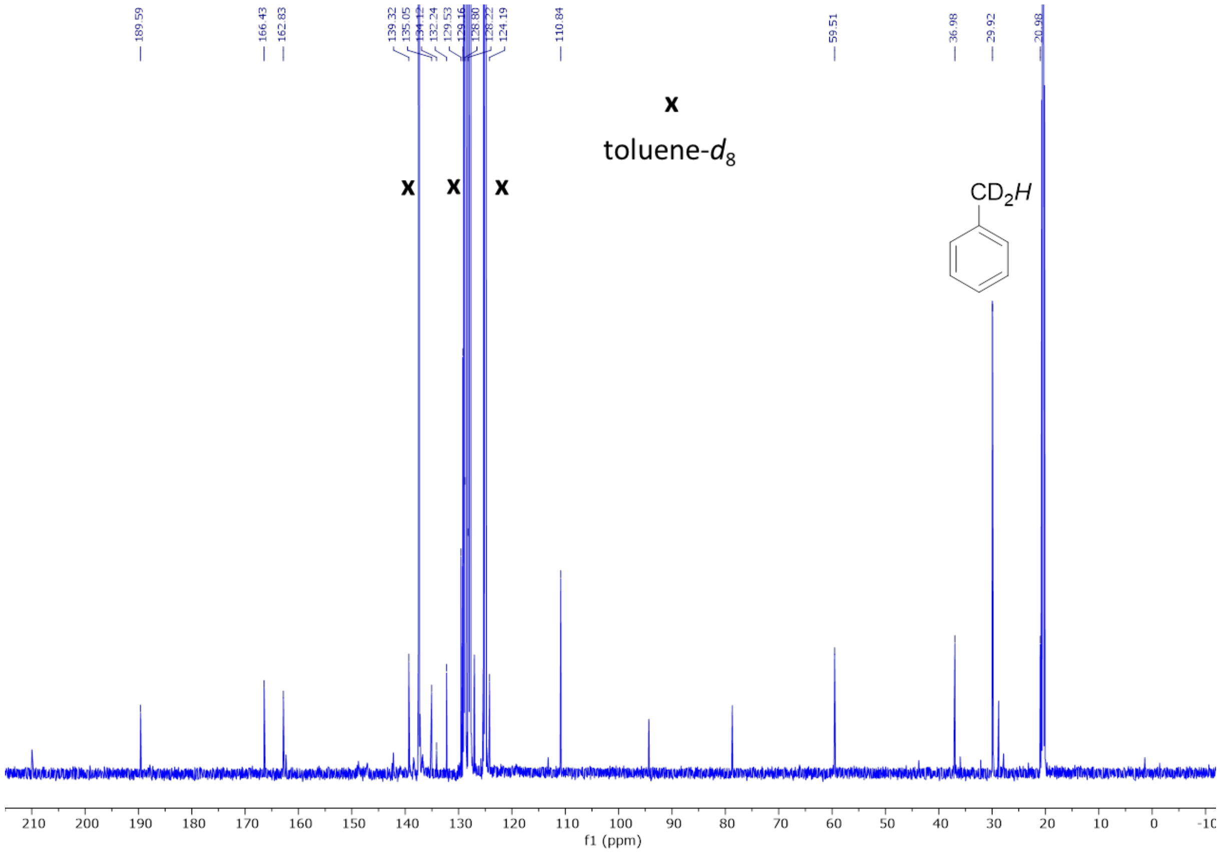

Figure SI 48: ^{13}C NMR spectrum of ketiminoborane pyridone complex **12** (151 MHz, toluene-d_8).

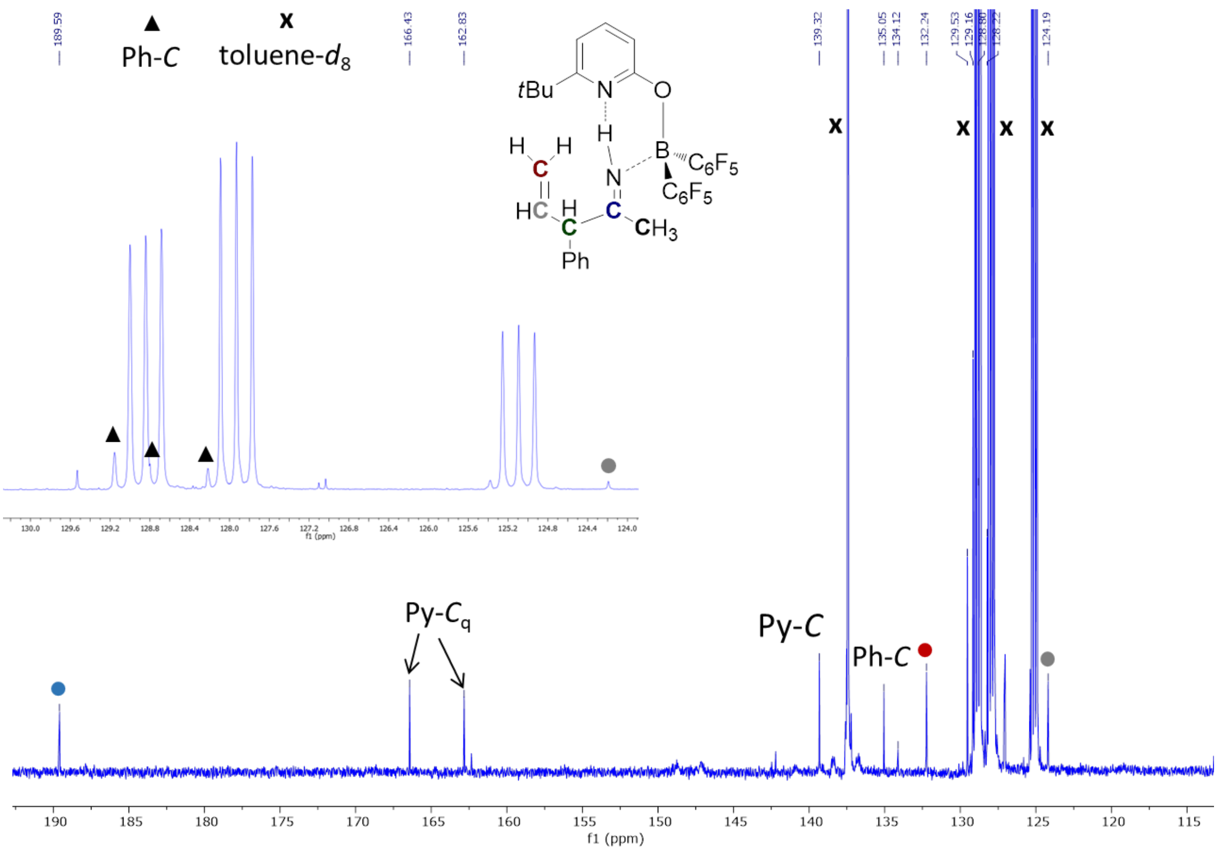

Figure SI 49: Low field shift of the ^{13}C NMR spectrum of ketiminoborane pyridone complex **12** (151 MHz, toluene-d_8).

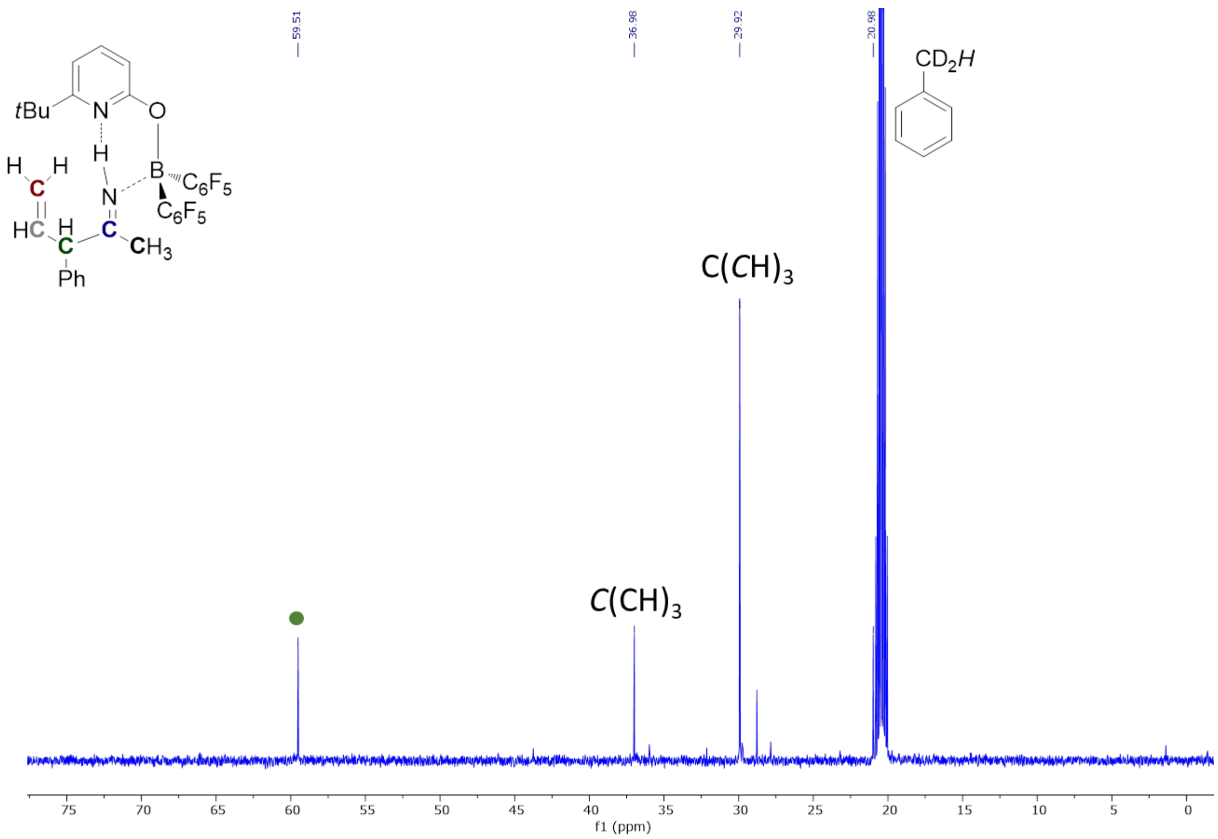

Figure SI 50: High field shift of the ^{13}C NMR spectrum of ketiminoborane pyridone complex **12** (151 MHz, toluene-d_8).

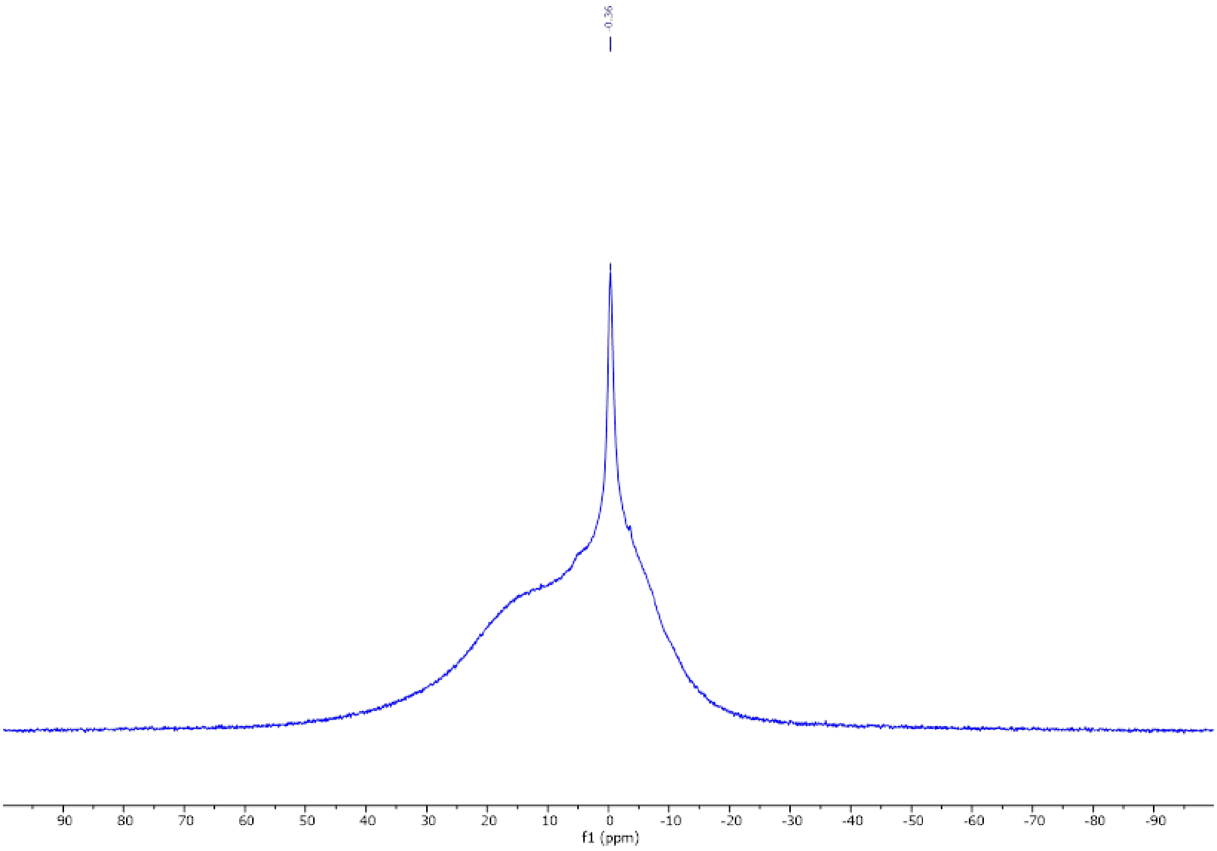

Figure SI 51: ^{11}B NMR spectrum of ketiminoborane pyridone complex **12** (193 MHz, toluene-d_8).

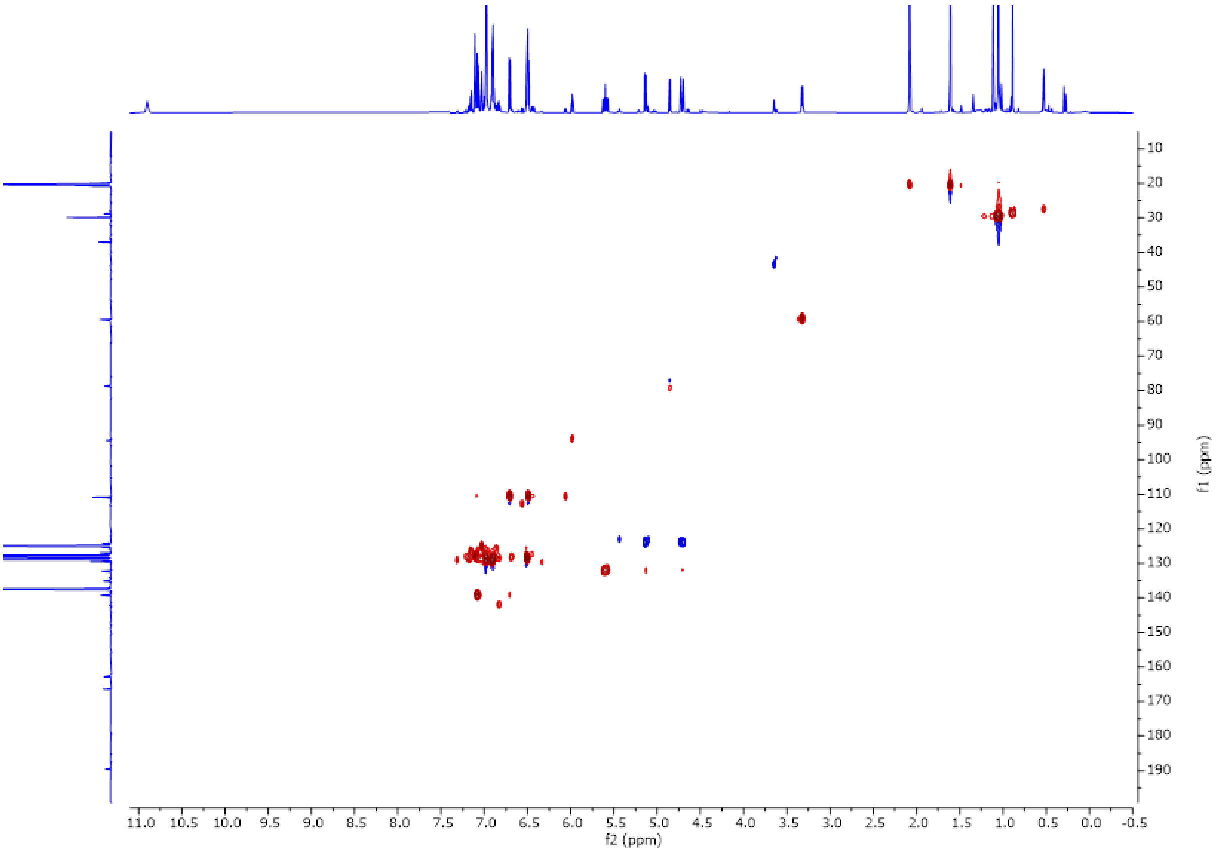

Figure SI 52: ^{1}H ^{13}C HSQC NMR spectrum of ketiminoborane pyridone complex **12** (151 MHz, toluene-d_8).

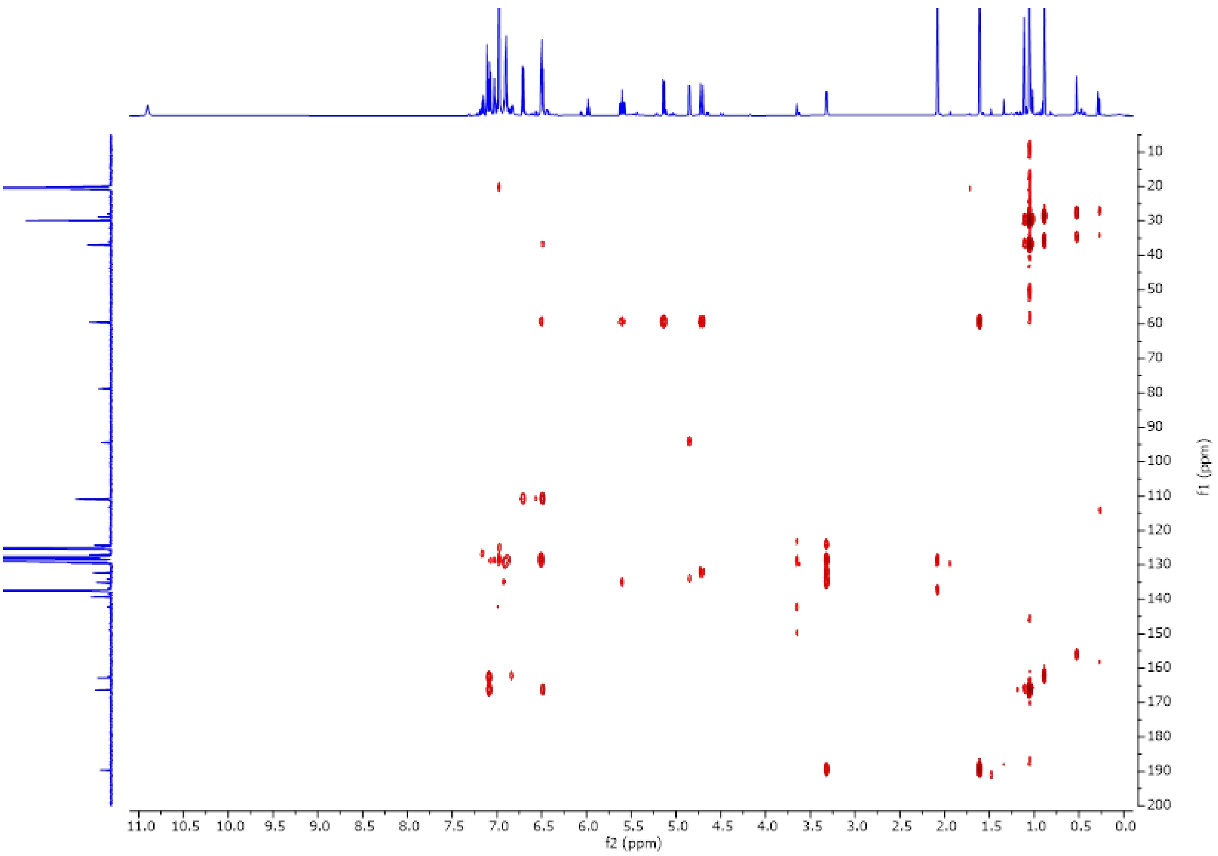

Figure SI 53: ^1H ^{13}C HMBC NMR spectrum of ketiminoborane pyridone complex **12** (151 MHz, toluene-d_8).

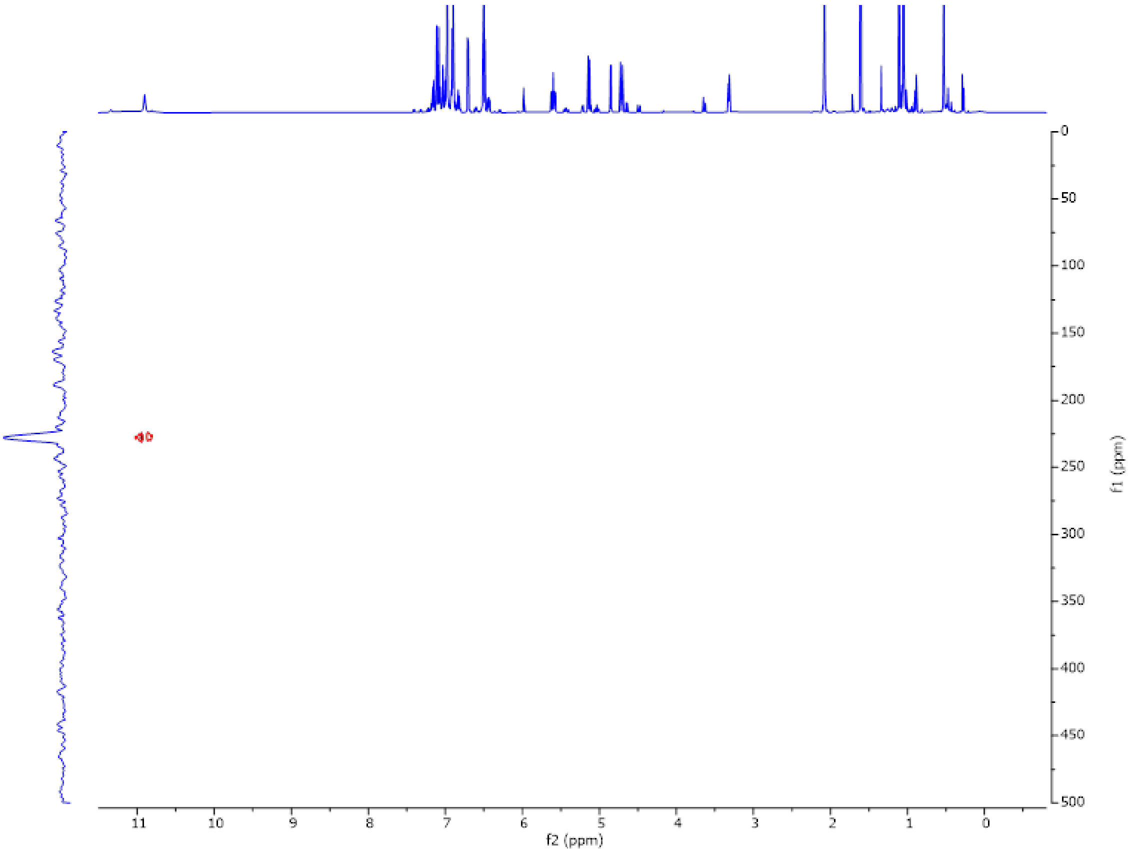

Figure SI 54: $^{1}H^{15}N$ HSQC NMR spectrum of ketiminoborane pyridone complex **12** (63 MHz, toluene-d_8).

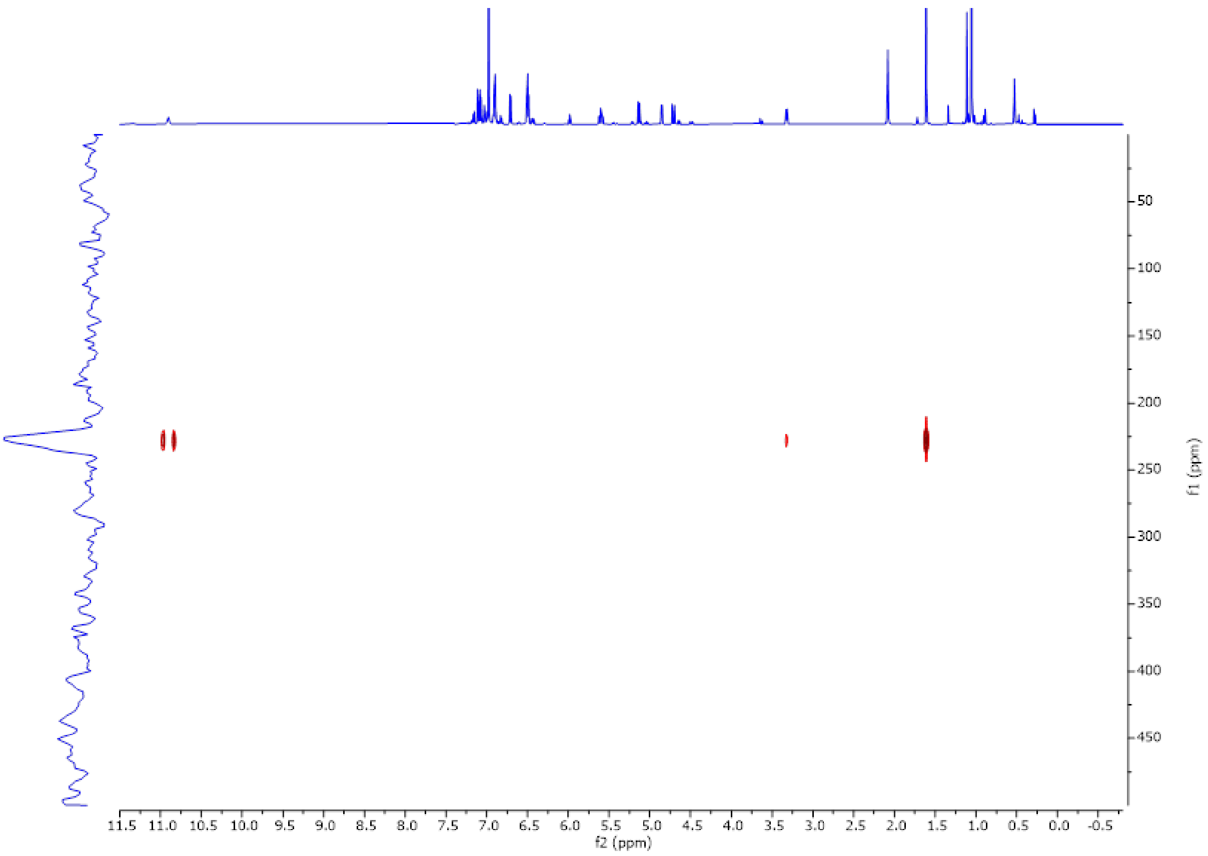

Figure SI 55: $^{1}H^{15}N$ HMBC NMR spectrum of ketiminoborane pyridone complex **12** (61 MHz, toluene-d_8).

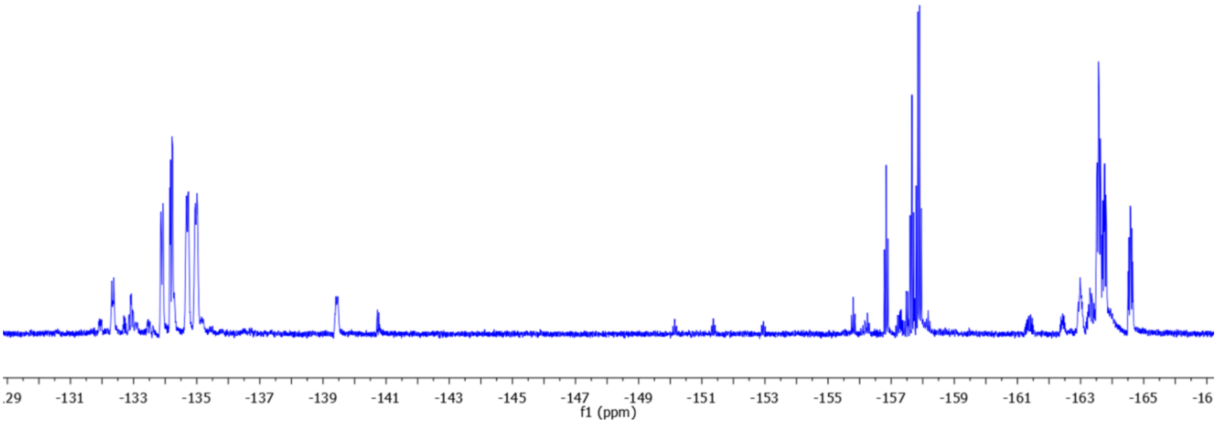

Figure SI 56: ^{19}F NMR spectrum of ketiminoborane pyridone complex **12** (377 MHz, benzene-d_6).

4 Synthesis of allenes

1-(trifluoromethyl)-4-vinylbenzene was synthesized from *p*-CF_3-benzaldehyde using a Wittig reaction (Scheme SI 3).[5]

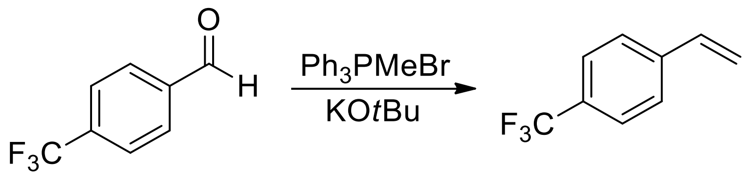

Scheme SI 3: Synthesis of *para*-CF_3 styrene from *para*-CF_3-benzaldehyde.

p-CF_3-phenylallene, *p*-Cl-phenylallene, phenylallen and 1-hexylallene were synthesized using the Doering-LaFlamme allene synthesis starting from the corresponding olefins (Scheme SI 4).[6]

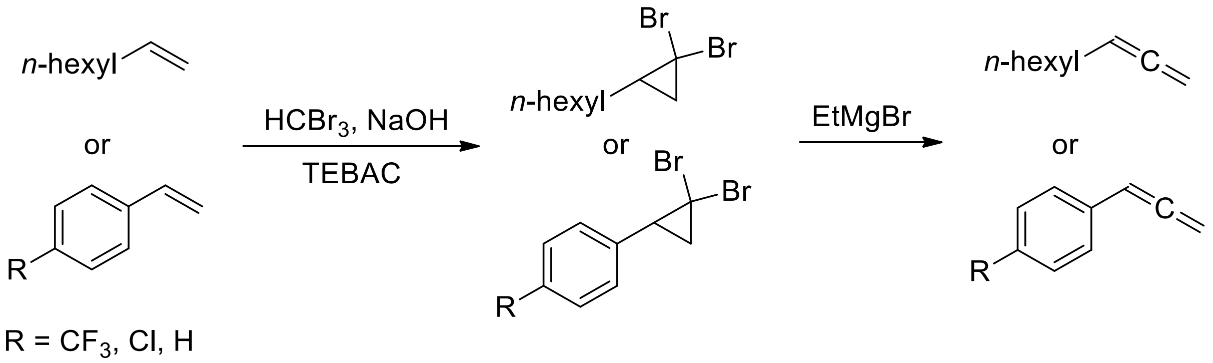

Scheme SI 4: Doering LaFlamme synthesis of different allenes starting from the corresponding olefins.

For the synthesis of 1,1-dibromo-2-hexylcyclopropane a slight variation of the above cited literature procedure was used.

In a flame dried three necked 500 mL flask with reflux condenser and stirring rod 1-octene (15.8 mL, 100.0 mmol, 1.00 equiv.), bromoform (13.0 mL, 148.7 mmol, 1.50 equiv.), triethylbenzylammonium-chloride (TEBAC) (501.1 mg, 2.20 mmol, 0.02 equiv.) were supended in DCM (10 mL). NaOH solution (16 g NaOH in 20 mL water, 400 mmol) was added and the reaction mixture was heated to 70 °C for 48 h. Water (100 mL) was added, the reaction mixture was transferred to a separation funnel and the aqueous phase was extracted with DCM four times. The combined organic phases were dried over $MgSO_4$ and the solvent was evaporated under reduced pressure. The product was purified by distillation under vacuum (30 mbar). The product was obtained as colourless oil in 39% yield (11.1 g). The analytical data are in accordance with the literature.[7]

p-*t*Bu-phenylallene was synthesized using the Crabbé reaction analogous to a literature known protocol (Scheme SI 5).[8]

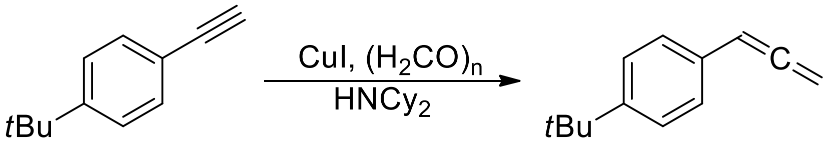

Scheme SI 5: Synthesis of *para*-*t*Bu-phenylallen using the Crabbé reaction starting from the corresponding acetylene.

1-Adamantylacetylene was synthesized starting from 1-bromoadamantan in a literature known two-step procedure (Scheme SI 6).[9]

A modified procedure for a microwave assisted Crabbé reaction was adapted for the synthesis of (1-adamantyl)allene starting from 1-adamantylacetylene (Scheme SI 6).[10]

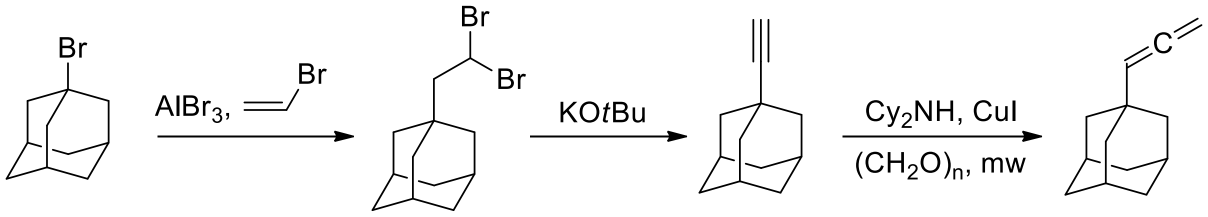

Scheme SI 6: Synthesis of 1-adamantylallene starting from 1-bromoadamantane.

A 35 mL microwave tube was charged with CuI (0.70 mg, 3.68 mmol, 0.37 equiv.), dicyclohexylamine (4.00 mL, 20.1 mmol, 2.01 equiv.), and dry degassed 1,4-dioxane (10 mL). Then, (1-adamantyl)acetylene (1.60 g, 10.0 mmol, 1.00 equiv.) and paraformaldehyde (2.25 g, 25.0 mmol, 2.50 equiv.) were added. The pale yellow white suspension was heated in a microwave to 140 °C for 20 min (300 W, 10 min ramp up). Afterwards, it was cooled to rt and filtered. The microwave tube was rinsed with *n*-pentane (3 x 5 mL). The yellow solution was washed with water (8 × 30 mL), dried over Na_2SO_4, filtered, and concentrated under reduced pressure. A yellow oil was obtained. It was purified by flash column chromatography (SiO_2: 200 g, *n*-pentane) (R_f = 0.7). A colourless oil was obtained (1.09 g, 6.26 mmol, 63 %).

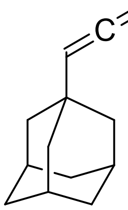

^{1}H NMR (400 MHz, Chloroform-*d*) δ 4.99 (t, *J* = 6.7 Hz, 1H, *H*C=C=C), 4.72 (d, *J* = 6.7 Hz, 2H, C=C=CH_2), 1.98 (s, 1H, $C_{Ad}H$), 1.81 – 1.55 (m, 9H, $C_{Ad}H_2$).

^{13}C{^{1}H} NMR (101 MHz, Chloroform-*d*) δ 206.6 (C=*C*=C), 102.0 (H*C*=C=CH$_2$), 76.4 (HC=C=*C*H$_2$), 43.0 (C_q-(*C*H$_2$)$_3$), 36.9 (*C*H$_2$), 33.2 (*C*$_q$), 28.9 (*C*$_{Ad}$H).

HRMS (ESI) m/z [M+H$^+$] calc. for $C_{13}H_{19}^+$: 175.1481; found: 175.1483

4.1.1 NMR spectra of (1-adamantyl)allene

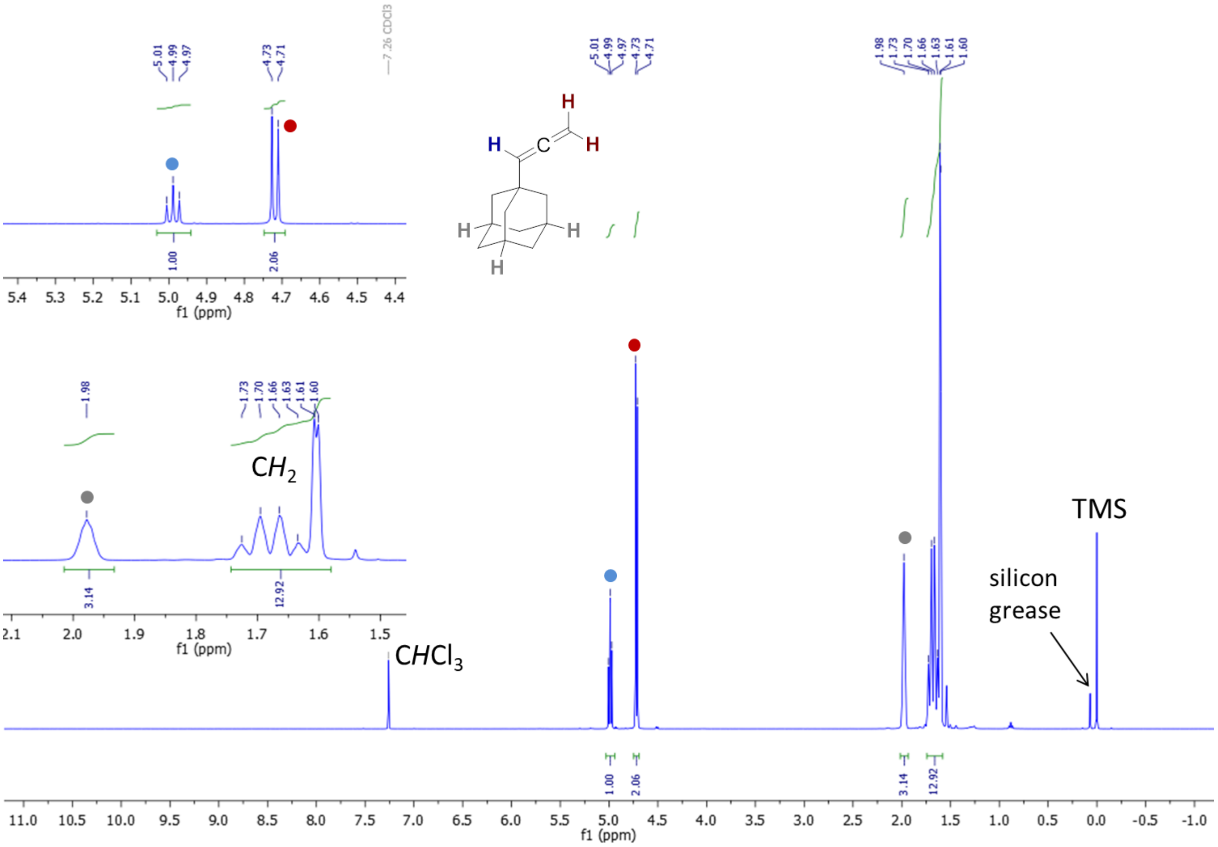

Figure SI 57: ^{1}H NMR spectrum of (1-adamantyl)allene (400 MHz, Chloroform-*d*).

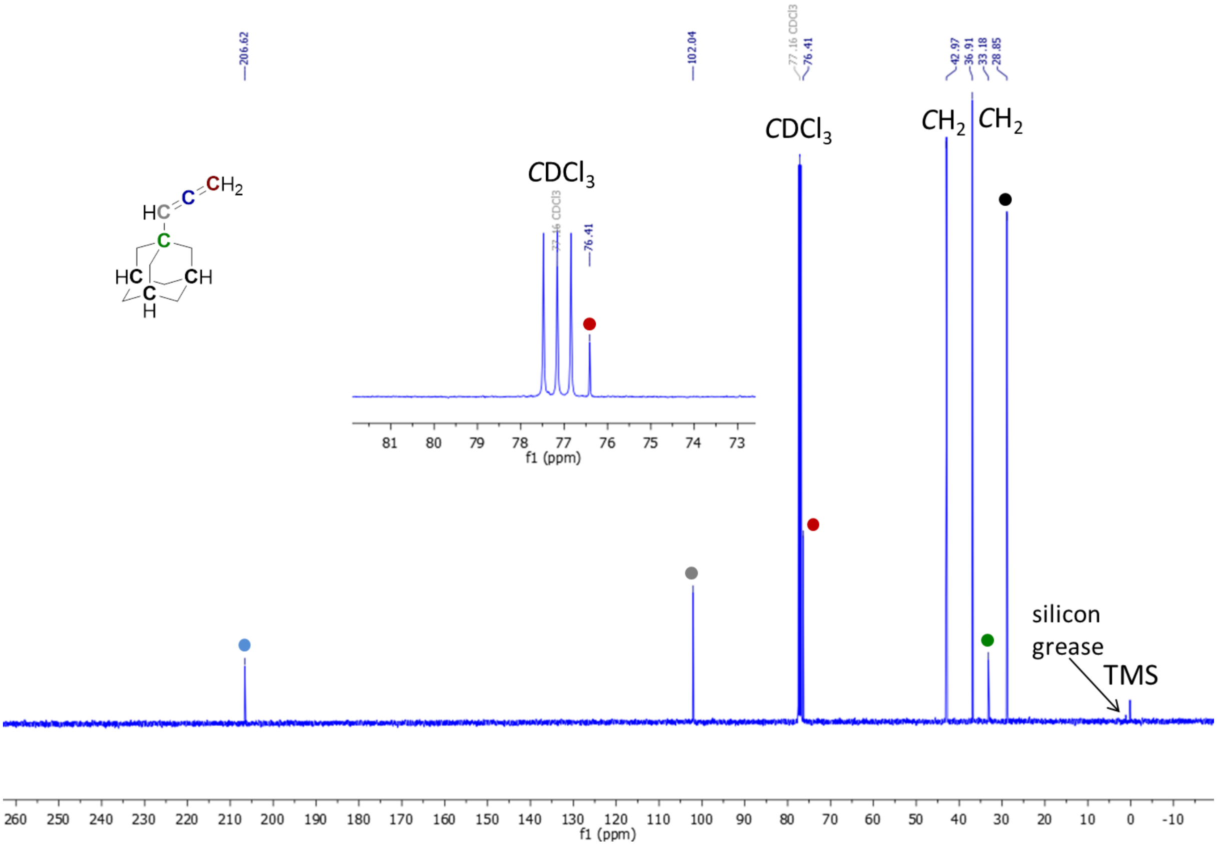

Figure SI 58: $^{13}C\{^{1}H\}$NMR spectrum of (1-adamantyl)allene (101 MHz, Chloroform-*d*).

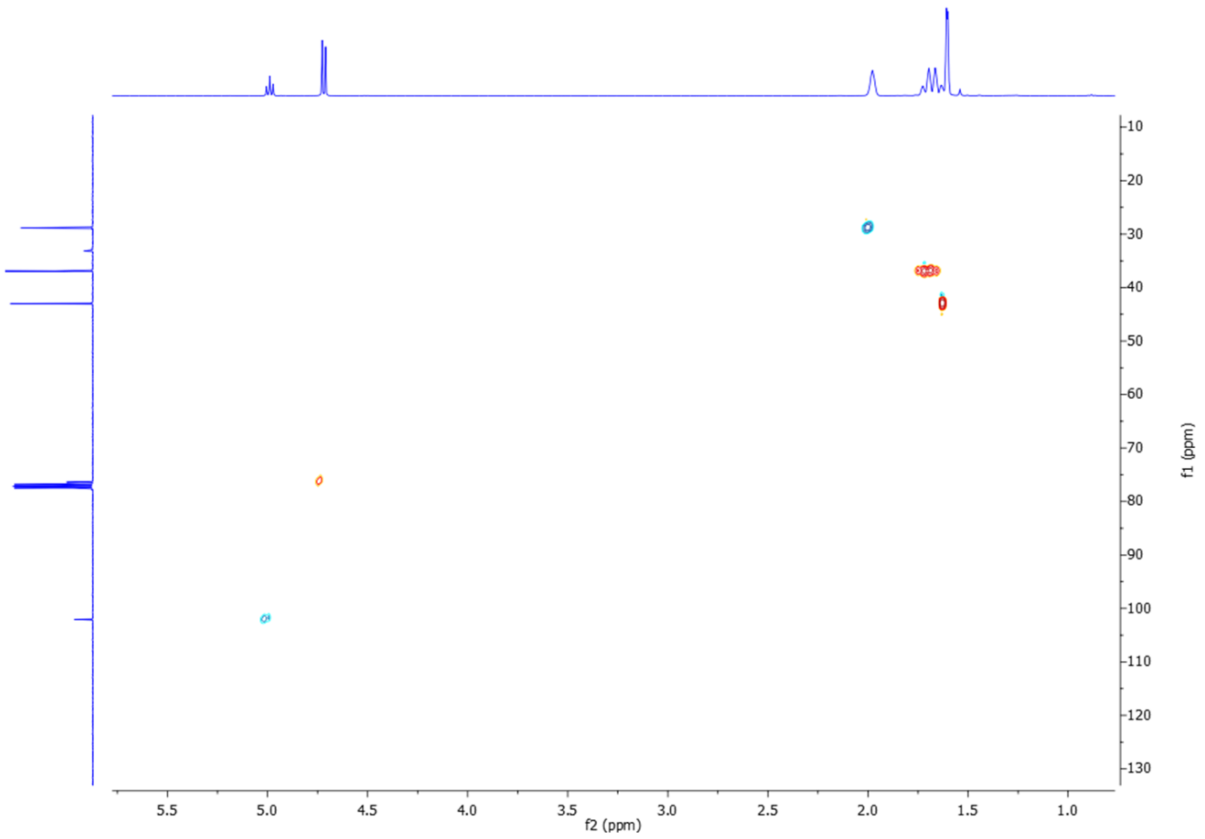

Figure SI 59: HSQC NMR spectrum of (1-adamantyl)allene (101 MHz, Chloroform-*d*).

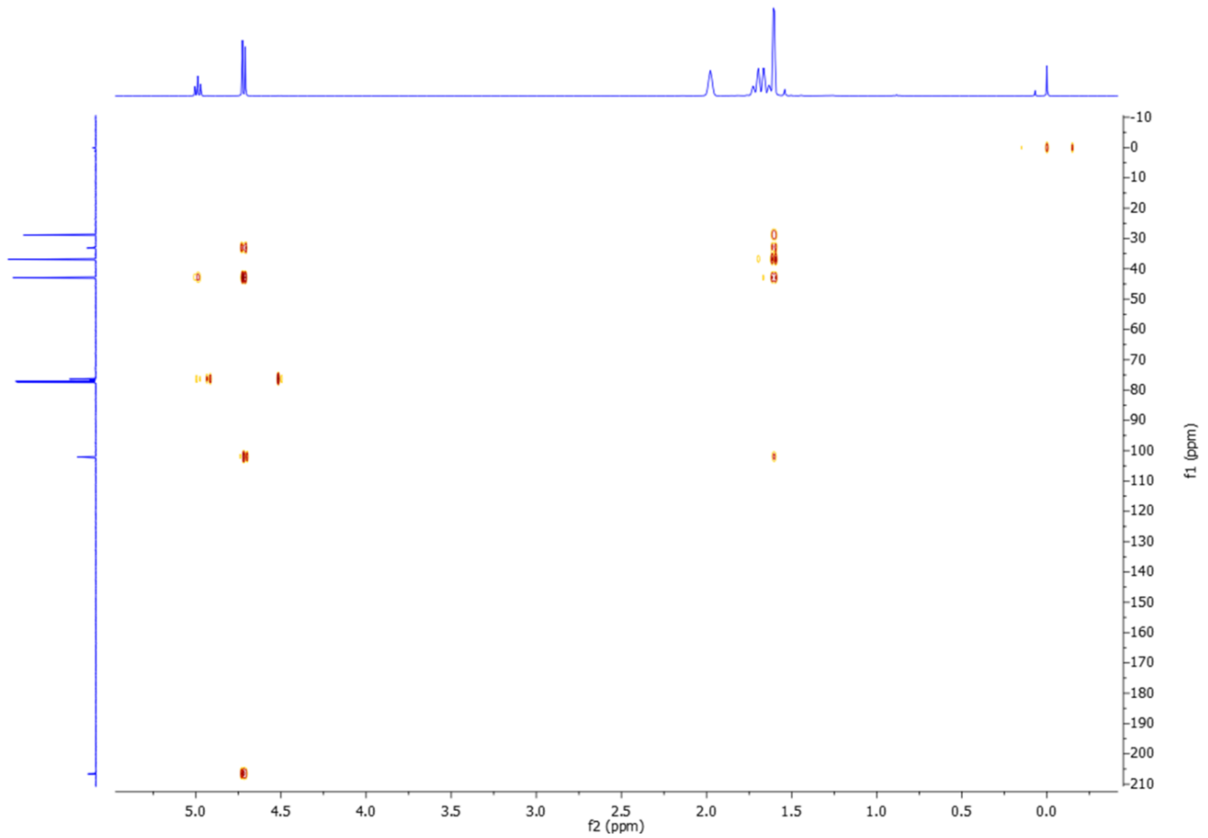

Figure SI 60: HMBC NMR spectrum of (1-adamantyl)allene (101 MHz, Chloroform-*d*).

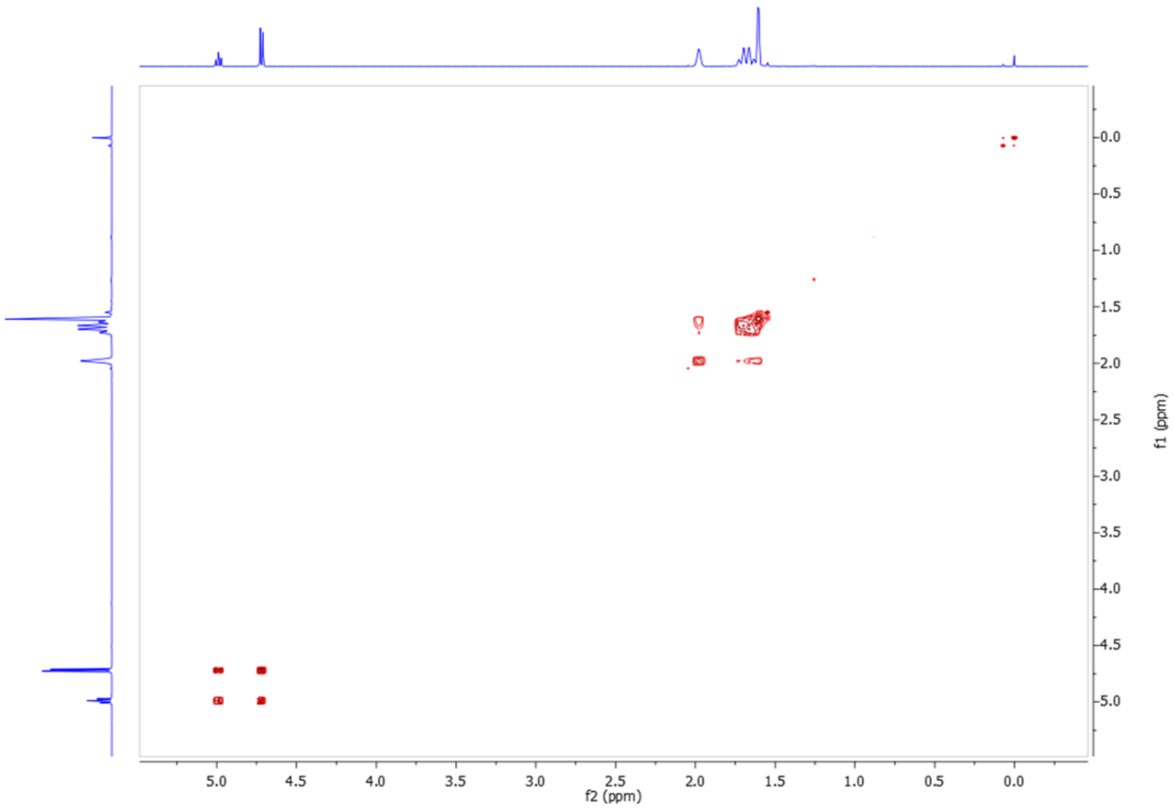

Figure SI 61: COSY NMR spectrum of (1-adamantyl)allene (400 MHz, Chloroform-*d*).

5 Catalytic allylation of Nitriles

5.1 General Procedure for the catalysis experiments

Inside the glovebox stock solution of 6-*tert*-butyl-2-pyridone **5** (75.6 mg, 0.50 mmol, 0.1 M) and Piers borane **6** (190 mg, 0.55 mmol, 0.11 M) in benzene-d_6 (5.00 mL) was prepared and stored in the freezer at –35 °C (it slowly released hydrogen and pyridonate borane **3** was formed; this did not impact the yield of the reactions). Catalyst solution (0.3 mL ≡ 10 mol% / 11 mol%) was put into a 60 mL Schlenk tube with J. Young valve and rinsed with dry benzene (1.00 mL). In a separate vial, tris(perfluorophenyl)borane (153.6 mg, 0.300 mmol, 1.00 equiv.) was dissolved in dry benzene (3.00 mL) and acetonitrile (15.7 µL, 0.300 mmol, 1.00 equiv.) was added. This solution was transferred to the Schlenk tube and rinsed two times with benzene (2x1.00 mL). The allene (0.360 mmol, 1.20 equiv.) was added, the tube was sealed, and taken out of the glovebox. It was subject to three freeze-pump-thaw cycles and connected to a H_2 bomb *via* a gas hose. The hose was purged several times with H_2. Afterwards, the Schlenk tube was pressurized with 1.1 bar of H_2, put into an 80 °C preheated oil bath, and stirred for the reported amount of time. ***Caution: Reaction builds up pressure, a shield has to be used.*** Afterwards, the tube was cooled to room temperature, the excess pressure was released, and the solvent was evaporated under reduced pressure. The crude product was purified by column chromatography.

5.2 General Procedure for the rearrangement of the allylimine borane complex to the vinylimine borane complex

Some of the allylimine borane complexes showed isomerization to the corresponding vinylimine borane complexes when exposed to silica during flash chromatography. Signals corresponding to the isomerized product were not detectable in the crude reaction mixture by ^{1}H NMR in all cases (Figure SI 64). To further support that the isomerization is due to exposure to silica, a 2D TLC experiment was performed (Figure SI 62, Figure SI 63).

We noticed that the isomerization during column chromatography is more pronounced when an *n*-hexane and ethyl acetate mixture was used as eluent. When a mixture of *n*-hexane and dichloromethane was used, we observed less isomerization but lower separation performance.

The isomerization can be quantitatively achieved by the addition of triethylamine. For this purpose the allylimine borane complex (about 100 mg depending on the yield of the catalytic allylation) was dissolved in THF (2.00 mL). Triethylamine (1.00 equiv.) was added and the reaction mixture was stirred for 30 min at room temperature. Afterwards the solvent was evaporated under reduced pressure. Analysis by ^{1}H NMR spectroscopy showed quantitative conversion for every substrate. This is also reflected in the isolated yield for the isomerization of the allylimine borane complex of nona-1,2-diene **18** (15.5 mg, 0.11 mmol) to the corresponding vinylimine borane complex **18’** (95% isolated yield). For an analytical sample of the vinylimine borane complexes the reaction mixtures were purified using column chromatography.

5.2.1 2D TLC experiment of the isomerization of 13 to 13’

To show that the isomerization of **13** to **13’** is a result of exposure to silica, a sample of the 93:7 mixture of **13** to **13’** obtained after column chromatography was dotted on a silica TLC using a *n*-hexane and ethyl acetate (4:1) mixture as eluent. After the first run the TLC shows spots under UV irradiation (254 nm) corresponding to **13** and **13**’ (Figure SI 62). A second run was performed with the TLC turned 90° showing the isomerization (Figure SI 63).

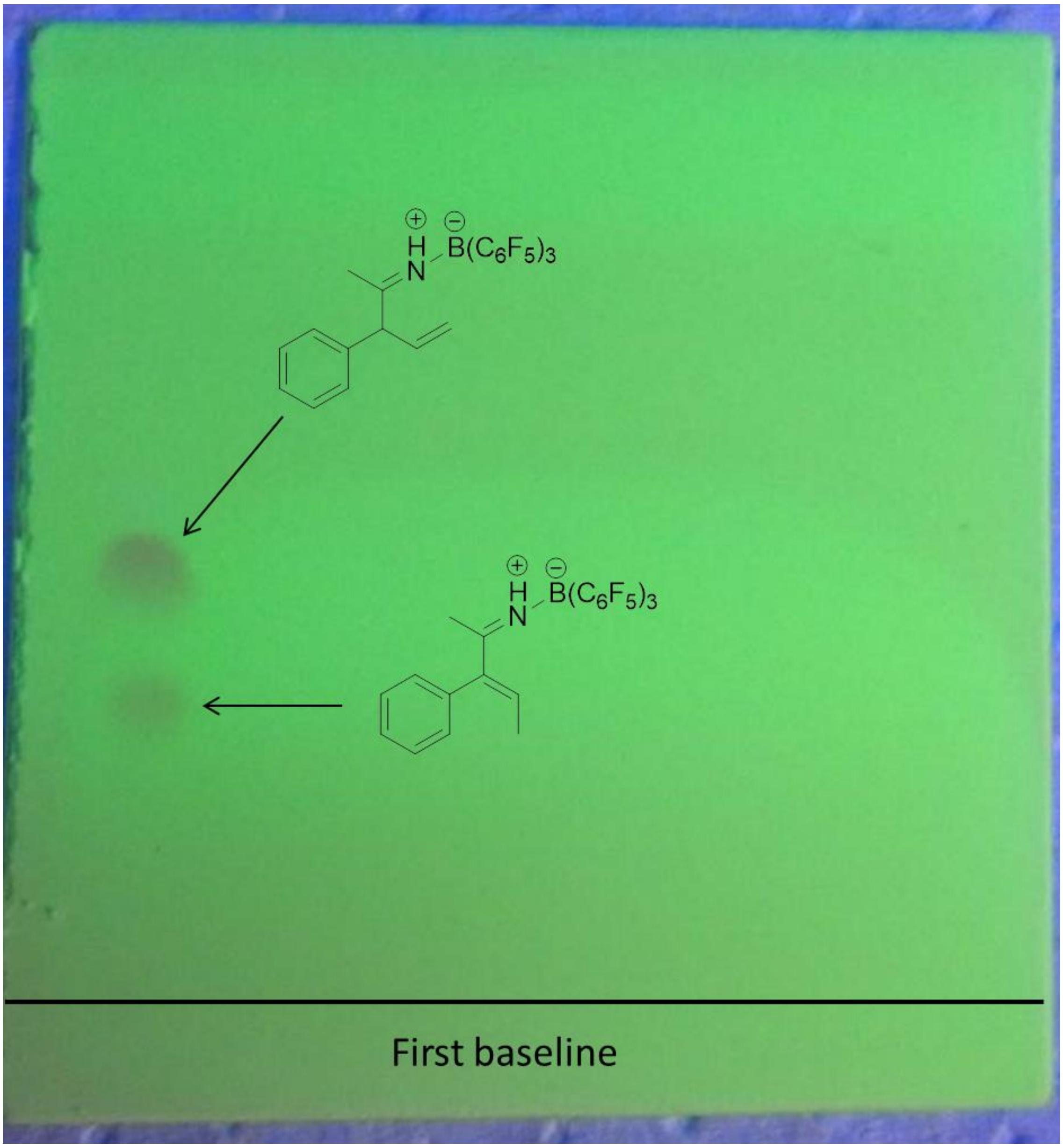

Figure SI 62 2D TLC experiment of the isomerization of **13** to **13’** after the first run using a 93:7 reaction mixture of **13** to **13’** (4:1 *n*-hexane/EtOAc mixture as eluent).

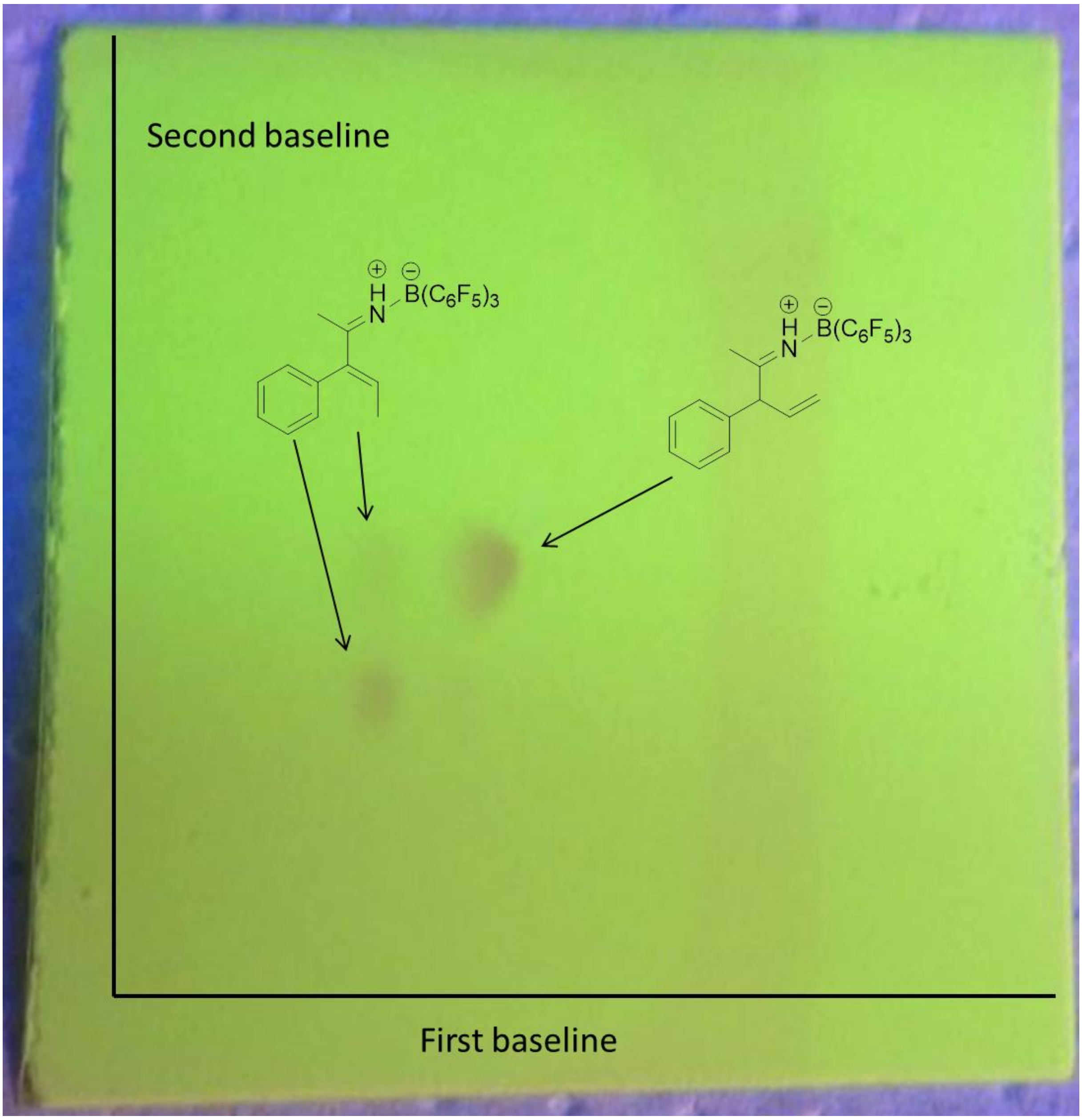

Figure SI 63: 2D TLC experiment of the isomerization of **13** to **13** after the second run using a 93:7 reaction mixture **13** to **13'** (4:1 *n*-hexane/EtOAc mixture as eluent).

5.3 Synthesis and characterization of allylimine borane complexes and the corresponding vinylimine borane complexes

5.3.1 3-Phenylpent-4-en-2-imine tris(perfluorophenyl)borane complex 13

3-Phenylpent-4-en-2-imine tris(perfluorophenyl)borane complex **13** was synthesized according to general procedure (chapter 5.1) with phenylallene (46.0 µL, 0.36 mmol, 1.20 equiv.) and a reaction time of 16 h.

The crude product was purified by flash column chromatography (13 g silica, *n*-hexane/EtOAc, 19:1) (R_f = 0.3).

The purification by column chromatography leads to an isomerization of the olefinic double bond. The allylimine borane **13** and the corresponding vinylimine borane **13'**were isolated as a mixture in a ratio of 93:7 in 65% yield (130.4 mg).

For an analytical sample of 3-phenylpent-4-en-2-imine tris(perfluorophenyl)borane complex **13**, the mixture was again purified by flash column chromatography.

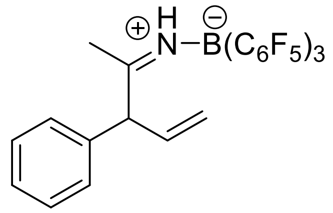

^{1}H NMR (400 MHz, Chloroform-*d*) δ 9.60 (s, 1H, N*H*), 7.49 – 7.38 (m, 3H, Ar-*H*), 7.13 (dd, *J* = 7.8, 1.4 Hz, 2H, Ar-*H*), 6.04 (ddd, *J* = 17.5, 10.3, 7.6 Hz, 1H, C_{sp2}-*H*), 5.68 (d, *J* = 10.3 Hz, 1H, C_{sp2}-*H*), 5.38 (d, *J* = 17.2 Hz, 1H, C_{sp2}-*H*), 4.46 (d, *J* = 7.5 Hz, 1H, C_{sp3}-*H*), 2.00 (s, 3H, C*H*$_3$).

^{13}C{^{1}H} NMR (101 MHz, Chloroform-*d*) δ 192.6 (*C*=N), 134.6 (Ar-C_q), 131.9 (C_{sp2}), 130.2 (Ar-*C*), 129.5 (Ar-*C*), 128.7 (Ar-*C*), 125.1 ($C_{sp2}H_2$), 60.9 (bn-*C*), 21.7 (*C*H$_3$).

Remark: The ^{13}C{^{1}H} NMR spectrum shows broad signals with low intensity at 149.1, 146.7, 141.5, 138.5, 136.1 ppm which can be tentatively assigned to the pentafluorophenyl groups.

^{19}F NMR (377 MHz, Chloroform-*d*) δ -134.22 (d, *J* = 22.2 Hz, *o-F*), -156.43 (d, *J* = 20.4 Hz, *p-F*), -163.01 (td, *J* = 24.3, 8.5 Hz, *m-F*).

^{11}B NMR (128 MHz, Chloroform-*d*) δ -9.0.

HRMS (ESI) m/z [M+Na$^+$] calc. for $C_{29}H_{13}BF_{15}NNa^+$: 694.0794; found: 694.0793.

5.3.2 (*E*)-3-Phenylpent-3-en-2-imine tris(perfluorophenyl)borane complex 13'

(*E*)-3-Phenylpent-3-en-2-imine tris(perfluorophenyl)borane complex **13'** was synthesized according to general procedure (chapter 5.2). It was purified by flash column chromatography (13 g silica, *n*-hexane/EtOAc, 9:1).

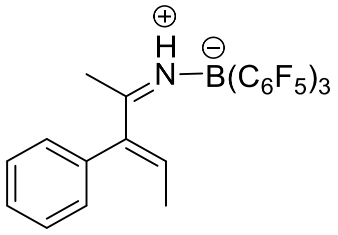

^{1}H NMR (600 MHz, Chloroform-*d*) δ 8.87 (s, 1H, N*H*), 7.57 – 7.48 (m, 3H, Ph-*H*), 7.16 – 7.12 (m, 2H, Ph-*H*), 7.09 (q, *J* = 7.0 Hz, 1H, C_{sp2}*H*), 2.28 (s, 3H, C*H*$_3$), 1.84 (d, *J* = 7.0 Hz, 3H, C*H*$_3$).

^{13}C{^{1}H} NMR (101 MHz, Chloroform-*d*) δ 181.6 (*C*=N), 145.1 (*C*$_{sp2}$H), 140.9 (*C*$_{q,}$C$_{sp2}$H), 130.7 (Ar-*C*$_q$), 130.2 (Ar-*C*), 130.1 (Ar-*C*), 130.0 (Ar-*C*), 17.7 (H$_3$*C*C=N), 17.3 (*C*H$_3$).

Remark: The ^{13}C{^{1}H} NMR spectrum shows broad signals with low intensity at 149.1, 146.7, 141.3, 138.8, 135.9 ppm which can be tentatively assigned to the pentafluorophenyl groups.

^{11}B NMR (128 MHz, Chloroform-*d*) δ -9.49.

^{19}F NMR (377 MHz, Chloroform-*d*) δ -134.35 (d, *J* = 21.0 Hz, o-*F*), -157.04 (t, *J* = 20.4 Hz, p-*F*), -163.46 (td, *J* = 23.6, 8.0 Hz, m-*F*).

HRMS (ESI) m/z [M+H$^+$] calc. for $C_{29}H_{13}BF_{15}NNa^+$: 694.0794; 694.0796.

Remark: The configuration of the double bond was determined by NOESY experiment and X-Ray diffractometry. The NOESY spectrum shows correlation signals between the singlet of the methyl group neighbouring the imine moiety and the olefinic hydrogen, and between the doublet of the methyl group next to the double bound and the *ortho* hydrogens of the phenyl ring (Figure SI 81, Figure SI 82).

5.3.3 3-(4-(chloro)phenyl)pent-4-en-2-imine tris(perfluorophenyl)borane complex 14

3-(4-(Chloro)phenyl)pent-4-en-2-imine tris(perfluorophenyl)borane complex was synthesized according to general procedure (chapter 5.1) with 1-(chloro)-4-(propa-1,2-dien-1-yl)benzene and a reaction time of 5 d.

The crude product was purified by flash column chromatography (20 g silica, *n*-hexane/DCM, 5:1).

The allylimine borane **14** and the corresponding vinylimine borane **14'** were isolated as a mixture in a ratio of 92:8 in 72% yield (151.5 mg).

Albeit several attempts to purify the product *via* column chromatography and crystallisation we were not able to completely separate **14** from **14'**.

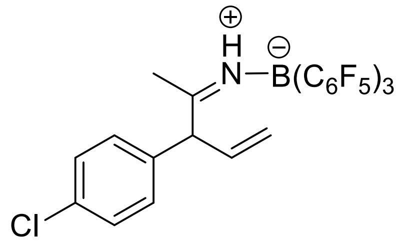

^{1}H NMR (400 MHz, Chloroform-*d*) δ 9.65 (s, 1H, N*H*), 7.43 (d, *J* = 8.5 Hz, 2H, Ar-*H*), 7.07 (d, *J* = 8.4 Hz, 2H, Ar-*H*), 6.00 (ddd, *J* = 17.4, 10.3, 7.4 Hz, 1H, C_{sp2}-*H*), 5.72 (d, *J* = 10.3 Hz, 1H, C_{sp2}-*H*), 5.38 (d, *J* = 17.2 Hz, 1H, C_{sp2}-*H*), 4.44 (m, *J* = 7.4 Hz, 1H, bn-*H*), 1.99 (s, 3H, CH_3).

^{13}C{^{1}H} NMR (101 MHz, Chloroform-*d*) δ 192.1 (*C*=N), 135.8 (Ar-C_q), 132.6 (Ar-C_q), 131.4 ($C_{sp2}H_2$), 130.4 (Ar-*C*), 130.0 (Ar-*C*), 125.7 (C_{sp2}H), 60.1 (bn-*C*), 21.7 (CH_3).

Remark: The ^{13}C{^{1}H} NMR spectrum shows broad signals with low intensity at 149.1, 146.7, 141.6, 139.1, 138.5 and 136.0 ppm which can be tentatively assigned to the pentafluorophenyl groups.

^{11}B NMR (128 MHz, Chloroform-*d*) δ -8.93.

^{19}F NMR (377 MHz, Chloroform-*d*) δ -134.28 (d, *J* = 21.8 Hz, o-*F*), -156.12 (t, *J* = 20.4 Hz, m-*F*), -162.82 (td, *J* = 24.3, 8.4 Hz, p-*F*).

HRMS (ESI) m/z [M+Na$^+$] calc. for $C_{29}H_{12}BClF_{15}NNa^+$: 728.0404; found: 728.0378.

5.3.4 (*E*)-3-(4-chlorophenyl)pent-3-en-2-imine tris(perfluorophenyl)borane complex 14’

(*E*)-3-(4-Chlorophenyl)pent-3-en-2-imine tris(perfluorophenyl)borane complex **14’** was synthesized according to general procedure (chapter 5.2). It was purified by flash column chromatography (15 g silica, *n*-hexane/DCM, 5:1).

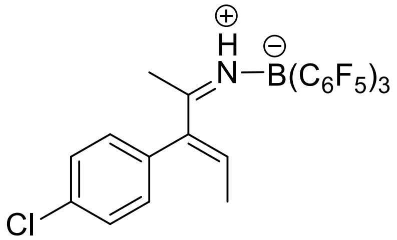

^{1}H NMR (400 MHz, Chloroform-*d*) δ 8.85 (s, 1H, N*H*), 7.54 (d, *J* = 8.4 Hz, 2H, Ar-*H*), 7.15-7.08 (m, H, Ar-*H*, C_{sp2}-*H*), 2.29 (s, 3H, CH_3), 1.84 (d, *J* = 7.0 Hz, 3H, CH_3).

^{13}C{^{1}H} NMR (101 MHz, Chloroform-*d*) δ 181.6 (*C*=N), 145.9 (C_{sp2}H), 139.8 ($C_{sp2,q}$), 136.6 (Ar-C_q), 131.5 (Ar-*C*), 130.5 (Ar-*C*), 129.1 (Ar-C_q), 17.9 (CH_3), 17.4 (CH_3).

Remark: The ^{13}C{^{1}H} NMR spectrum shows broad signals with low intensity at 149.1, 146.6, 141.3, 138.9, 138.4 and 135.9 ppm which can be tentatively assigned to the pentafluorophenyl groups.

^{11}B NMR (128 MHz, Chloroform-*d*) δ -9.38.

^{19}F NMR (377 MHz, Chloroform-*d*) δ -134.45 (d, *J* = 21.0 Hz,. o-*F*), -156.81 (t, *J* = 20.4 Hz, p-*F*), -163.29 (td, *J* = 23.4, 7.7 Hz, m-*F*).

HRMS (ESI) m/z [M-H$^+$] calc. for $C_{29}H_{12}BClF_{15}N^-$: 704.0439; found: 704.0397.

5.3.5 3-(4-(Trifluoromethyl)phenyl)pent-4-en-2-imine tris(perfluorophenyl)borane complex 15

3-(4-(Trifluoromethyl)phenyl)pent-4-en-2-imine tris(perfluorophenyl)borane complex **15** was synthesized according to general procedure (chapter 5.1) with 1-(trisfluoromehtyl)-4-(1,2-propadien-1-yl)-benzene (66.3 mg, 0.36 mmol) and a reaction time of 5 d.

The crude product was purified by flash column chromatography (20 g silica, *n*-hexane/DCM, 5:1) (R_f = 0.15).

The allylimine borane **15** and the corresponding vinylimine borane **15'** were isolated as a mixture in a ratio of 95:5 in 80% yield (177.8 mg).

Albeit several attempts to purify the product *via* column chromatography and crystallisation we were not able to completely separate **15** from **15'**.

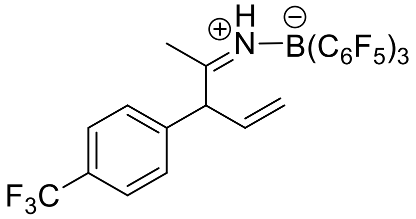

^{1}H NMR (400 MHz, Chloroform-*d*) δ 9.70 (s, 1H, N-*H*), 7.73 (d, *J* = 8.1 Hz, 2H, Ar-*H*), 7.29 (d, *J* = 8.1 Hz, 2H, Ar-*H*), 6.03 (ddd, *J* = 17.4, 10.3, 7.4 Hz, 1H, C_{sp2}-*H*), 5.76 (d, *J* = 10.3 Hz, 1H, C_{sp2}-*H*), 5.42 (d, *J* = 17.2 Hz, 1H, C_{sp2}-*H*), 4.56 (d, *J* = 7.4 Hz, 1H, bn-*H*), 2.02 (s, 3H, CH_3).

^{13}C{^{1}H} NMR (101 MHz, Chloroform-*d*) δ 191.8 (*C*=N), 138.25 (Ar-C_q), 131.9 (q, *J* = 33.1 Hz, Ar-C_q), 131.0 (C_{sp2}H), 129.2 (Ar-*C*), 127.2 (q, *J* = 3.7 Hz, Ar-*C*), 126.2 ($C_{sp2}H_2$), 123.7 (q, *J* = 272.4 Hz, CF_3), 60.5 (bn-*C*), 21.8 (CH_3).

Remark: The ^{13}C{^{1}H} NMR spectrum shows broad signals with low intensity at 149.1, 146.7, 141.6, 139.1, 138.6 and 136.1 ppm which can be tentatively assigned to the pentafluorophenyl groups.

^{11}B NMR (128 MHz, Chloroform-*d*) δ -8.78.

^{19}F NMR (377 MHz, Chloroform-*d*) δ -134.4 (d, *J* = 21.6 Hz), -156.20 (t, *J* = 20.3 Hz), -162.90 (td, *J* = 24.3, 8.3 Hz).

HRMS (ESI) m/z [M-H$^+$] calc. for $C_{30}H_{11}BF_{18}N^-$: 738.0691; found: 738.0610.

5.3.6 (*E*)-3-(4-(trifluoromethyl)phenyl)pent-3-en-2-imine tris(perfluorophenyl)borane complex 15'

(*E*)-3-(4-(Trifluoromethyl)phenyl)pent-3-en-2-imine tris(perfluorophenyl)borane complex **15'** was synthesized according to general procedure (chapter 5.2). It was purified by flash column chromatography (15 g silica, *n*-hexane/DCM, 5:1).

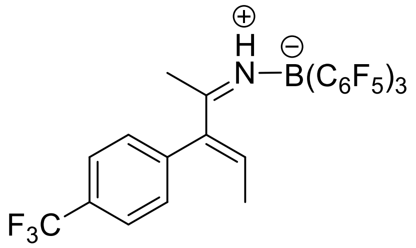

^{1}H NMR (400 MHz, Chloroform-*d*) δ 8.77 (s, 1H, N*H*), 7.83 (d, *J* = 8.0 Hz, 2H, Ar-*H*), 7.32 (d, *J* = 8.0 Hz, 2H, Ar-*H*), 7.15 (q, *J* = 7.0 Hz, 1H, C_{sp2}-*H*), 2.29 (s, 3H, CH_3), 1.86 (d, *J* = 7.1 Hz, 3H, CH_3).

^{13}C{^{1}H} NMR (101 MHz, Chloroform-*d*) δ 181.5 (*C*=N), 146.0 (C_{sp2}H), 139.7 ($C_{sp2,q}$), 134.8 (Ar-C_q), 132.6 (q, *J* = 33.2 Hz, Ar-C_q), 130.8 (Ar-*C*), 127.2 (q, *J* = 3.6 Hz, Ar-*C*), 123.6 (q, *J* = 272.5 Hz, *C*F_3), 18.0 (*C*H_3), 17.4 (*C*H_3).

Remark: The ^{13}C{^{1}H} NMR spectrum shows broad signals with low intensity at 149.0, 146.6, 141.3, 138.9, 138.4 and 135.9 ppm which can be tentatively assigned to the pentafluorophenyl groups.

^{11}B NMR (128 MHz, Chloroform-*d*) δ -9.35.

^{19}F NMR (377 MHz, Chloroform-*d*) δ -63.28 (s, C*F*$_3$), -134.54 (d, *J* = 21.0 Hz,. o-*F*), -156.60 (t, *J* = 20.4 Hz, p-*F*), -163.03 – -163.48 (m, m-*F*).

HRMS (ESI) m/z [M+Na$^+$] calc. for $C_{30}H_{12}BF_{18}NNa^+$: 762.0668; found: 762.0668.

5.3.7 3-(4-(tert-Butyl)phenyl)pent-4-en-2-imine tris(perfluorophenyl)borane complex 16

3-(4-(tert-Butyl)phenyl)pent-4-en-2-imine tris(perfluorophenyl)borane complex **16** was synthesized according to general procedure (chapter 5.1) with 1-(tert-butyl)-4-(propa-1,2-dien-1-yl)benzene (62.0 µL, 0.36 mmol) and a reaction time of 16 h.

The crude product was purified by flash column chromatography (15 g silica, *n*-hexane/DCM, 4:1). The product was isolated as a single isomer in 49% yield (106.8 mg).

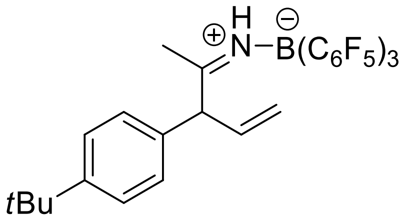

^{1}H NMR (400 MHz, Chloroform-*d*) δ 9.51 (s, 1H, N*H*), 7.46 (d, *J* = 8.4 Hz, 2H, Ar-*H*), 7.07 (d, *J* = 8.3 Hz, 2H, Ar-*H*), 6.03 (ddd, *J* = 17.6, 10.2, 7.8 Hz, 1H, C_{sp2}-*H*), 5.63 (d, *J* = 10.2 Hz, 1H, C_{sp2}-*H*), 5.35 (d, *J* = 17.2 Hz, 1H, C_{sp2}-*H*), 4.44 (d, *J* = 7.8 Hz, 1H, bn-*H*), 2.03 (s, 3H, CH_3), 1.32 (s, 9H, C(CH_3)$_3$).

^{13}C{^{1}H} NMR (101 MHz, Chloroform-*d*) δ 192.9 (*C*=N), 153.0 (C_{sp2}H), 132.1 (C_{sp2}H), 130.6 (Ar-C_q), 128.5 (Ar-*C*), 127.2 (Ar-*C*), 124.3 ($C_{sp2}H_2$), 60.6 (bn-*C*), 34.9 (*C*(CH$_3$)$_3$), 31.3 (C(*C*H$_3$)$_3$), 21.7 (*C*H$_3$).

Remark: The $^{13}C\{^1H\}$ NMR spectrum shows broad signals with low intensity at 149.1, 146.7, 141.4, 138.5, 135.9 ppm which can be tentatively assigned to the pentafluorophenyl groups.

^{19}F NMR (377 MHz, Chloroform-*d*) δ -134.22 (d, *J* = 21.5 Hz, o-*F*), -156.57 (t, *J* = 20.4 Hz, p-*F*), -163.07 (td, *J* = 24.3, 8.3 Hz, m-*F*).

^{11}B NMR (128 MHz, Chloroform-*d*) δ -9.17.

HRMS (ESI) m/z [M+Na$^+$] calc. for $C_{33}H_{21}BF_{15}NNa^+$: 750.1420; found: 750.1419.

5.3.8 (*E*)-3-(4-(tert-butyl)phenyl)pent-3-en-2-imine tris(perfluorophenyl)borane complex 16'

(*E*)-3-Phenylpent-3-en-2-imine tris(perfluorophenyl)borane complex **16'** was synthesized according to general procedure (chapter 5.2). It was purified by flash column chromatography (15 g silica n-hexane/EtOAc, 9:1).

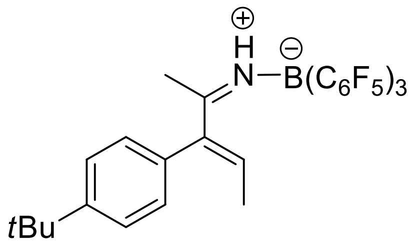

^1H NMR (400 MHz, Chloroform-*d*) δ 8.88 (m, 1H, N*H*), 7.54 (d, *J* = 8.3 Hz, 2H, Ar-*H*), 7.09 – 7.01 (m, 3H, Ar-*H*, $C_{sp2}H$), 2.25 (s, 3H, CH_3), 1.86 (d, *J* = 7.0 Hz, 3H, CH_3), 1.34 (s, 9H, $C(CH_3)_3$).

$^{13}C\{^1H\}$ NMR (101 MHz, Chloroform-*d*) δ 181.7 (*C*=N), 153.5 (Ar-C_q), 144.8 ($C_{sp2}H$), 140.9 ($C_{q,sp2}$), 129.8 (Ar-*C*), 127.5 (Ar-C_q), 127.1 (Ar-*C*), 35.0 ($C(CH_3)_3$), 31.2 ($C(CH_3)_3$), 17.6 (CH_3), 17.4 (CH_3).

Remark: The $^{13}C\{^1H\}$ NMR spectrum shows broad signals with low intensity at 149.1, 146.7, 141.2, 138.5, 135.9 ppm which can be tentatively assigned to the pentafluorophenyl groups.

^{19}F NMR (377 MHz, Chloroform-*d*) δ -134.29 (d, *J* = 20.6 Hz, o-*F*), -157.21 (t, *J* = 20.4 Hz, p-*F*), -163.50 (td, *J* = 23.2, 7.6 Hz, m-*F*).

^{11}B NMR (128 MHz, Chloroform-*d*) δ -9.51.

HRMS (ESI) m/z [M+Na$^+$] calc. for $C_{33}H_{21}BF_{15}NNa^+$: 750.1420; found: 750.1419.

5.3.9 3-Cyclohexylpent-4-en-2-imine tris(perfluorophenyl)borane complex 17

3-Cyclohexylpent-4-en-2-imine tris(perfluorophenyl)borane complex **17'** was synthesized according to general procedure (chapter 5.1) with cyclohexylallene (0.36 mmol, 51.7 µL) and a reaction time of 7 d.

The crude product was purified by flash column chromatography (13 g silica, *n*-hexane/EtOAc, 14:1). The product was obtained as single isomer in 76% yield (155.0 mg).

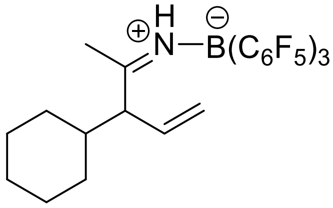

^{1}H NMR (400 MHz, Chloroform-*d*) δ 9.40 (s, 1H, N*H*), 5.65 (ddd, *J* = 16.8, 10.0, 8.9 Hz, 1H, C_{sp2}*H*), 5.52 (d, *J* = 10.0 Hz, 1H, C_{sp2}*H*), 5.36 (d, *J* = 16.8 Hz, 1H, C_{sp2}*H*), 2.93 (t, *J* = 8.4 Hz, 1H, C_{sp3}*H*-C=N), 2.05 (s, 3H, C*H*$_3$), 1.71 (dq, *J* = 19.9, 8.5, 6.8 Hz, 4H, Cy-*H*), 1.55 (d, *J* = 12.8 Hz, 1H, Cy-*H*), 1.38 (d, *J* = 12.7 Hz, 1H, Cy-*H*), 1.31 – 0.88 (m, 5H, Cy-*H*).

^{13}C{^{1}H} NMR (101 MHz, Chloroform-*d*) δ 194.8 (*C*=N), 130.8 (*C*$_{sp2}$H), 124.3 (*C*$_{sp2}$H), 63.4 (C_{sp3}C=N), 39.8 (Cy-*C*), 31.4 (Cy-*C*), 29.5 (Cy-*C*), 26.0 (Cy-*C*), 25.9 (Cy-*C*), 25.9 (Cy-*C*), 25.9 (Cy-*C*).

^{11}B NMR (128 MHz, Chloroform-*d*) δ -9.02.

^{19}F NMR (377 MHz, Chloroform-*d*) δ -133.87 (d, *J* = 22.3 Hz, o-*F*), -156.73 (t, *J* = 20.4 Hz, p-*F*), -163.18 (td, *J* = 24.6, 8.4 Hz, m-*F*).

HRMS (ESI) m/z [M+Na$^+$] calc. for $C_{29}H_{19}BF_{15}NNa^+$: 700.1263; found: 700.1260.

5.3.10 3-vinylnonan-2-imine tris(perfluorophenyl)borane complex 18

3-Vinylnonan-2-imine tris(perfluorophenyl)borane complex **18** was synthesized according to general procedure (chapter 5.1) with 1,2-nonadiene (59.0 µL, 0.36 mmol) and a reaction time of 5 d.

It was purified by flash column chromatography (15 g silica, *n*-hexane/EtOAc, 19:1). The product was isolated as a single isomer in 51% yield (103.8 mg)

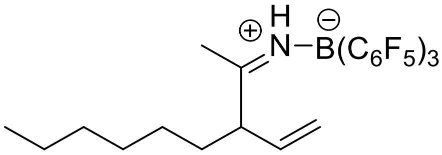

^{1}H NMR (400 MHz, Chloroform-*d*) δ 9.48 (s, 1H, N*H*), 5.65 (ddd, *J* = 16.9, 10.2, 7.5 Hz, 1H, C_{sp2}*H*), 5.57 (d, *J* = 9.6 Hz, 1H, C_{sp2}*H*), 5.40 (d, *J* = 16.8 Hz, 1H, C_{sp2}*H*), 3.16 (q, *J* = 7.9 Hz, 1H, C_{sp3}*H*-C=N), 2.05 (s, 4H, C*H*$_3$), 1.81 – 1.59 (m, 2H, C*H*$_2$), 1.36 – 1.21 (m, 8H, C*H*$_2$), 0.89 (t, *J* = 6.9 Hz, 3H, C*H*$_3$).

^{13}C{^{1}H} NMR (101 MHz, Chloroform-*d*) δ 194.4 (*C*=N), 132.3 (*C*$_{sp2}$H), 123.5 (*C*$_{sp2}$H), 55.4 (*C*$_{sp3}$H-C=N), 31.5 (*C*H$_2$), 30.6 (*C*H$_2$), 28.8 (*C*H$_2$), 26.8 (*C*H$_2$), 22.5 (*C*H$_2$), 20.7 (*C*H$_3$), 13.9 (*C*H$_3$).

Remark: The ^{13}C{^{1}H} NMR spectrum shows broad signals with low intensity at 149.1, 146.7, 141.3, 138.5, 136.0 ppm which can be tentatively assigned to the pentafluorophenyl groups.

^{11}B NMR (128 MHz, Chloroform-*d*) δ -9.12.

^{19}F NMR (377 MHz, Chloroform-*d*) δ -134.11 (d, *J* = 22.1 Hz, o-*F*), -156.48 (t, *J* = 20.4 Hz, p-*F*), -162.94 (td, *J* = 24.5, 8.5 Hz, m-*F*).

HRMS (ESI) m/z [M+Na$^+$] calc. for $C_{29}H_{21}BF_{15}NNa^+$: 750.1420; found: 702.1423.

5.3.11 (*E*)-3-ethylidenenonan-2-imine tris(perfluorophenyl)borane complex 18'

(*E*)-3-Ethylidenenonan-2-imine tris(perfluorophenyl)borane complex **18'** was synthesized according to general procedure (chapter 5.2) with 3-vinylnonan-2-imine tris(perfluorophenyl)borane complex (75.5 mg, 0.11 mmol) **18** and triethylamine (15.4 µL, 0.11 mmol). It was purified by flash column chromatography (15 g silica, *n*-hexane/EtOAc, 9:1). The product was isolated in 95% yield (72 mg).

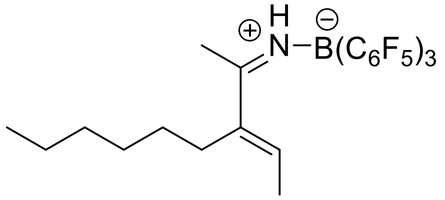

^{1}H NMR (400 MHz, Chloroform-*d*) δ 9.19 (s, 1H, N*H*), 6.68 (q, *J* = 7.0 Hz, 1H, $C_{sp2}H$), 2.41 – 2.33 (m, 2H, CH_2-$C_{sp2,q}$), 2.15 (s, 3H, CH_3-C=N), 1.98 (d, *J* = 7.0 Hz, 3H, CH_3-C_{sp2}H), 1.42 – 1.20 (m, 8H, CH_2-groups), 0.91 – 0.84 (t, *J* = 6.9 Hz, 3H).

^{13}C{^{1}H} NMR (101 MHz, Chloroform-*d*) δ 184.2 (*C*=N), 142.0 (C_{sp2}H), 139.1 ($C_{sp2,q}$), 31.4 (CH_2), 29.3 (CH_2), 28.0 (CH_2), 25.8 (CH_2-$C_{sp2,q}$), 22.6 (CH_2), 18.8 (CH_3), 15.6 (CH_3), 14.0 (CH_3)

Remark: The ^{13}C{^{1}H} NMR spectrum shows broad signals with low intensity at 149.2, 146.7, 141.4, 138.6, 136.0 ppm which can be tentatively assigned to the pentafluorophenyl groups.

^{11}B NMR (128 MHz, Chloroform-*d*) δ -9.23.

^{19}F NMR (377 MHz, Chloroform-*d*) δ -134.17 (d, *J* = 21.8 Hz, o-*F*), -156.87 (t, *J* = 20.4 Hz, p-*F*), -163.15 (td, *J* = 23.9, 8.0 Hz, m-*F*).

HRMS (ESI) m/z [M+Na$^+$] calc. for $C_{29}H_{21}BF_{15}NNa^+$: 750.1420; found: 702.1418.

5.3.12 3-(1-Adamantyl)pent-4-en-2-imine tris(perfluorophenyl)borane complex 19

3-(1-Adamantyl)pent-4-en-2-imine tris(perfluorophenyl)borane complex **19** was synthesized according to general procedure (chapter 5.1.) with (1-adamantyl)allene (62.7 mg, 0.36 mmol) and a reaction time of 5 d.

It was purified by flash column chromatography (30 g silica, *n*-hexane/EtOAc, 39:1, R_f = 0.2). The product was isolated as a single isomer in 90% yield (196.8 mg).

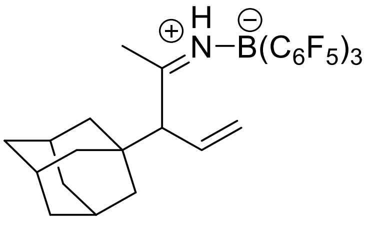

^{1}H NMR (400 MHz, Chloroform-*d*) δ ^{1}H NMR (400 MHz, Chloroform-*d*) δ 9.22 (s, 1H, N*H*), 5.85 (dt, *J* = 16.6, 10.0 Hz, 1H, $C_{sp2}H$), 5.42 (d, *J* = 10.5 Hz, 1H, $C_{sp2}H$), 5.24 (d, *J* = 16.6 Hz, 1H, $C_{sp2}H$), 2.72 (d, *J* = 9.9 Hz, $C_{sp3}H$-C=N), 2.06 (s, 3H, CH_3), 2.05-2.00 (m, 3H, Ad-C*H*), 1.77-1.69 (m, 3H, Ad-CH_2), 1.65-1.57 (m, 6H, Ad-CH_2), 1.50-1.43 (m, Ad-CH_2).

$^{13}C\{^{1}H\}$ NMR (101 MHz, Chloroform-*d*) δ 195.2 (*C*=N), 129.5 (C_{sp2}H), 123.0 (C_{sp2}H), 68.9 (C_{sp3}H-C=N), 40.2 (Ad-CH_2), 38.0 (Ad-C_q), 36.4 (Ad-CH_2), 28.5 (Ad-*C*H), 24.3 (CH_3).

Remark: The $^{13}C\{^{1}H\}$ NMR spectrum shows broad signals with low intensity at 149.1, 146.7, 141.5, 138.9, 138.5, 136.0 ppm which can be tentatively assigned to the pentafluorophenyl groups.

^{11}B NMR (128 MHz, Chloroform-*d*) δ -9.12.

^{19}F NMR (377 MHz, Chloroform-*d*) δ ^{19}F NMR (377 MHz, Chloroform-*d*) δ -133.44 (d, *J* = 21.8 Hz, *o*-F), -156.52 (t, *J* = 20.4 Hz, *p*-F), -162.99 (td, *J* = 24.1, 8.0 Hz, *m*-F).

HRMS (ESI) m/z [$M+H^+$] calc. for $C_{33}H_{23}BF_{15}^-$: 728.1611; found: 728.1319.

5.4 Additional NMR spectra of catalysis products

5.4.1 3-phenylpent-4-en-2-imine tris(perfluorophenyl)borane complex 13

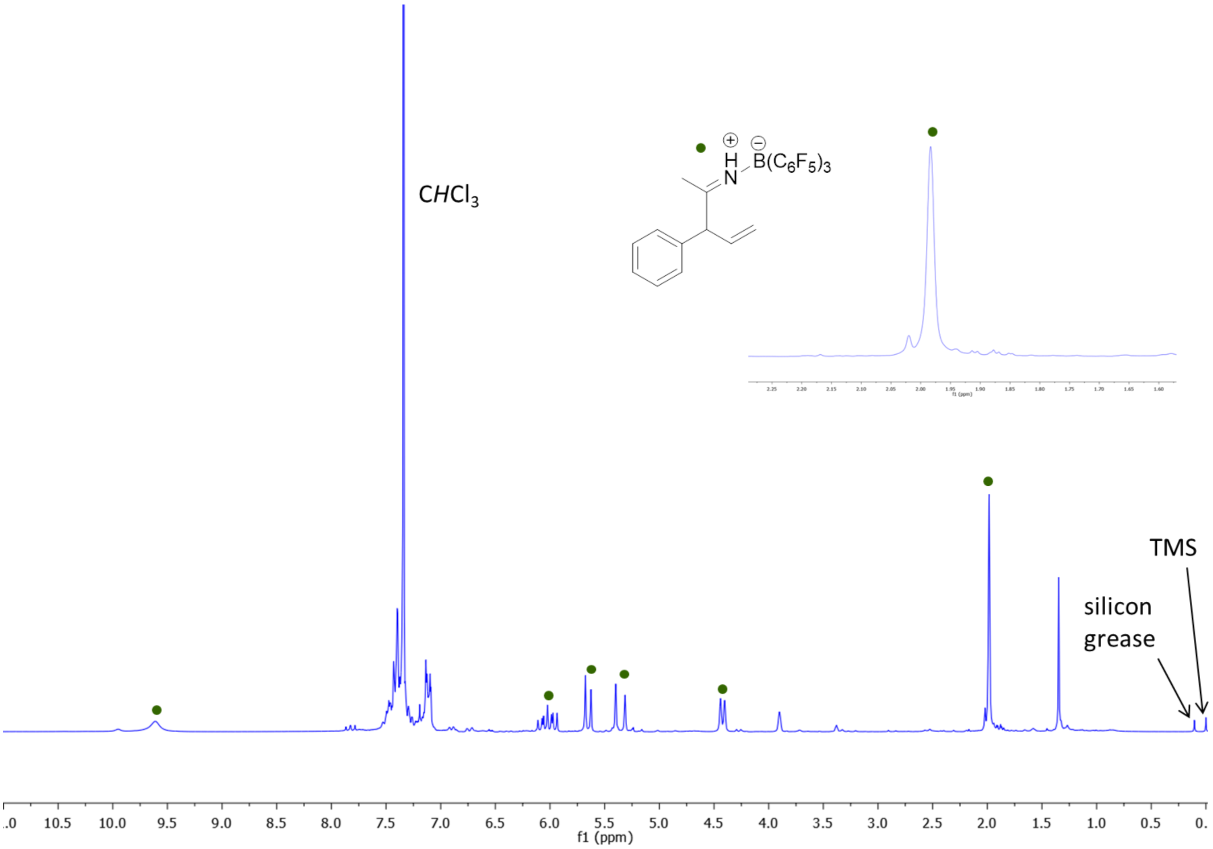

Figure SI 64: ^{1}H NMR of the crude reaction mixture with high field excerpt containing only **13** showing that the isomerization to **13’** is due to purification *via* column chromatography (200 MHz, Chloroform-*d*).

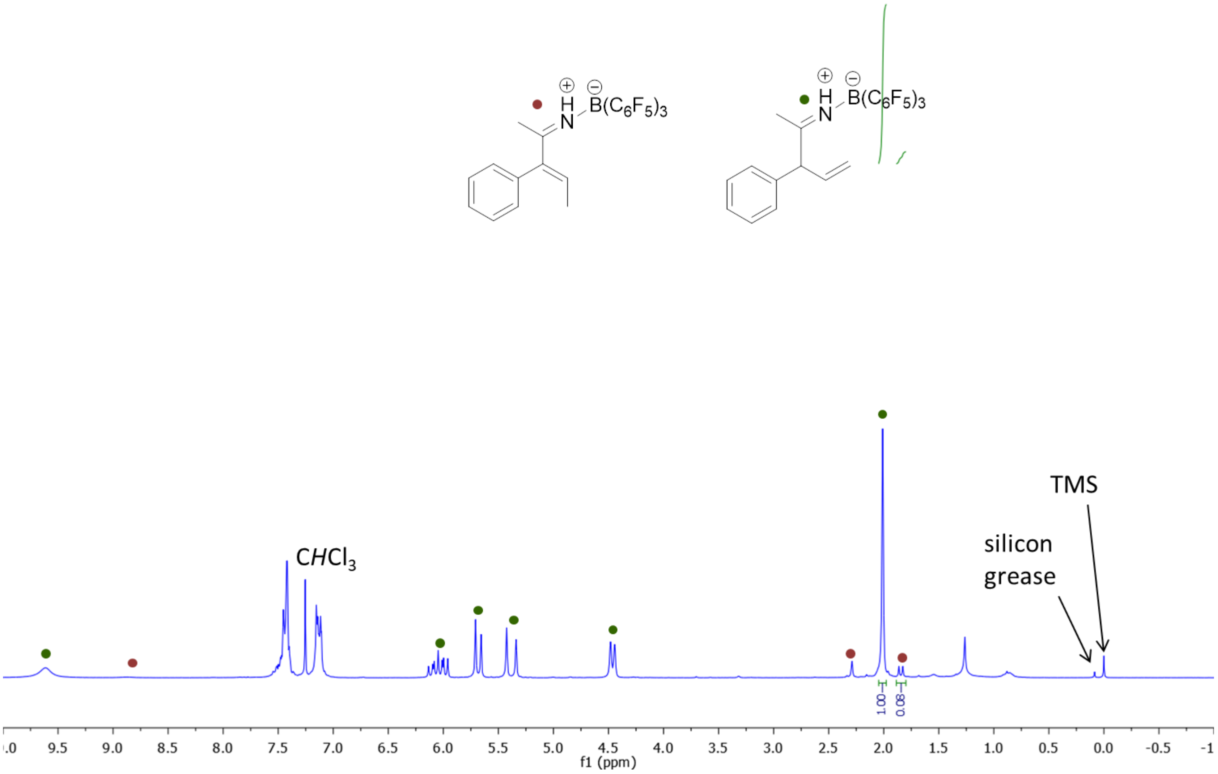

Figure SI 65: ^{1}H NMR of the product containing **13** and **13'** in a 93 to 7 ratio after purification of the crude reaction product via column chromatography (200 MHz, Chloroform-*d*).

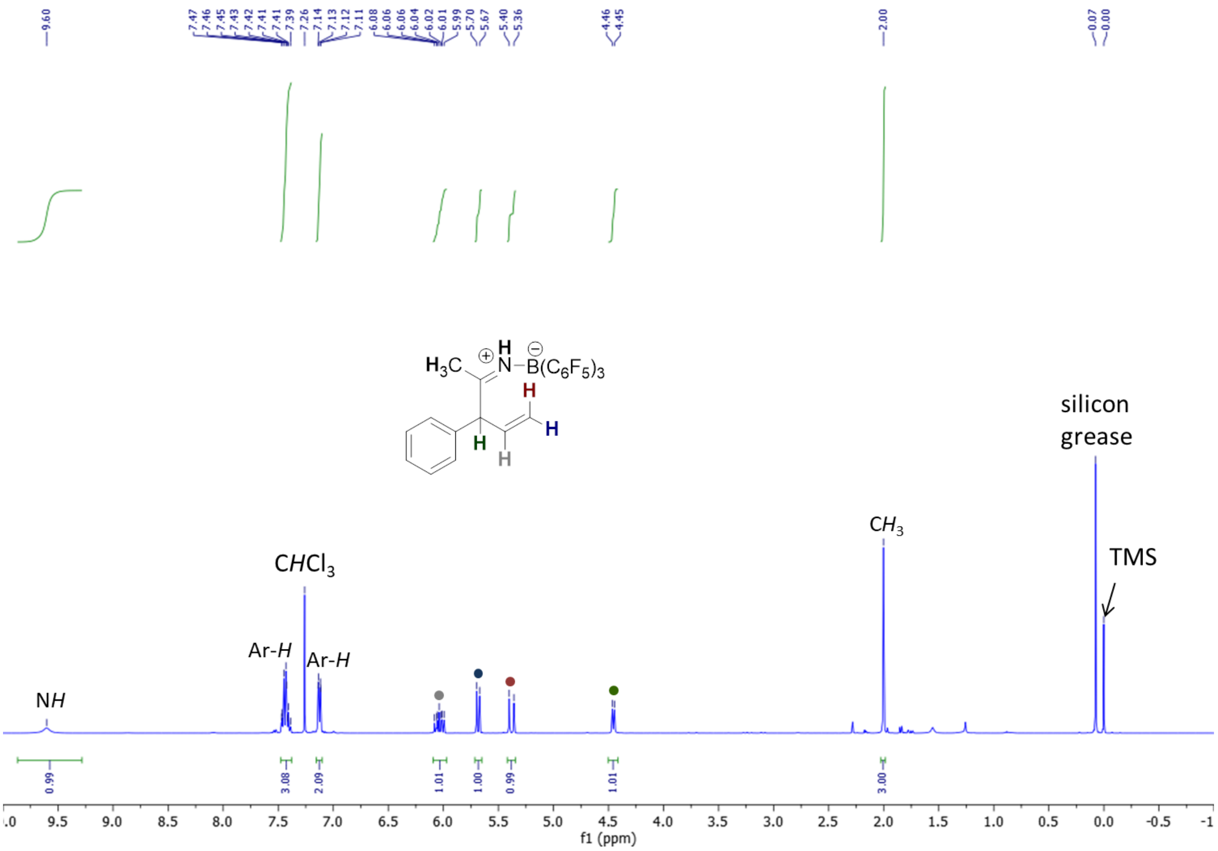

Figure SI 66: ^{1}H NMR spectrum of the analytical sample of the 3-phenylpent-4-en-2-imine tris(perfluorophenyl)borane complex **13** (400 MHz, Chloroform-*d*).

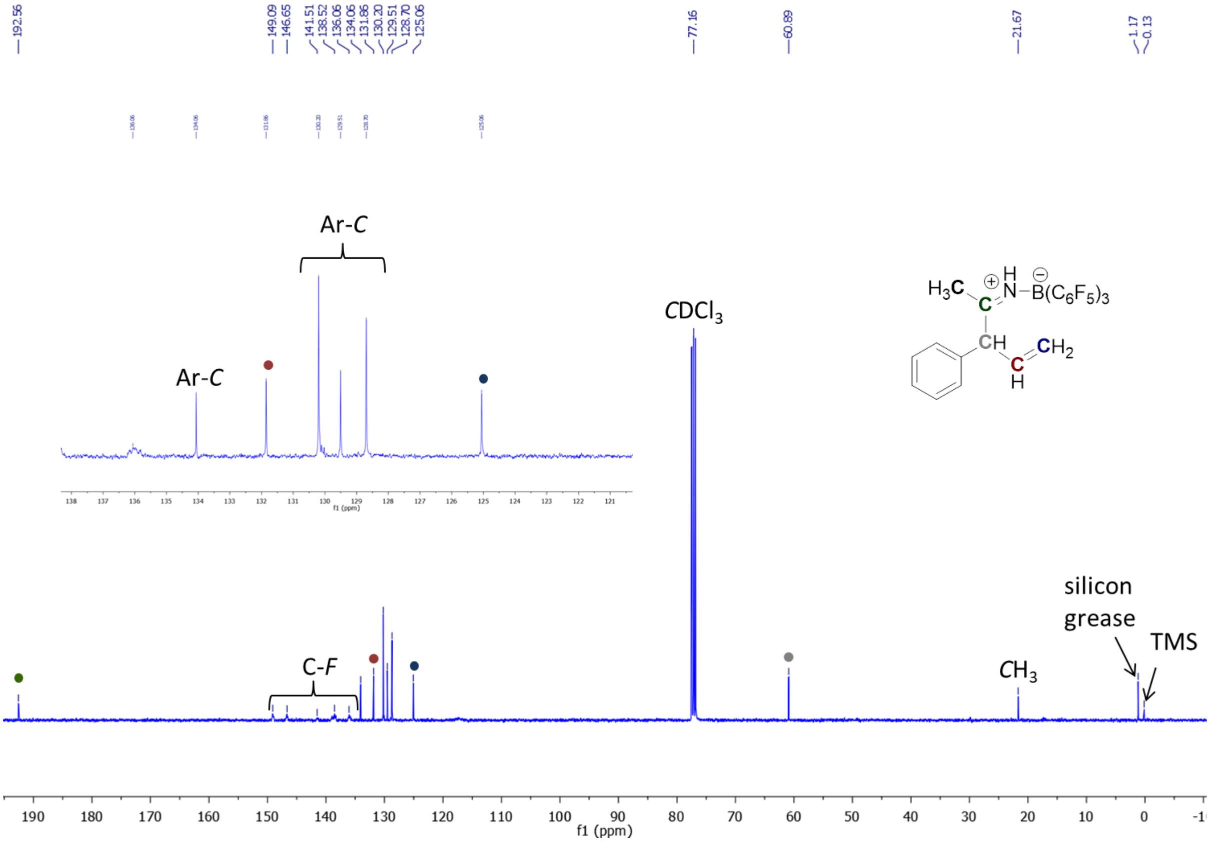

Figure SI 67: $^{13}C\{^1H\}$ NMR spectrum of the analytical sample of the 3-phenylpent-4-en-2-imine tris(perfluorophenyl)borane complex **13** (101 MHz, Chloroform-*d*).

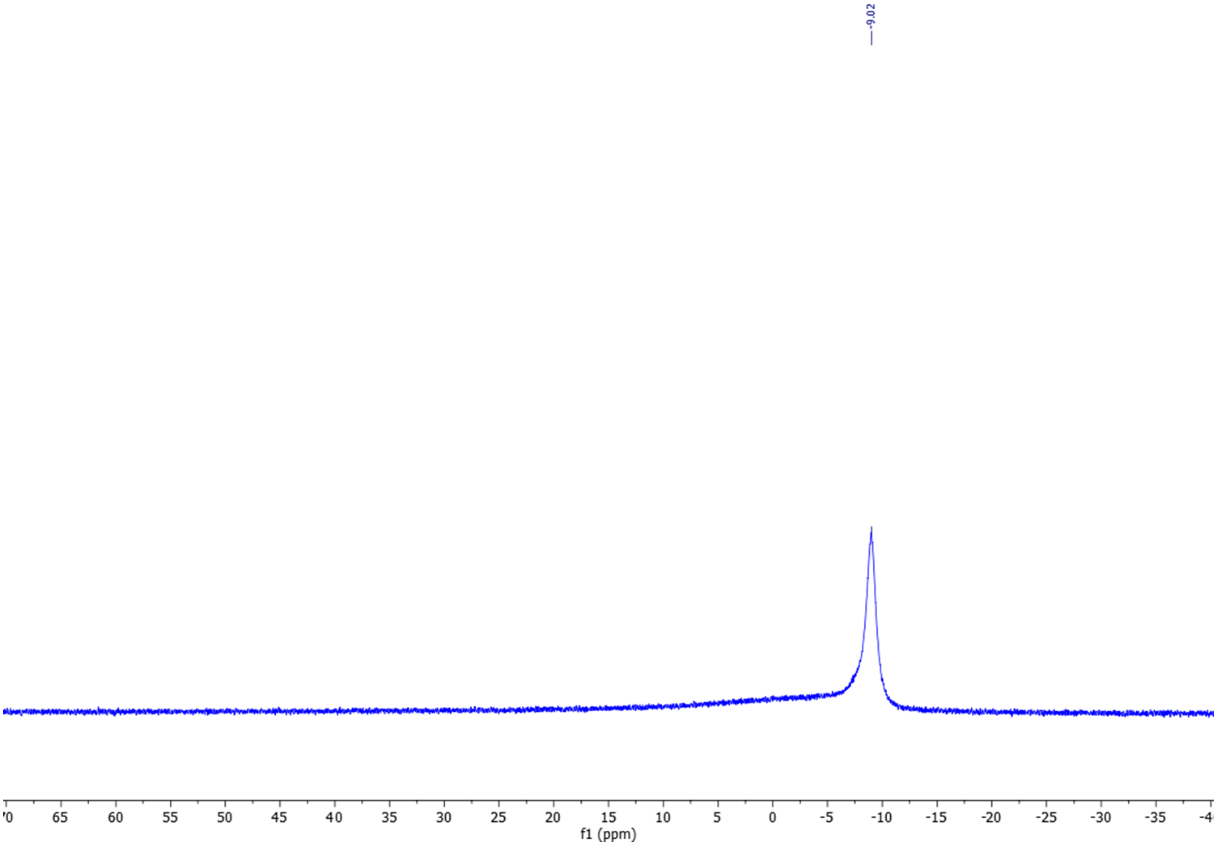

Figure SI 68: ^{11}B NMR spectrum of the analytical sample of the 3-phenylpent-4-en-2-imine tris(perfluorophenyl)borane complex **13** (128 MHz, Chloroform-*d*).

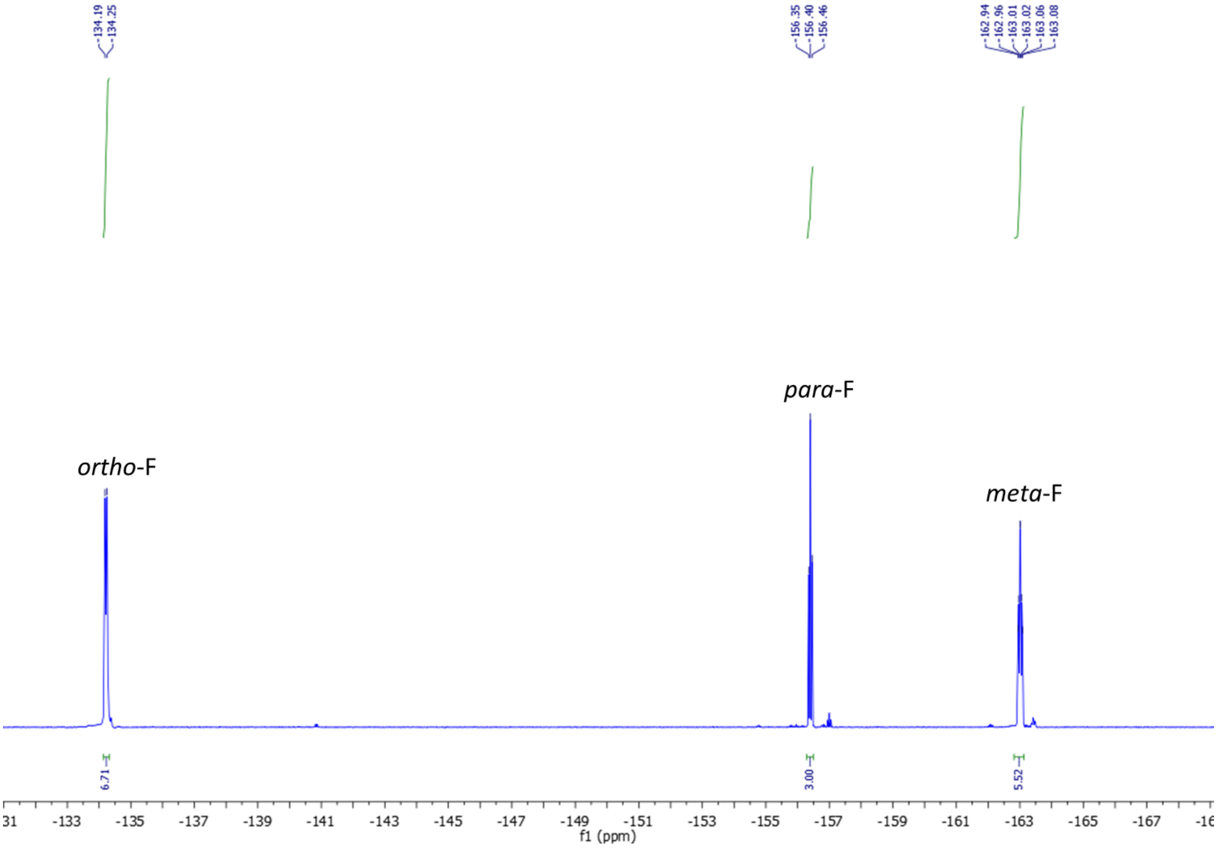

Figure SI 69: ^{19}F NMR spectrum of the analytical sample of the 3-phenylpent-4-en-2-imine tris(perfluorophenyl)borane complex **13** (377 MHz, Chloroform-*d*).

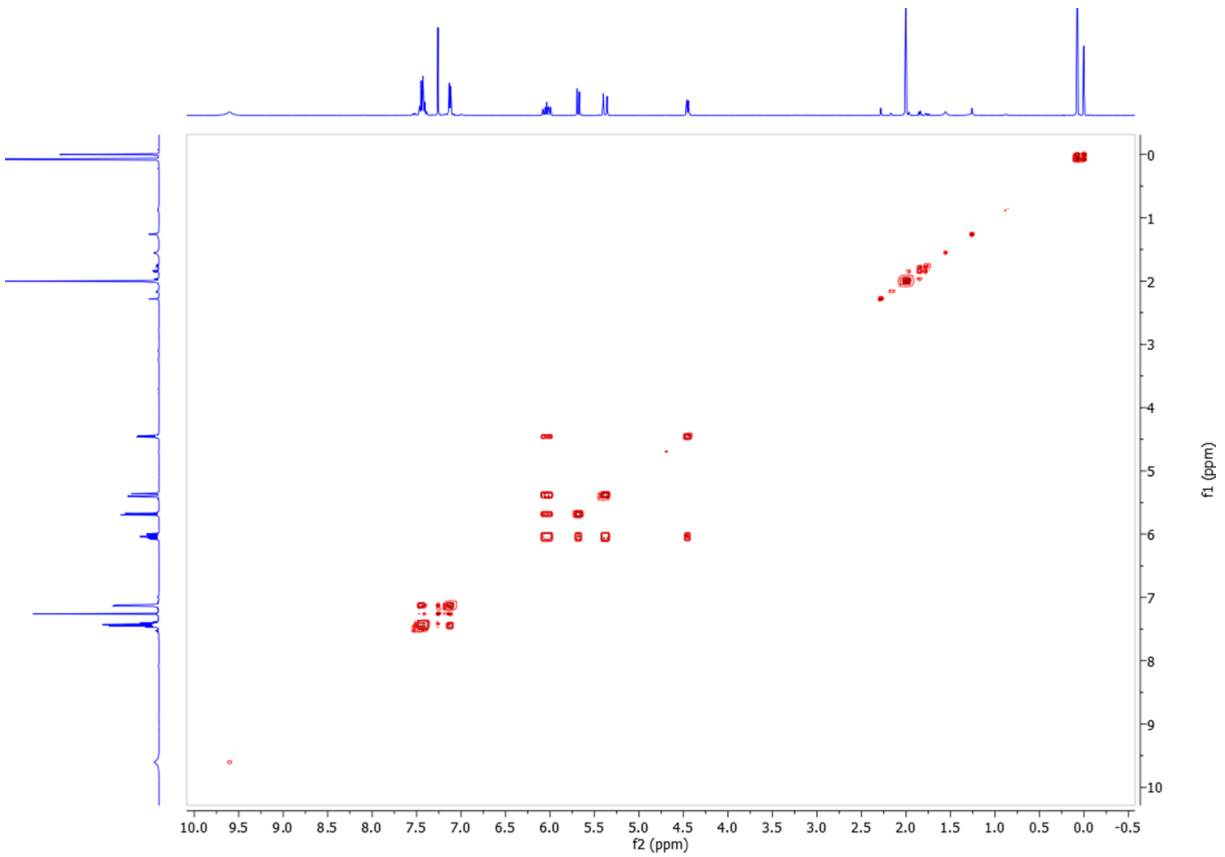

Figure SI 70: COSY NMR spectrum of the analytical sample of the 3-phenylpent-4-en-2-imine tris(perfluorophenyl)borane complex **13** (400 MHz, Chloroform-*d*).

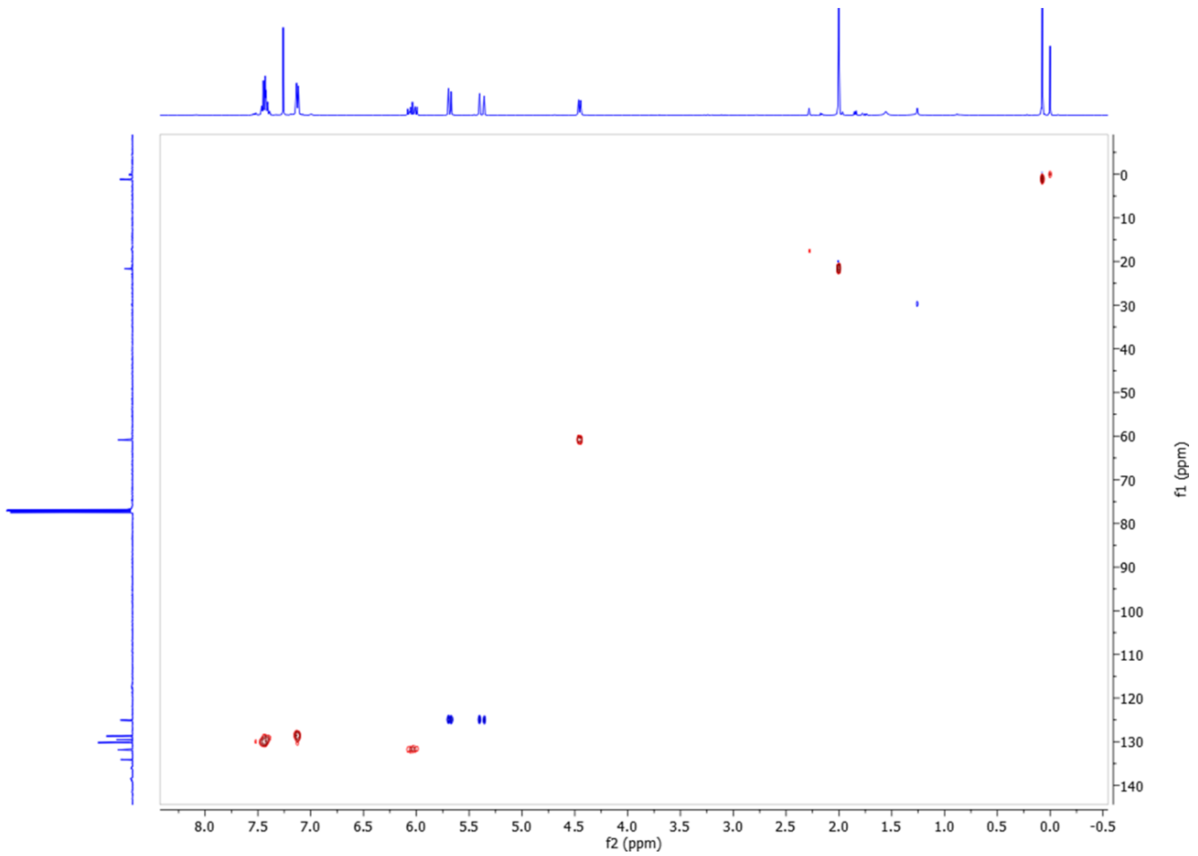

Figure SI 71: HSQC NMR spectrum of the analytical sample of the 3-phenylpent-4-en-2-imine tris(perfluorophenyl)borane complex **13** (101 MHz, Chloroform-*d*).

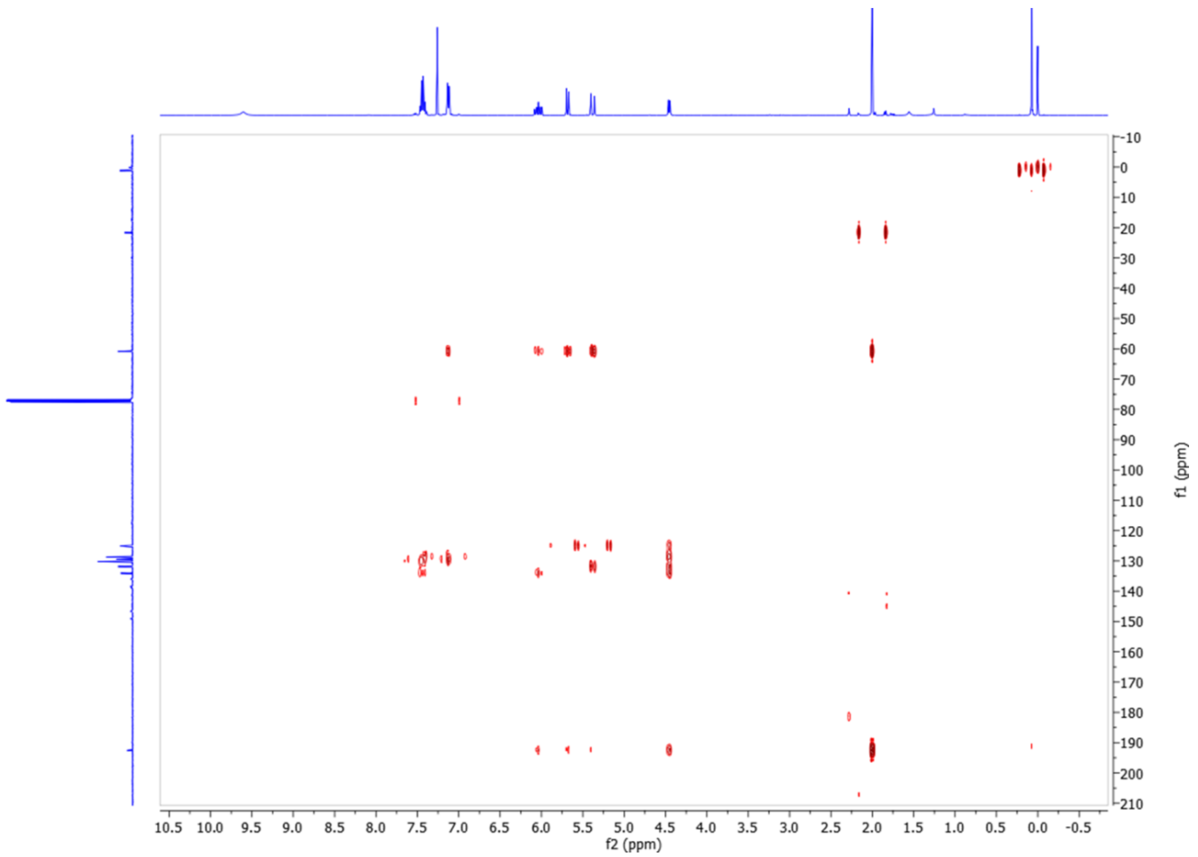

Figure SI 72: HMBC NMR spectrum of the analytical sample of the 3-phenylpent-4-en-2-imine tris(perfluorophenyl)borane complex **13** (101 MHz, Chloroform-*d*).

5.4.2 (*E*)-3-phenylpent-3-en-2-imine tris(perfluorophenyl)borane complex 13’

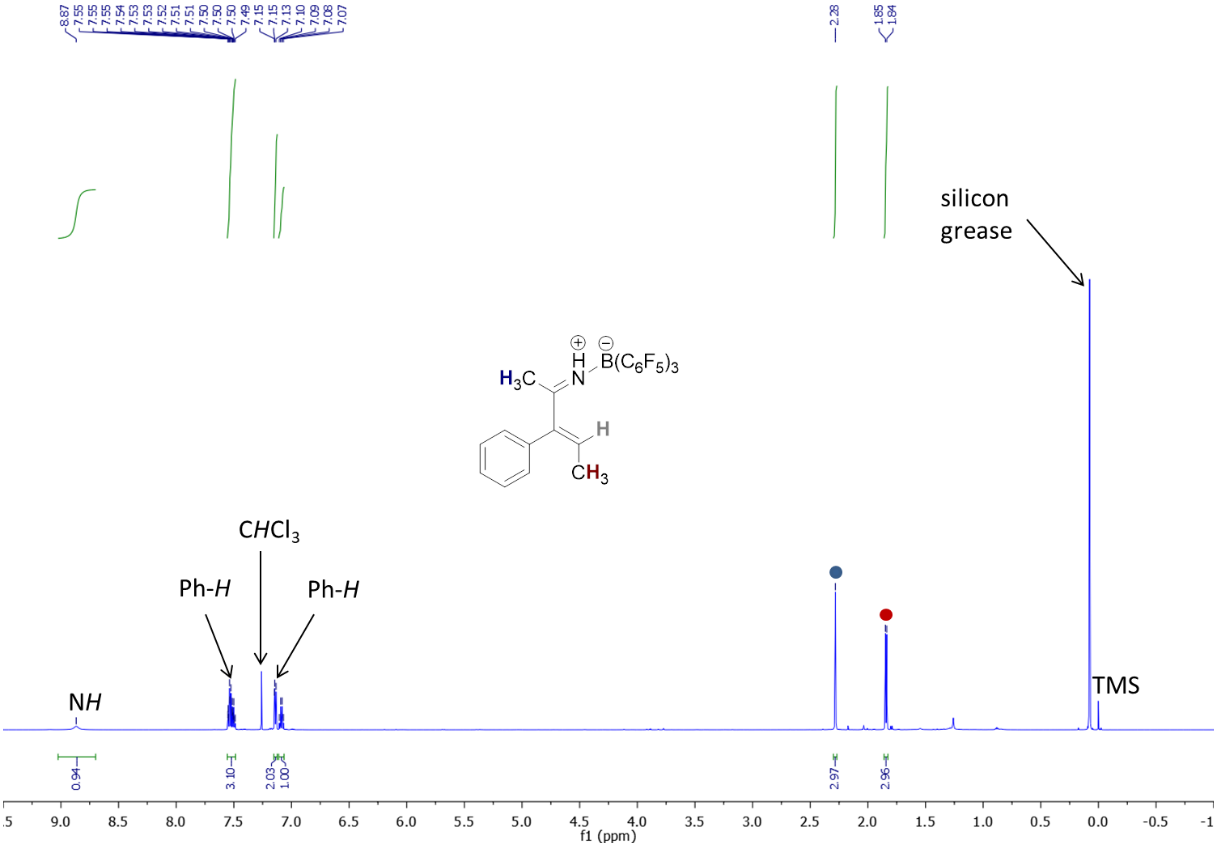

Figure SI 73: ^{1}H NMR spectrum of the analytical sample of the (*E*)-3-phenylpent-3-en-2-imine tris(perfluorophenyl)borane complex **13’** (400 MHz, Chloroform-*d*).

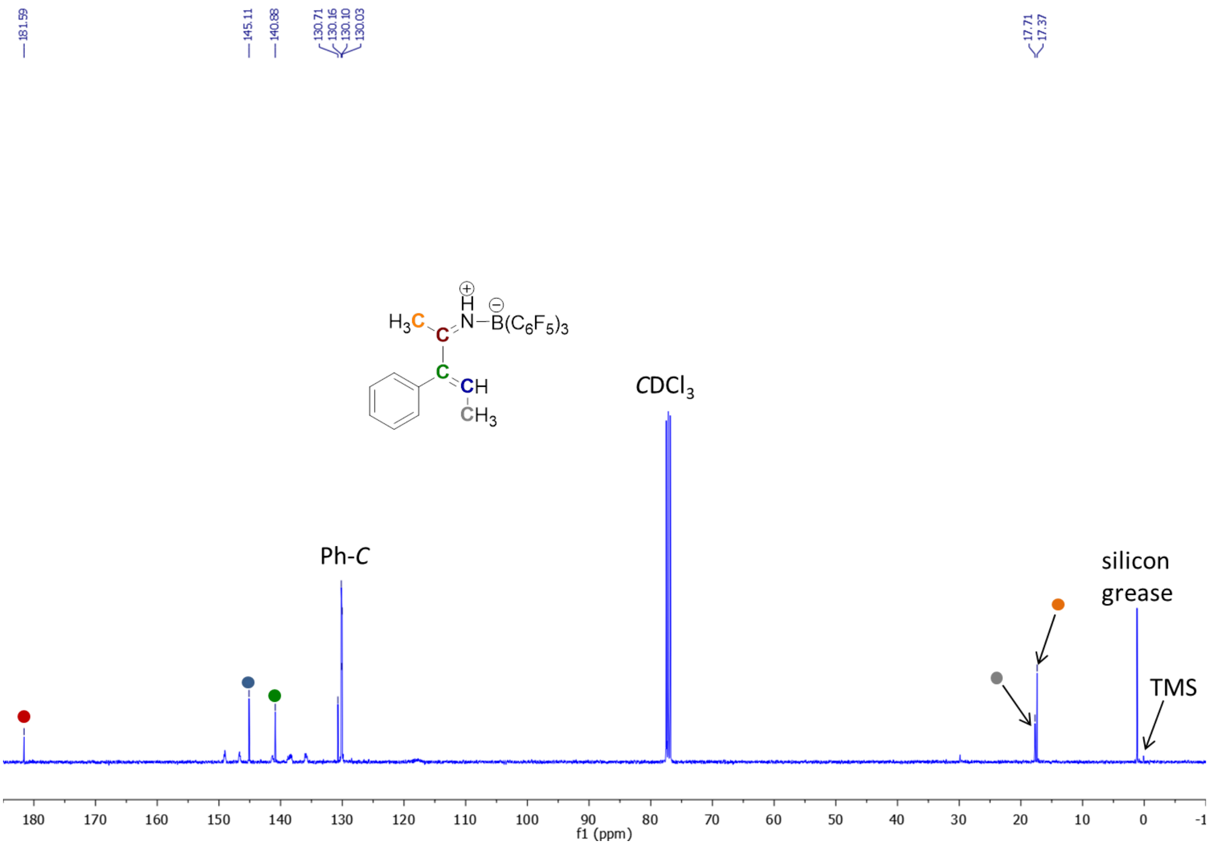

Figure SI 74: $^{13}C\{^{1}H\}$ NMR spectrum of the analytical sample of the (*E*)-3-phenylpent-3-en-2-imine tris(perfluorophenyl)borane complex **13’** (101 MHz, Chloroform-*d*).

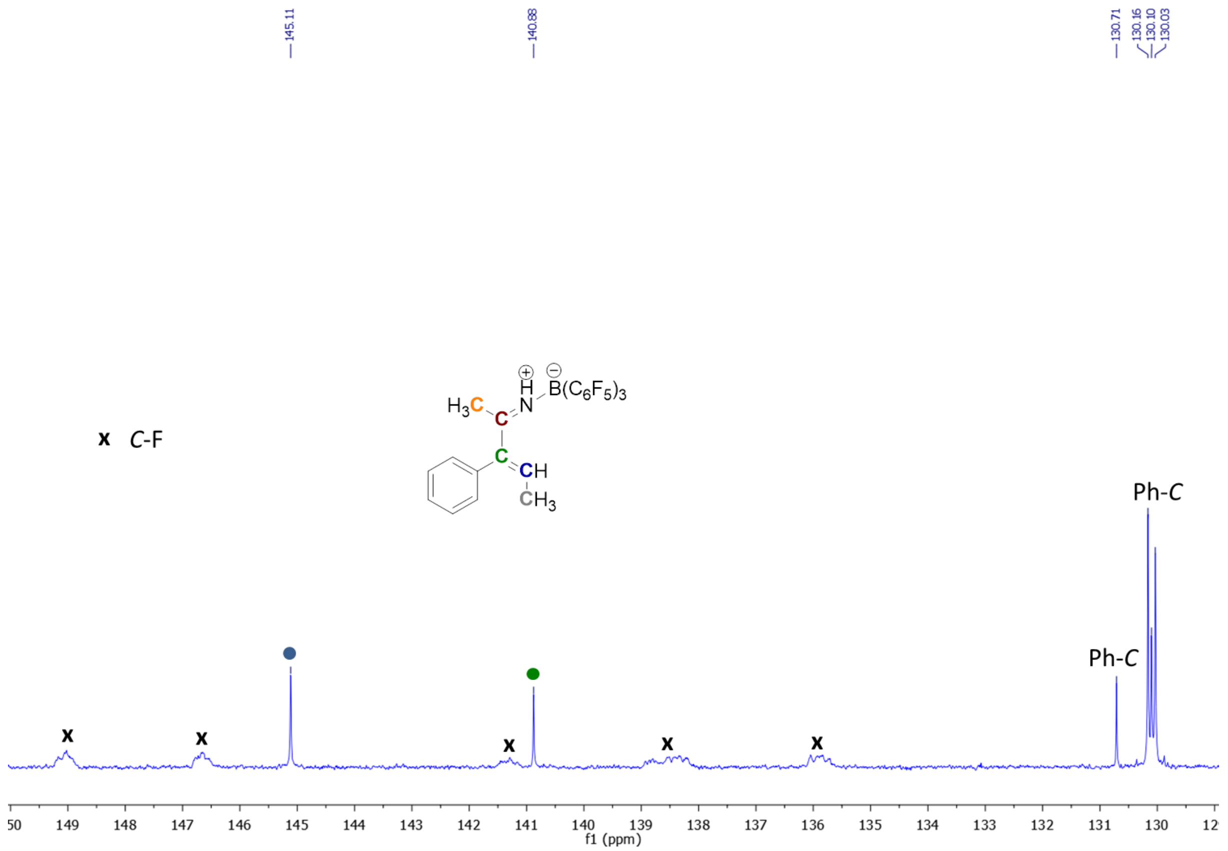

Figure SI 75: Low field excerpt of the $^{13}C\{^{1}H\}$ NMR spectrum of the analytical sample of the (*E*)-3-phenylpent-3-en-2-imine tris(perfluorophenyl)borane complex **13'** (101 MHz, Chloroform-*d*).

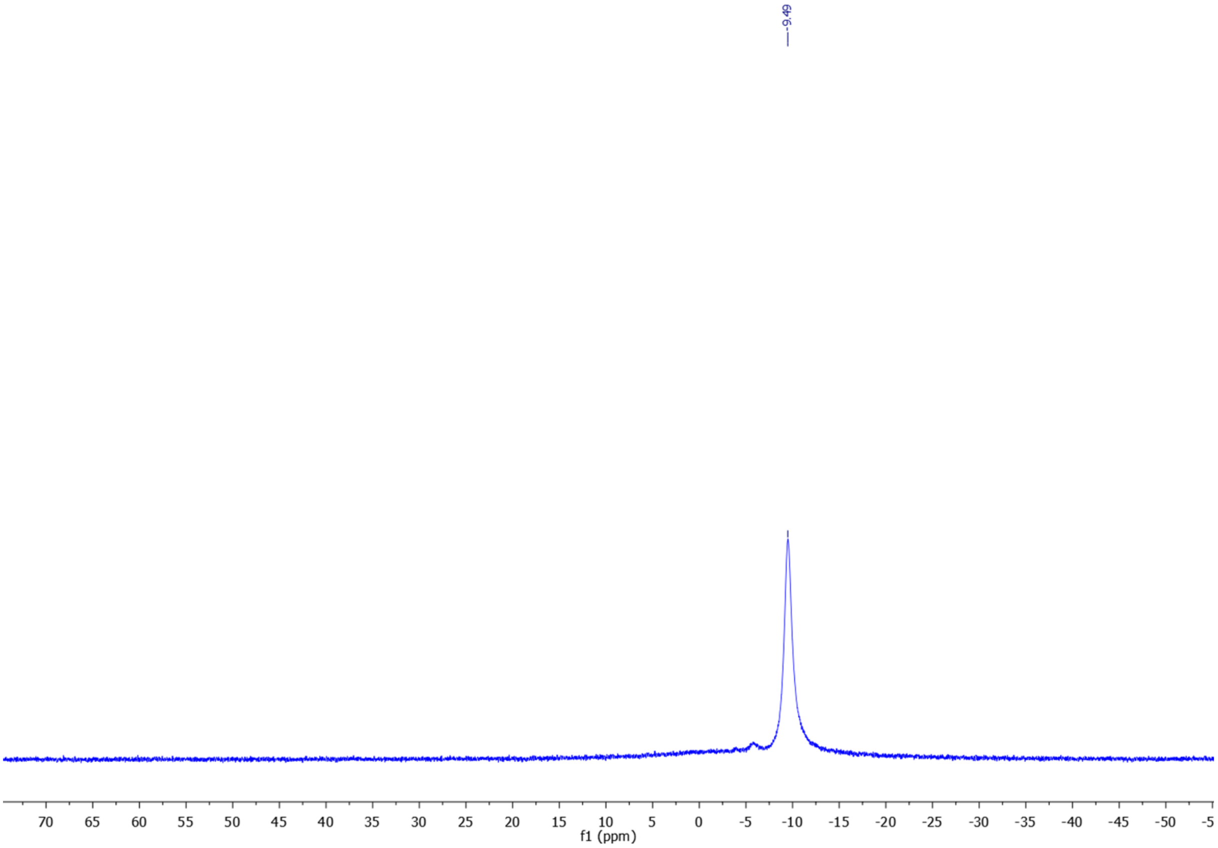

Figure SI 76: ^{11}B spectrum of the analytical sample of the (*E*)-3-phenylpent-3-en-2-imine tris(perfluorophenyl)borane complex **13'** (128 MHz, Chloroform-*d*).

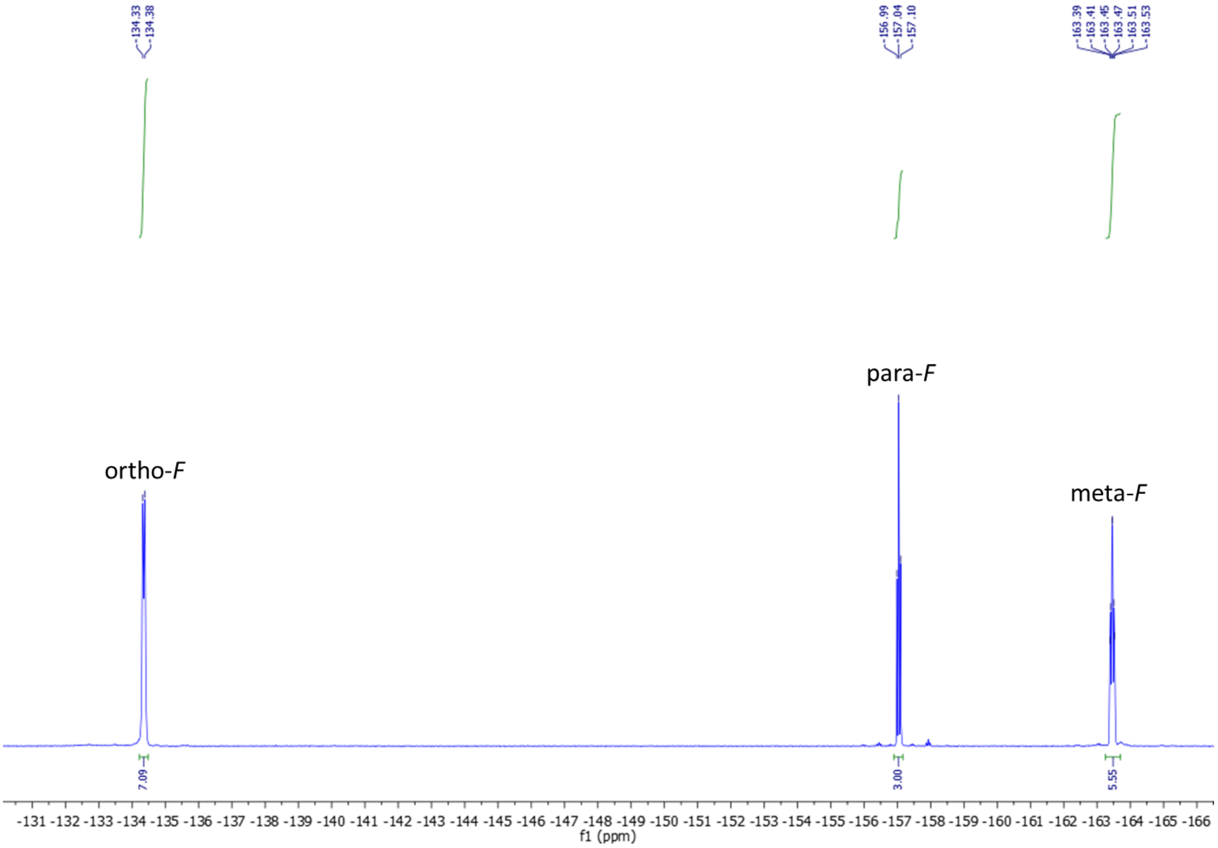

Figure SI 77: ^{19}F spectrum of the analytical sample of the (*E*)-3-phenylpent-3-en-2-imine tris(perfluorophenyl)borane complex **13'** (377 MHz, Chloroform-*d*).

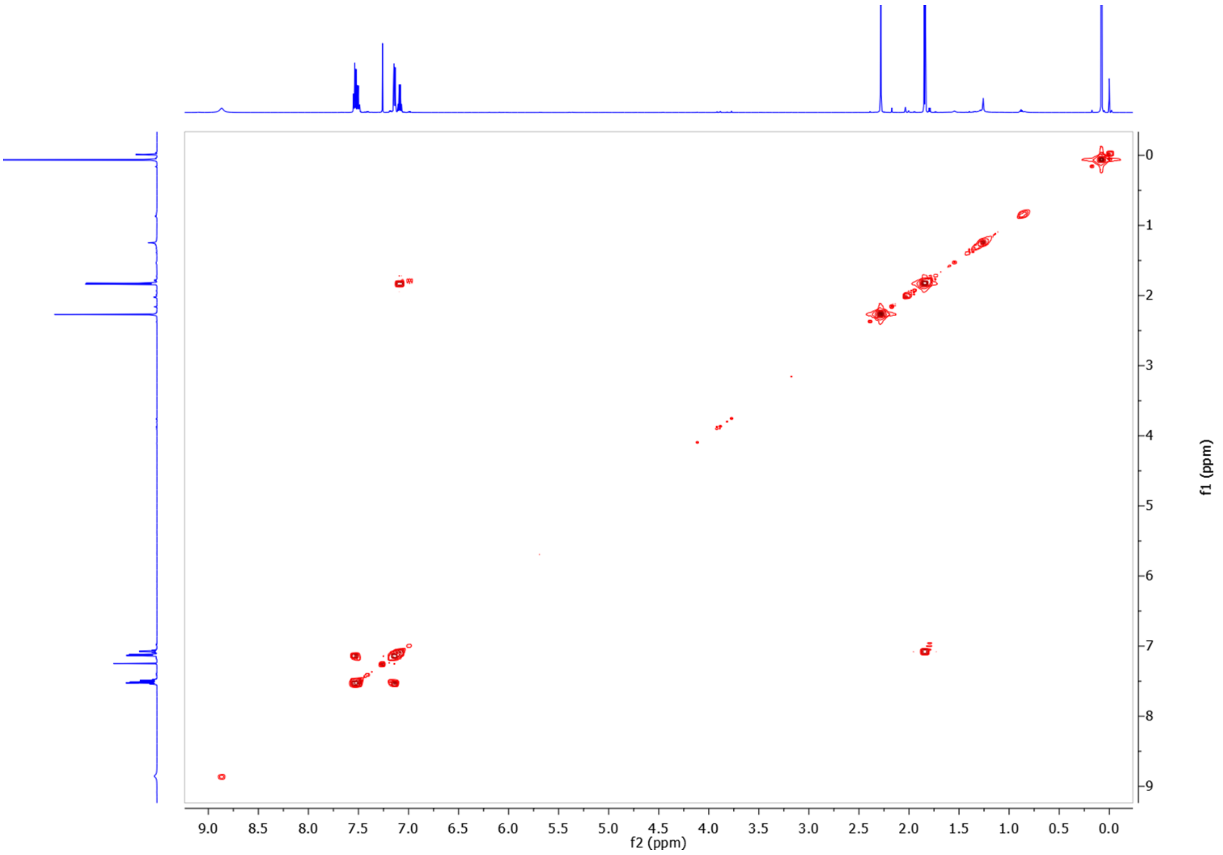

Figure SI 78: COSY spectrum of the analytical sample of the (*E*)-3-phenylpent-3-en-2-imine tris(perfluorophenyl)borane complex **13’** (400 MHz, Chloroform-*d*).

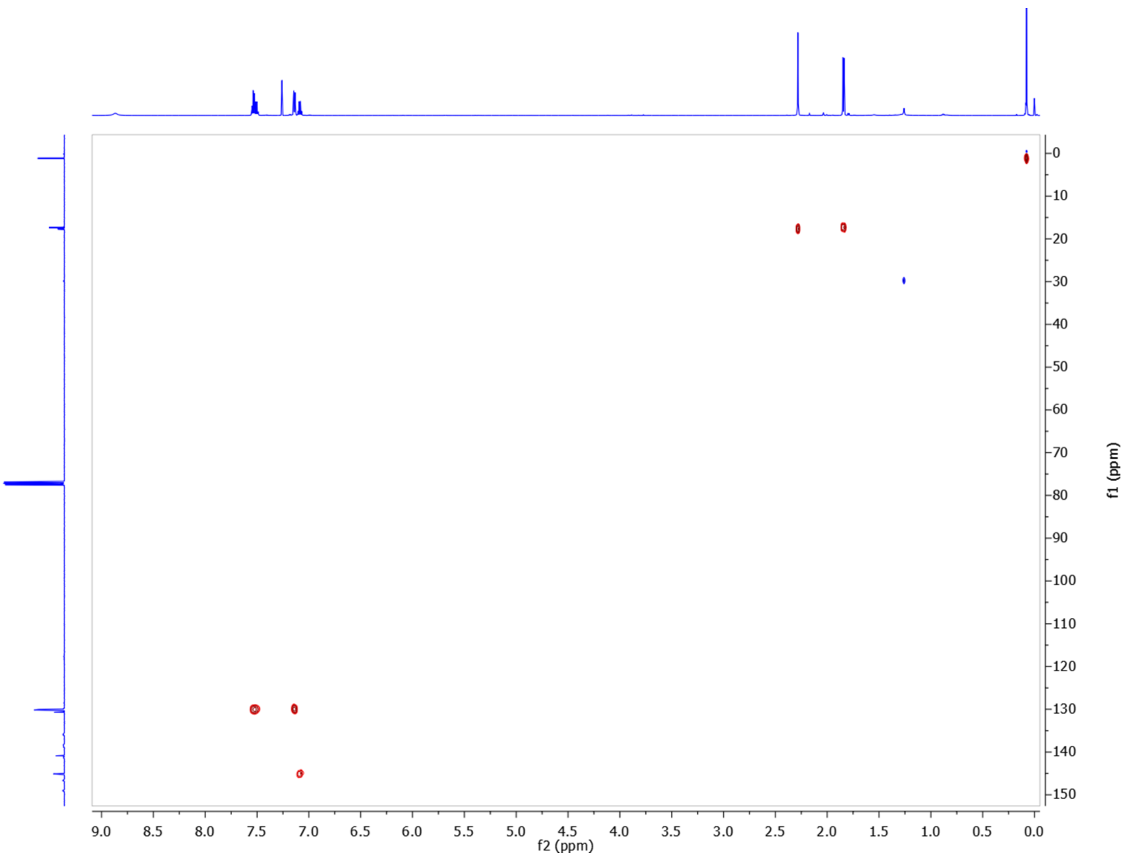

Figure SI 79: HSQC spectrum of the analytical sample of the (*E*)-3-phenylpent-3-en-2-imine tris(perfluorophenyl)borane complex **13'** (101 MHz, Chloroform-*d*).

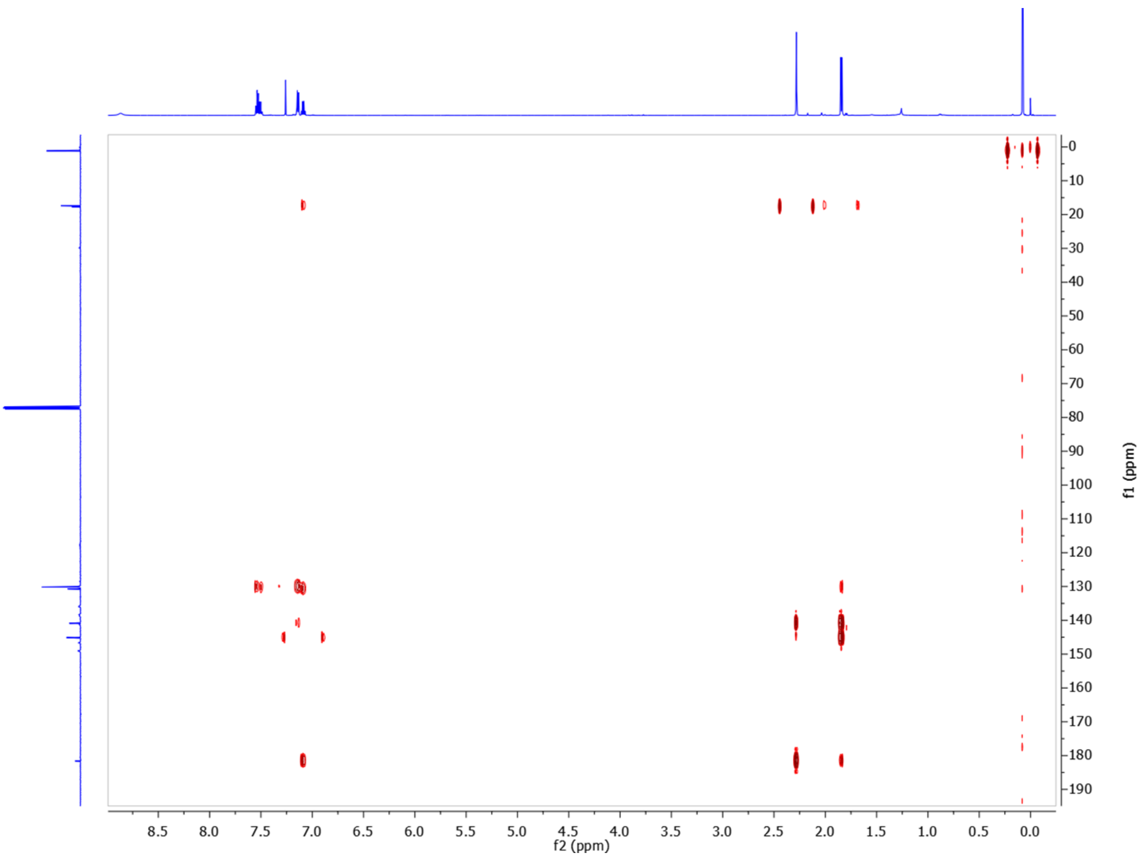

Figure SI 80: HMBC spectrum of the analytical sample of the (*E*)-3-phenylpent-3-en-2-imine tris(perfluorophenyl)borane complex **13’** (101 MHz, Chloroform-*d*).

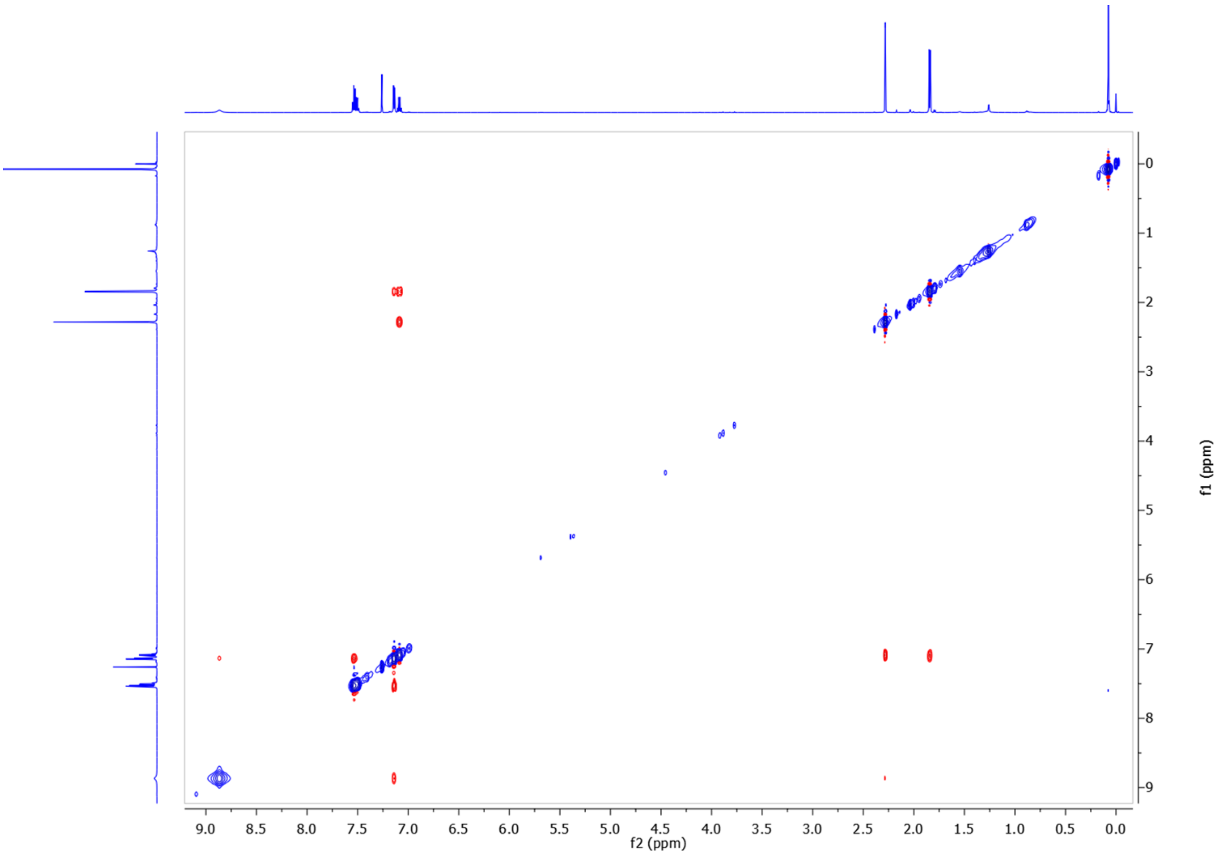

Figure SI 81: NOESY spectrum of the analytical sample of the (*E*)-3-phenylpent-3-en-2-imine tris(perfluorophenyl)borane complex **13'** (400 MHz, Chloroform-*d*).

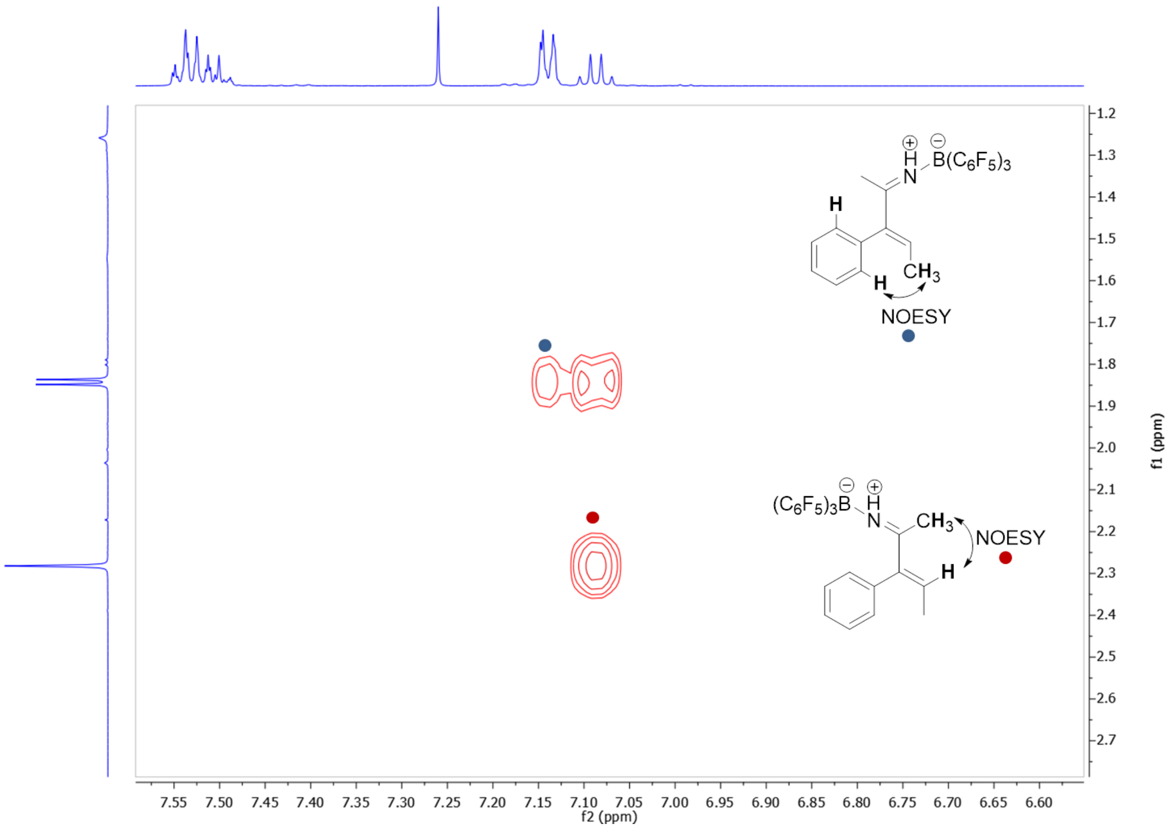

Figure SI 82: Excerpt of the NOESY spectrum of the analytical sample of the (*E*)-3-phenylpent-3-en-2-imine tris(perfluorophenyl)borane complex **13’** (400 MHz, Chloroform-*d*).

5.4.3 3-(4-(chloro)phenyl)pent-4-en-2-imine tris(perfluorophenyl)borane complex 14

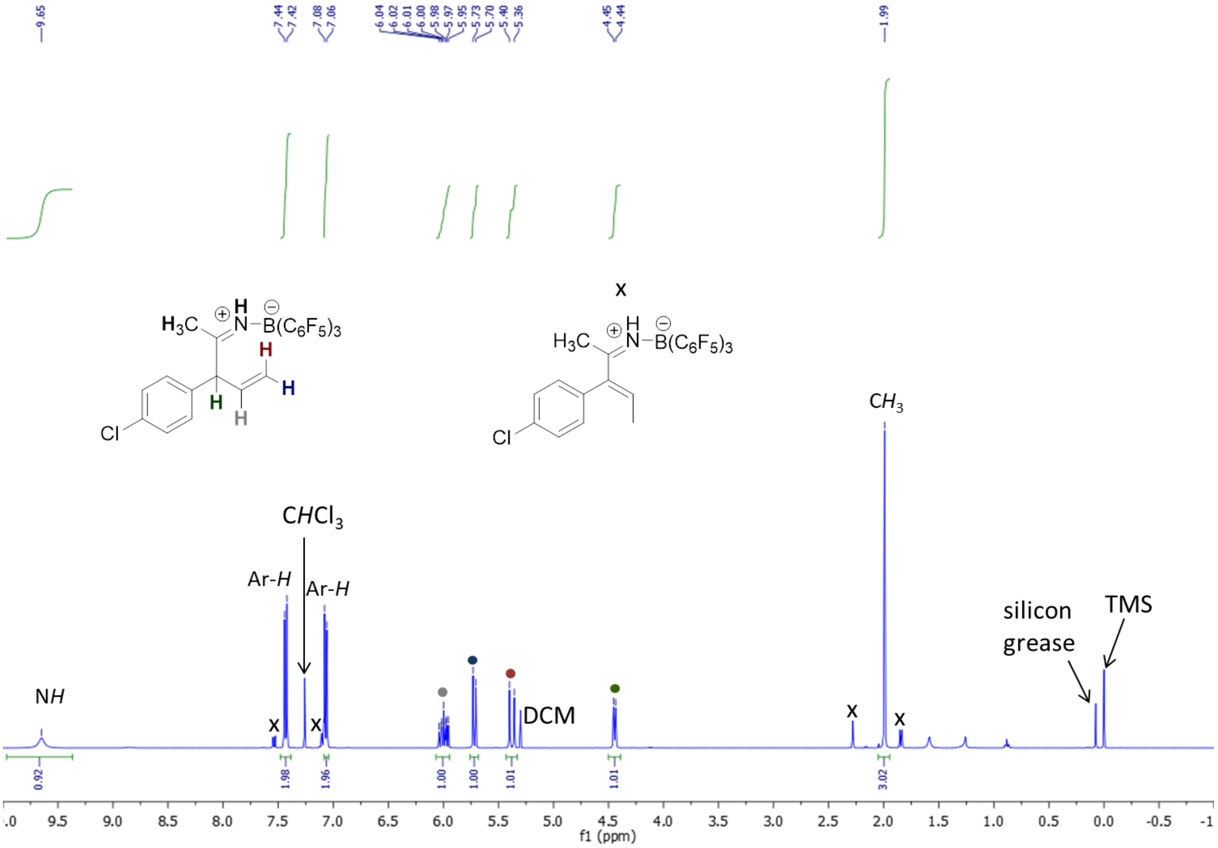

Figure SI 83: ^{1}H NMR of the reaction mixture containing **14** and **14'** in a 92 to 8 ratio after purification of the crude reaction product via column chromatography (400 MHz, Chloroform-*d*).

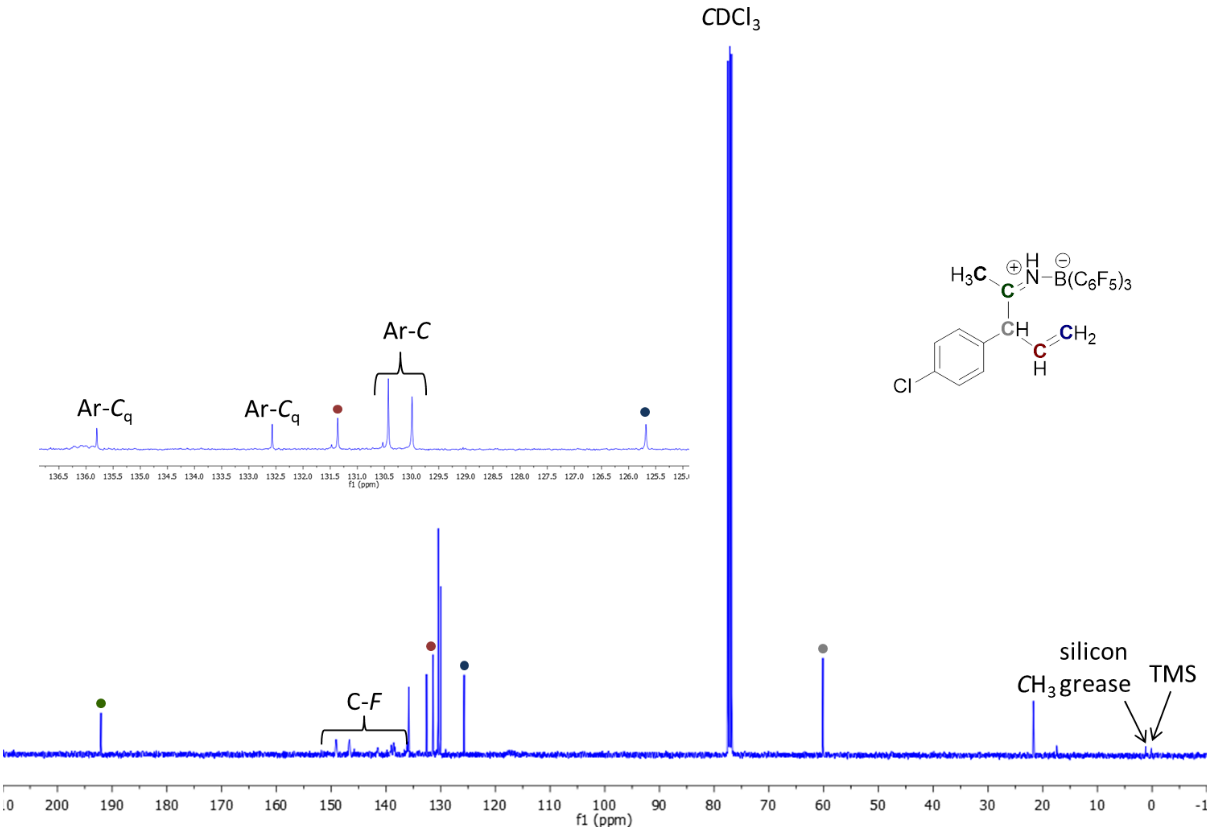

Figure SI 84: $^{13}C\{^{1}H\}$ NMR spectrum of the analytical sample of 3-(4-chlorophenyl)pent-4-en-2-imine tris(perfluorophenyl)borane complex **14** (101 MHz, Chloroform-*d*).

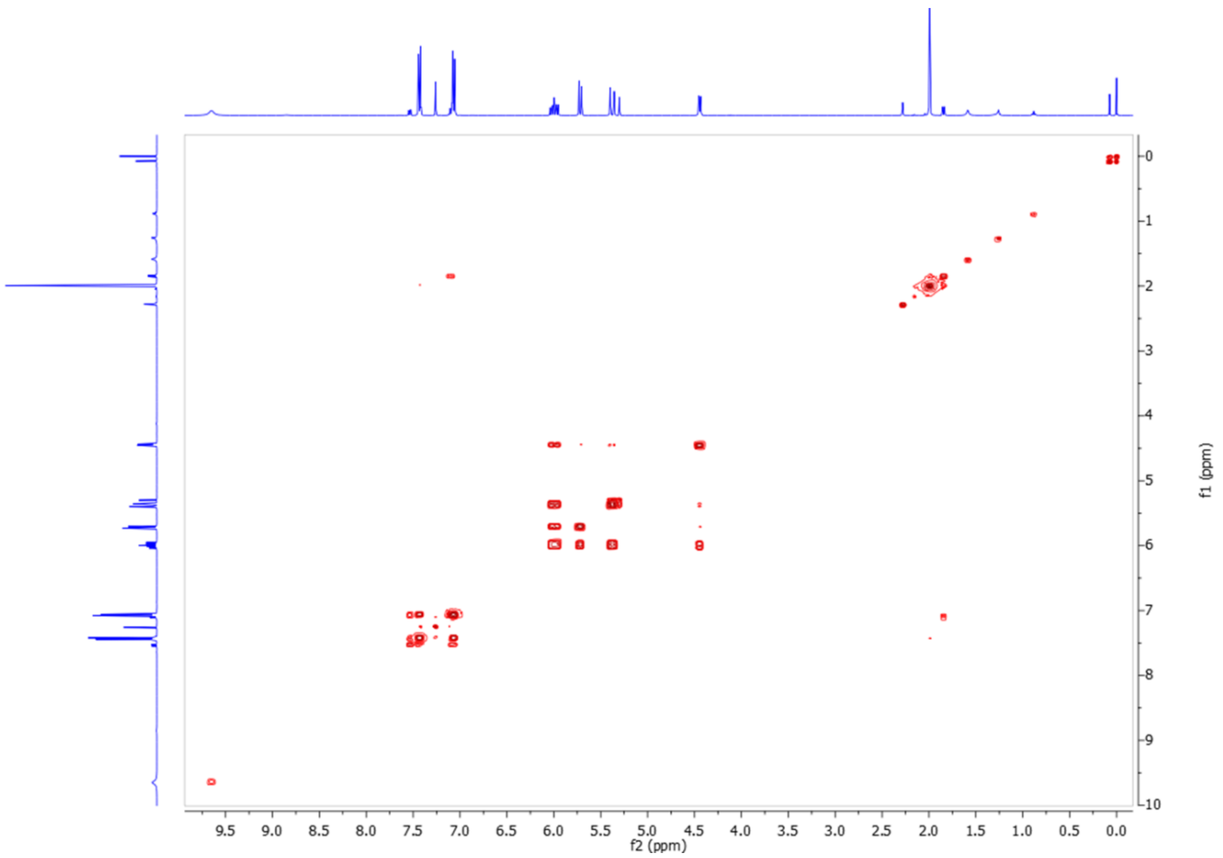

Figure SI 85: COSY NMR spectrum of the analytical sample of 3-(4-chlorophenyl)pent-4-en-2-imine tris(perfluorophenyl)borane complex **14** (400 MHz, Chloroform-*d*).

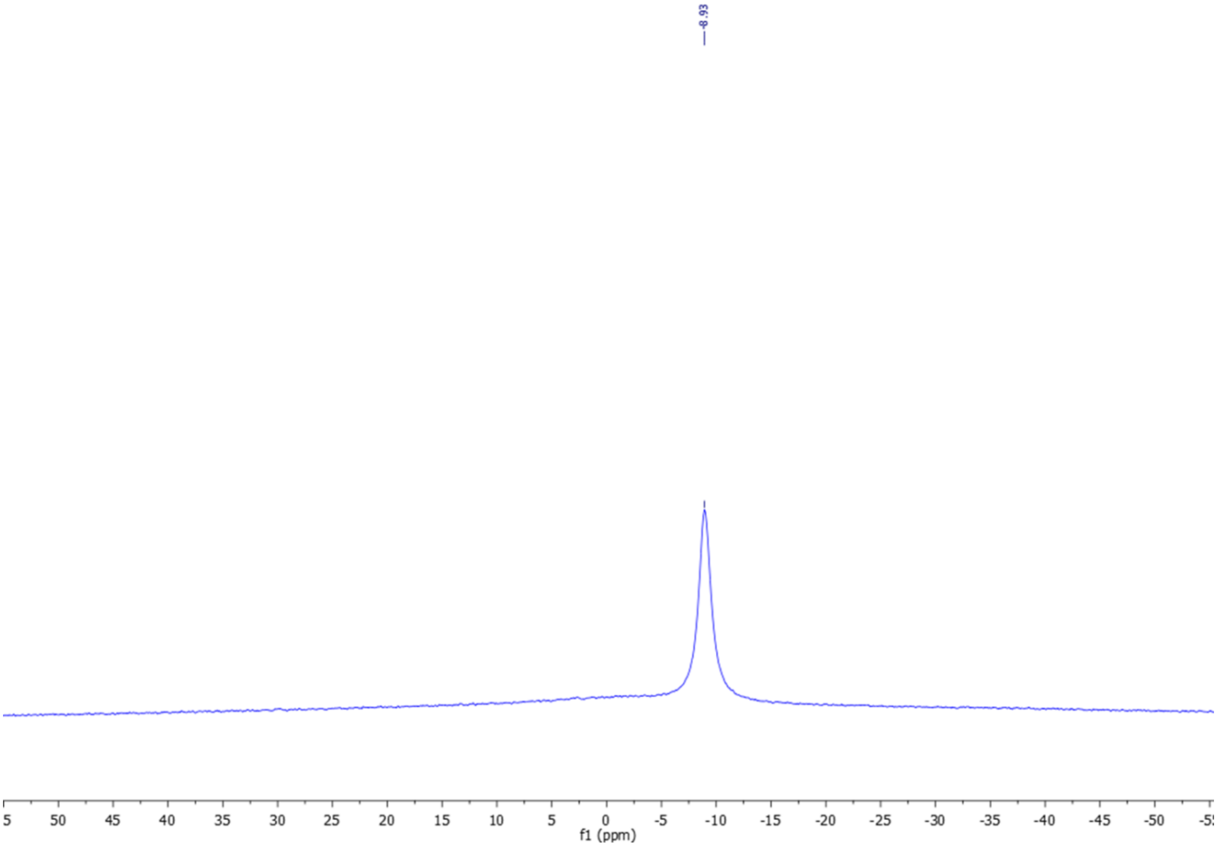

Figure SI 86: ^{11}B NMR spectrum of the analytical sample of 3-(4-chlorophenyl)pent-4-en-2-imine tris(perfluorophenyl)borane complex **14** (128 MHz, Chloroform-*d*).

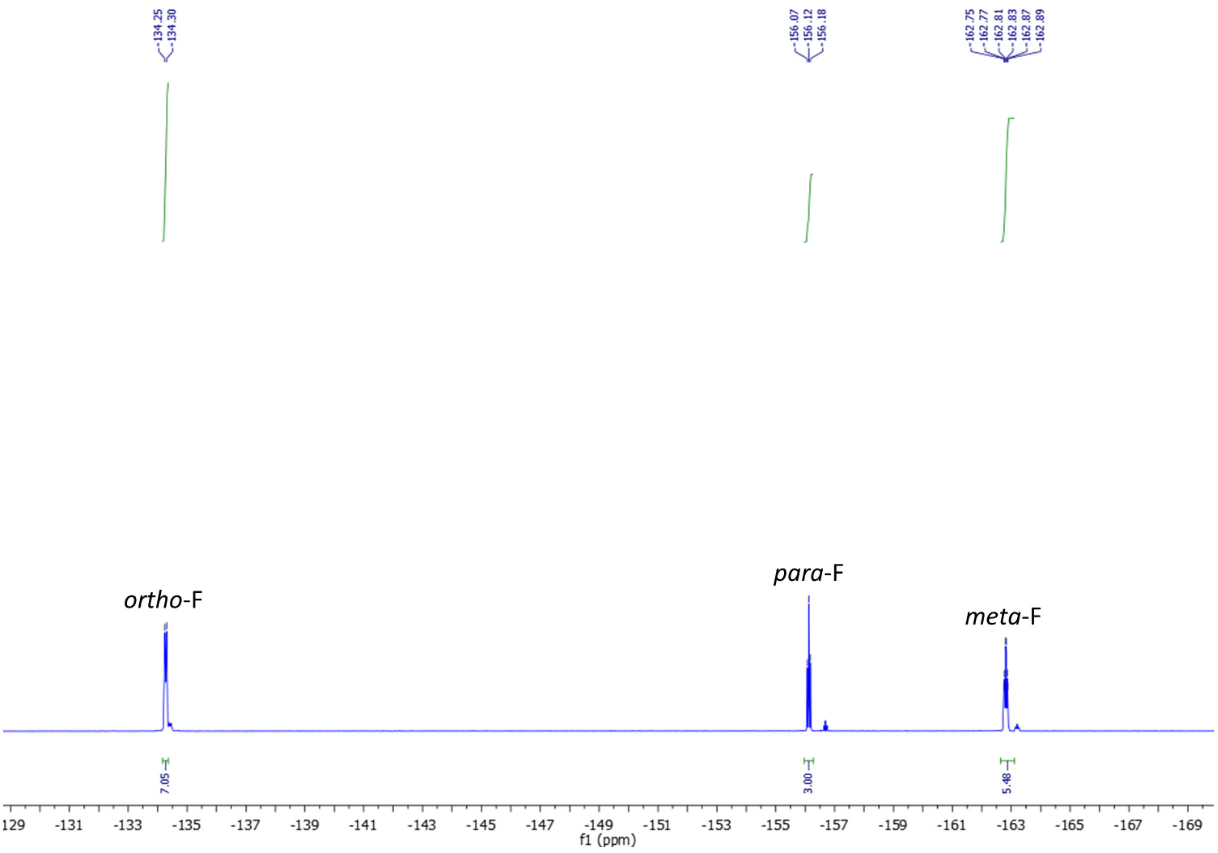

Figure SI 87: ^{19}F NMR spectrum of the analytical sample of 3-(4-chlorophenyl)pent-4-en-2-imine tris(perfluorophenyl)borane complex **14** (377 MHz, Chloroform-*d*).

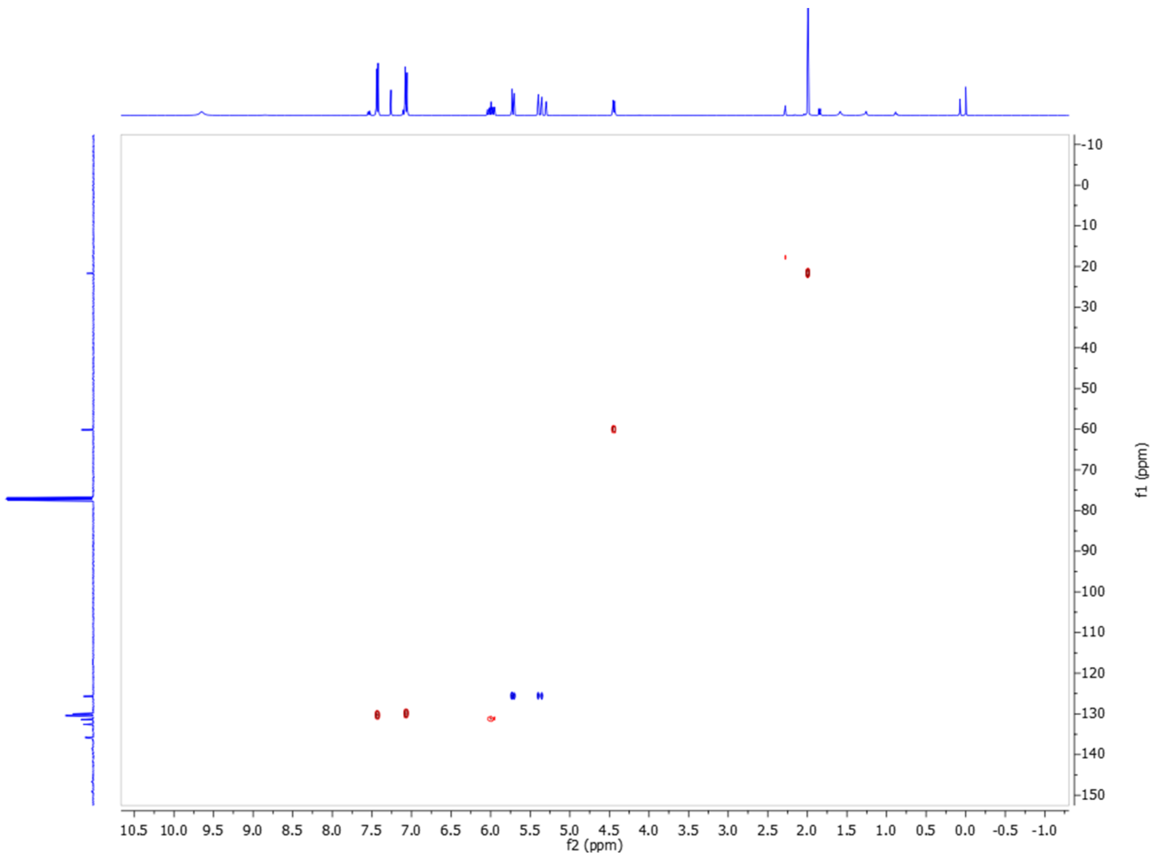

Figure SI 88: HSQC NMR spectrum of the analytical sample of 3-(4-chlorophenyl)pent-4-en-2-imine tris(perfluorophenyl)borane complex **14** (101 MHz, Chloroform-*d*).

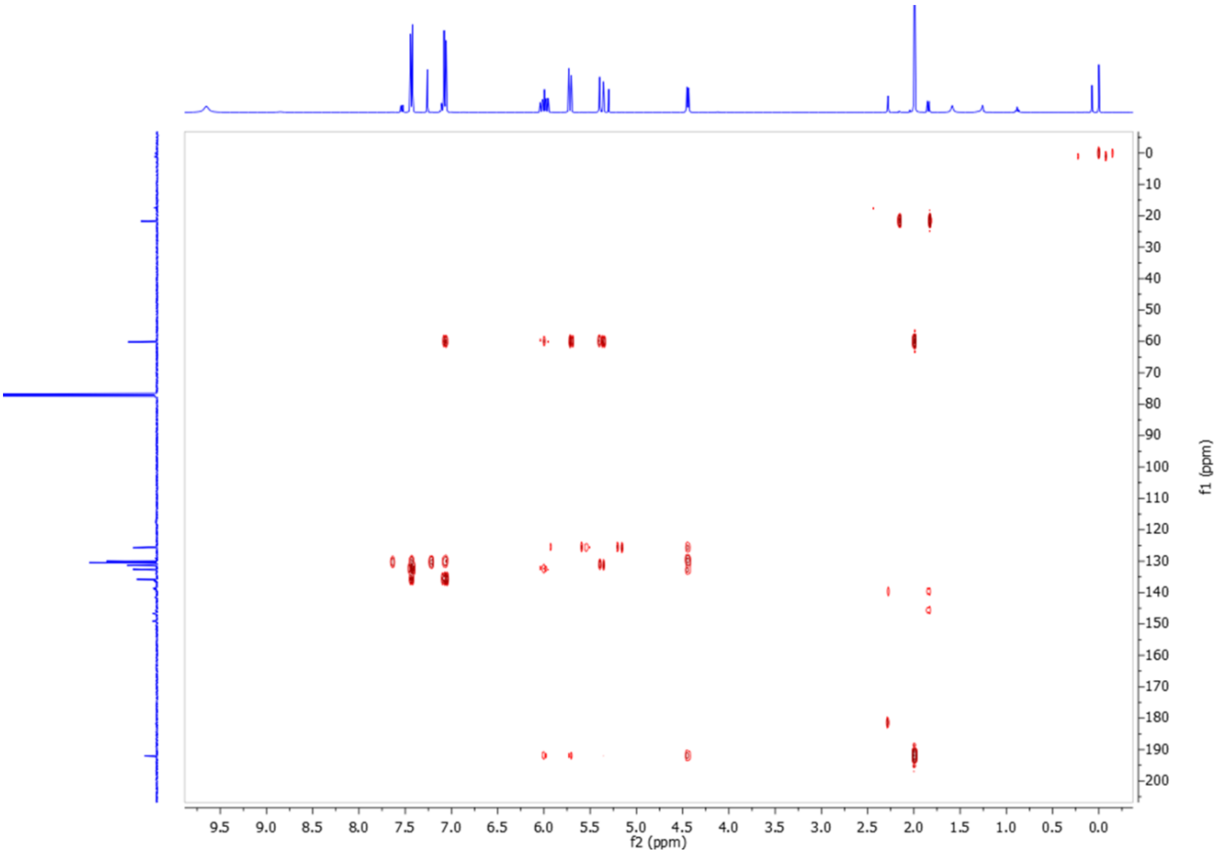

Figure SI 89: HMBC NMR spectrum of the analytical sample of 3-(4-chlorophenyl)pent-4-en-2-imine tris(perfluorophenyl)borane complex **14** (101 MHz, Chloroform-*d*).

5.4.4 (*E*)-3-(4-chlorophenyl)pent-3-en-2-imine tris(perfluorophenyl)borane complex 14'

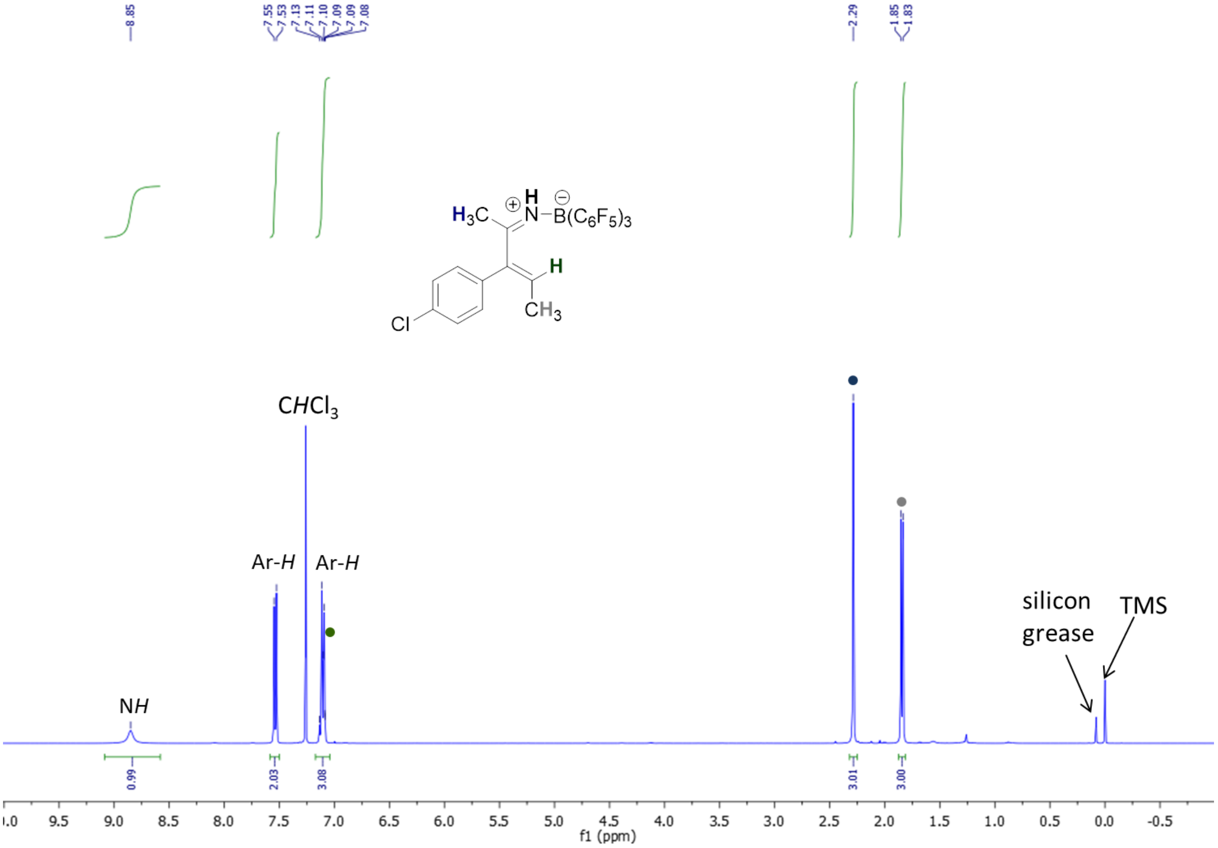

Figure SI 90: ^{1}H NMR spectrum of the analytical sample of the (*E*)-3-(4-chlorophenyl)pent-3-en-2-imine tris(perfluorophenyl)borane complex **14'** (400 MHz, Chloroform-*d*).

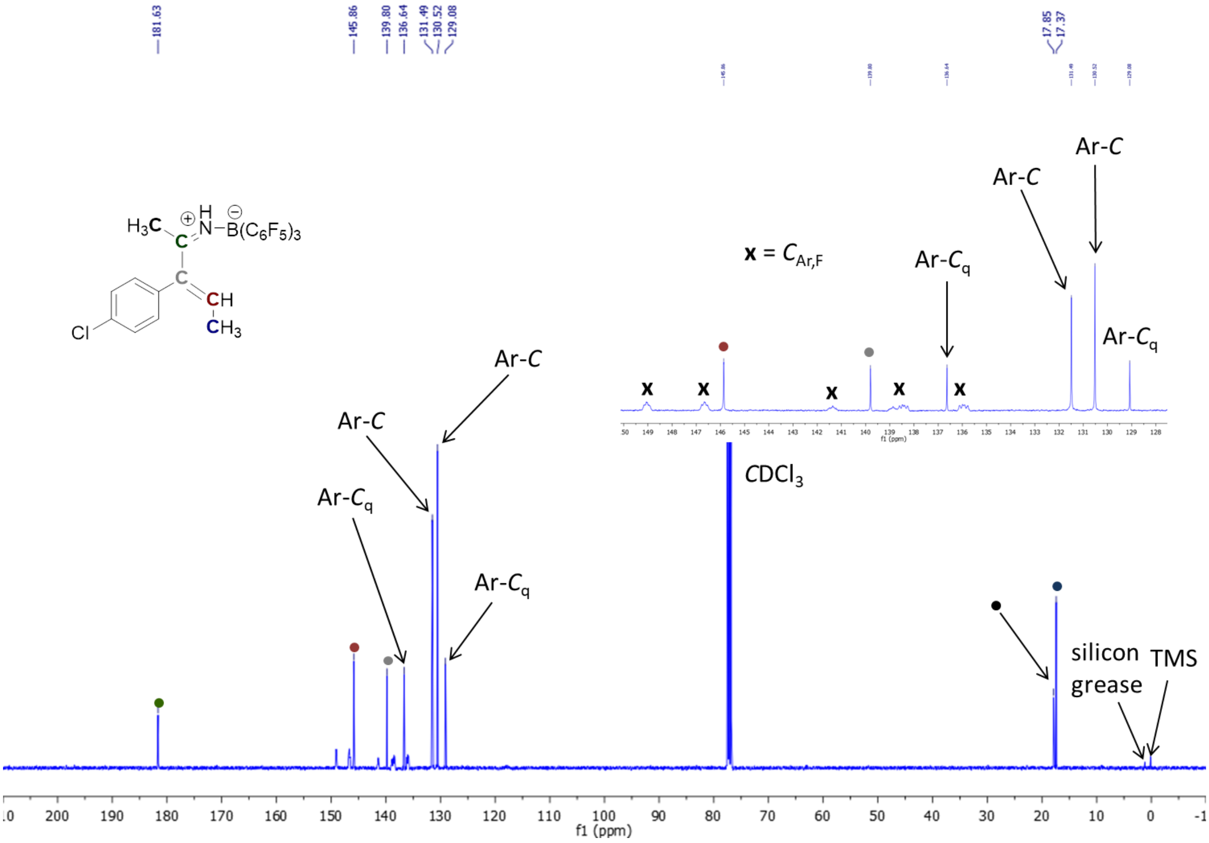

Figure SI 91: $^{13}C\{^{1}H\}$ NMR spectrum of the analytical sample of (*E*)-3-(4-chlorophenyl)pent-3-en-2-imine tris(perfluorophenyl)borane complex **14’** (101 MHz, Chloroform-*d*).

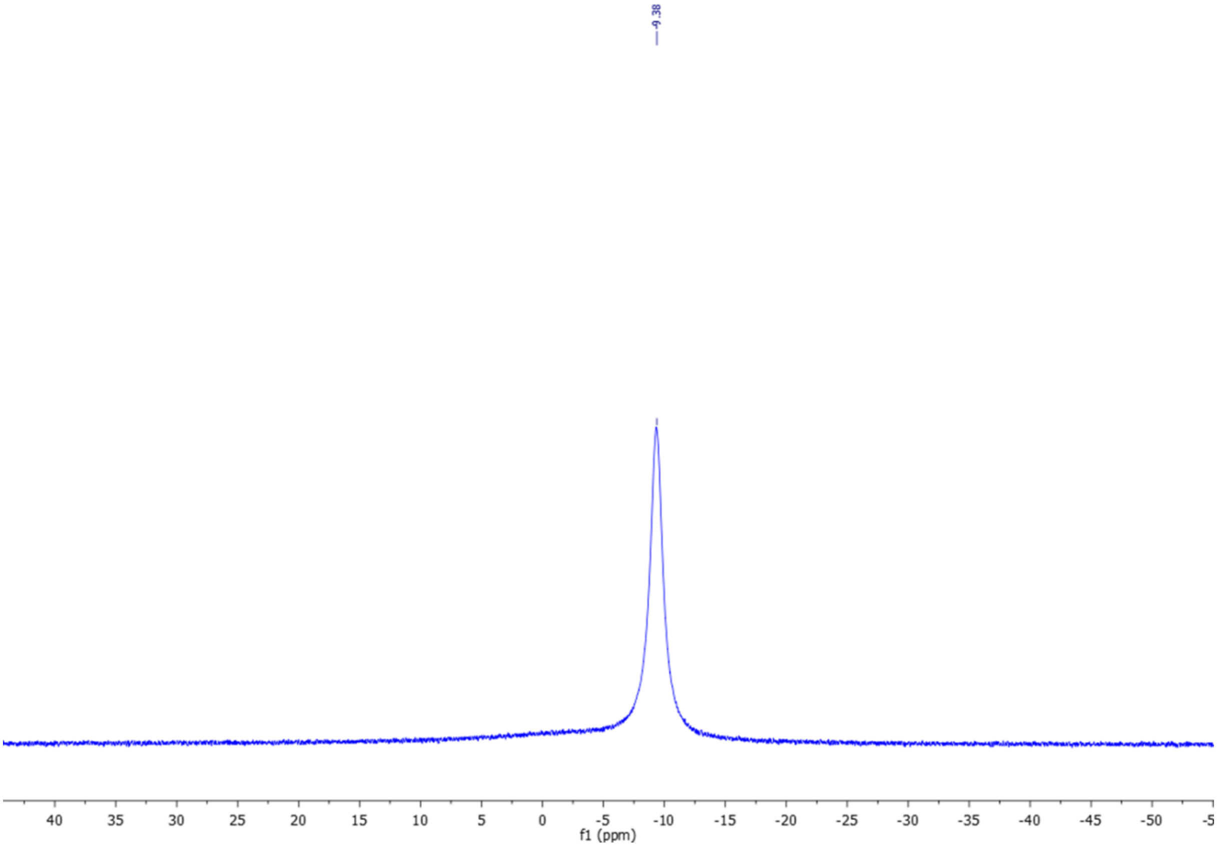

Figure SI 92: ^{11}B NMR spectrum of the analytical sample of the (*E*)-3-(4-chlorophenyl)pent-3-en-2-imine tris(perfluorophenyl)borane complex **14'** (128 MHz, Chloroform-*d*).

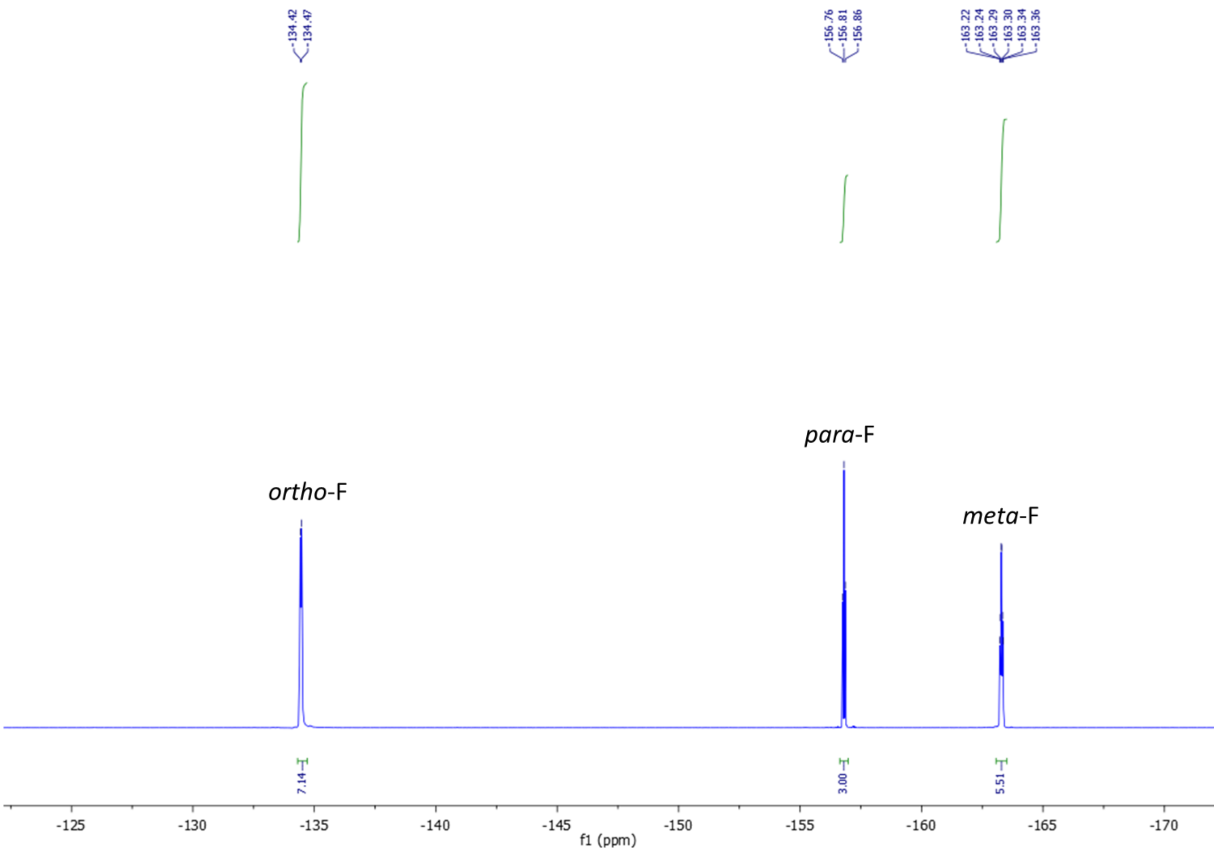

Figure SI 93: ^{19}F NMR spectrum of the analytical sample of the (*E*)-3-(4-chlorophenyl)pent-3-en-2-imine tris(perfluorophenyl)borane complex **14’** (377 MHz, Chloroform-*d*).

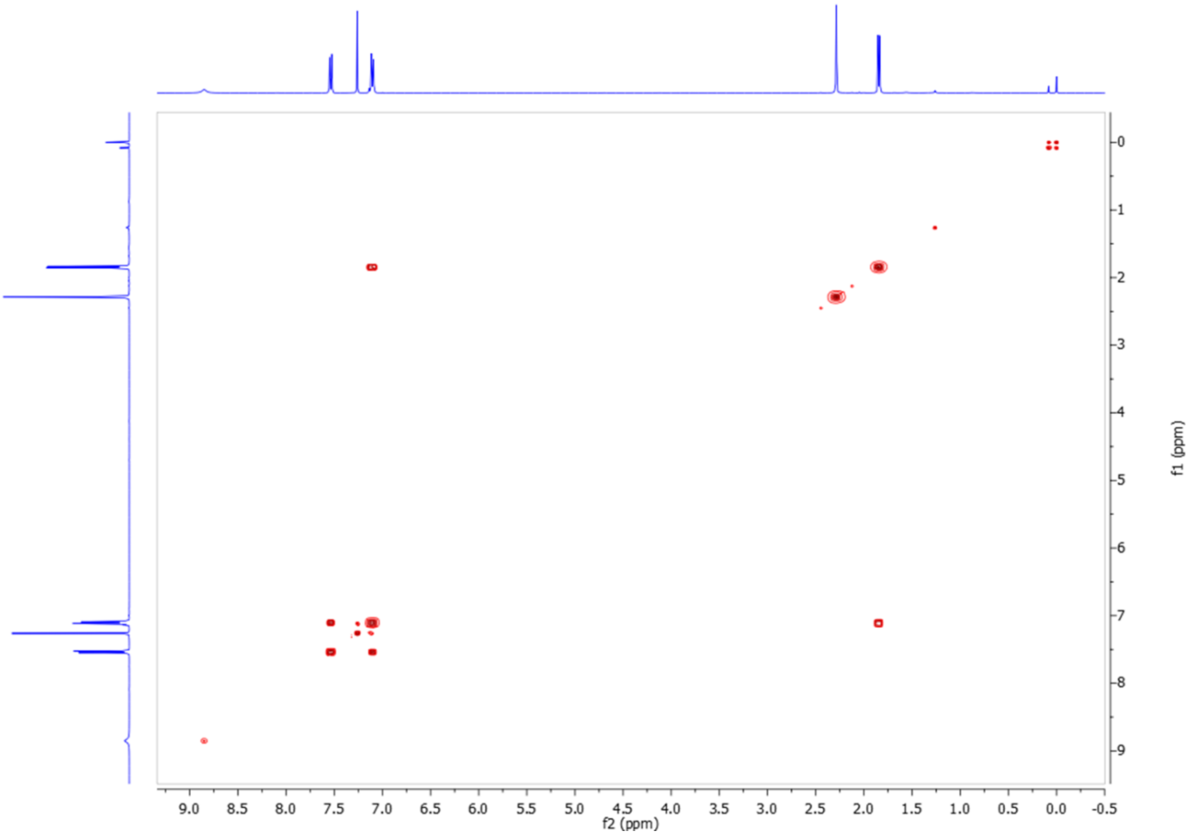

Figure SI 94: COSY NMR spectrum of the analytical sample of the (*E*)-3-(4-chlorophenyl)pent-3-en-2-imine tris(perfluorophenyl)borane complex **14’** (400 MHz, Chloroform-*d*).

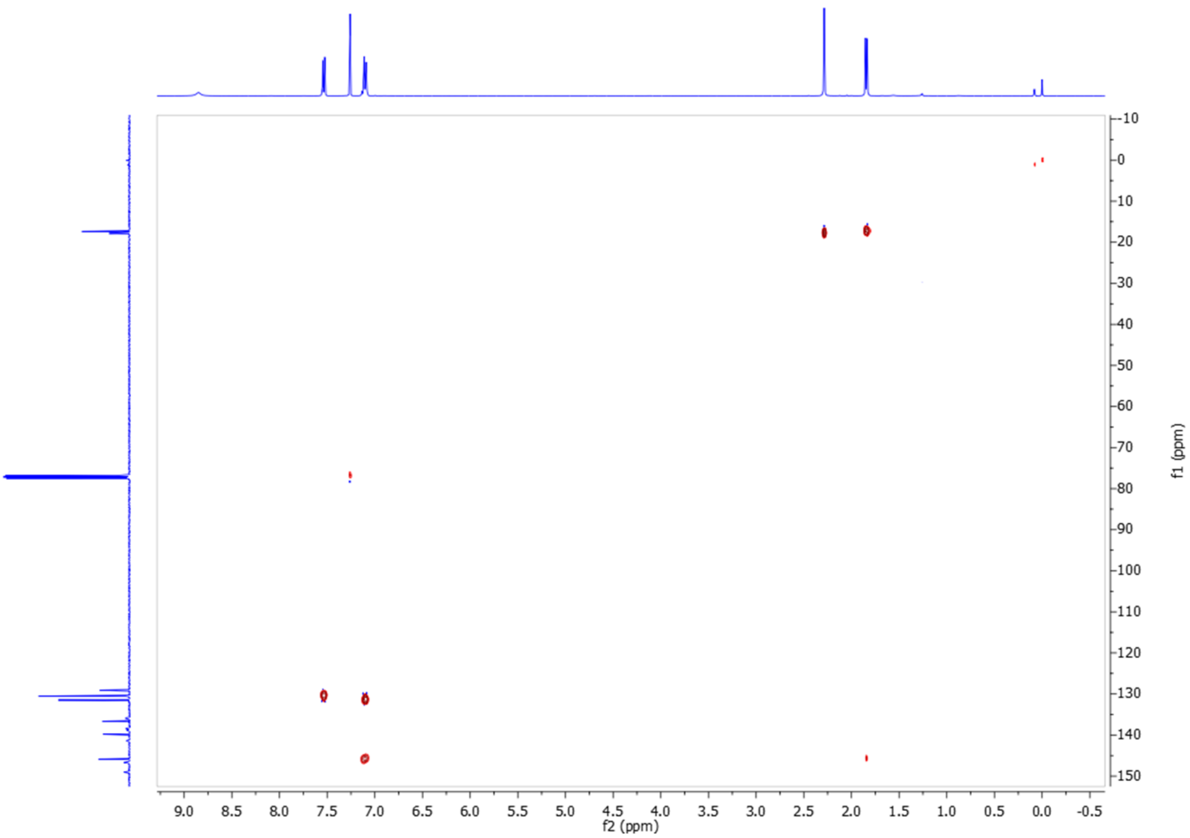

Figure SI 95: HSQC NMR spectrum of the analytical sample of the (*E*)-3-(4-chlorophenyl)pent-3-en-2-imine tris(perfluorophenyl)borane complex **14’** (101 MHz, Chloroform-*d*).

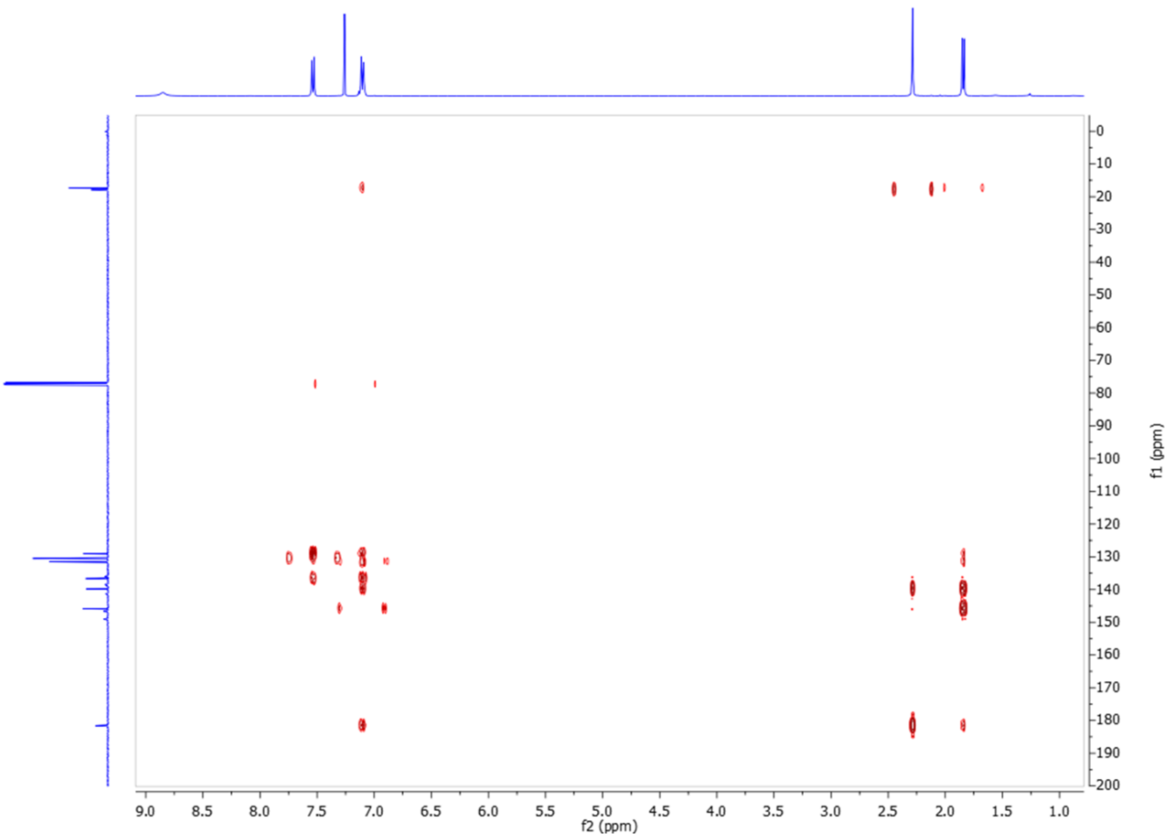

Figure SI 96: HMBC NMR spectrum of the analytical sample of the (*E*)-3-(4-chlorophenyl)pent-3-en-2-imine tris(perfluorophenyl)borane complex **14'** (101 MHz, Chloroform-*d*).

5.4.5 3-(4-(Trifluoromethyl)phenyl)pent-4-en-2-imine tris(perfluorophenyl)borane complex 15

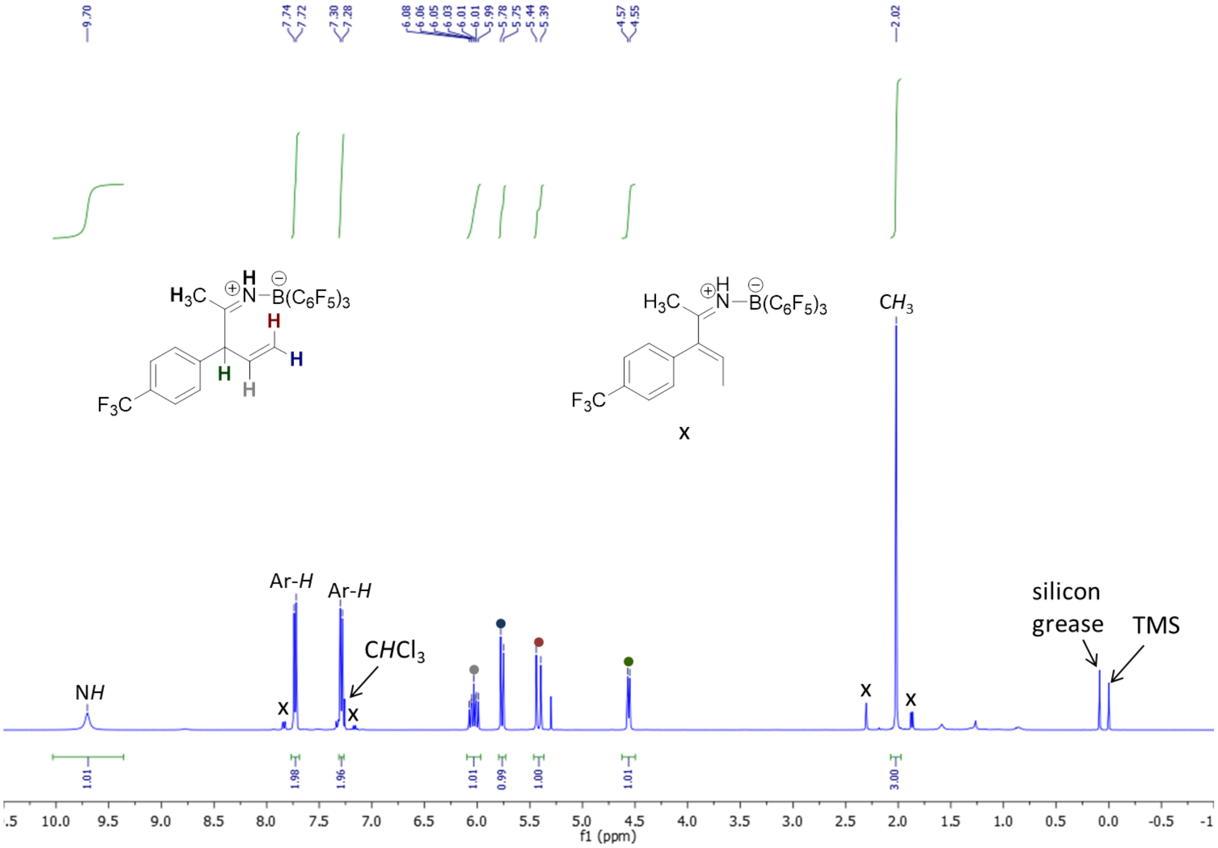

Figure SI 97: ^{1}H NMR of the reaction mixture containing **15** and **15'** in a 95 to 5 ratio after purification of the crude reaction product via column chromatography (400 MHz, Chloroform-*d*).

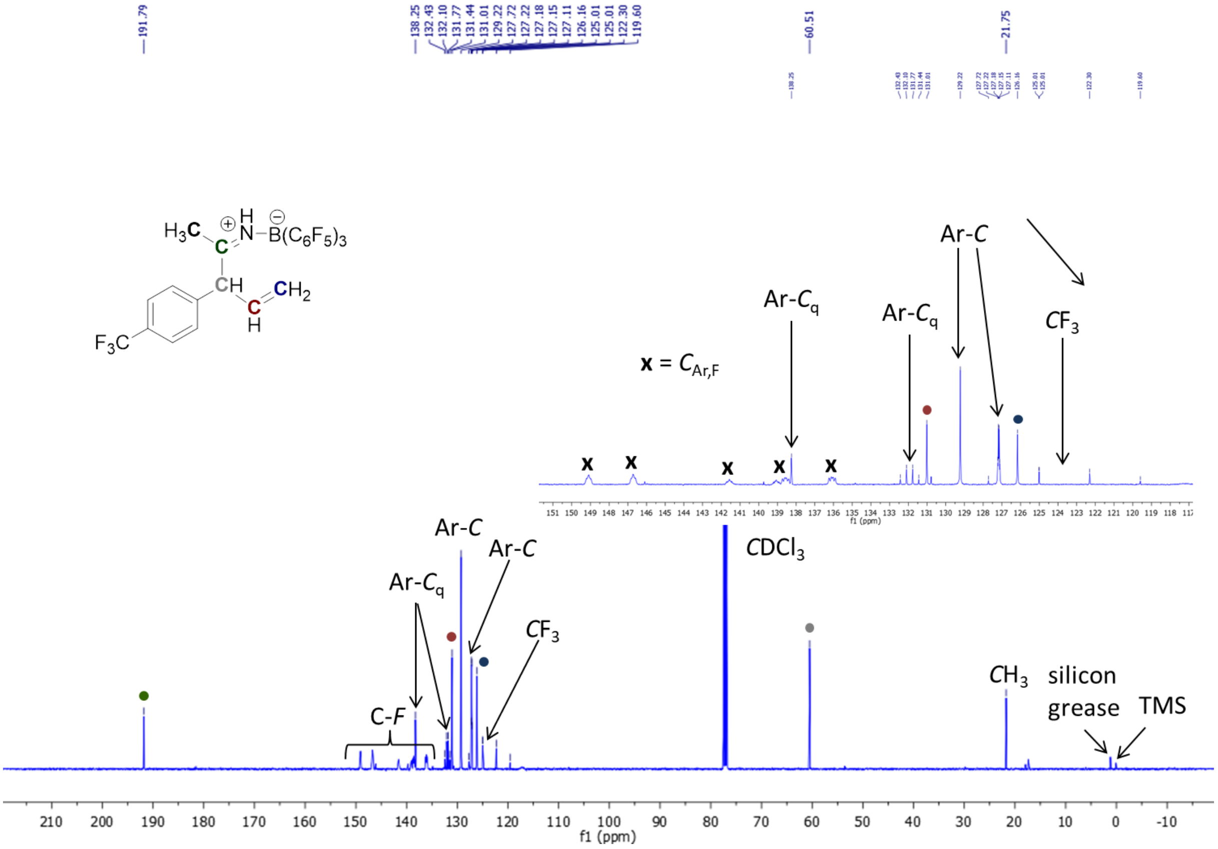

Figure SI 98: $^{13}C\{^1H\}$ NMR spectrum of the analytical sample of 3-(4-(trifluoromethyl)phenyl)pent-4-en-2-imine tris(perfluorophenyl)borane complex **15** (101 MHz, Chloroform-*d*).

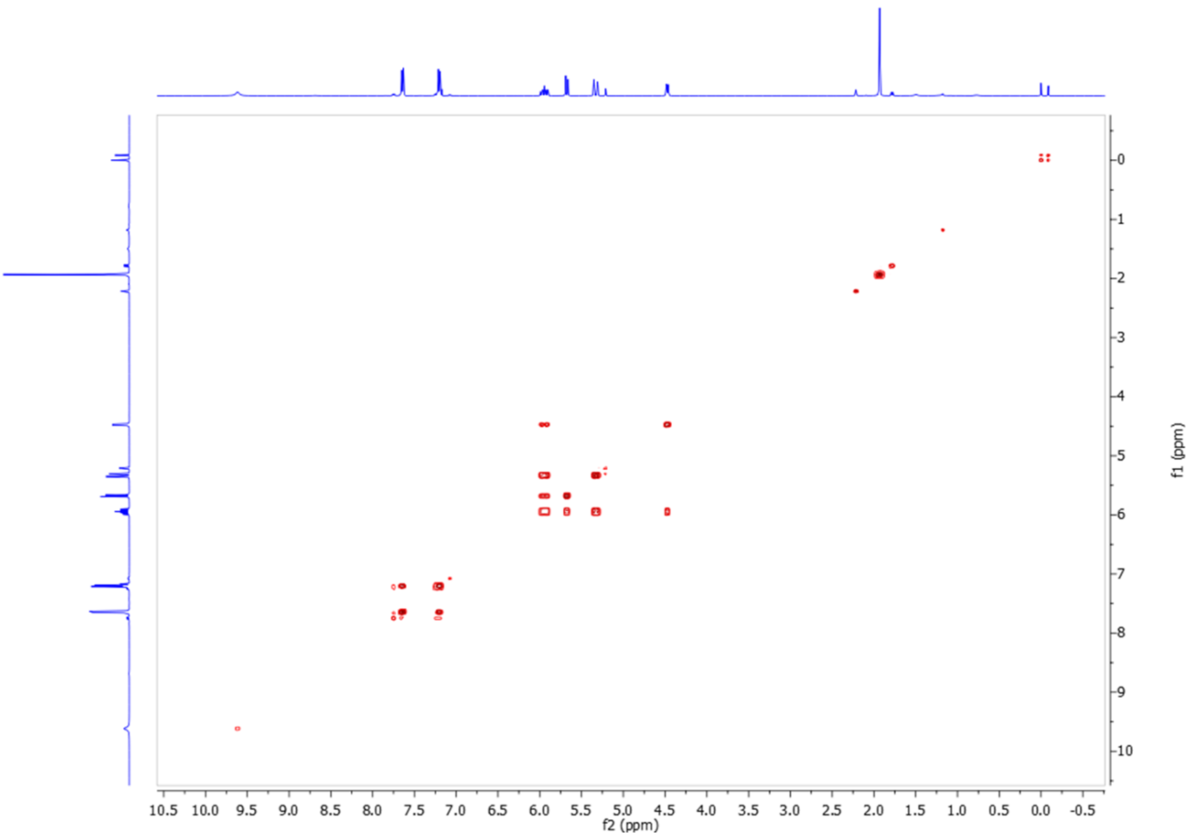

Figure SI 99: COSY NMR spectrum of the analytical sample of the 3-(4-(trifluoromethyl)phenyl)pent-4-en-2-imine tris(perfluorophenyl)borane complex **15** (400 MHz, Chloroform-*d*).

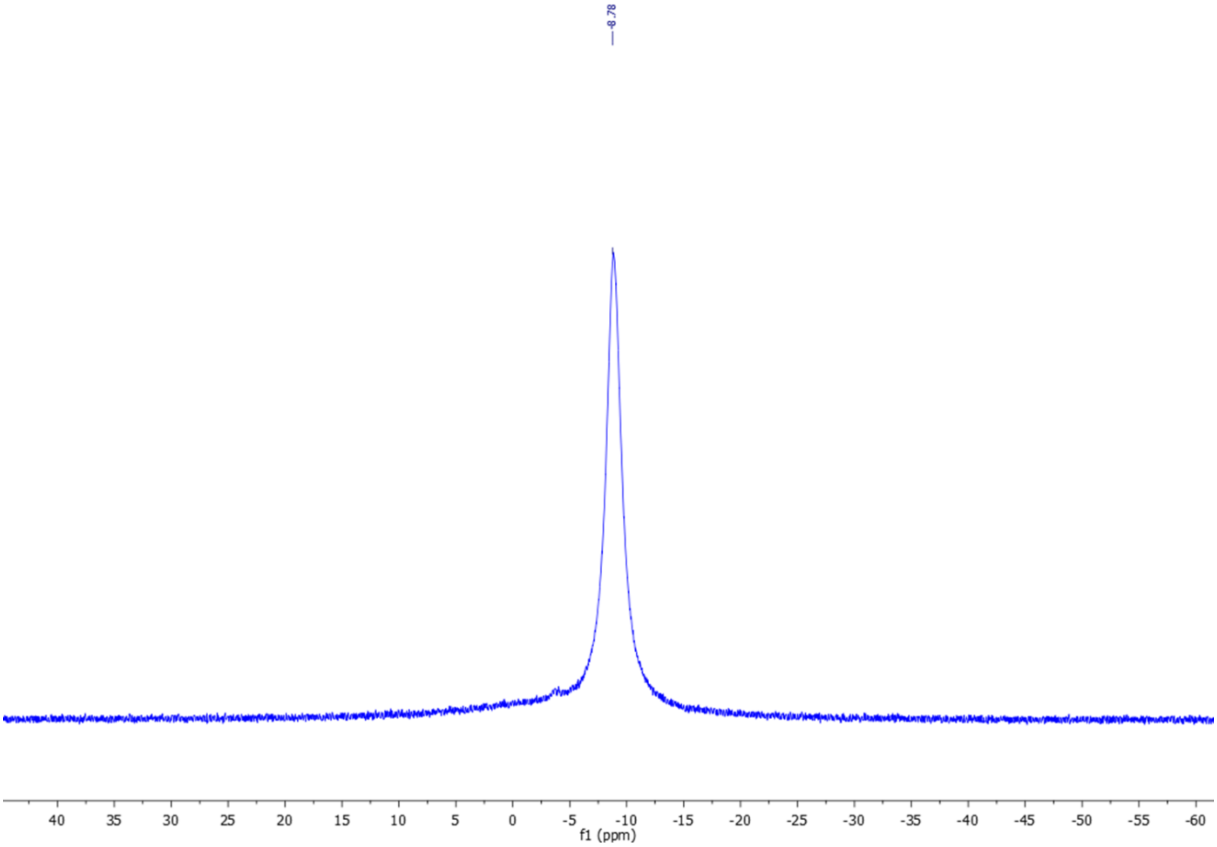

Figure SI 100: ^{11}B NMR spectrum of the analytical sample of the 3-(4-(trifluoromethyl)phenyl)pent-4-en-2-imine tris(perfluorophenyl)borane complex **15** (128 MHz, Chloroform-*d*).

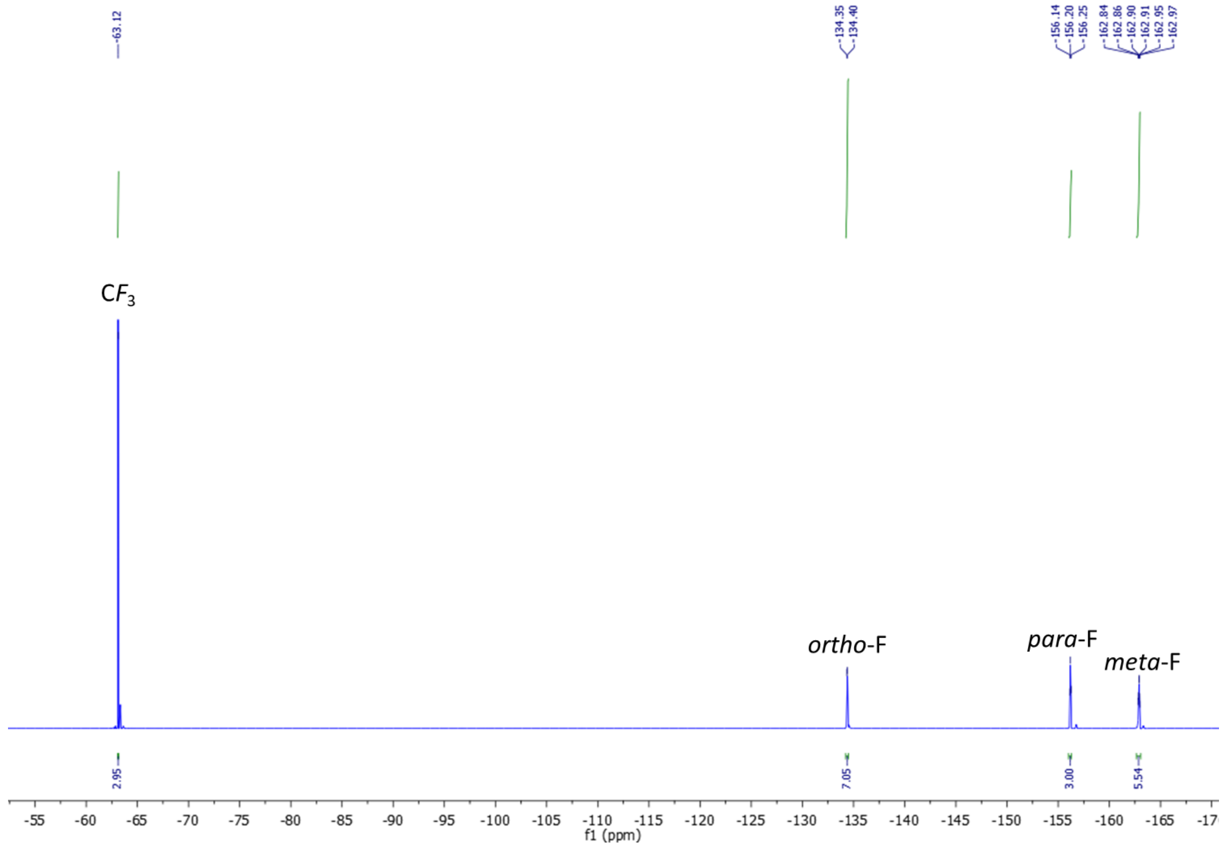

Figure SI 101: ^{19}F NMR spectrum of the analytical sample of the 3-(4-(trifluoromethyl)phenyl)pent-4-en-2-imine tris(perfluorophenyl)borane complex **15** (377 MHz, Chloroform-*d*).

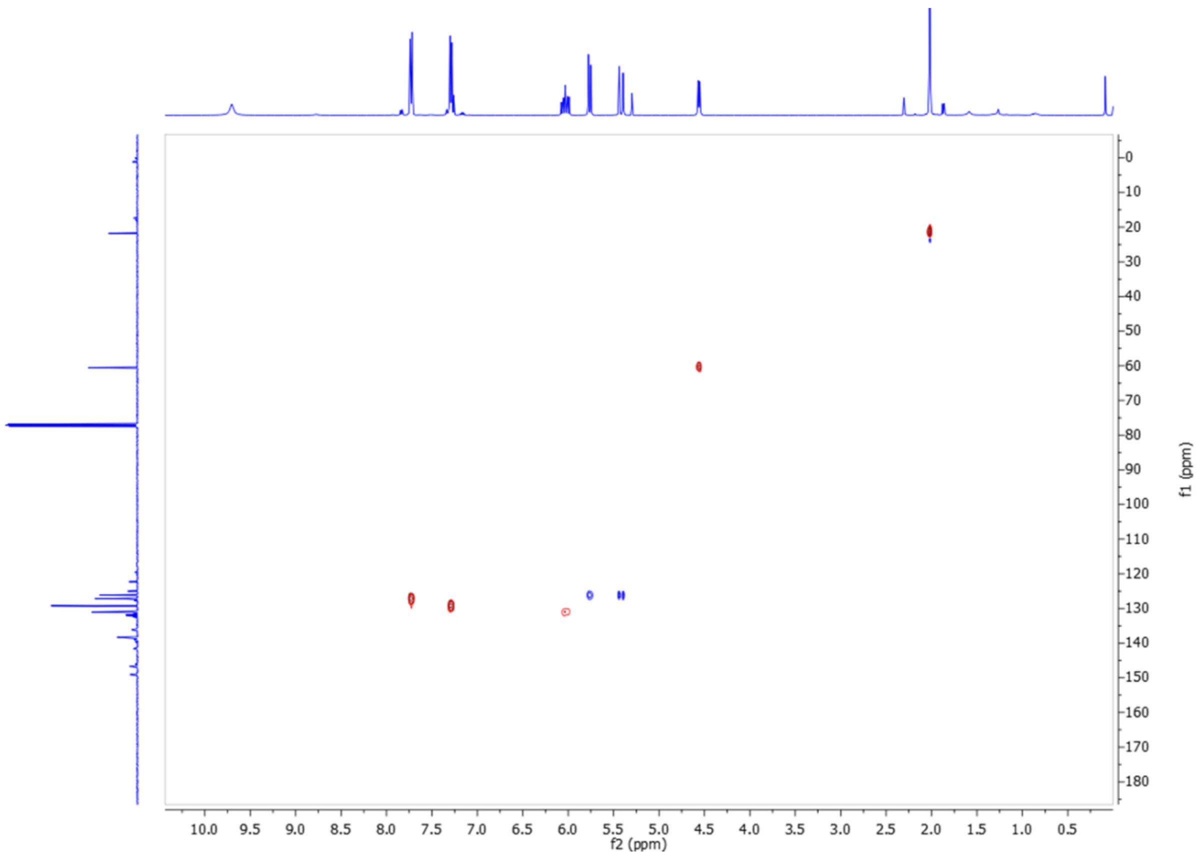

Figure SI 102: HSQC NMR spectrum of the analytical sample of the 3-(4-(trifluoromethyl)phenyl)pent-4-en-2-imine tris(perfluorophenyl)borane complex **15** (101 MHz, Chloroform-*d*).

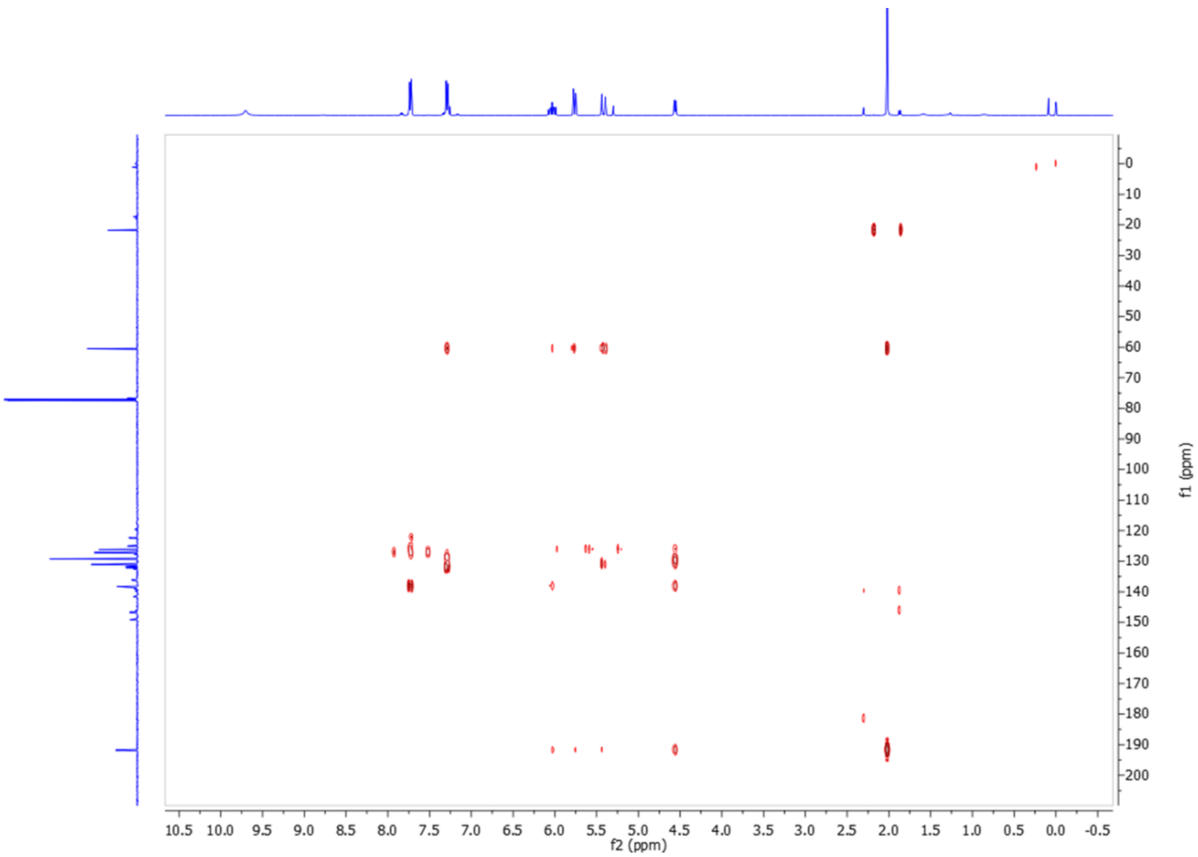

Figure SI 103: HMBC NMR spectrum of the analytical sample of the 3-(4-(trifluoromethyl)phenyl)pent-4-en-2-imine tris(perfluorophenyl)borane complex **15** (101 MHz, Chloroform-*d*).

5.4.6 (*E*)-3-(4-(trifluoromethyl)phenyl)pent-3-en-2-imine tris(perfluorophenyl)borane complex 15’

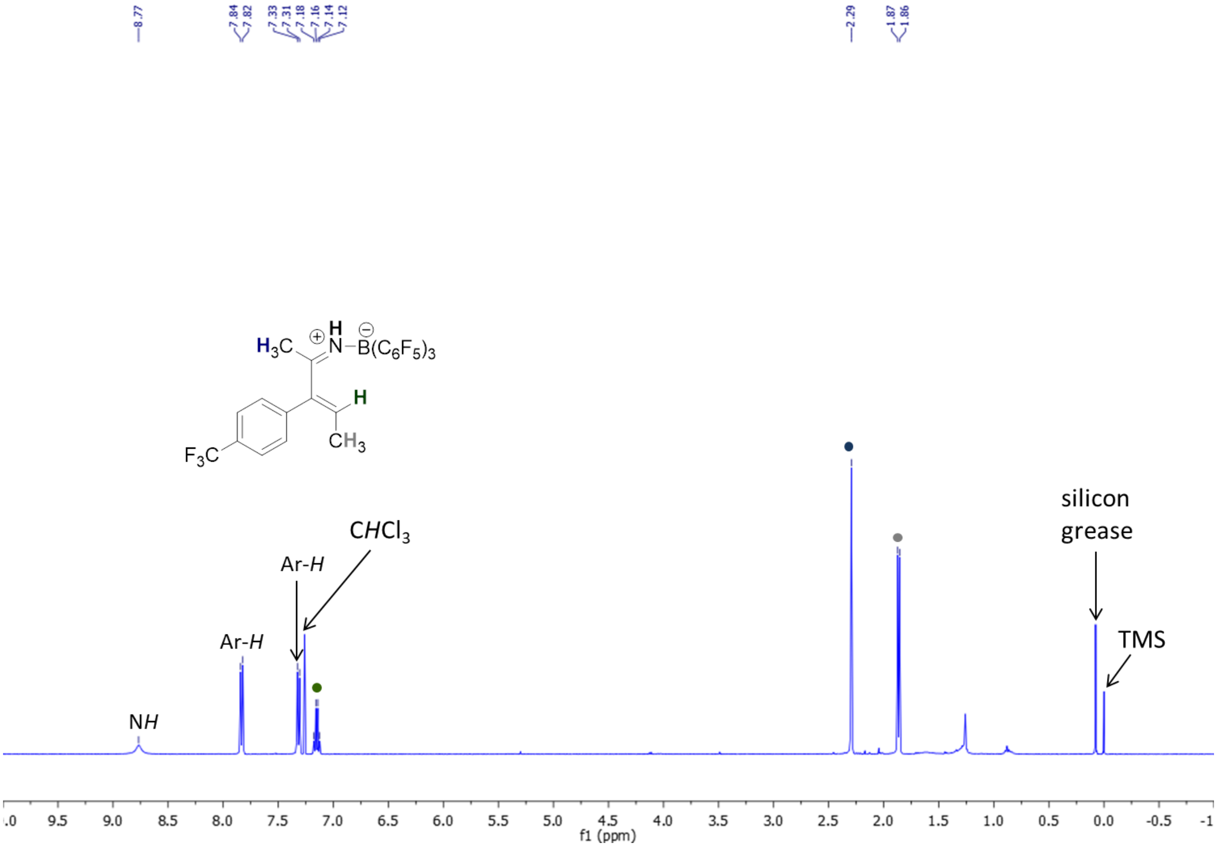

Figure SI 104: ^{1}H NMR spectrum of the analytical sample of the (*E*)-3-(4-(trifluoromethyl)phenyl)pent-3-en-2-imine tris(perfluorophenyl)borane complex **15’** (400 MHz, Chloroform-*d*).

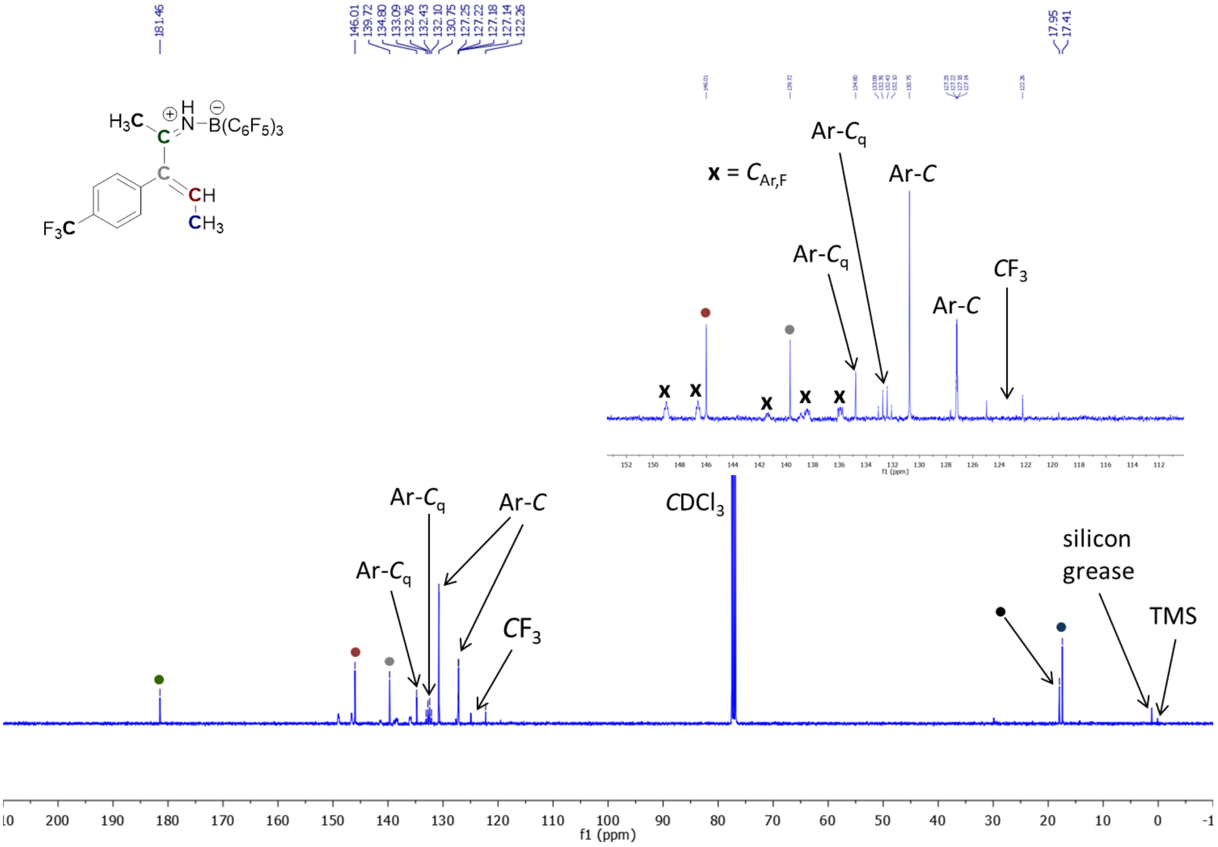

Figure SI 105: $^{13}C\{^{1}H\}$ NMR spectrum of the analytical sample of (*E*)-3-(4-(trifluoromethyl)phenyl)pent-3-en-2-imine tris(perfluorophenyl)borane complex **15’** (101 MHz, Chloroform-*d*).

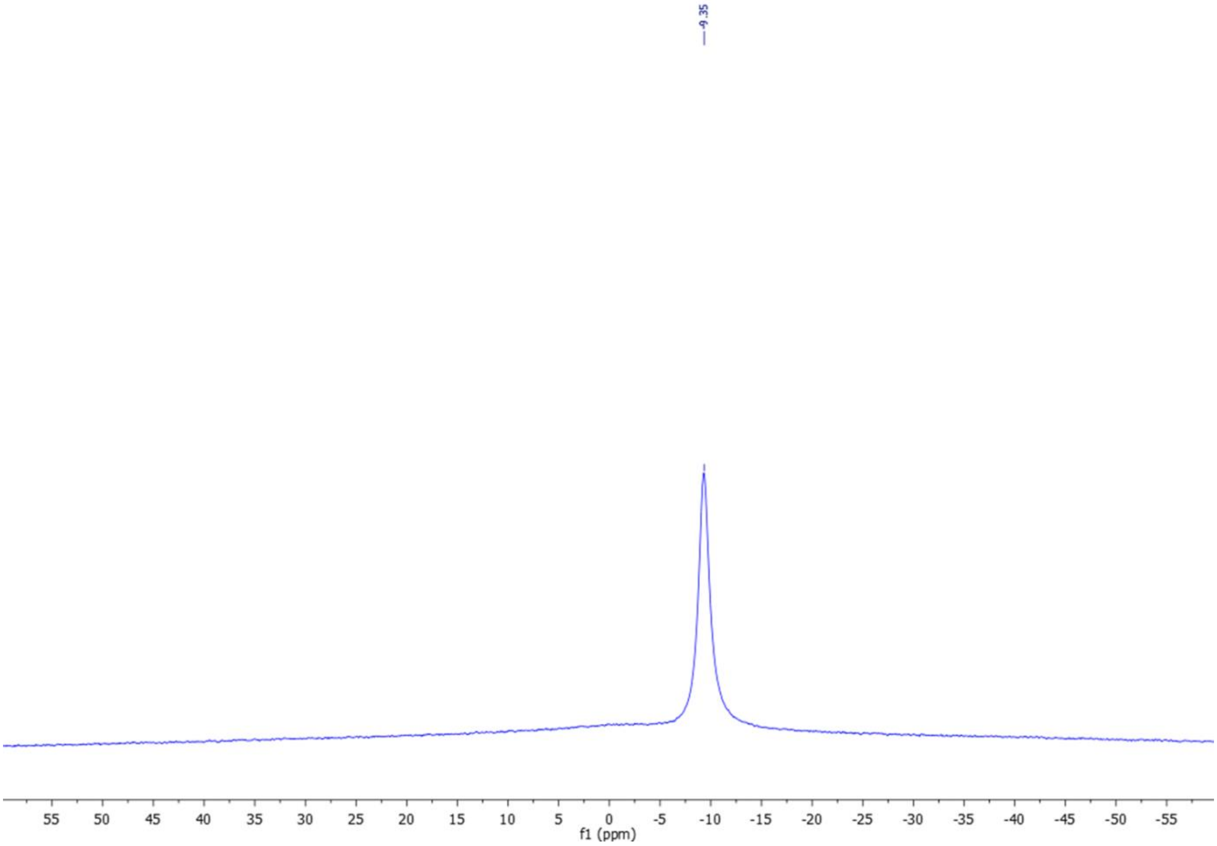

Figure SI 106: ^{11}B NMR spectrum of the analytical sample of the (*E*)-3-(4-(trifluoromethyl)phenyl)pent-3-en-2-imine tris(perfluorophenyl)borane complex **15’** (128 MHz, Chloroform-*d*).

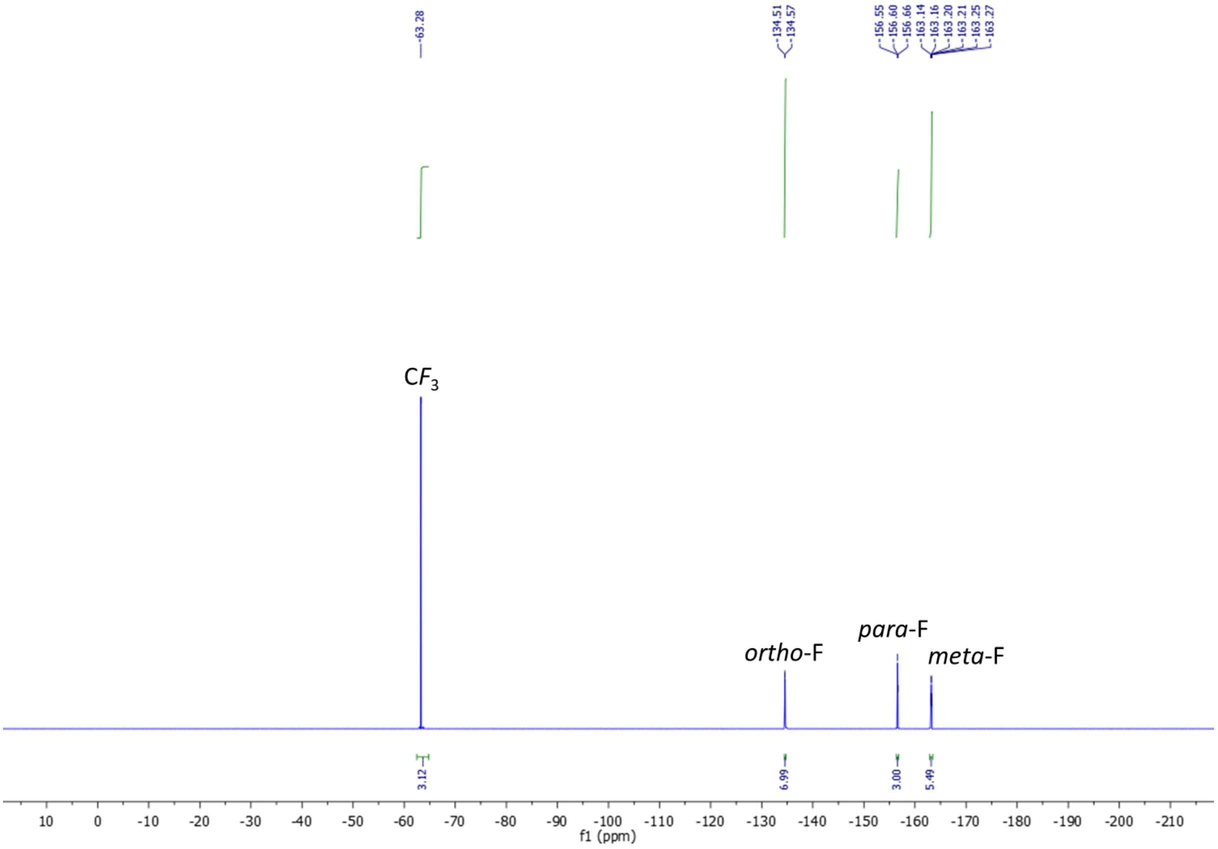

Figure SI 107: ^{19}F NMR spectrum of the analytical sample of the (*E*)-3-(4-(trifluoromethyl)phenyl)pent-3-en-2-imine tris(perfluorophenyl)borane complex **15’** (377 MHz, Chloroform-*d*).

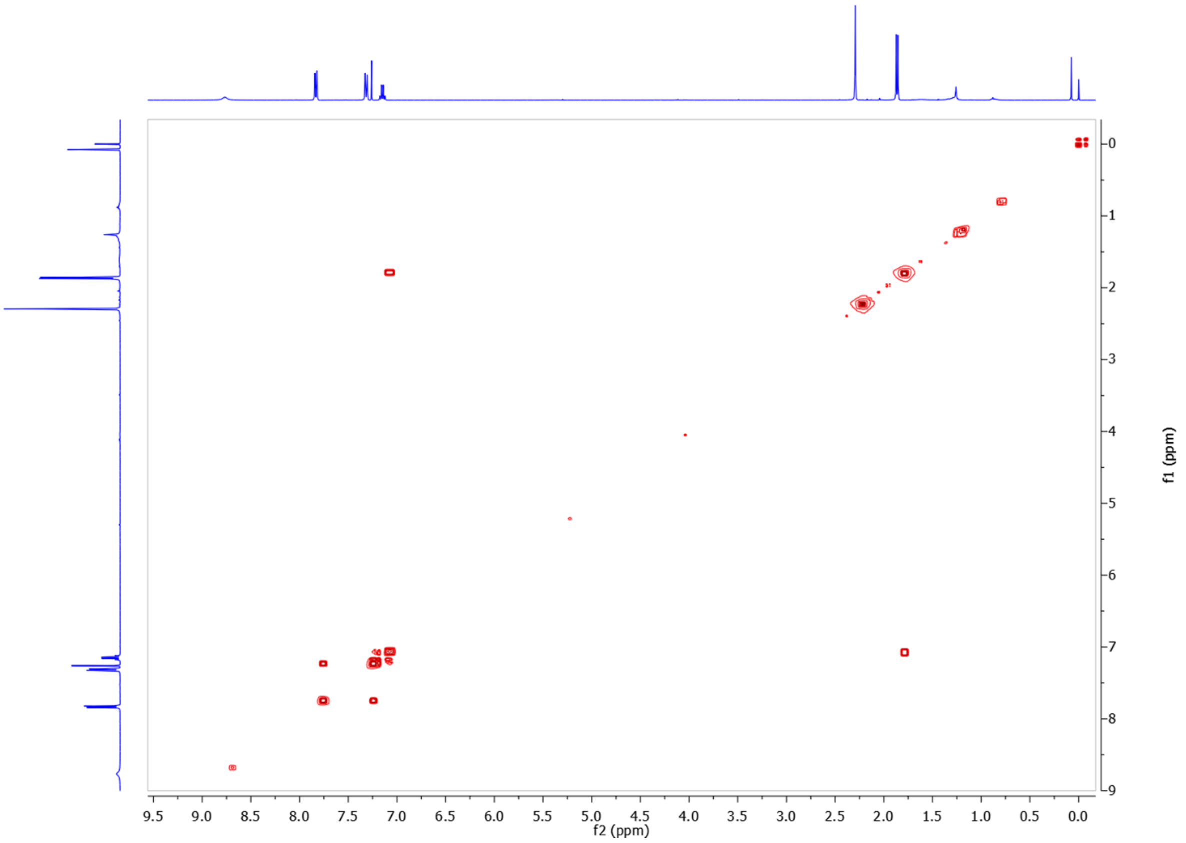

Figure SI 108: COSY NMR spectrum of the analytical sample of the (*E*)-3-(4-(trifluoromethyl)phenyl)pent-3-en-2-imine tris(perfluorophenyl)borane complex **15’** (400 MHz, Chloroform-*d*).

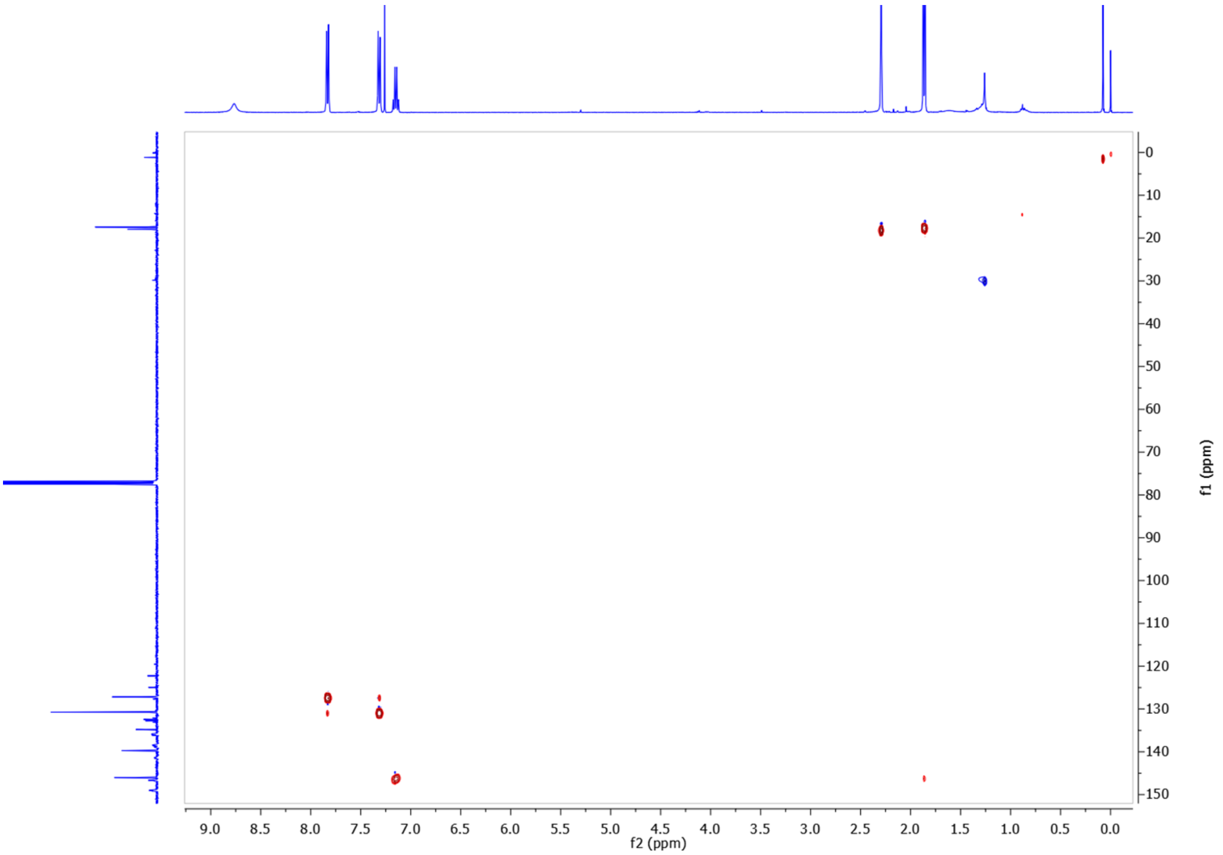

Figure SI 109: HSQC NMR spectrum of the analytical sample of the (*E*)-3-(4-(trifluoromethyl)phenyl)pent-3-en-2-imine tris(perfluorophenyl)borane complex **15’** (101 MHz, Chloroform-*d*).

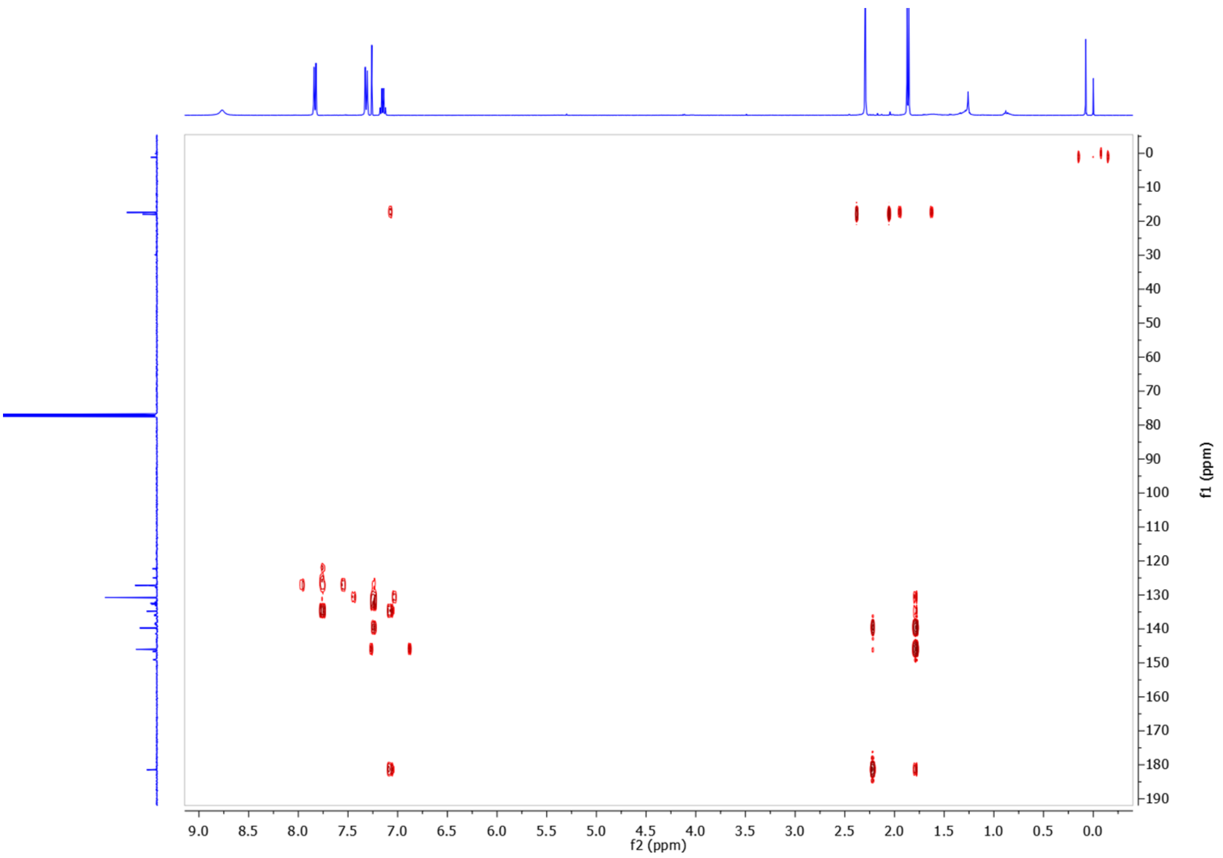

Figure SI 110: HMBC NMR spectrum of the analytical sample of the (*E*)-3-(4-(trifluoromethyl)phenyl)pent-3-en-2-imine tris(perfluorophenyl)borane complex **15'** (101 MHz, Chloroform-*d*).

5.4.7 3-(4-(tert-butyl)phenyl)pent-4-en-2-imine tris(perfluorophenyl)borane complex 16

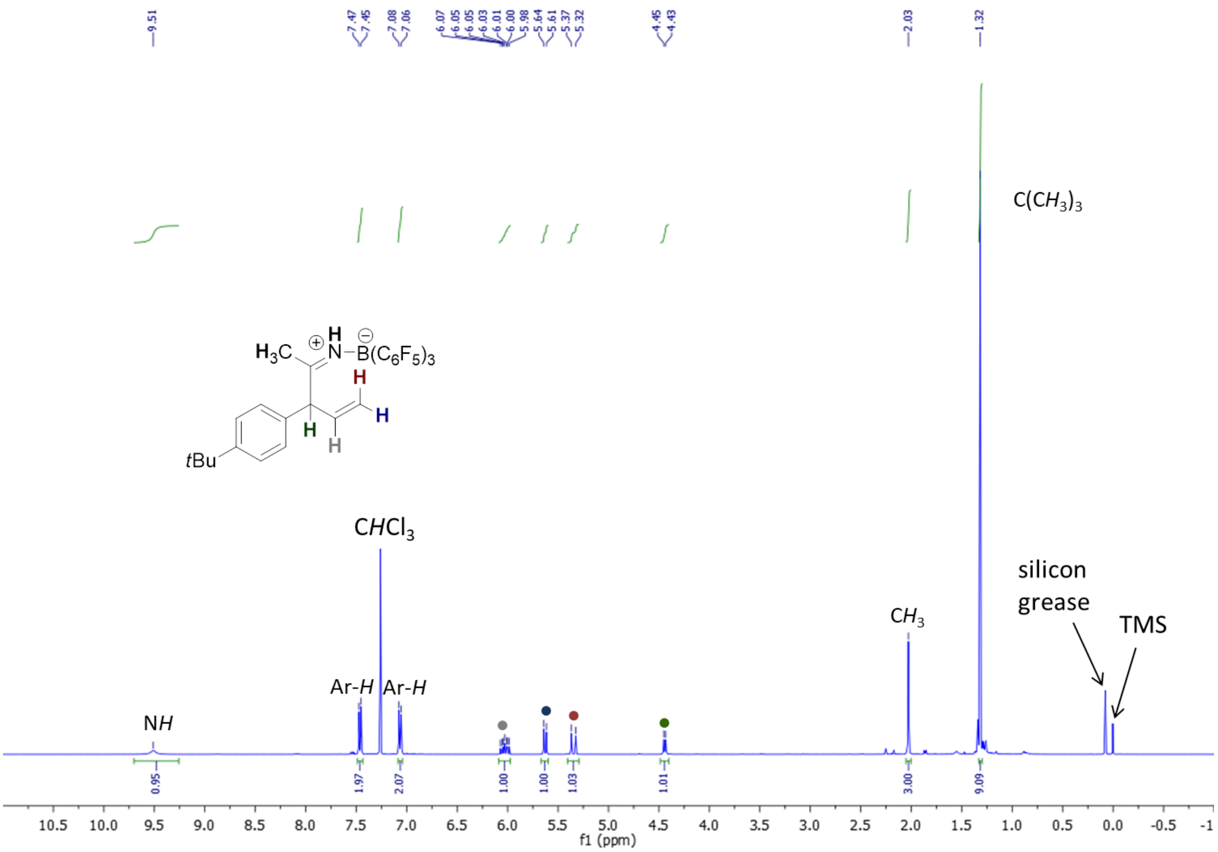

Figure SI 111: ^{1}H NMR spectrum of the analytical sample of the 3-(4-(*tert*-butyl)phenyl)pent-4-en-2-imine tris(perfluorophenyl)borane complex **16** (400 MHz, Chloroform-*d*).

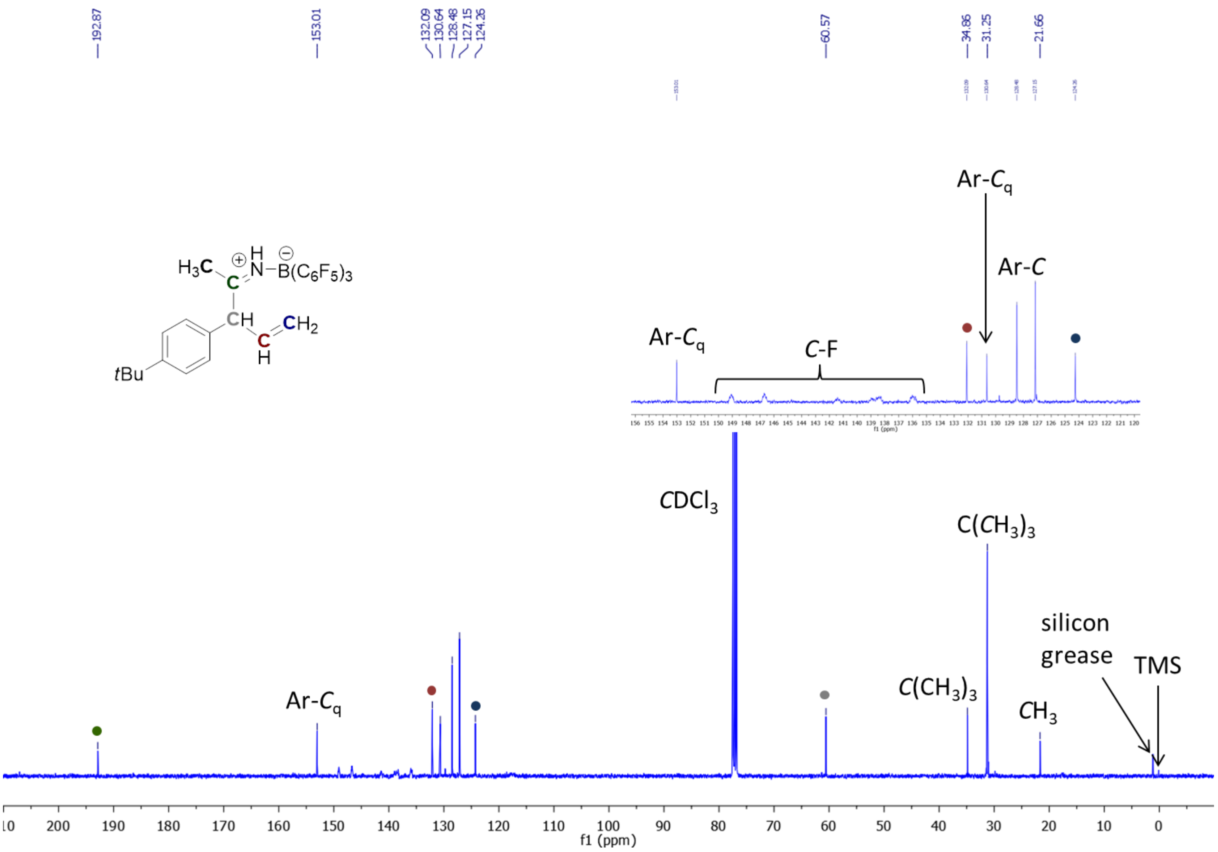

Figure SI 112: $^{13}C\{^{1}H\}$ NMR spectrum of the analytical sample of 3-(4-(*tert*-butyl)phenyl)pent-4-en-2-imine tris(perfluorophenyl)borane complex **16** (101 MHz, Chloroform-*d*).

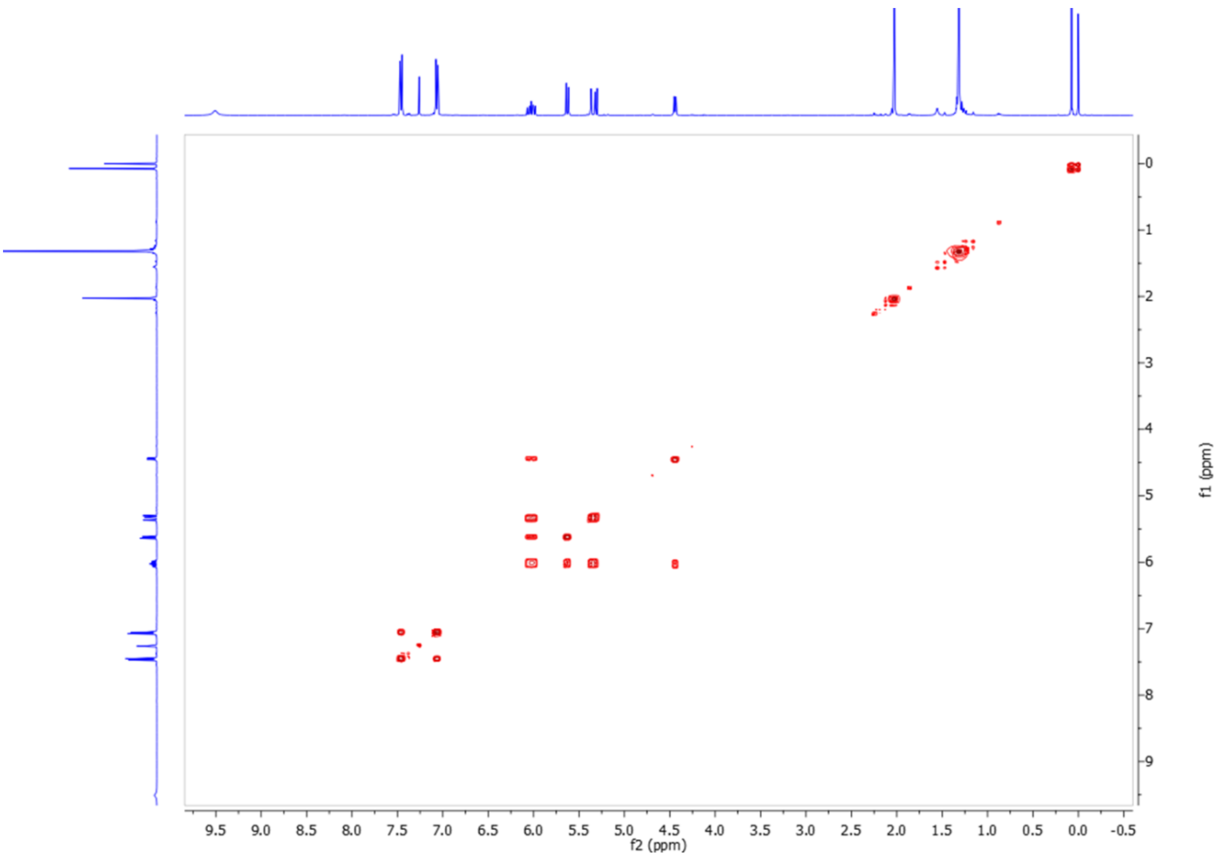

Figure SI 113: COSY NMR spectrum of the analytical sample of 3-(4-(*tert*-butyl)phenyl)pent-4-en-2-imine tris(perfluorophenyl)borane complex **16** (400 MHz, Chloroform-*d*).

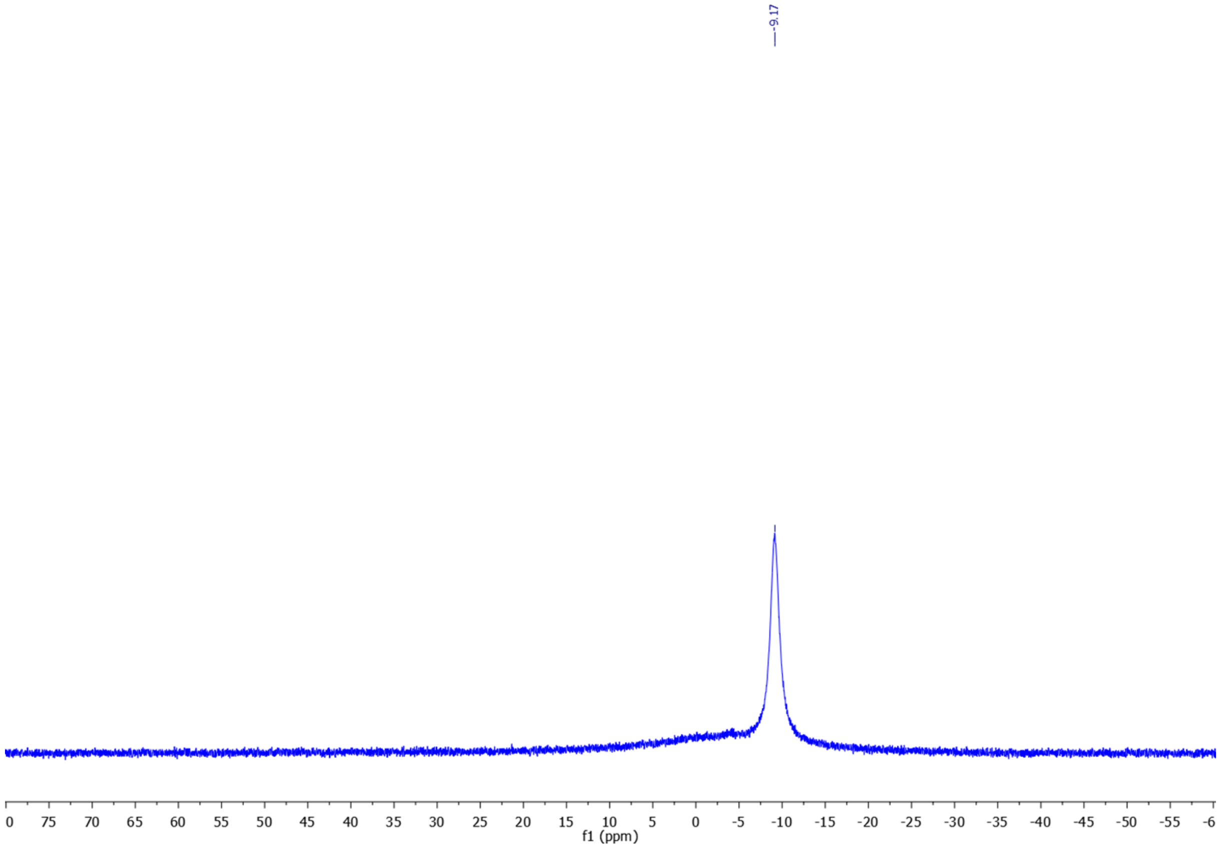

Figure SI 114: ^{11}B NMR spectrum of the analytical sample of 3-(4-(*tert*-butyl)phenyl)pent-4-en-2-imine tris(perfluorophenyl)borane complex **16** (128 MHz, Chloroform-*d*).

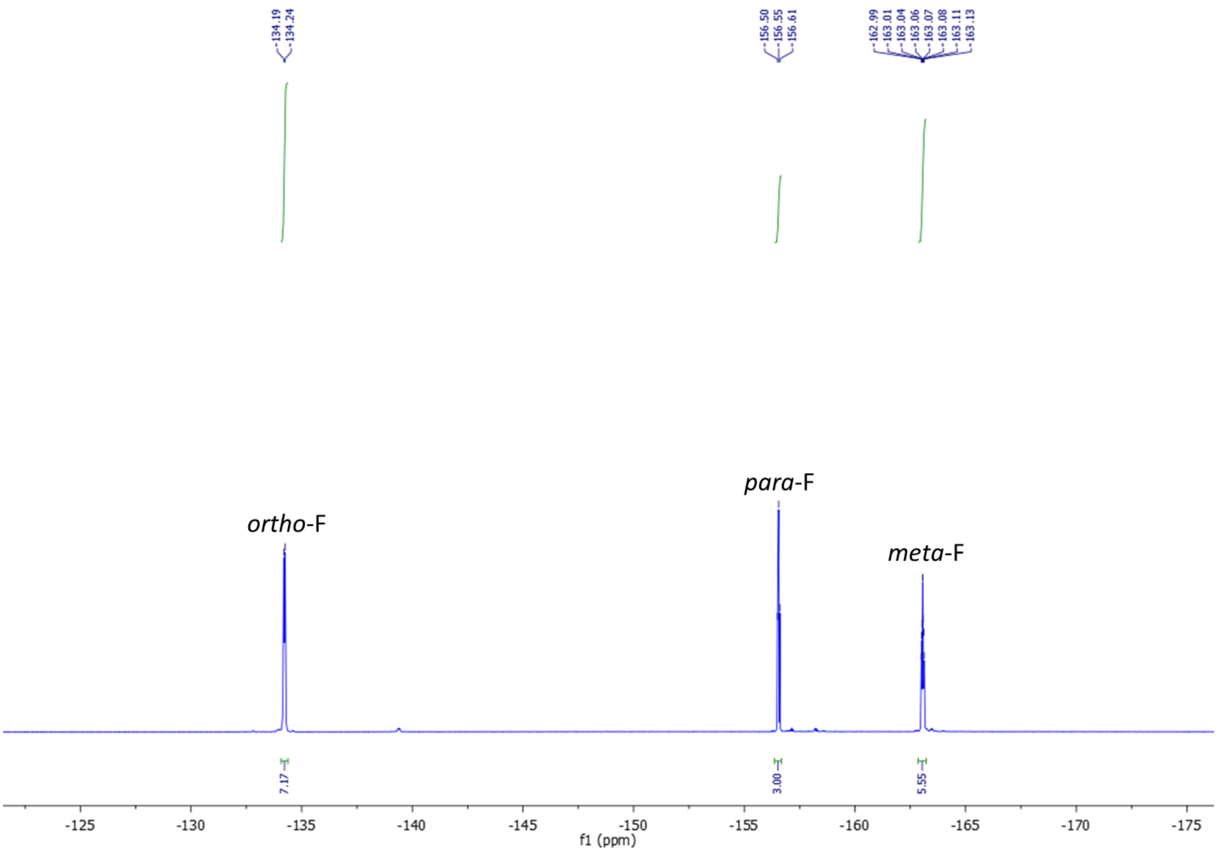

Figure SI 115: ^{19}F NMR spectrum of the analytical sample of 3-(4-(*tert*-butyl)phenyl)pent-4-en-2-imine tris(perfluorophenyl)borane complex **16** (377 MHz, Chloroform-*d*).

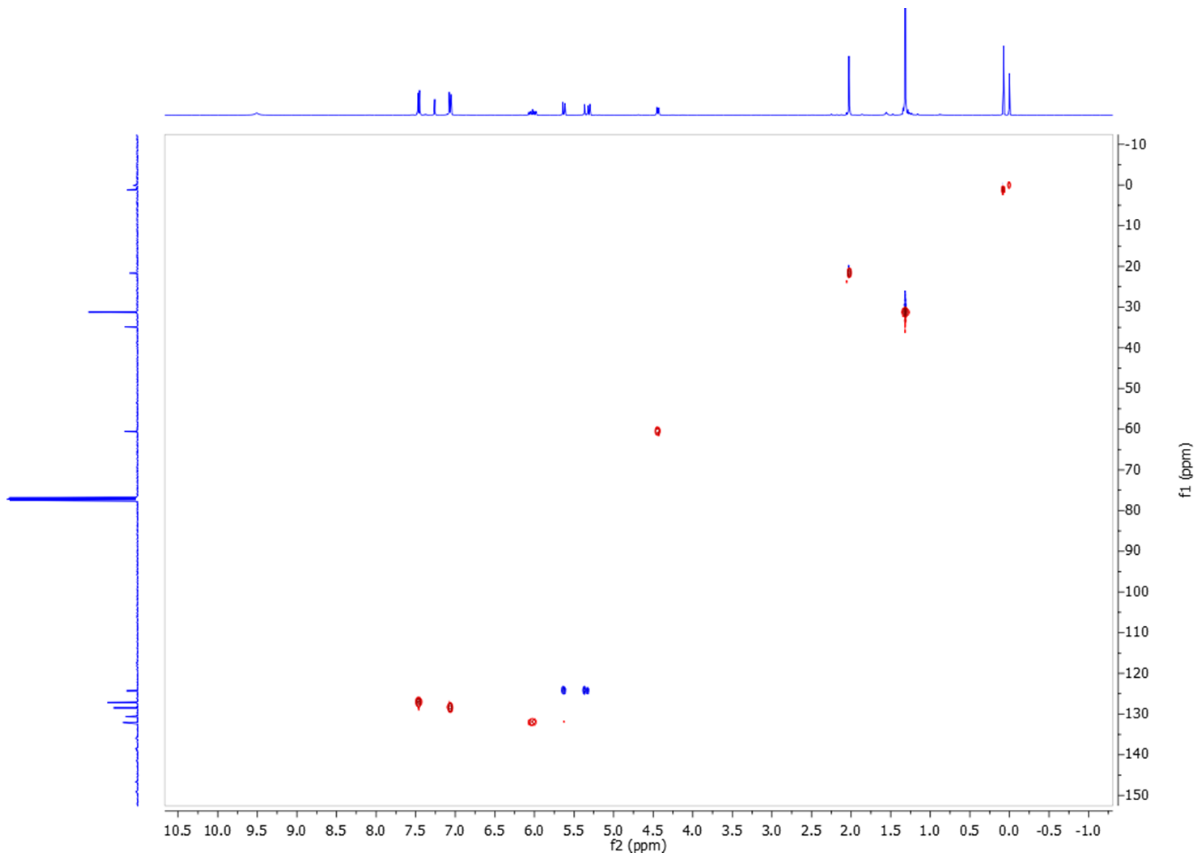

Figure SI 116: HSQC NMR spectrum of the analytical sample of 3-(4-(*tert*-butyl)phenyl)pent-4-en-2-imine tris(perfluorophenyl)borane complex **16** (101 MHz, Chloroform-*d*).

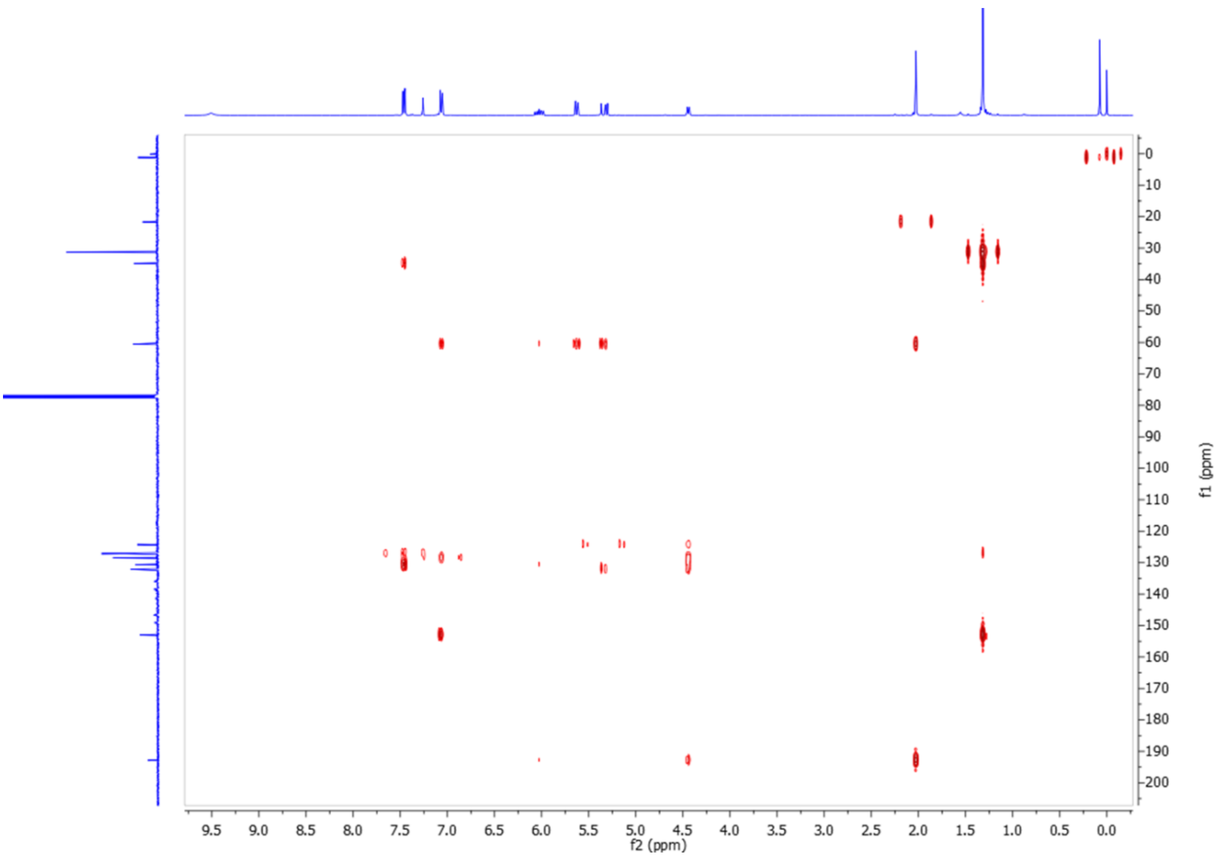

Figure SI 117: HMBC NMR spectrum of the analytical sample of 3-(4-(*tert*-butyl)phenyl)pent-4-en-2-imine tris(perfluorophenyl)borane complex **16** (101 MHz, Chloroform-*d*).

5.4.8 (*E*)-3-(4-(tert-butyl)phenyl)pent-3-en-2-imine tris(perfluorophenyl)borane complex 16'

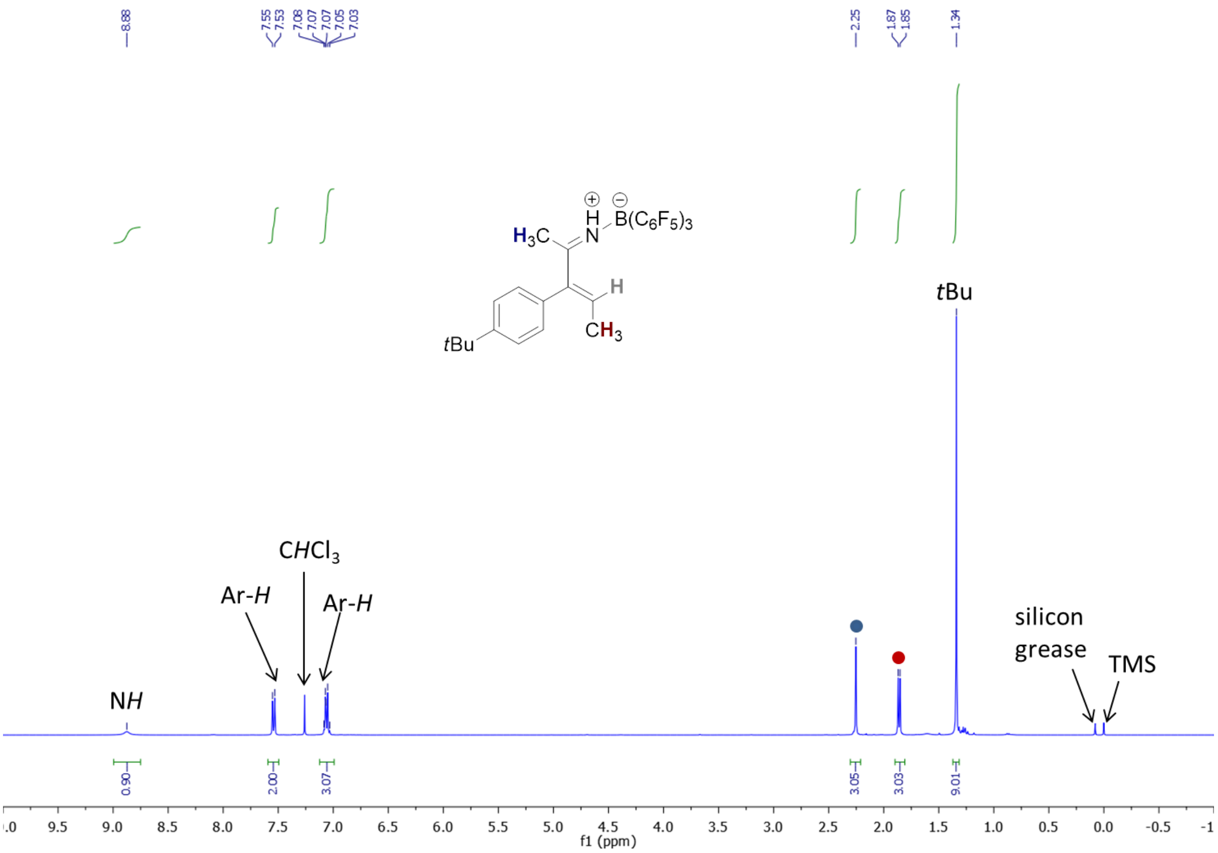

Figure SI 118: ^{1}H NMR spectrum of the analytical sample of the (*E*)-3-(4-(*tert*-butyl)phenyl)pent-3-en-2-imine tris(perfluorophenyl)borane complex **16'** (400 MHz, Chloroform-*d*).

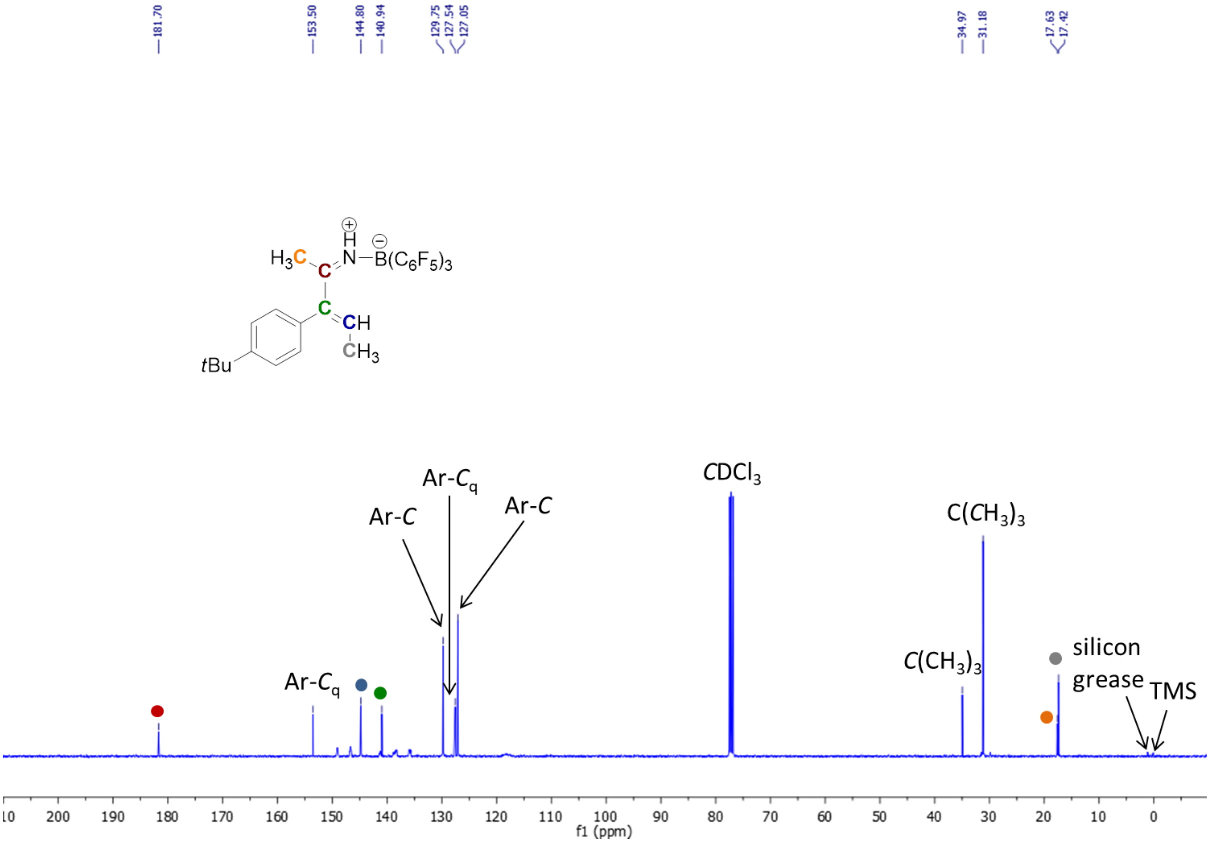

Figure SI 119: $^{13}C\{^1H\}$ NMR spectrum of the analytical sample of the (*E*)-3-(4-(*tert*-butyl)phenyl)pent-3-en-2-imine tris(perfluorophenyl)borane complex **16'** (101 MHz, Chloroform-*d*).

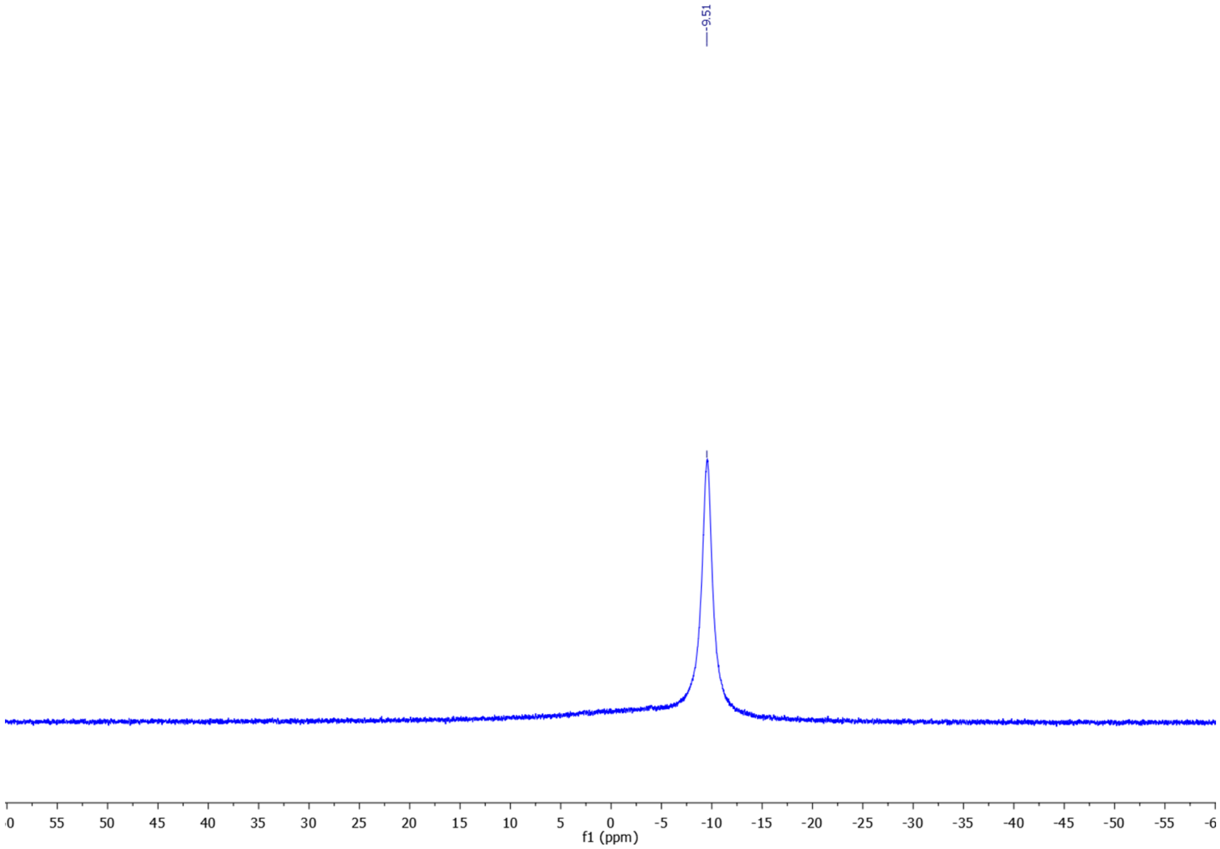

Figure SI 120: ^{11}B NMR spectrum of the analytical sample of the (*E*)-3-(4-(*tert*-butyl)phenyl)pent-3-en-2-imine tris(perfluorophenyl)borane complex **16’** (128 MHz, Chloroform-*d*).

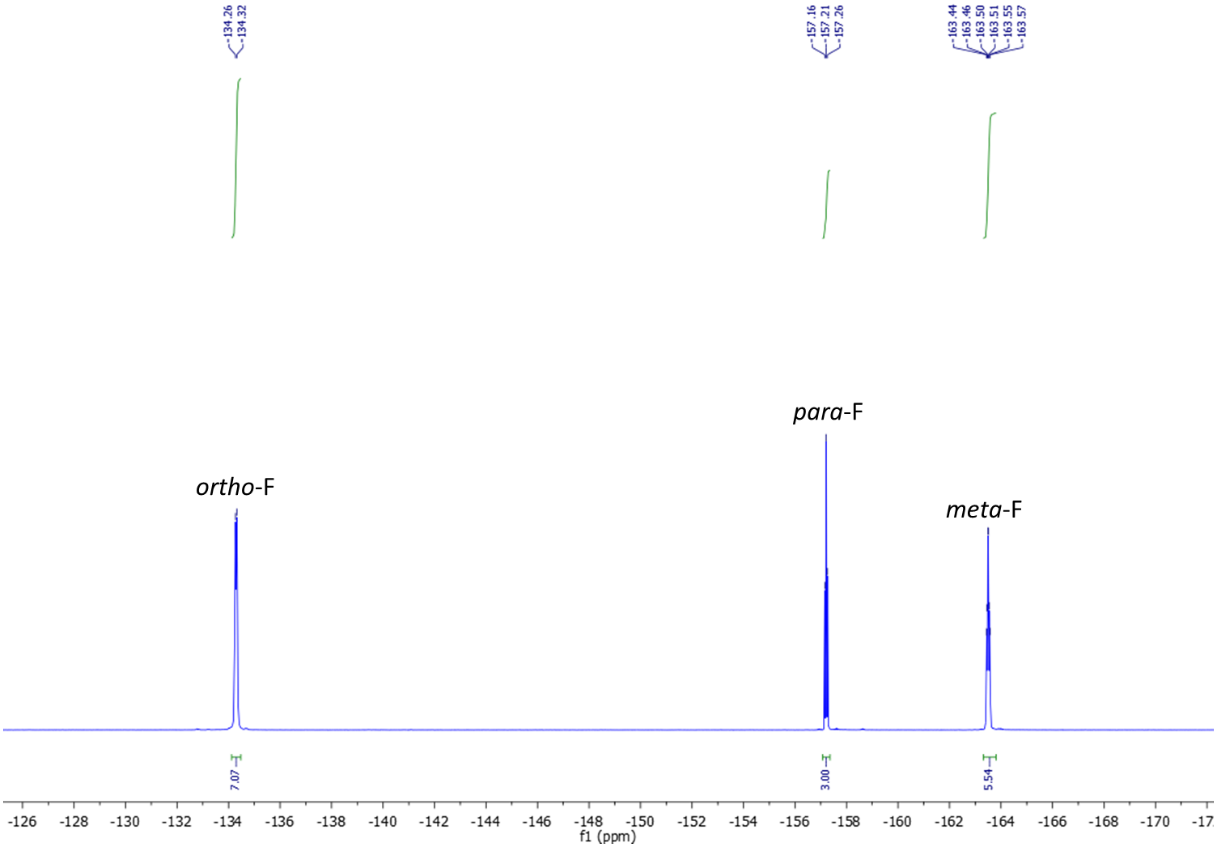

Figure SI 121: ^{19}F NMR spectrum of the analytical sample of the (*E*)-3-(4-(*tert*-butyl)phenyl)pent-3-en-2-imine tris(perfluorophenyl)borane complex **16'** (377 MHz, Chloroform-*d*).

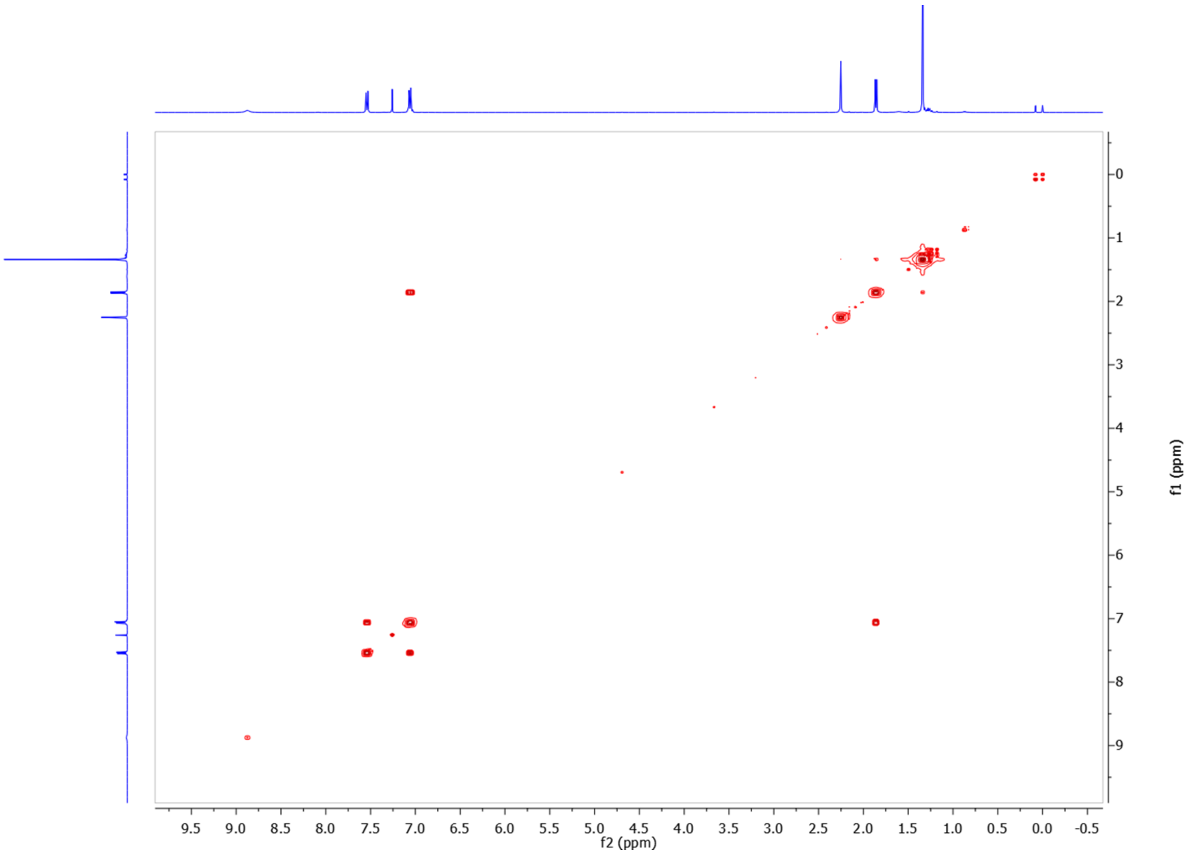

Figure SI 122: HH COSY NMR spectrum of the analytical sample of the (*E*)-3-(4-(*tert*-butyl)phenyl)pent-3-en-2-imine tris(perfluorophenyl)borane complex **16’** (400 MHz, Chloroform-*d*).

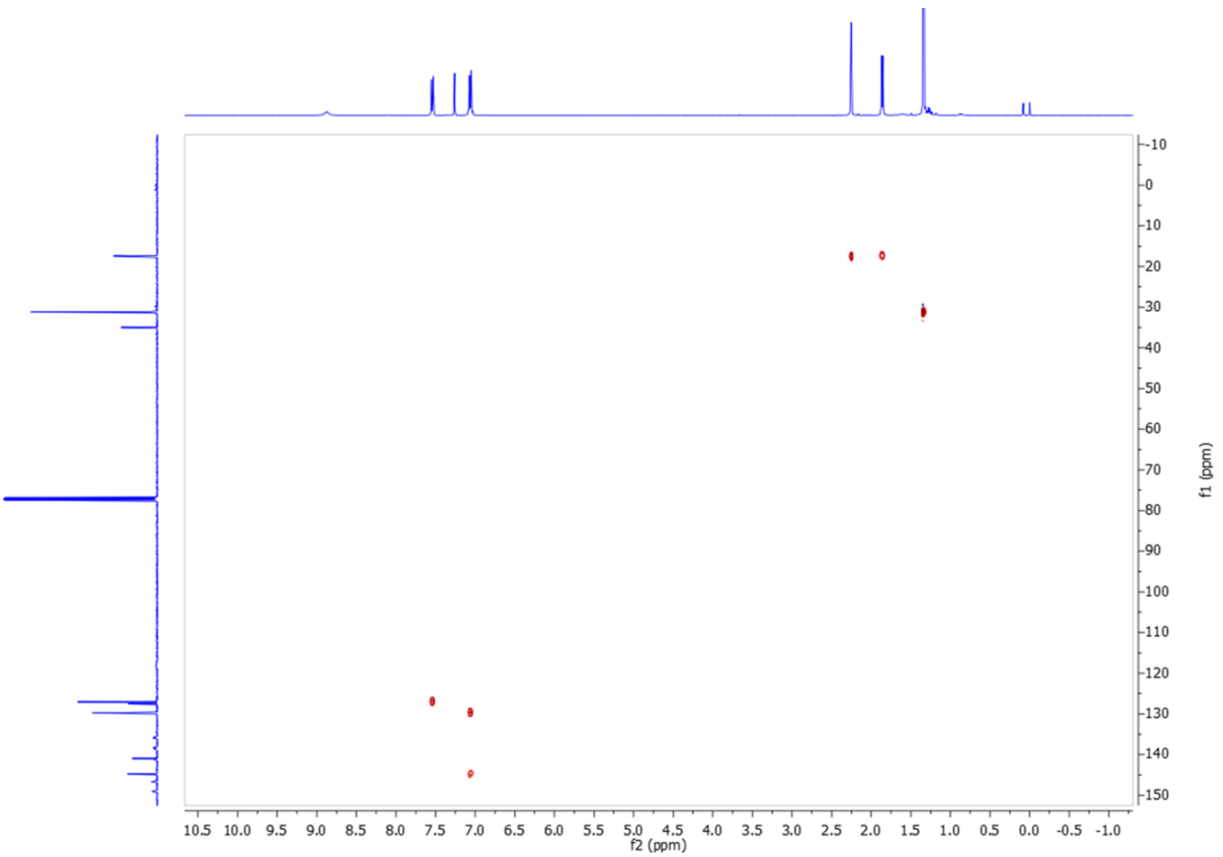

Figure SI 123: HSQC NMR spectrum of the analytical sample of the (*E*)-3-(4-(*tert*-butyl)phenyl)pent-3-en-2-imine tris(perfluorophenyl)borane complex **16’** (101 MHz, Chloroform-*d*).

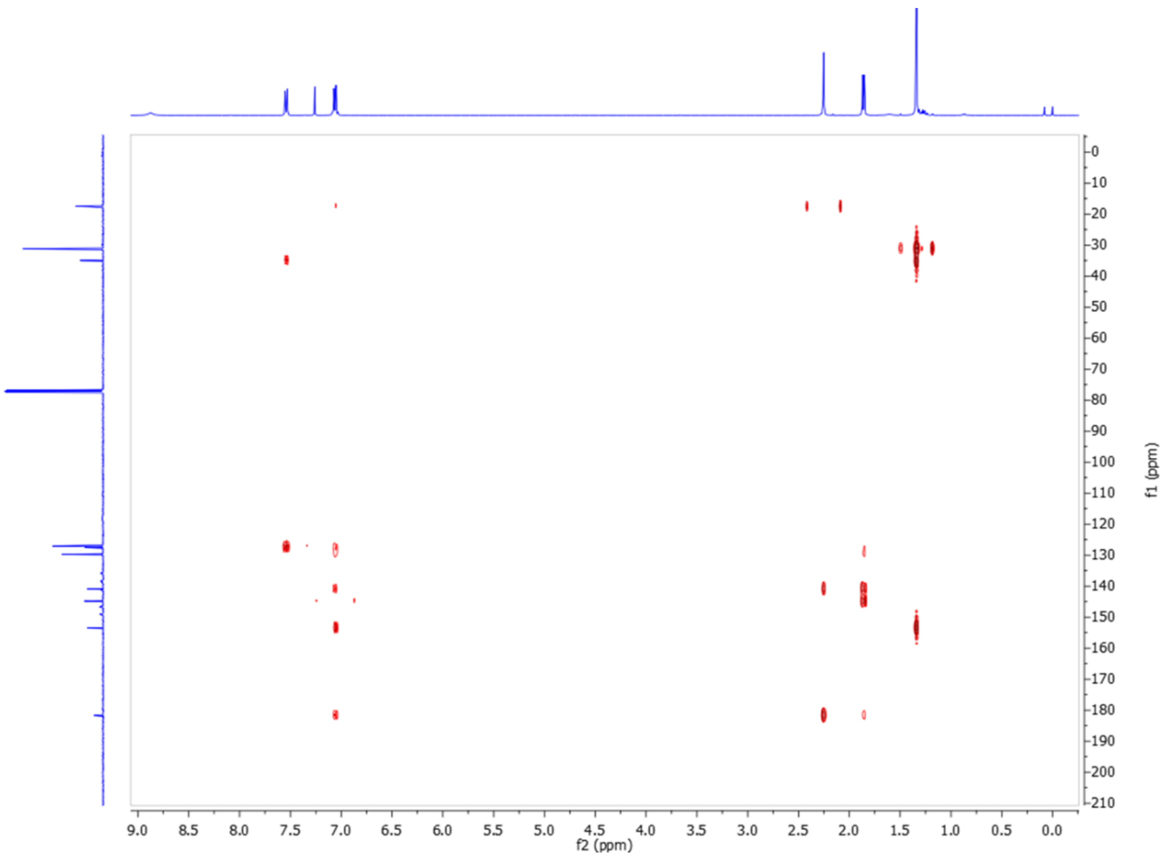

Figure SI 124: HMBC NMR spectrum of the analytical sample of the (*E*)-3-(4-(*tert*-butyl)phenyl)pent-3-en-2-imine tris(perfluorophenyl)borane complex **16’** (101 MHz, Chloroform-*d*).

5.4.9 3-cyclohexylpent-4-en-2-imine tris(perfluorophenyl)borane complex 17

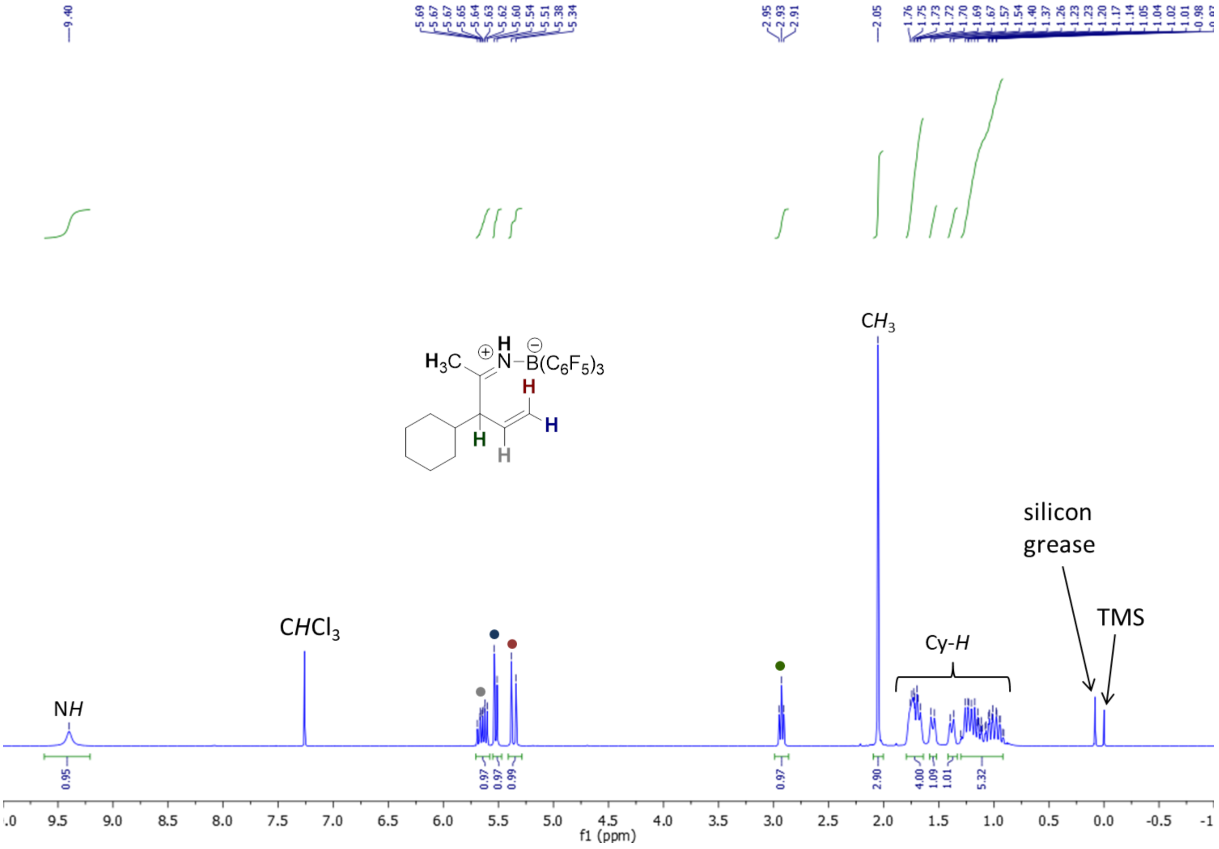

Figure SI 125: ^{1}H NMR spectrum of the analytical sample of the 3-cyclohexylpent-4-en-2-imine tris(perfluorophenyl)borane complex **17** (400 MHz, Chloroform-*d*).

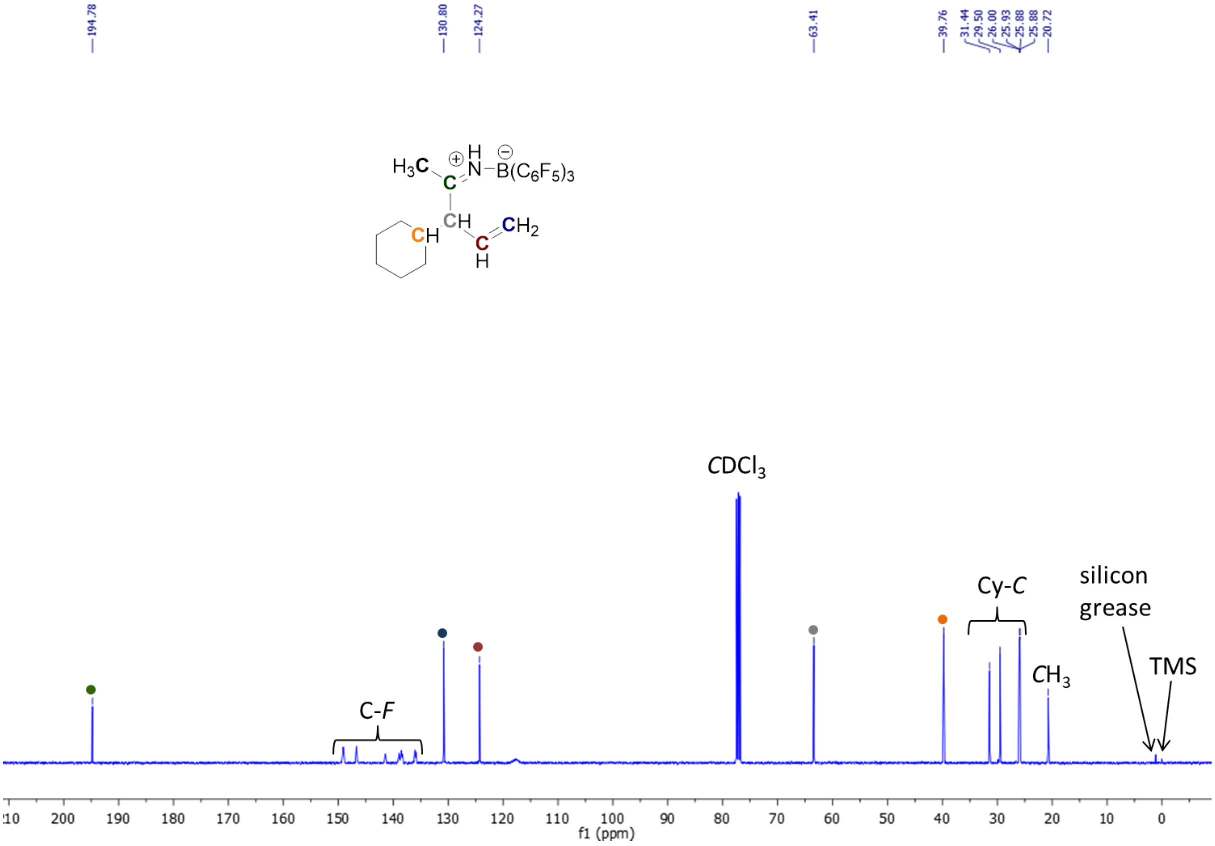

Figure SI 126: $^{13}C\{^1H\}$ NMR spectrum of the analytical sample of the 3-cyclohexylpent-4-en-2-imine tris(perfluorophenyl)borane complex **17** (101 MHz, Chloroform-*d*).

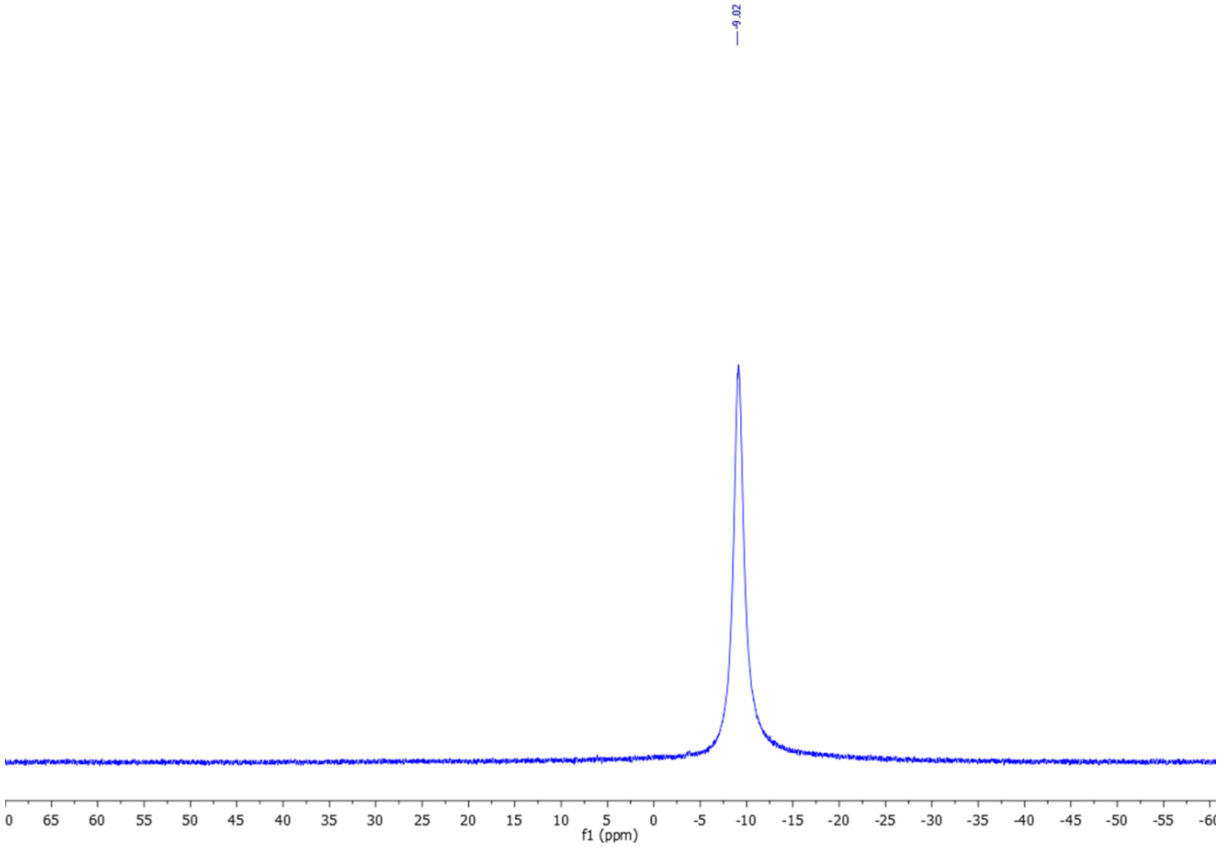

Figure SI 127: ^{11}B NMR spectrum of the analytical sample of the 3-cyclohexylpent-4-en-2-imine tris(perfluorophenyl)borane complex **17** (128 MHz, Chloroform-*d*).

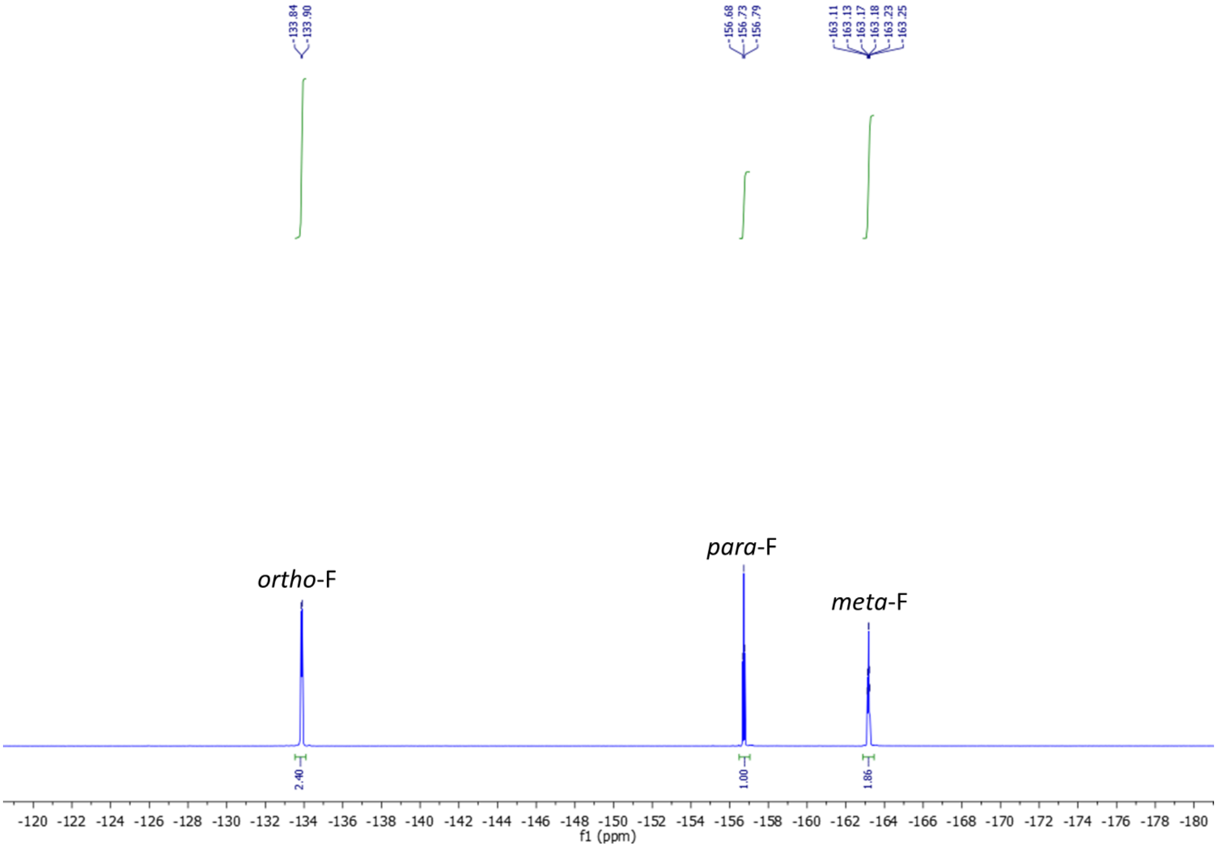

Figure SI 128: ^{19}F NMR spectrum of the analytical sample of the 3-cyclohexylpent-4-en-2-imine tris(perfluorophenyl)borane complex **17** (377 MHz, Chloroform-*d*).

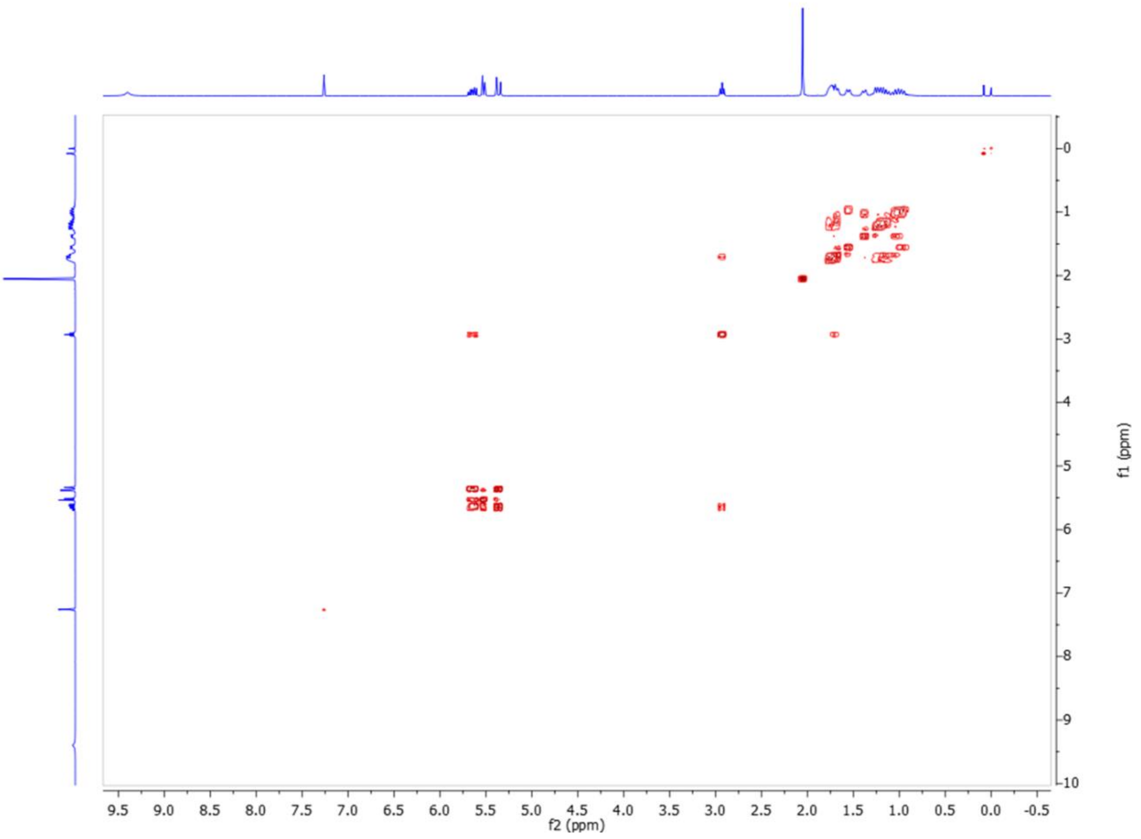

Figure SI 129: COSY NMR spectrum of the analytical sample of the 3-cyclohexylpent-4-en-2-imine tris(perfluorophenyl)borane complex **17** (400 MHz, Chloroform-*d*).

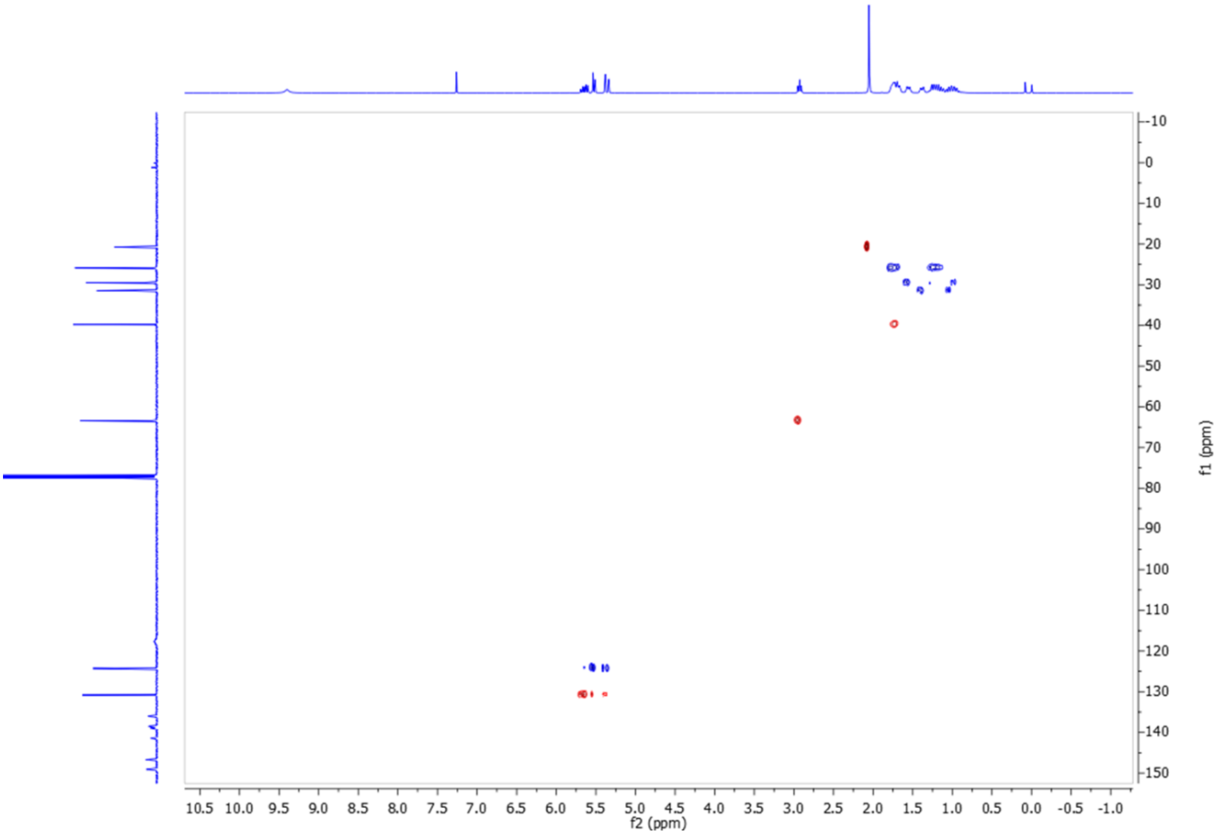

Figure SI 130: HSQC NMR spectrum of the analytical sample of the 3-cyclohexylpent-4-en-2-imine tris(perfluorophenyl)borane complex **17** (101 MHz, Chloroform-*d*).

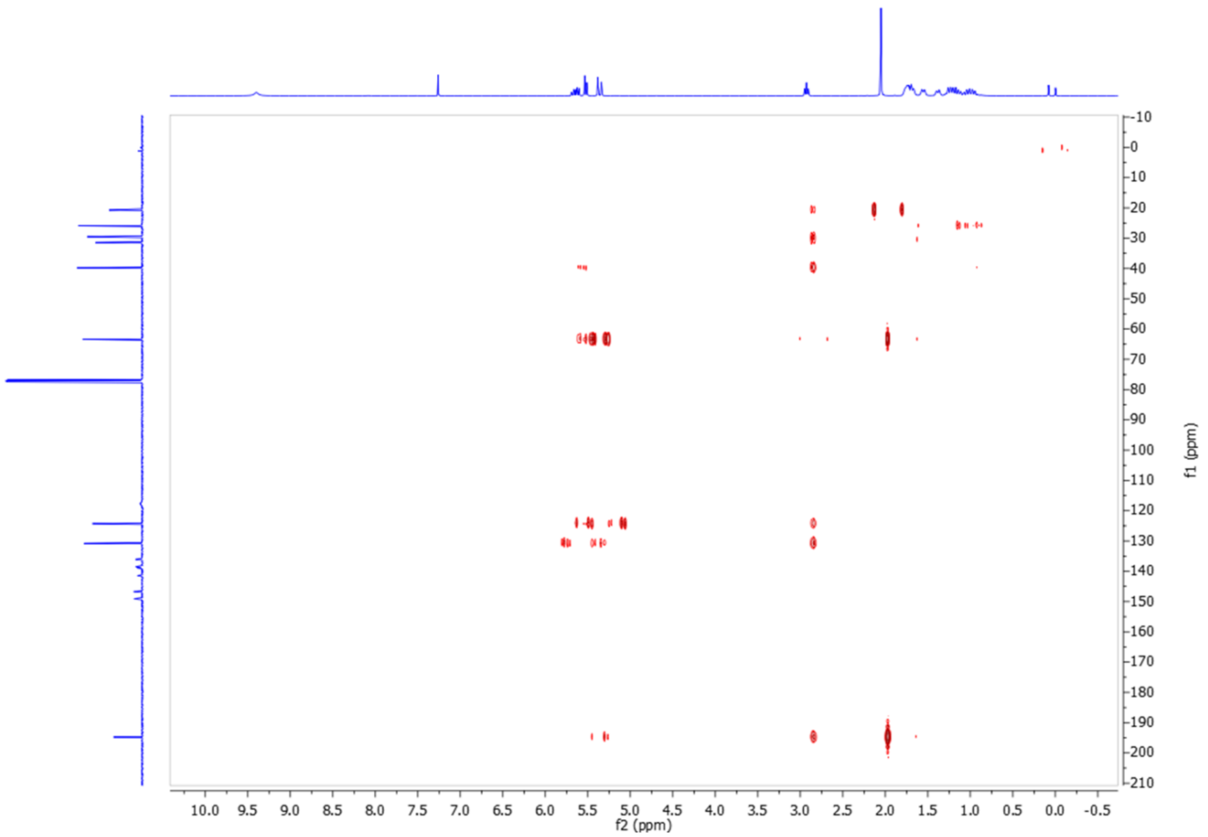

Figure SI 131: HMBC NMR spectrum of the analytical sample of the 3-cyclohexylpent-4-en-2-imine tris(perfluorophenyl)borane complex **17** (101 MHz, Chloroform-*d*).

5.4.10 3-vinylnonan-2-imine tris(perfluorophenyl)borane complex 18

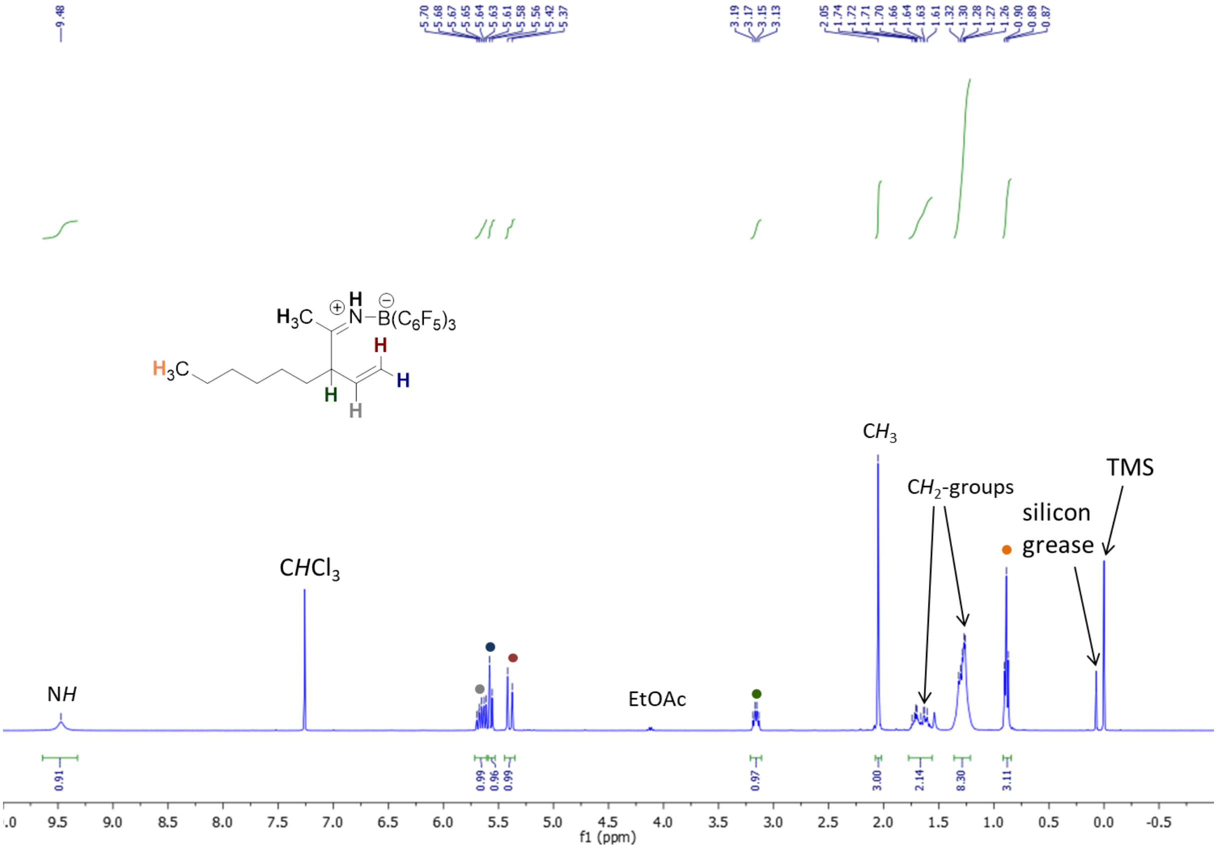

Figure SI 132: ^{1}H NMR spectrum of the analytical sample of the 3-vinylnonan-2-imine tris(perfluorophenyl)borane complex **18** (400 MHz, Chloroform-*d*).

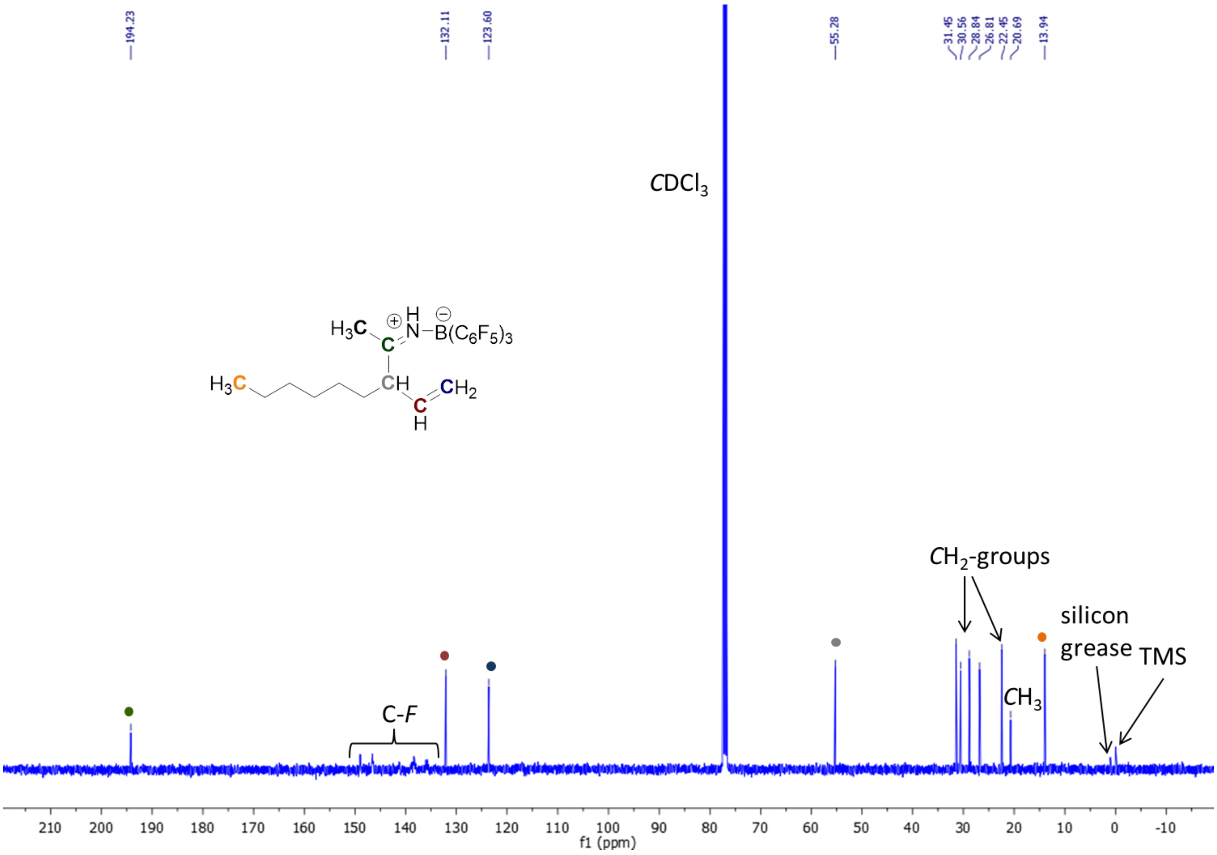

Figure SI 133: $^{13}C\{^1H\}$ NMR spectrum of the analytical sample of the 3-vinylnonan-2-imine tris(perfluorophenyl)borane complex **18** (101 MHz, Chloroform-*d*).

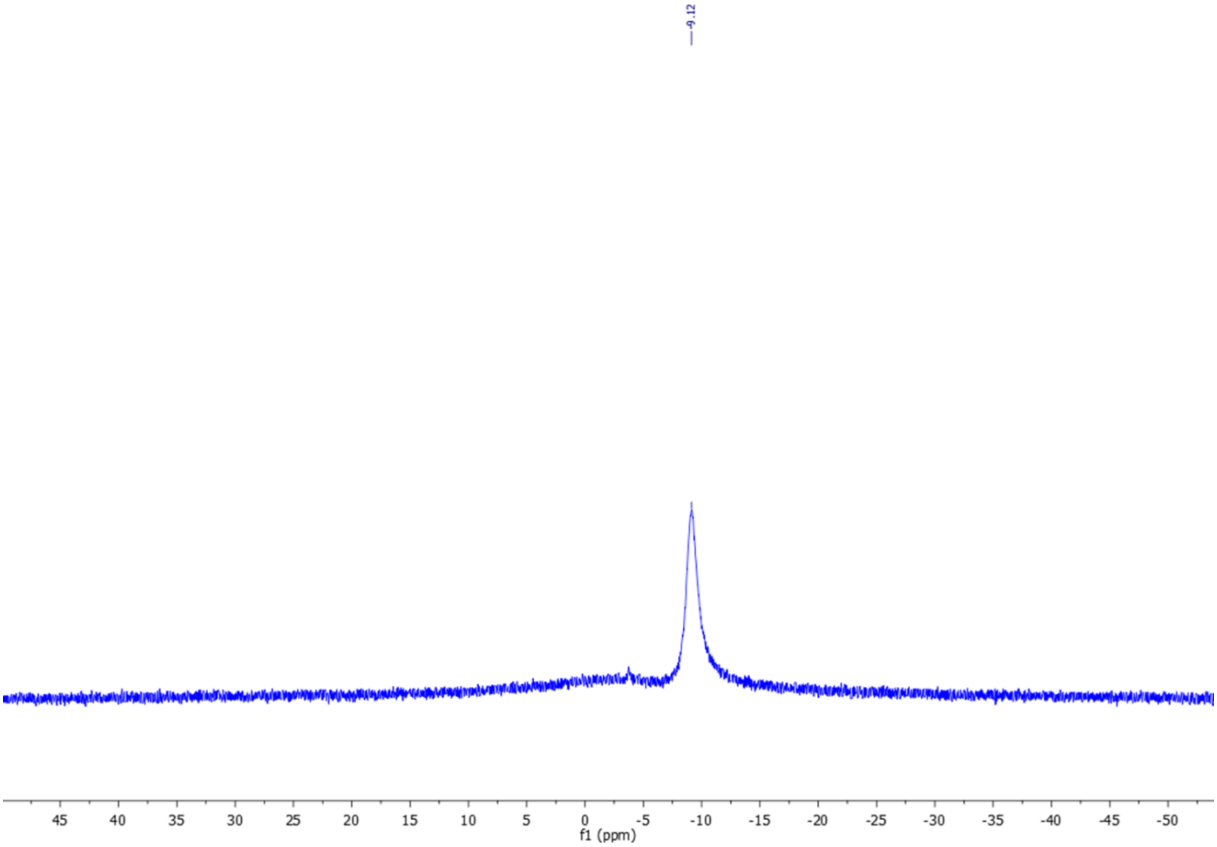

Figure SI 134: ^{11}B NMR spectrum of the analytical sample of the 3-vinylnonan-2-imine tris(perfluorophenyl)borane complex **18** (128 MHz, Chloroform-*d*).

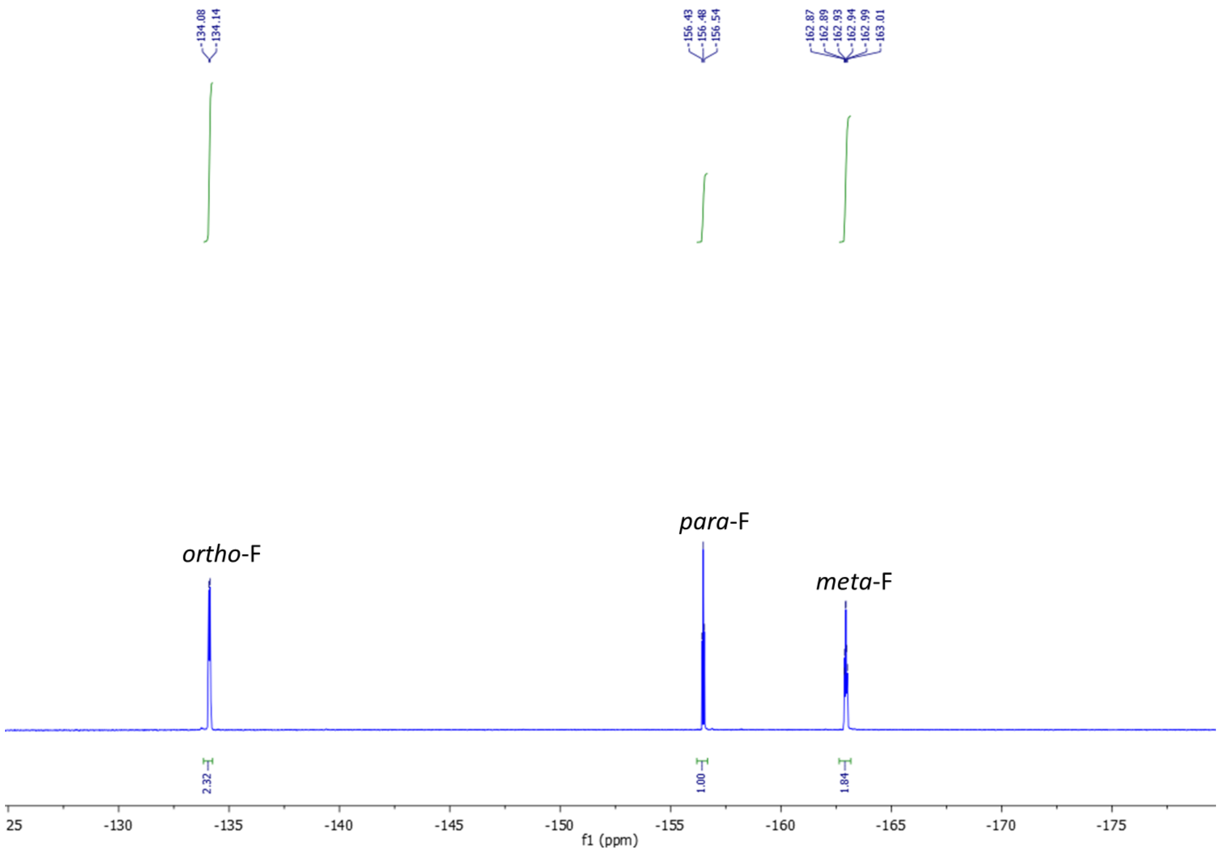

Figure SI 135: ^{19}F NMR spectrum of the analytical sample of the 3-vinylnonan-2-imine tris(perfluorophenyl)borane complex **18** (377 MHz, Chloroform-*d*).

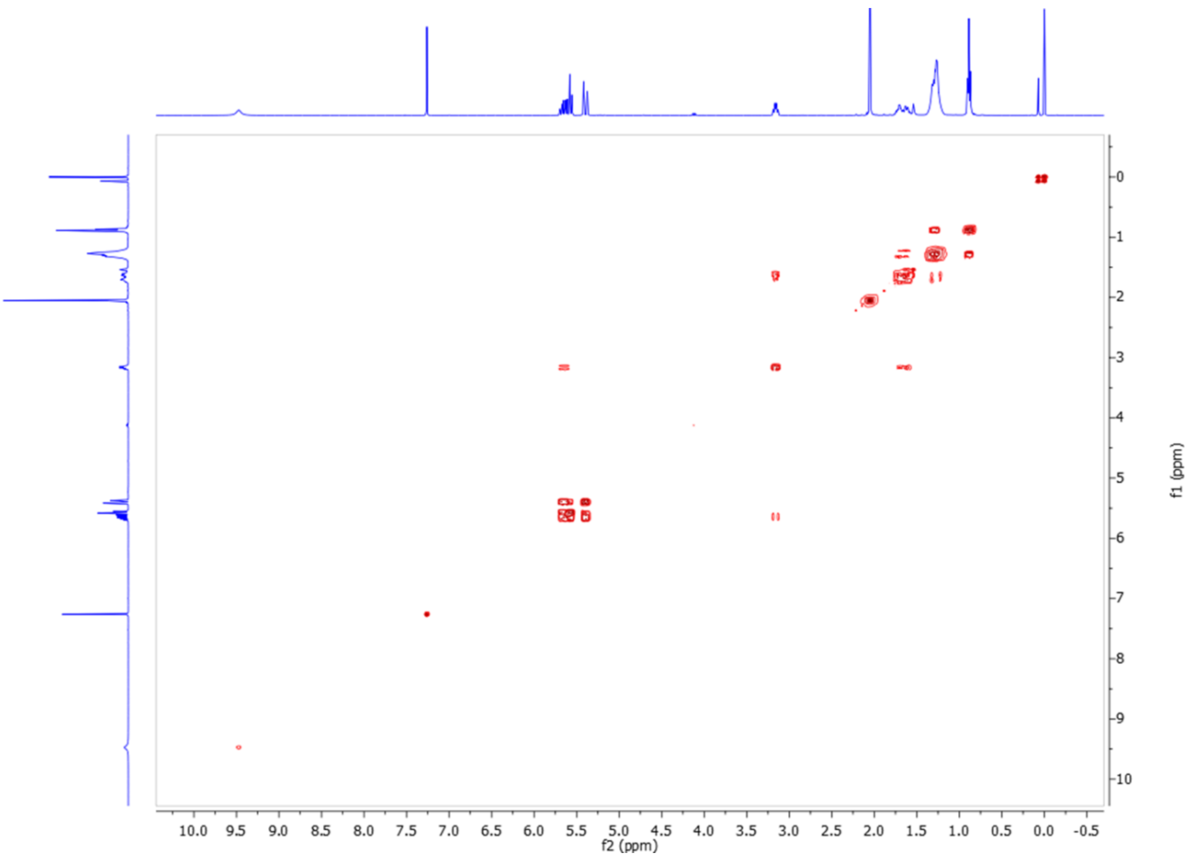

Figure SI 136: COSY NMR spectrum of the analytical sample of the 3-vinylnonan-2-imine tris(perfluorophenyl)borane complex **18** (400 MHz, Chloroform-*d*).

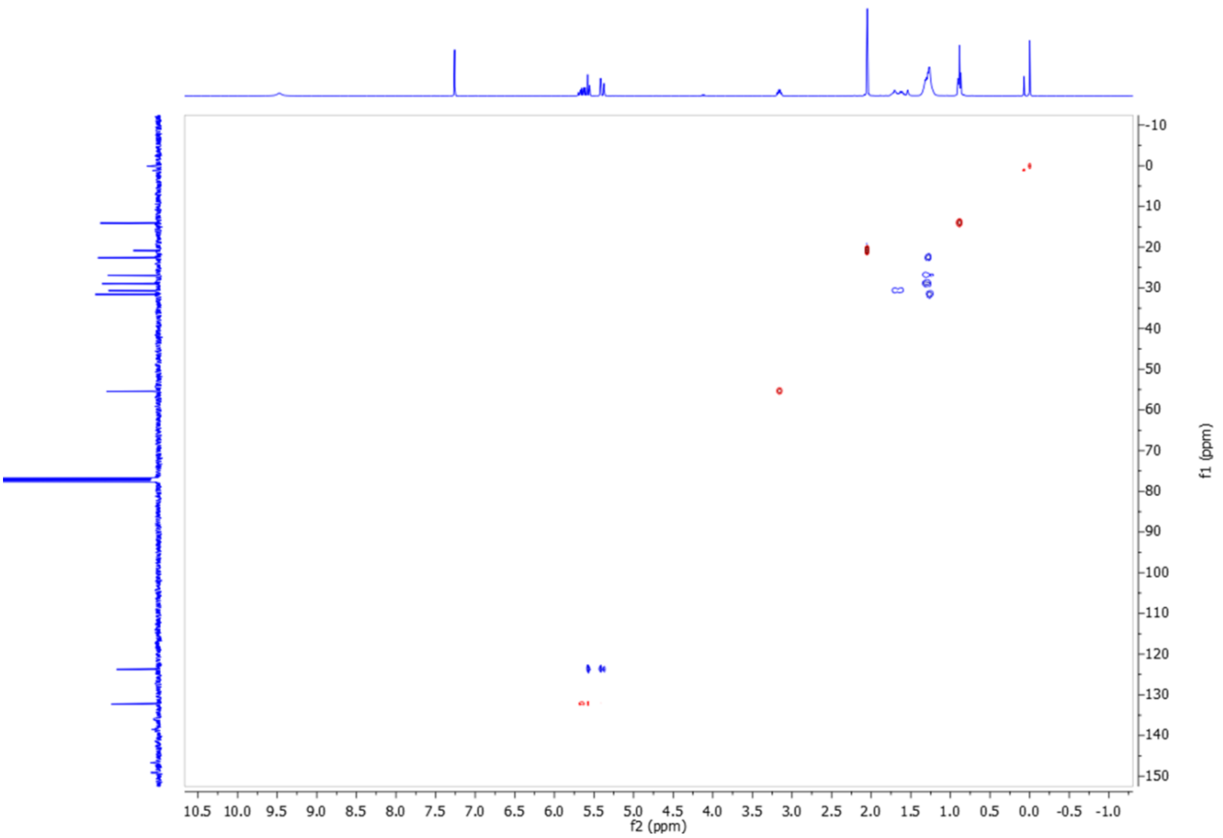

Figure SI 137: HSQC NMR spectrum of the analytical sample of the 3-vinylnonan-2-imine tris(perfluorophenyl)borane complex **18** (101 MHz, Chloroform-*d*).

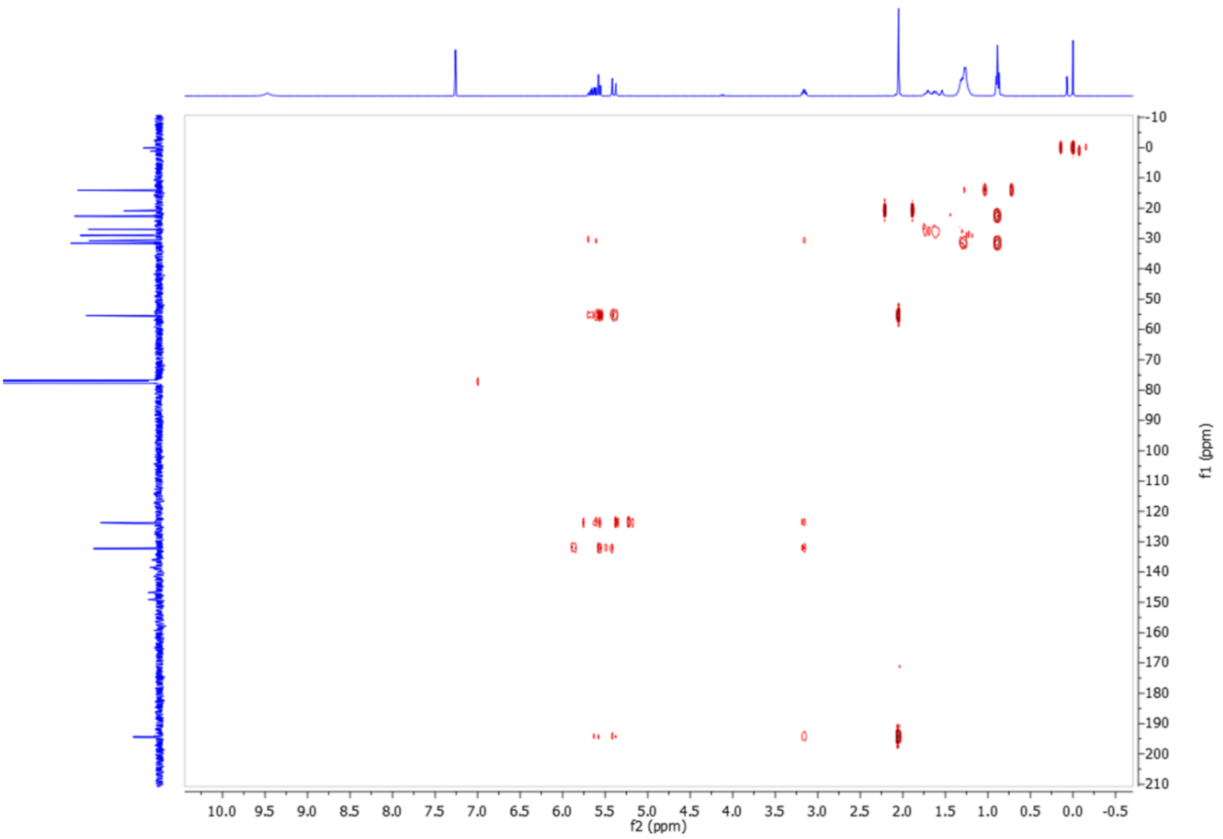

Figure SI 138: HMBC NMR spectrum of the analytical sample of the 3-vinylnonan-2-imine tris(perfluorophenyl)borane complex **18** (101 MHz, Chloroform-*d*).

5.4.11 (*E*)-3-ethylidenenonan-2-imine tris(perfluorophenyl)borane complex 18’

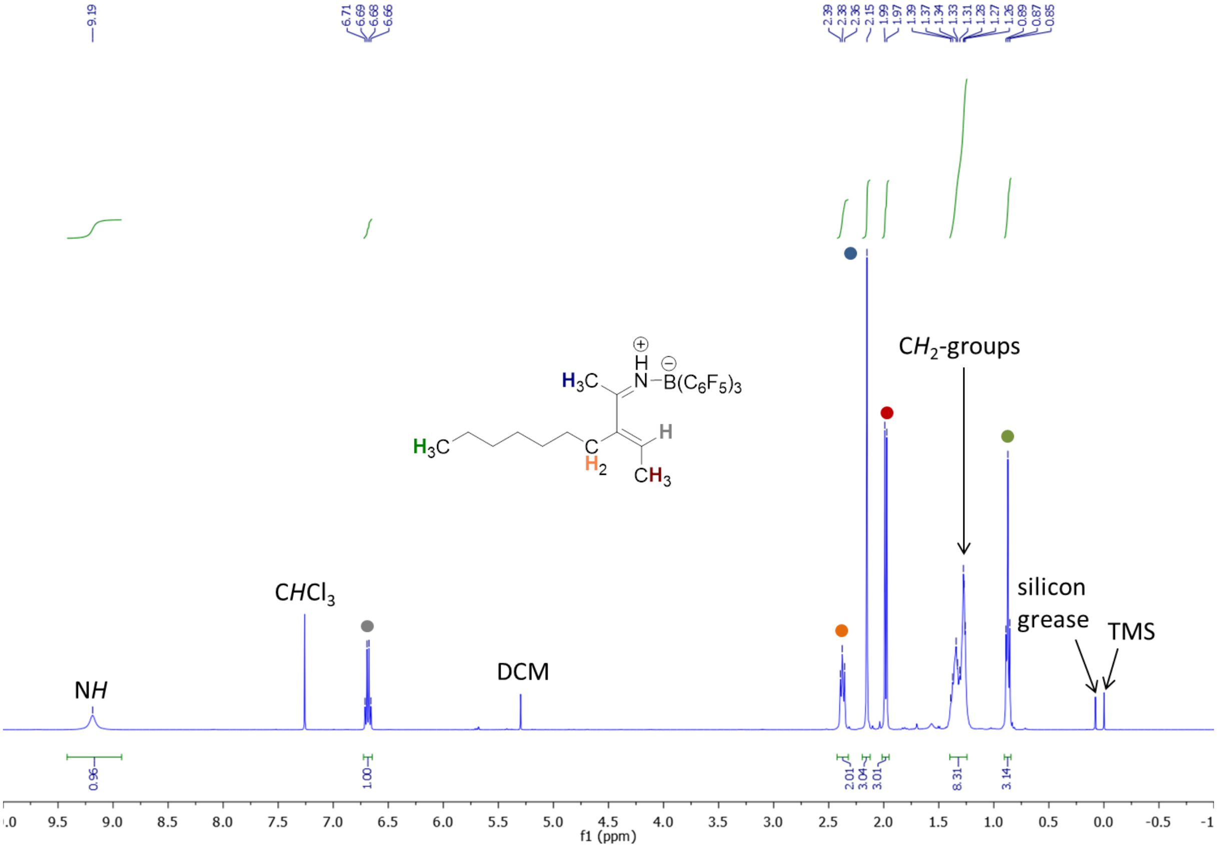

Figure SI 139: ^{1}H NMR spectrum of the analytical sample of the (*E*)-3-ethylidenenonan-2-imine tris(perfluorophenyl)borane complex **18’** (400 MHz, Chloroform-*d*).

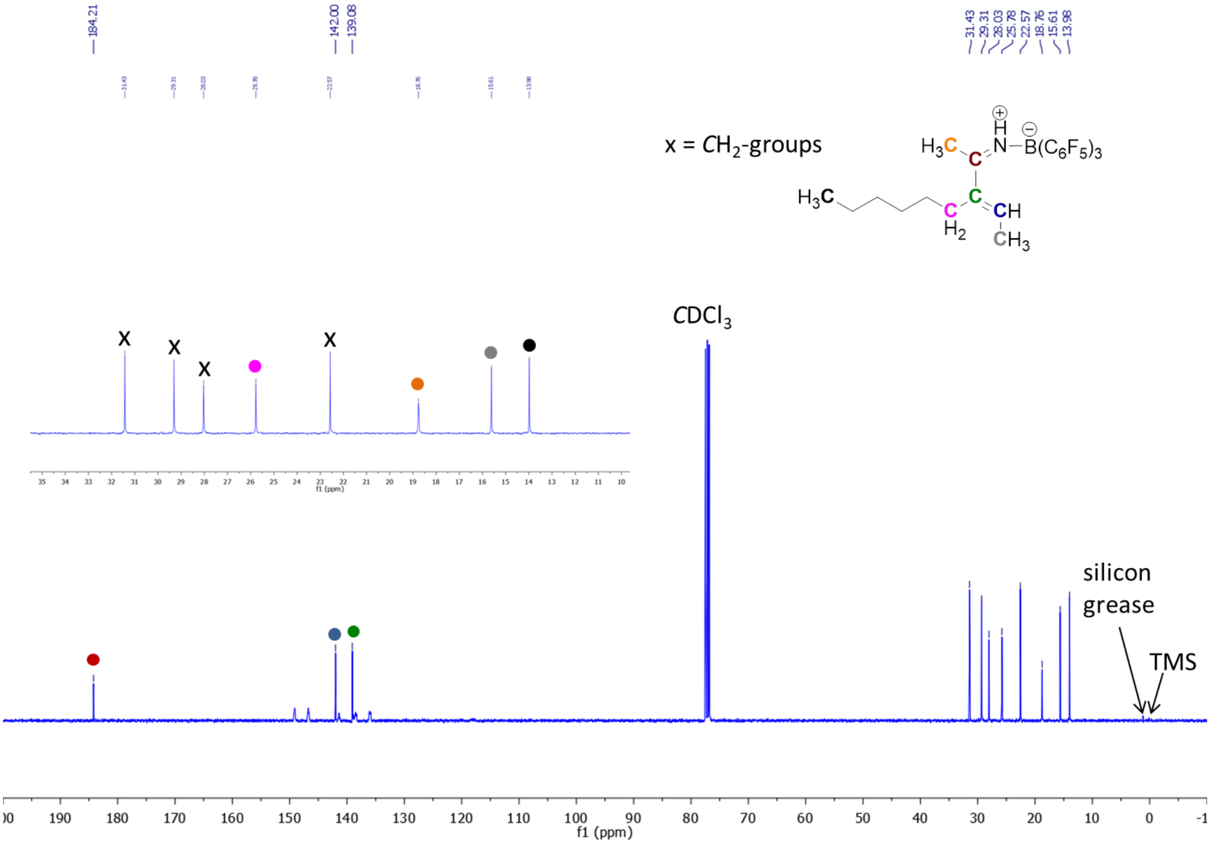

Figure SI 140: $^{13}C\{^{1}H\}$ NMR spectrum of the analytical sample of (*E*)-3-ethylidenenonan-2-imine tris(perfluorophenyl)borane complex **18’** (101 MHz, Chloroform-*d*).

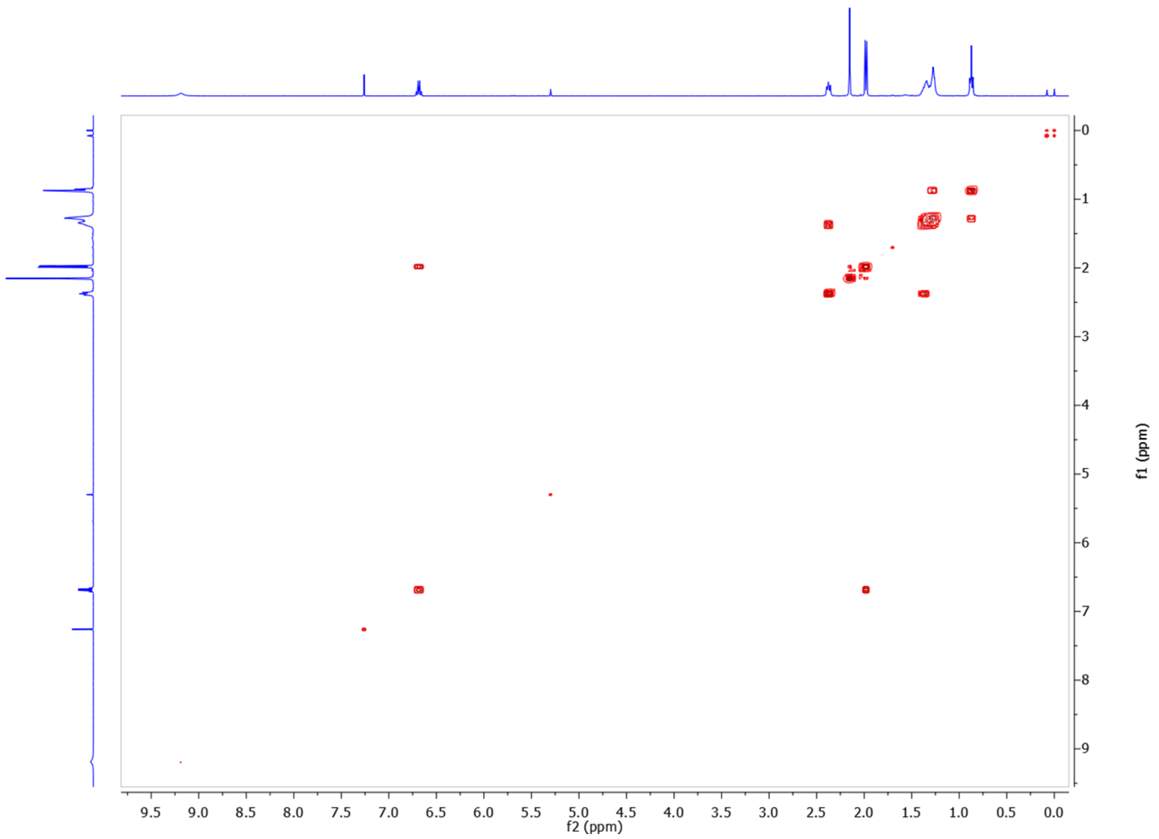

Figure SI 141: COSY NMR spectrum of the analytical sample of the (*E*)-3-ethylidenenonan-2-imine tris(perfluorophenyl)borane complex **18’** (400 MHz, Chloroform-*d*).

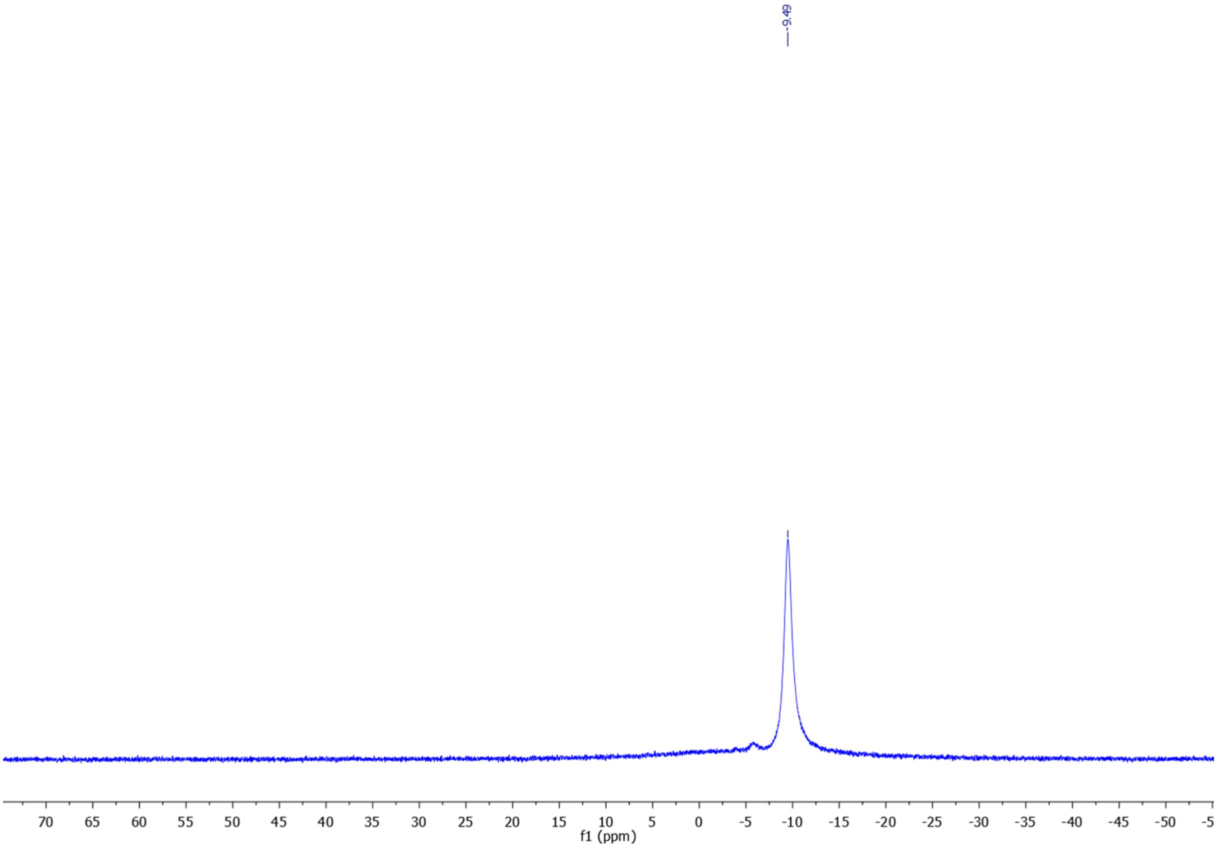

Figure SI 142: ^{11}B NMR spectrum of the analytical sample of the (*E*)-3-ethylidenenonan-2-imine tris(perfluorophenyl)borane complex **18'** (128 MHz, Chloroform-*d*).

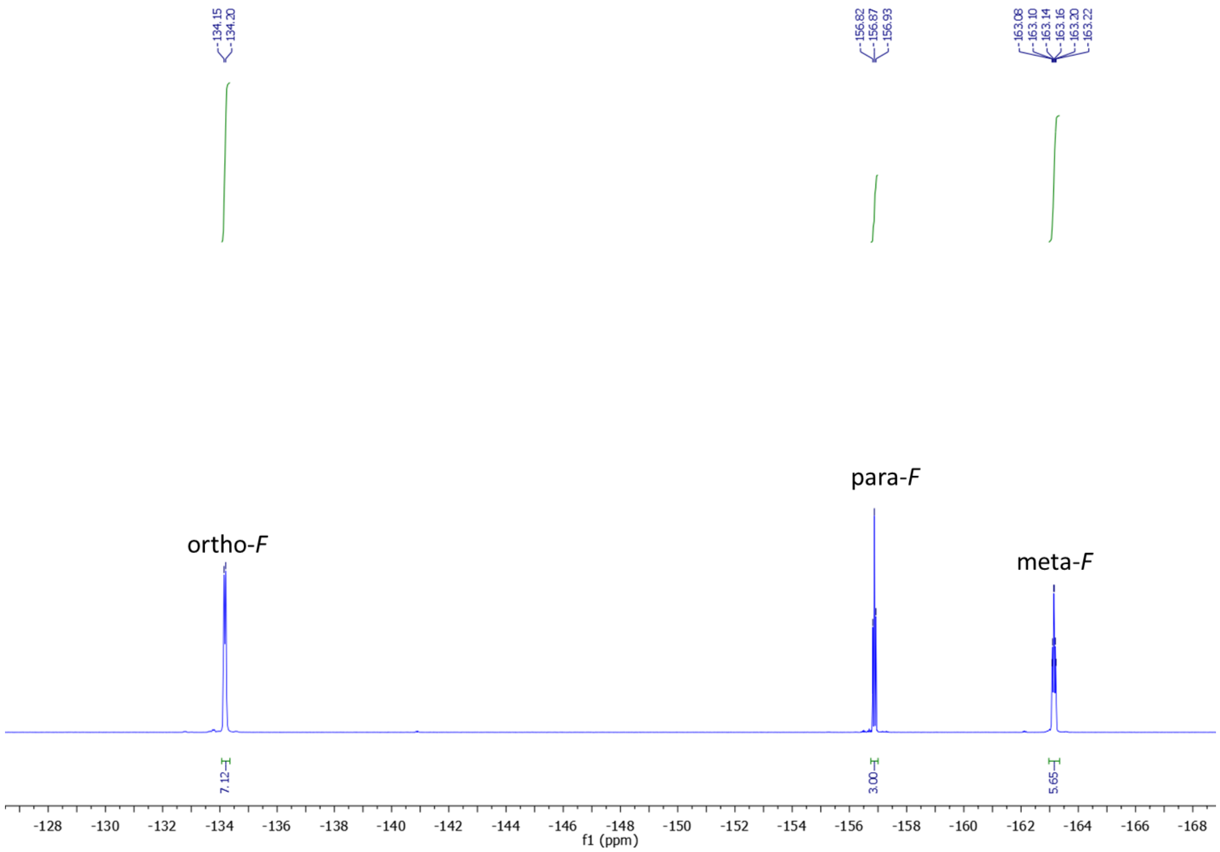

Figure SI 143: ^{19}F NMR spectrum of the analytical sample of the (*E*)-3-ethylidenenonan-2-imine tris(perfluorophenyl)borane complex **18'** (377 MHz, Chloroform-*d*).

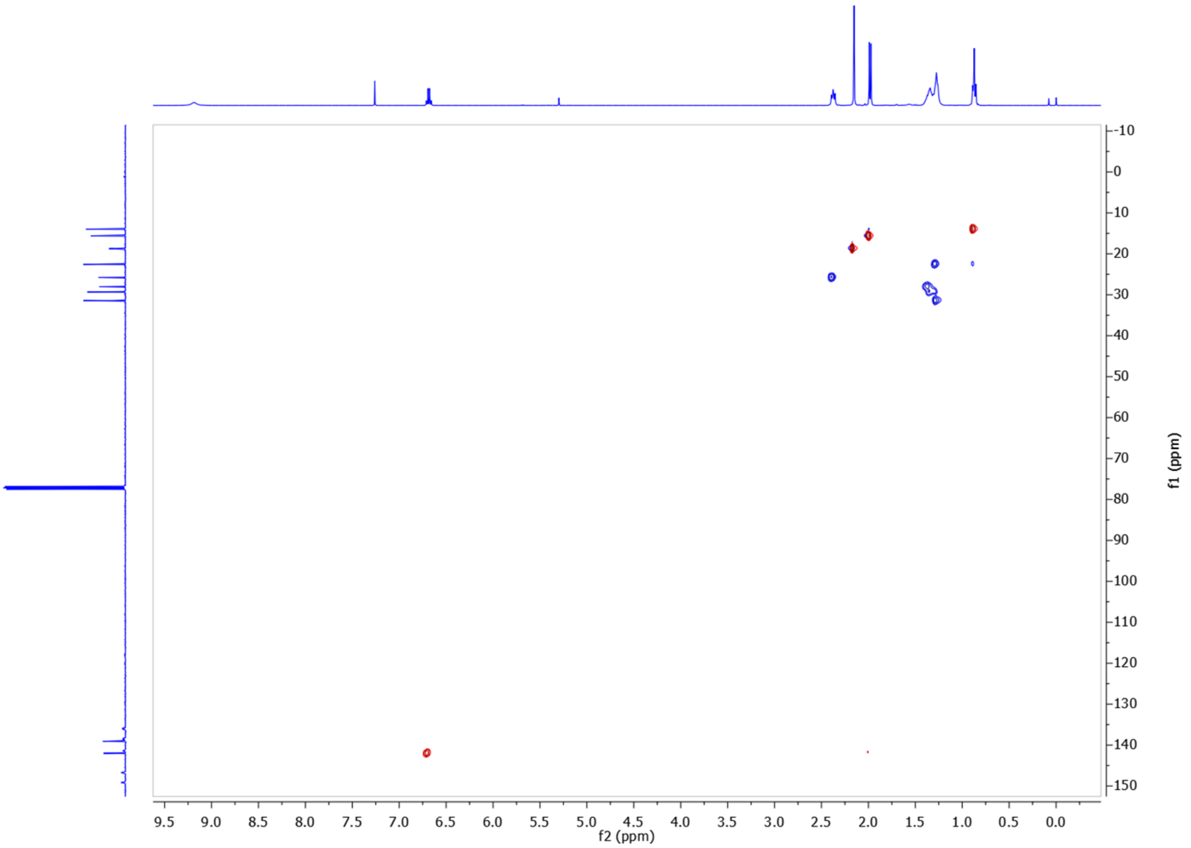

Figure SI 144: HSQC NMR spectrum of the analytical sample of the (*E*)-3-ethylidenenonan-2-imine tris(perfluorophenyl)borane complex **18'** (101 MHz, Chloroform-*d*).

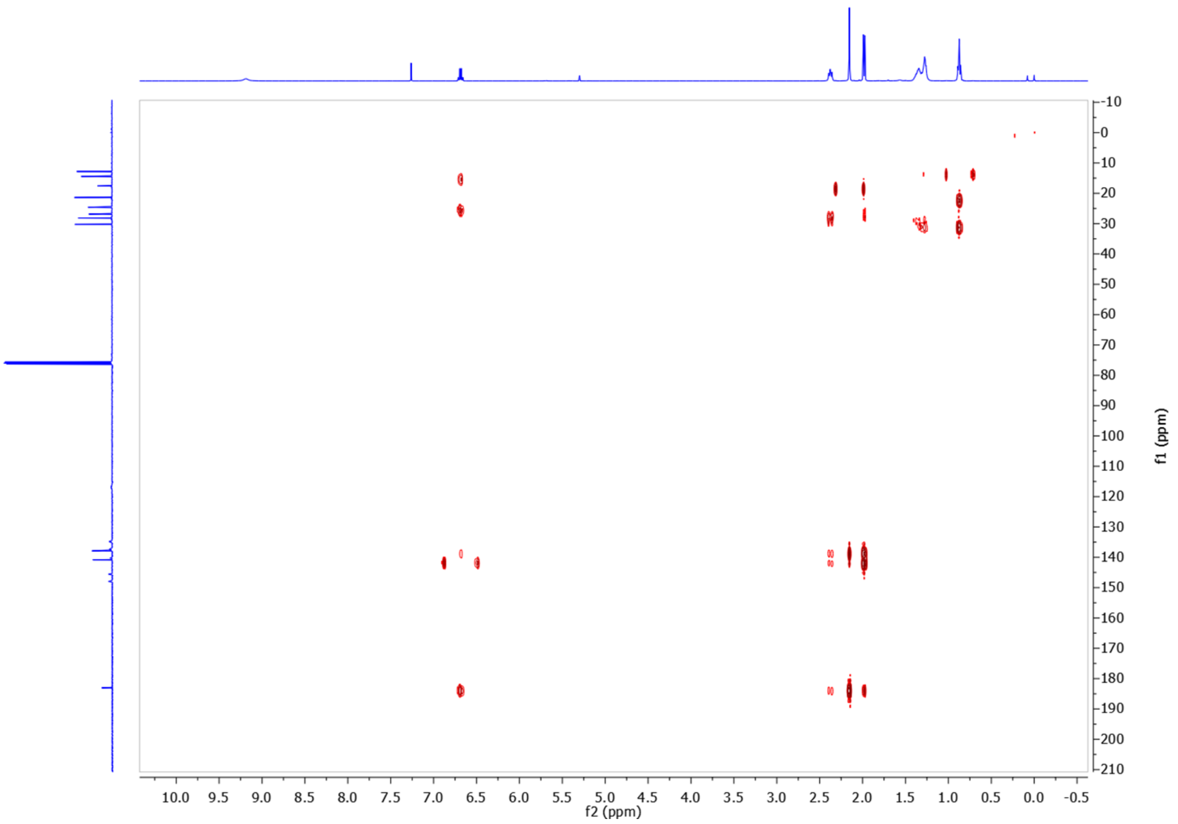

Figure SI 145: HMBC NMR spectrum of the analytical sample the (*E*)-3-ethylidenenonan-2-imine tris(perfluorophenyl)borane complex **18’** (101 MHz, Chloroform-*d*).

5.4.12 3-(adamantan-1-yl)pent-4-en-2-imine tris(perfluorophenyl)borane complex 19

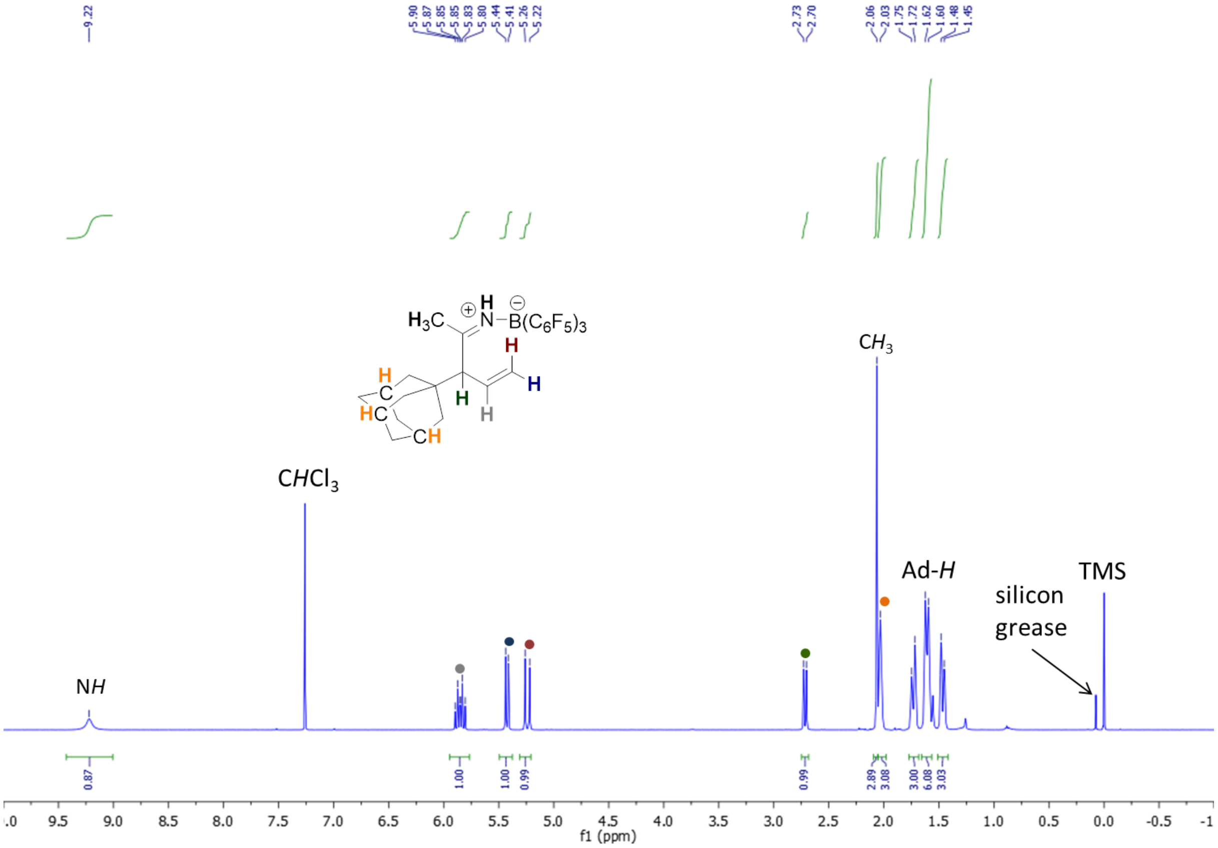

Figure SI 146: ^{1}H NMR of the analytical sample of 3-(adamantan-1-yl)pent-4-en-2-imine tris(perfluorophenyl)borane complex **19** (400 MHz, Chloroform-*d*).

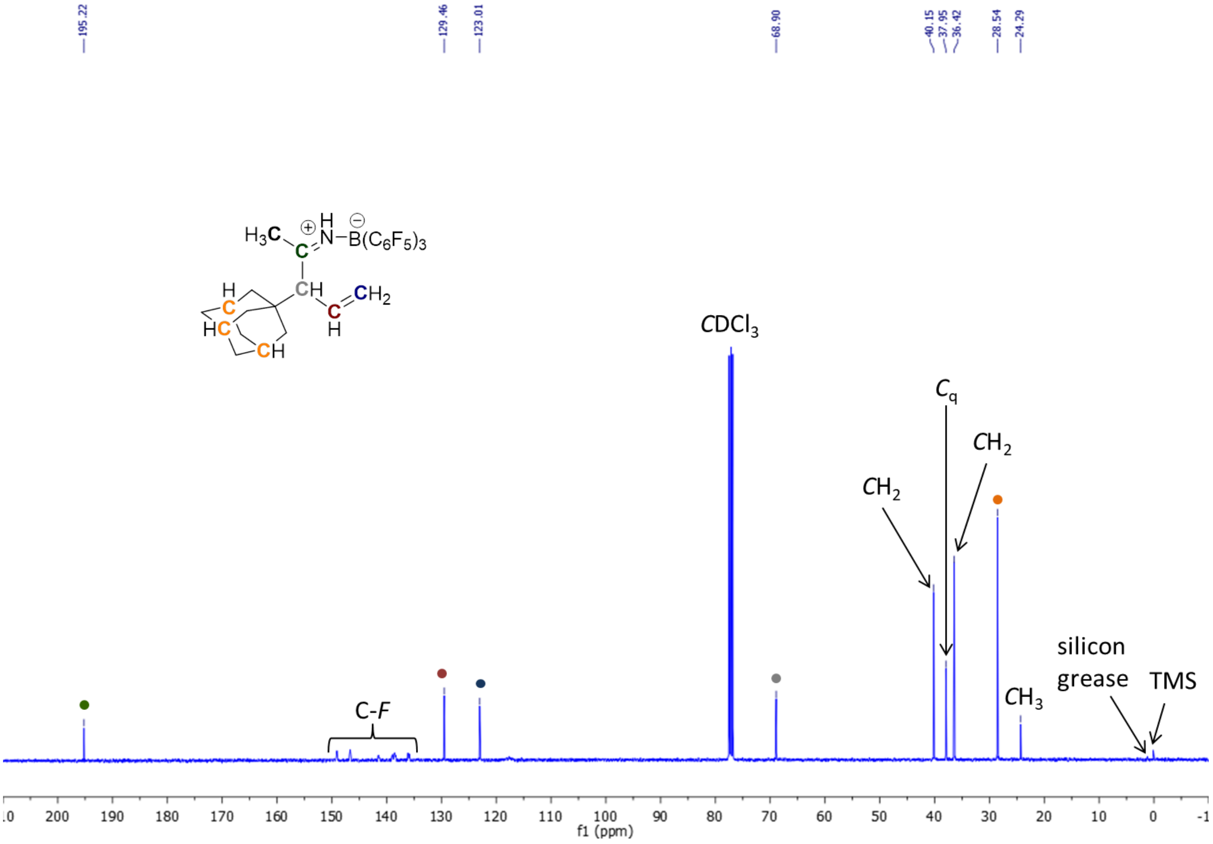

Figure SI 147: $^{13}C\{^{1}H\}$ NMR spectrum of the analytical sample of 3-(adamantan-1-yl)pent-4-en-2-imine tris(perfluorophenyl)borane complex **19** (101 MHz, Chloroform-*d*).

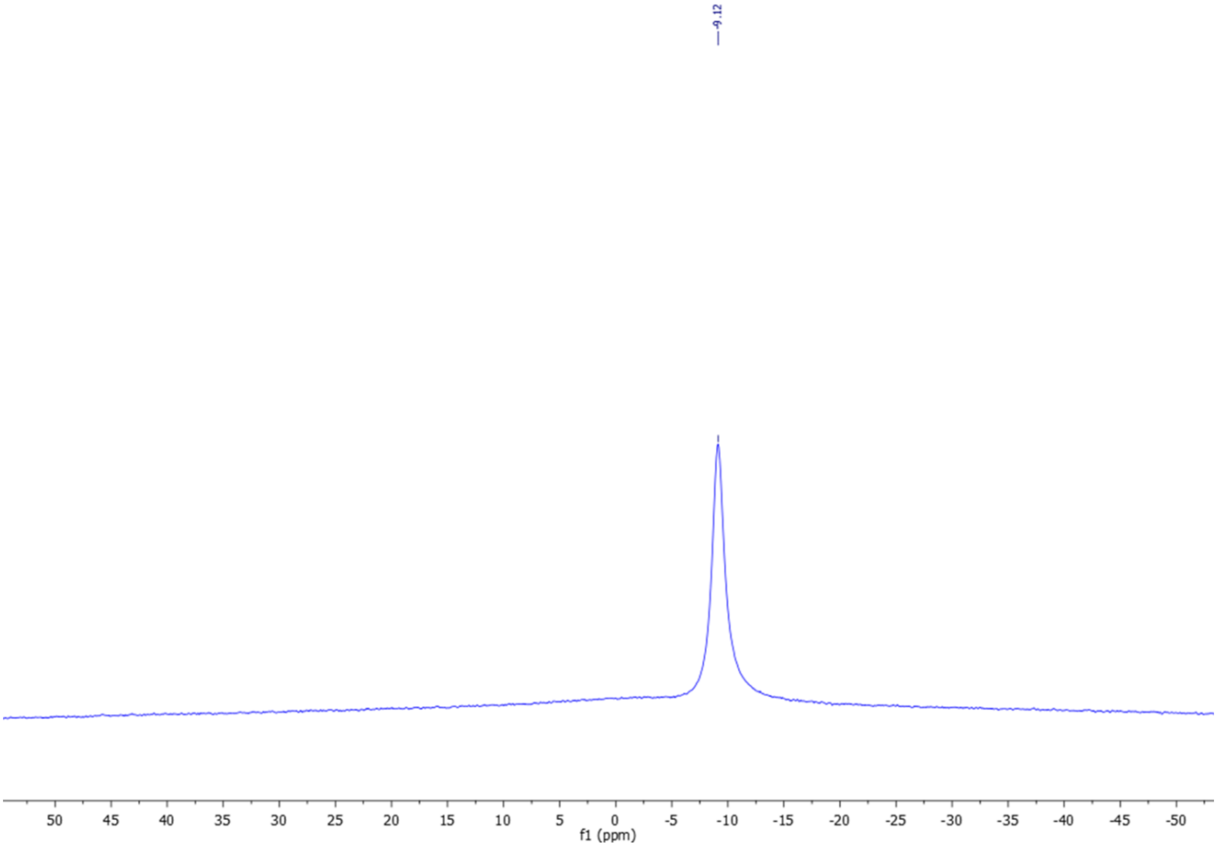

Figure SI 148: ^{11}B NMR spectrum of the analytical sample of the 3-(adamantan-1-yl)pent-4-en-2-imine tris(perfluorophenyl)borane complex **19** (128 MHz, Chloroform-*d*).

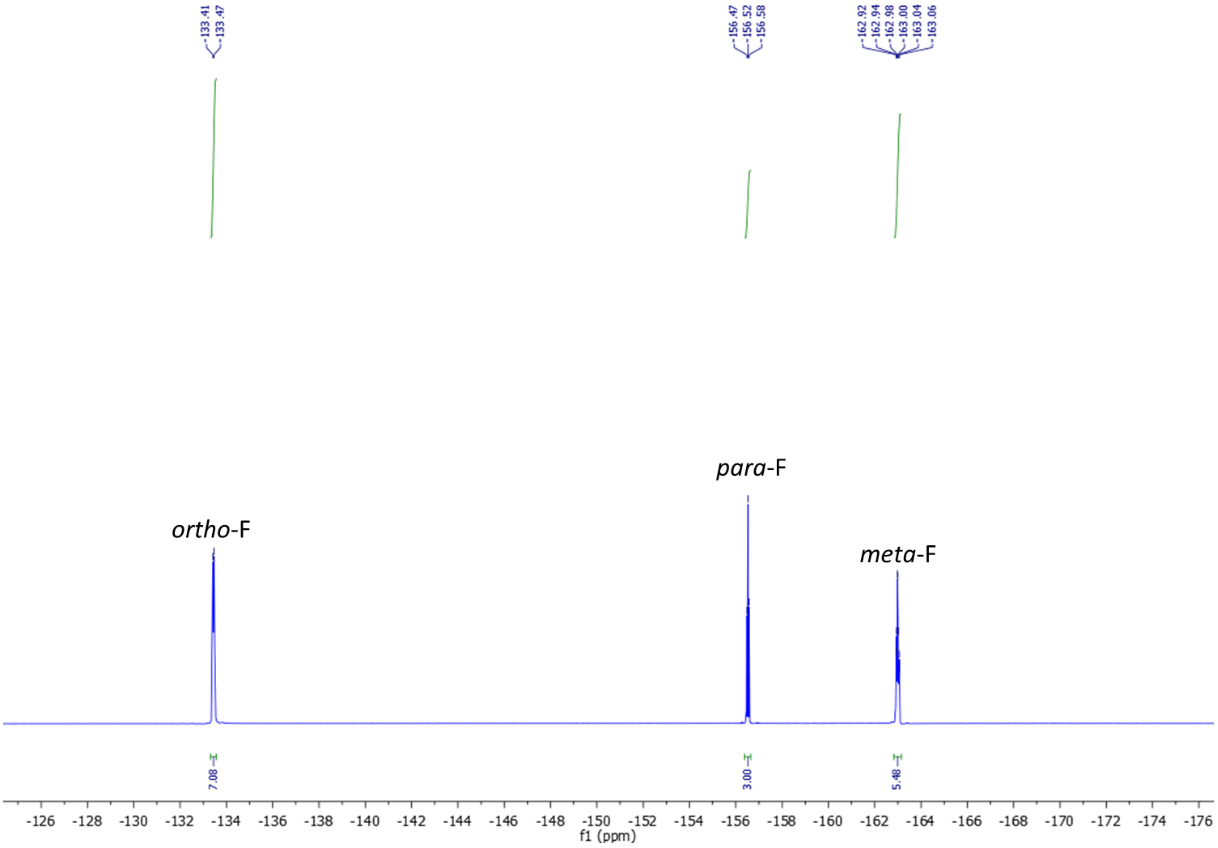

Figure SI 149: ^{19}F NMR spectrum of the analytical sample of the 3-(adamantan-1-yl)pent-4-en-2-imine tris(perfluorophenyl)borane complex **19** (377 MHz, Chloroform-*d*).

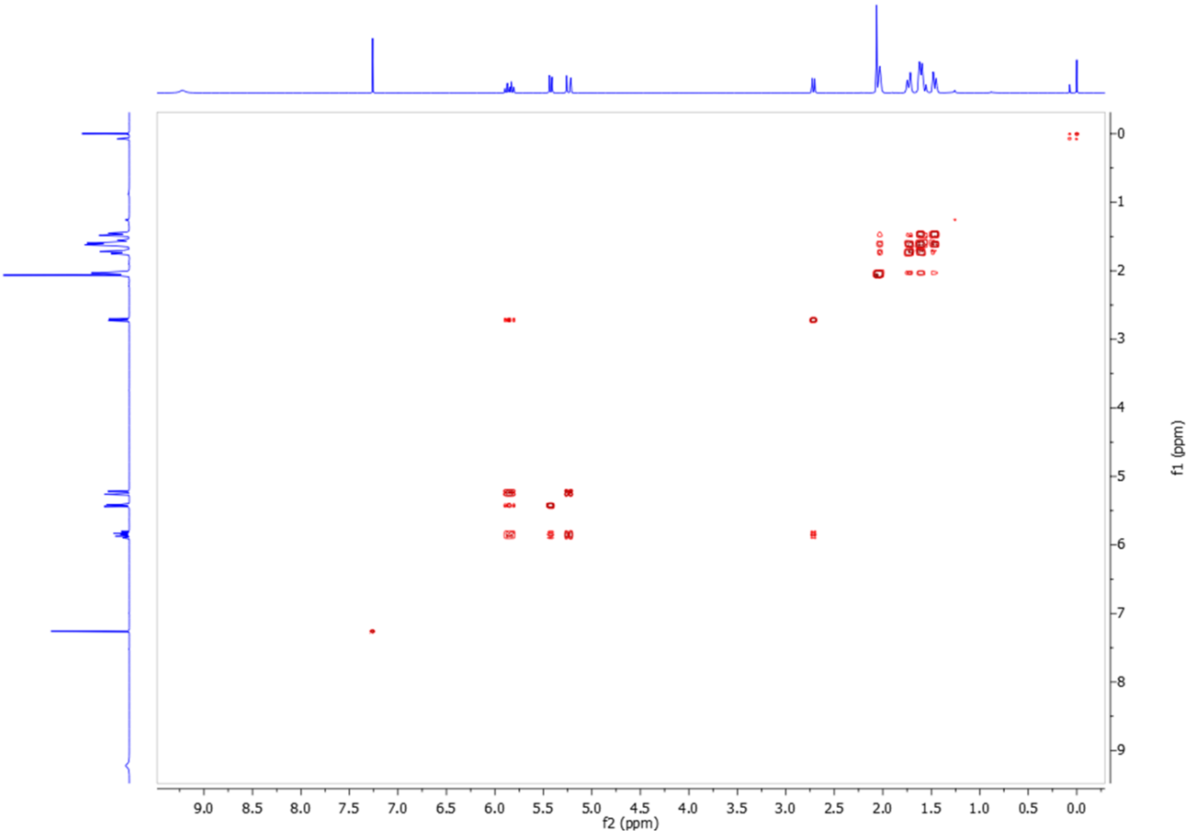

Figure SI 150: COSY NMR spectrum of the analytical sample of the 3-(adamantan-1-yl)pent-4-en-2-imine tris(perfluorophenyl)borane complex **19** (400 MHz, Chloroform-*d*).

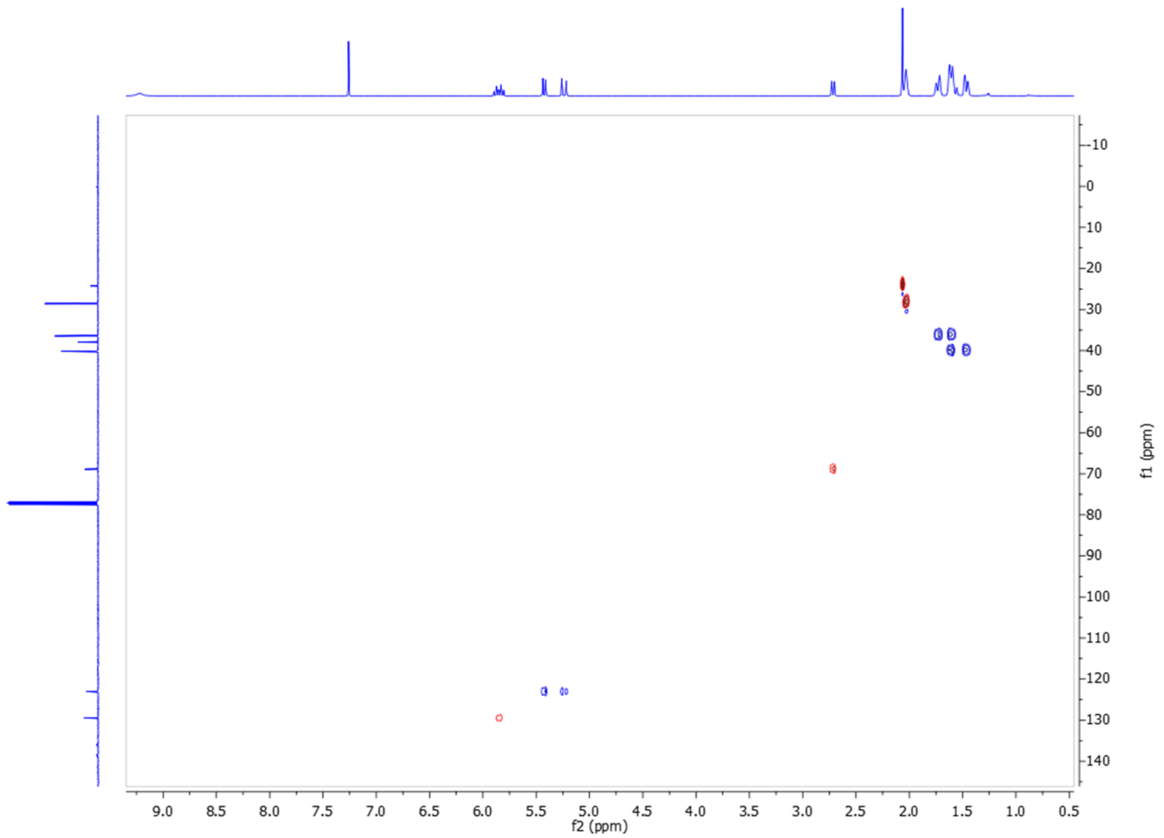

Figure SI 151: HSQC NMR spectrum of the analytical sample of the 3-(adamantan-1-yl)pent-4-en-2-imine tris(perfluorophenyl)borane complex **19** (101 MHz, Chloroform-*d*).

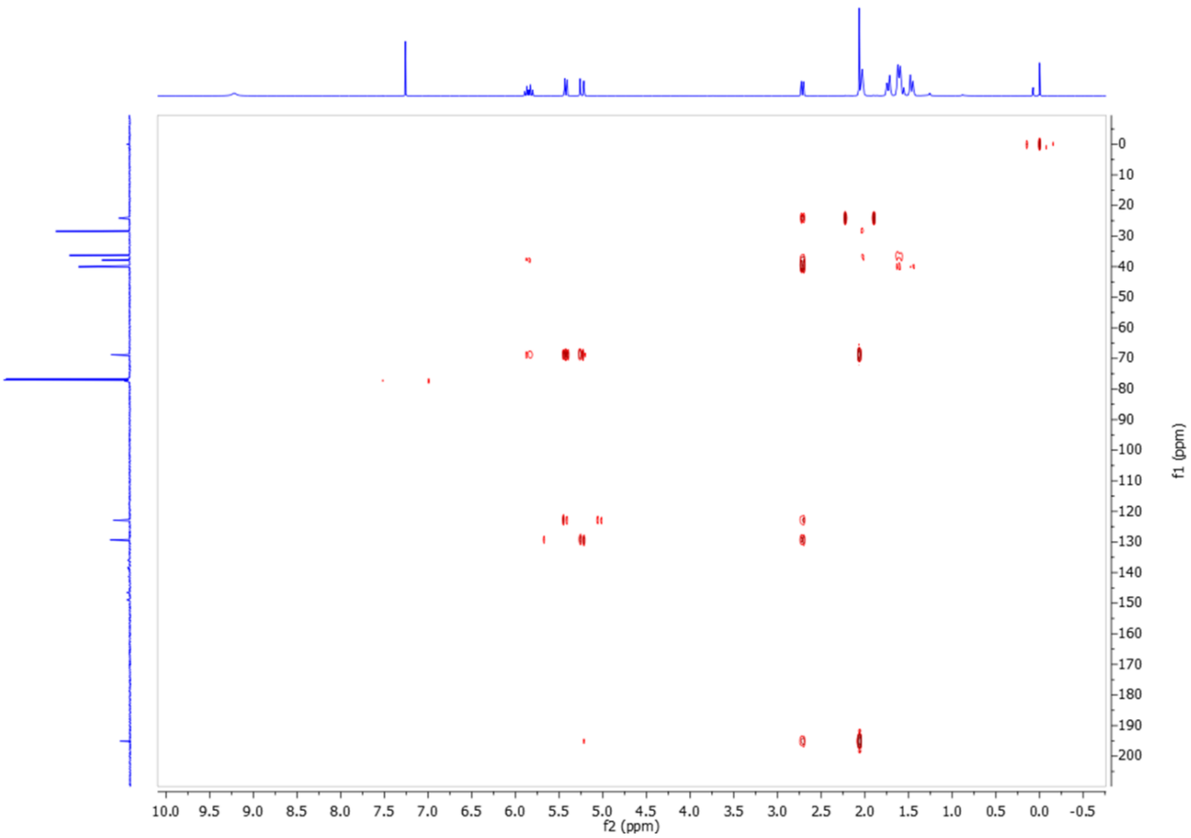

Figure SI 152: HMBC NMR spectrum of the analytical sample of the 3-(adamantan-1-yl)pent-4-en-2-imine tris(perfluorophenyl)borane complex **19** (101 MHz, Chloroform-*d*).

6 Mechanistic investigations

6.1 Consecutive H_2 activation and hydroboration of phenylallene starting from pyridonate borane 3

To show that after H_2 activation of pyridonate borane **3**, pyridone borane **4** exhibits borane reactivity, the H_2 activation of **3** in the presence of phenylallene was investigated. After dissociation from pyridone **5**, the free Piers borane **6** should react in a hydroboration reaction with phenylallene, yielding the allylborane pyridone complex **7** (Scheme SI 7).

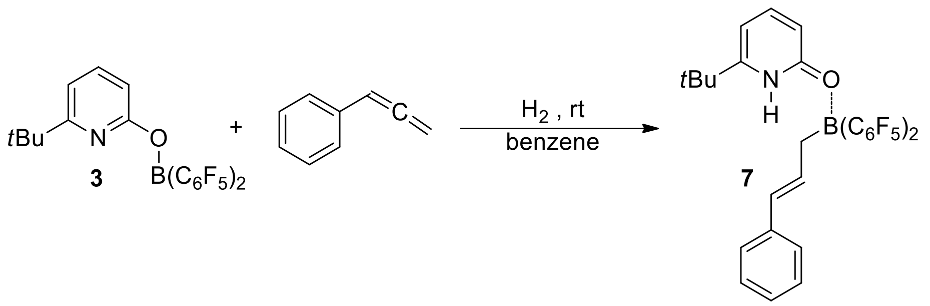

Scheme SI 7: Activation of H_2 and subsequent hydroboration of styrene starting from pyridonate borane **3**.

A stock solution of pyridone **5** and Piers borane **6** (300 µL) (0.1 M pyridone **5** and 0.11 M Piers borane **6** in benzene-d_6) was transferred to an NMR tube with J. Young valve, rinsed with benzene-d_6 (0.1 mL), subject to three freeze-pump-thawed cycles, and heated to 60 °C overnight under passive vacuum. The ^{1}H NMR spectrum shows the formation of pyridonate borane **3** (Figure SI 153).

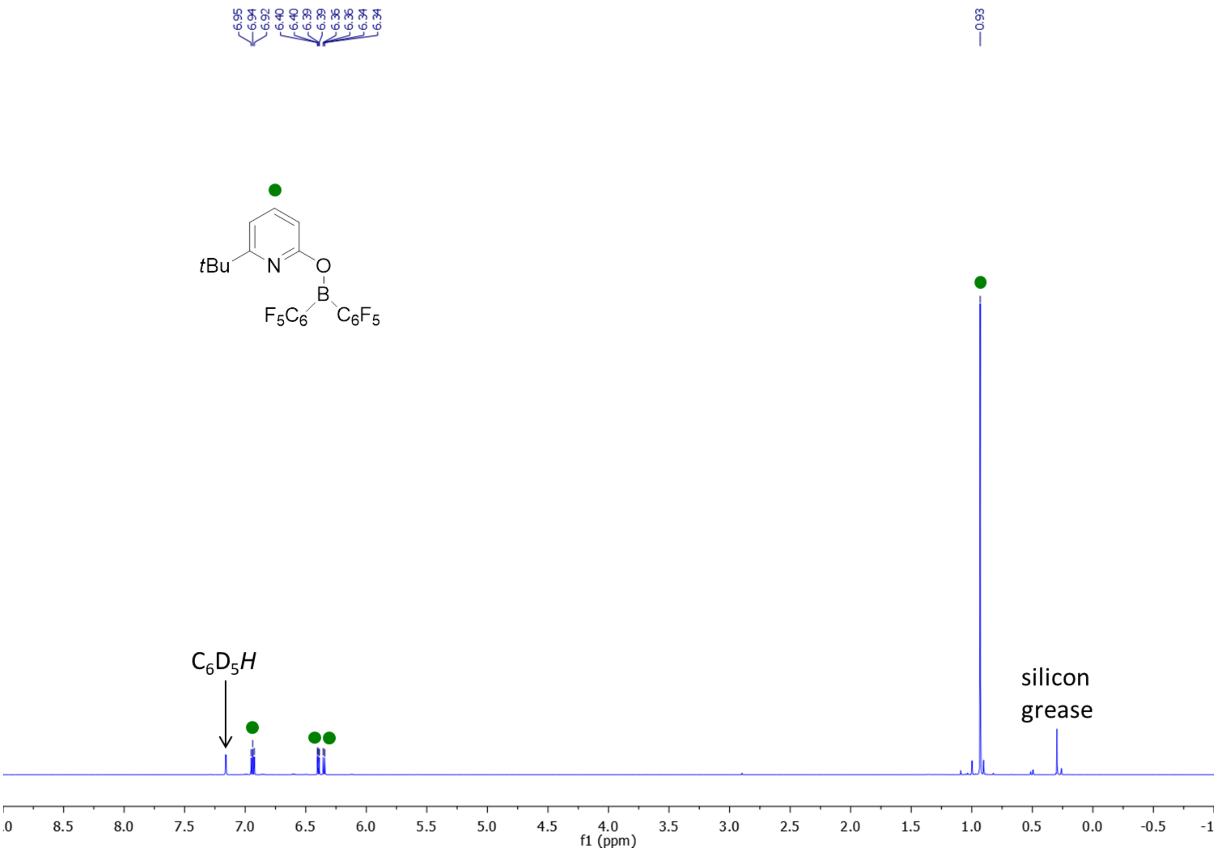

Figure SI 153: ^{1}H NMR spectrum of pyridonate borane **3** (600 MHz, benzene-d_6).

Then, phenylallene (3.8 µL, 0.03 mmol) was added and a ^{1}H and ^{11}B NMR spectra were measured showing unreacted phenylallene and pyridonate borane **3** (Figure SI 154, Figure SI 155).

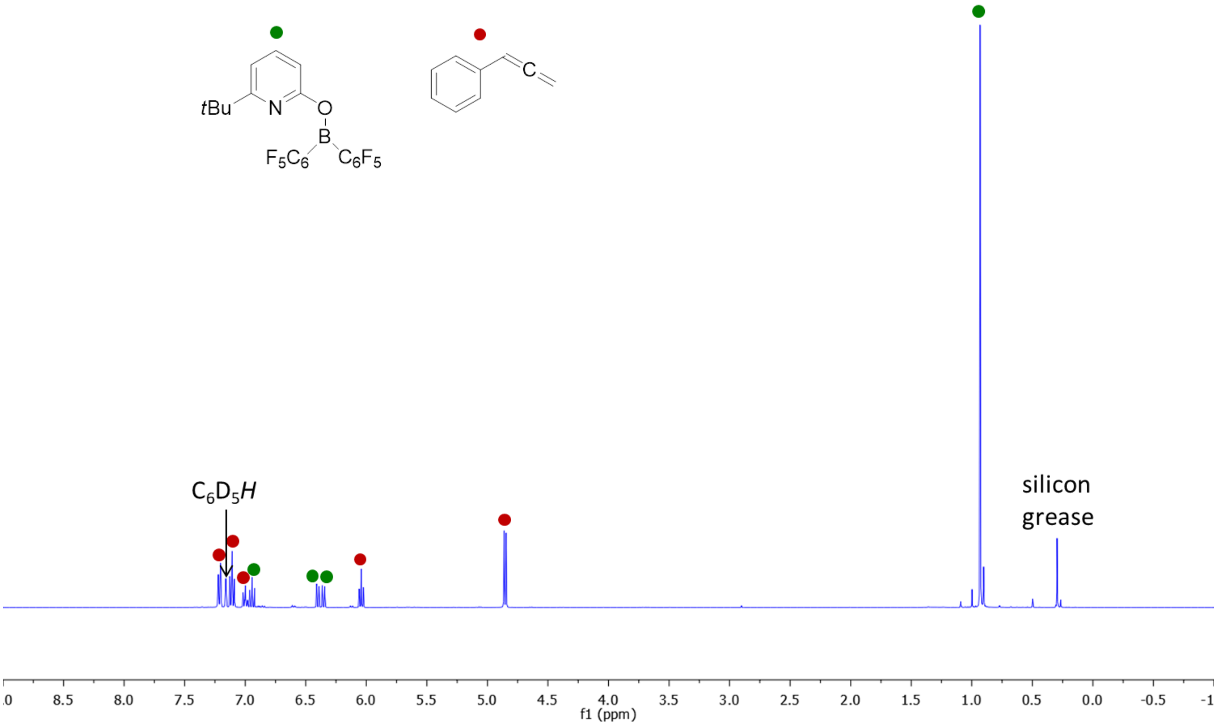

Figure SI 154: ^{1}H NMR spectrum of a reaction mixture of pyridonate borane **3** (green dot) and phenylallene (red dot) (400 MHz, benzene-d_6).

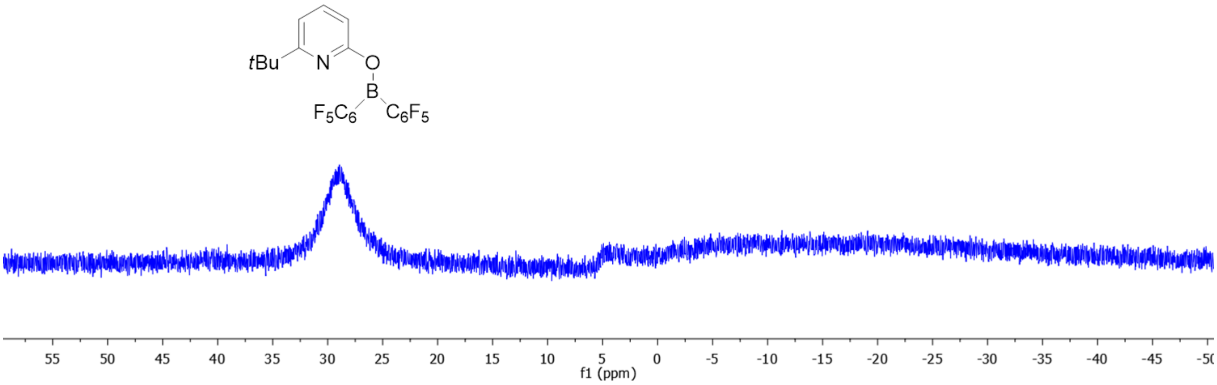

Figure SI 155: ^{11}B NMR spectrum of a reaction mixture of pyridonate borane **3** and phenylallene (128 MHz, benzene-d_6).

The reaction mixture was subject to three freeze-pump-thaw cycles and pressurized with H_2 (2.5 bar). The reaction mixture was kept at room temperature overnight. Afterwards it was again pressurized with H_2 (2.5 bar) and left overnight at room temperature. ^{1}H and ^{11}B NMR spectra were measured (Figure SI 156, Figure SI 157, Figure SI 158, Figure SI 159). The spectra show a complex reaction mixture. The three main components which could be identified are unreacted phenylallene, bispyridone complex **10** and allylborane pyridone complex **7**. The signals for **7** are identical to the allylborane pyridone complex **7** synthesized *via* the hydroboration of phenylallene with Piers borane **6** and addition of one equivalent pyridone **5** (see chapter 3.3).

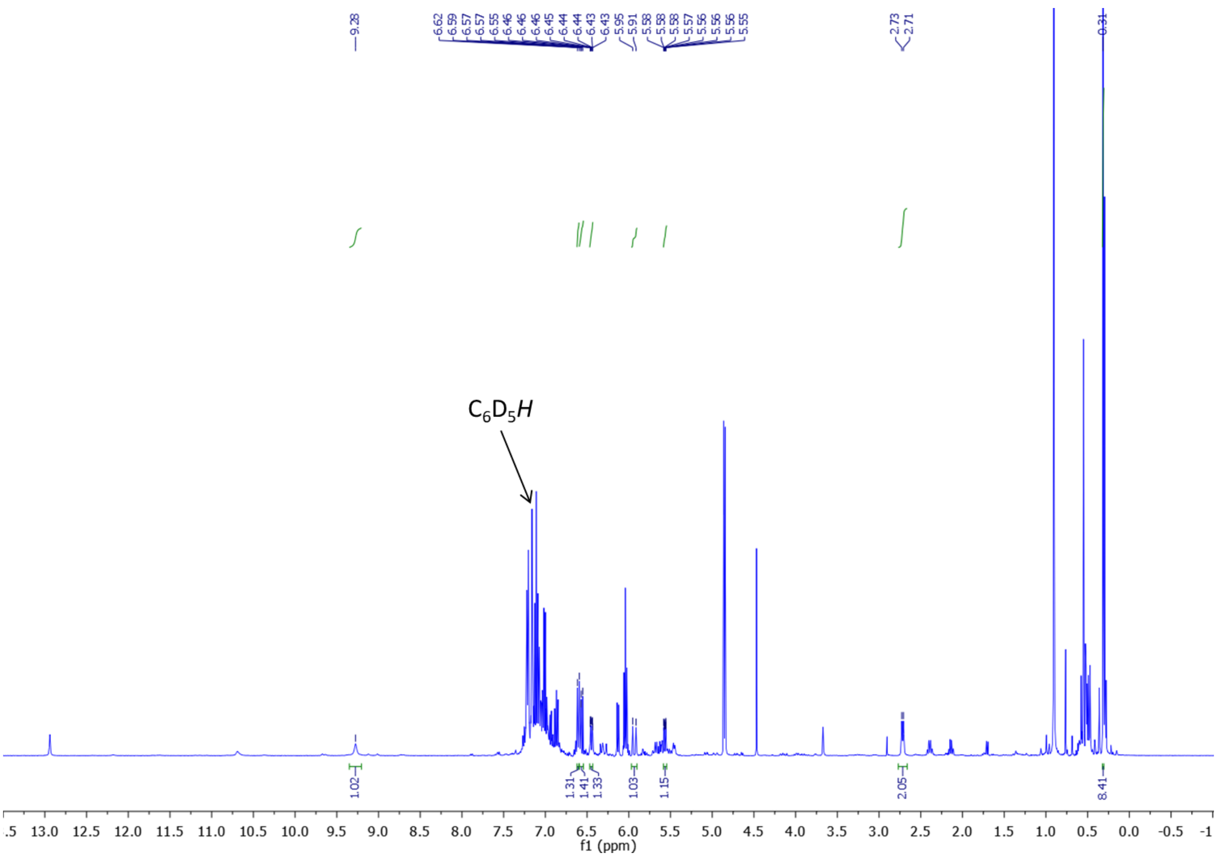

Figure SI 156: ^{1}H NMR spectrum of a reaction mixture of pyridonate borane **3** and phenylallene after 3 d at room temperature under H_2 pressure (2.5 bar) (400 MHz, benzene-d_6).

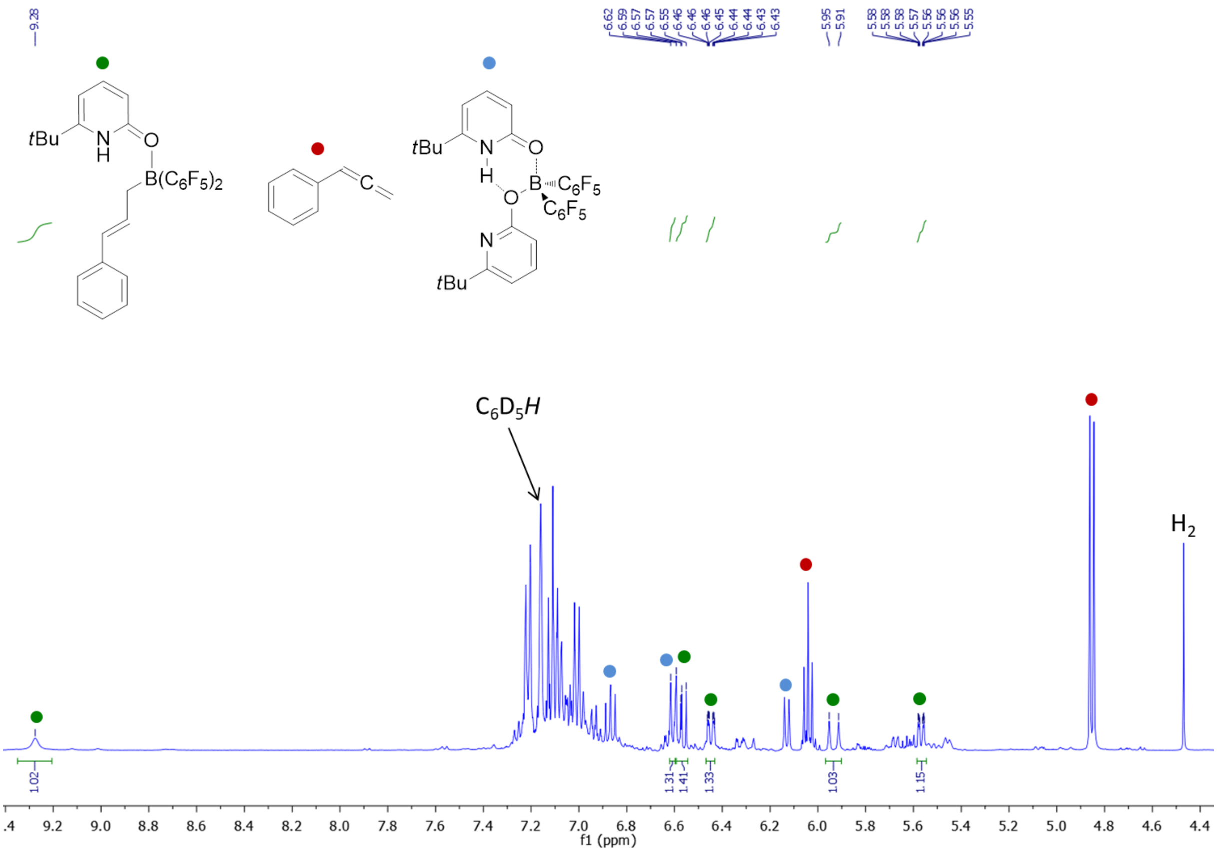

Figure SI 157: Low field excerpt of the ^{1}H NMR spectrum of a reaction mixture of pyridonate borane **3** and phenylallene (red dot) after 3 d at room temperature under H_2 pressure (2.5 bar) (400 MHz, benzene-d_6).

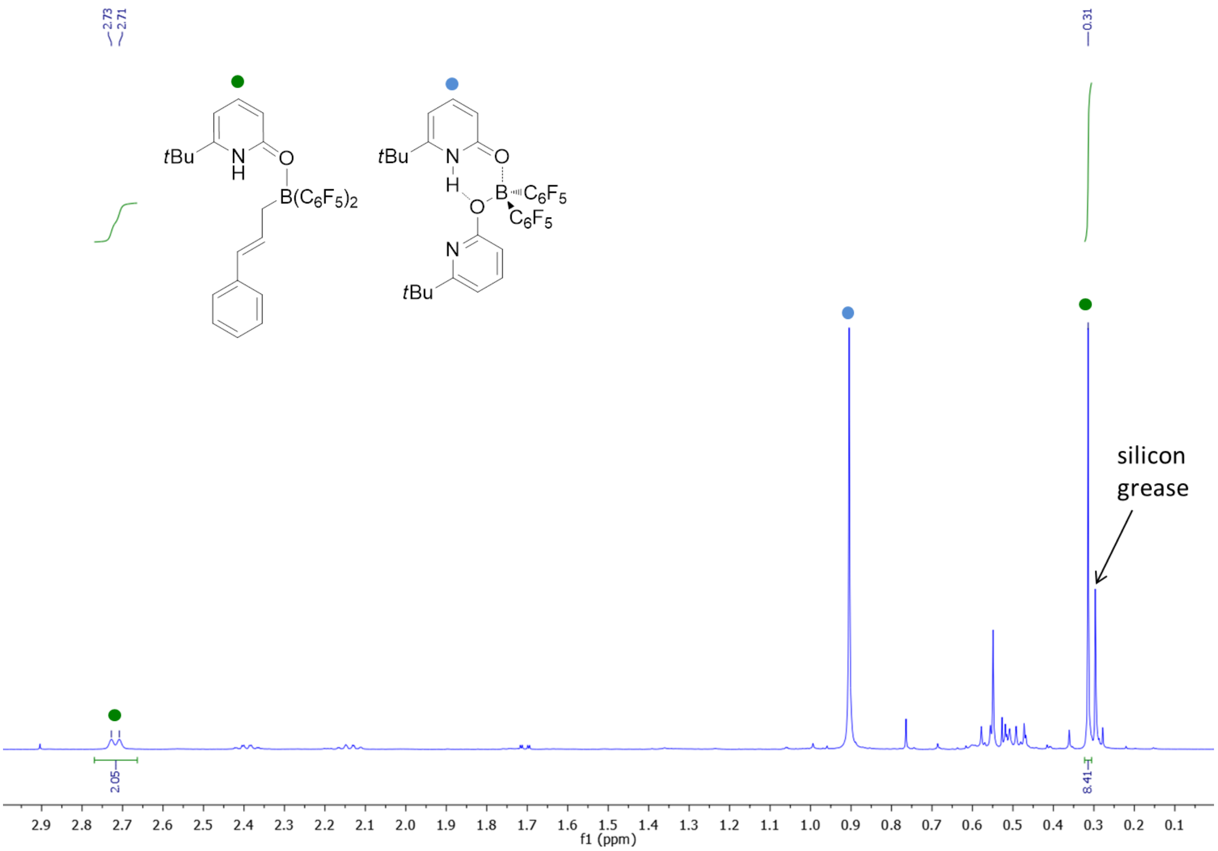

Figure SI 158: High field excerpt of the ^{1}H NMR spectrum of a reaction mixture of pyridonate borane **3** and phenylallene after 3 d at room temperature under H_2 pressure (2.5 bar) (400 MHz, benzene-d_6).

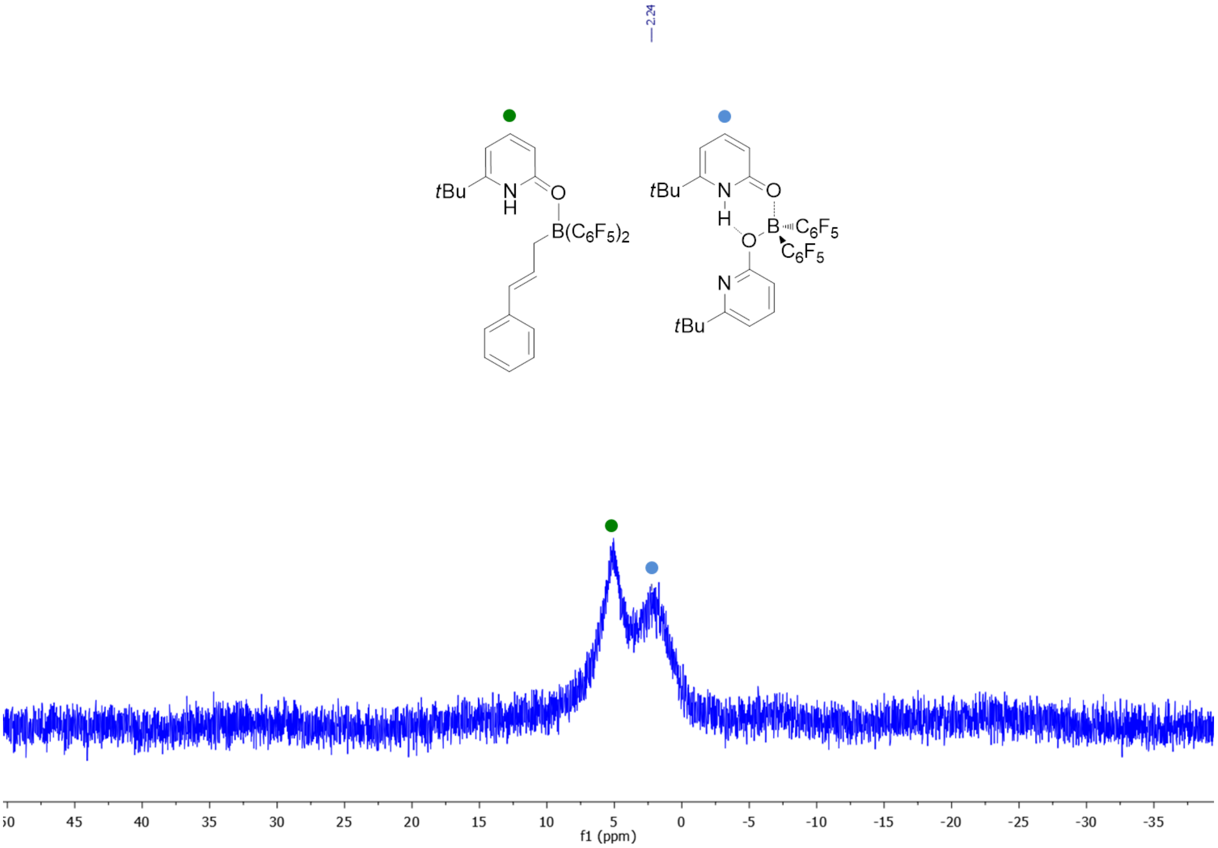

Figure SI 159: ^{11}B NMR spectrum of a reaction mixture of pyridonate borane **3** and phenylallene after 3 d at room temperature under H_2 pressure (2.5 bar) (128 MHz, benzene-d_6).

6.2 Stochiometric synthesis of β-diketiminate borane complex 9 from pyridonate borane 3, phenylallene, and acetonitrile

A stock solution of pyridone **5** and Piers borane **6** (300 µL) (0.1 M pyridone **5** and 0.11 M Piers borane **6** in benzene-d_6) was transferred to an NMR tube with J. Young valve. Benzene-d_6 was added (0.1 mL), the tube was freeze-pump-thawed three times and heated to 60 °C overnight under passive vacuum. Phenylallene (3.8 µL, 0.03 mmol) and acetonitrile (1.6 µL, 0.03 mmol) were added. The tube was again subject to three freeze-pump-thaw cycles, pressurized with H_2 (2.5 bar) and kept at room temperature overnight. The next day a ^{1}H and ^{11}B NMR spectrum were measured. The ^{1}H NMR spectrum shows phenylallene, β-diketiminate borane complex **9**, and bispyridone complex **10** as main components in a 2.5 : 1 :2.5 ratio (Figure SI 160, Figure SI 161, Figure SI 162).

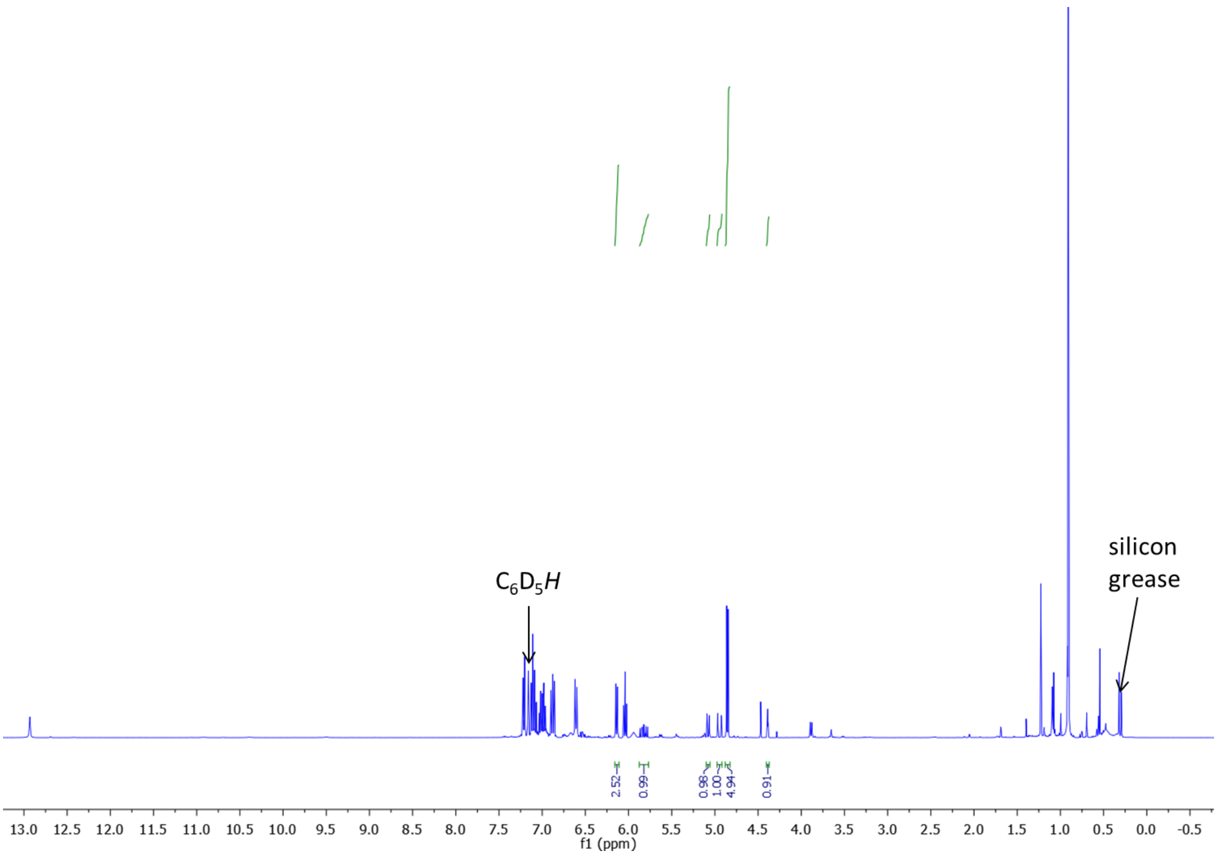

Figure SI 160: ^{1}H NMR spectrum after the reaction of pyridonate borane **3** with phenylallene and acetonitrile under H_2 pressure (400 MHz, benzene-d_6).

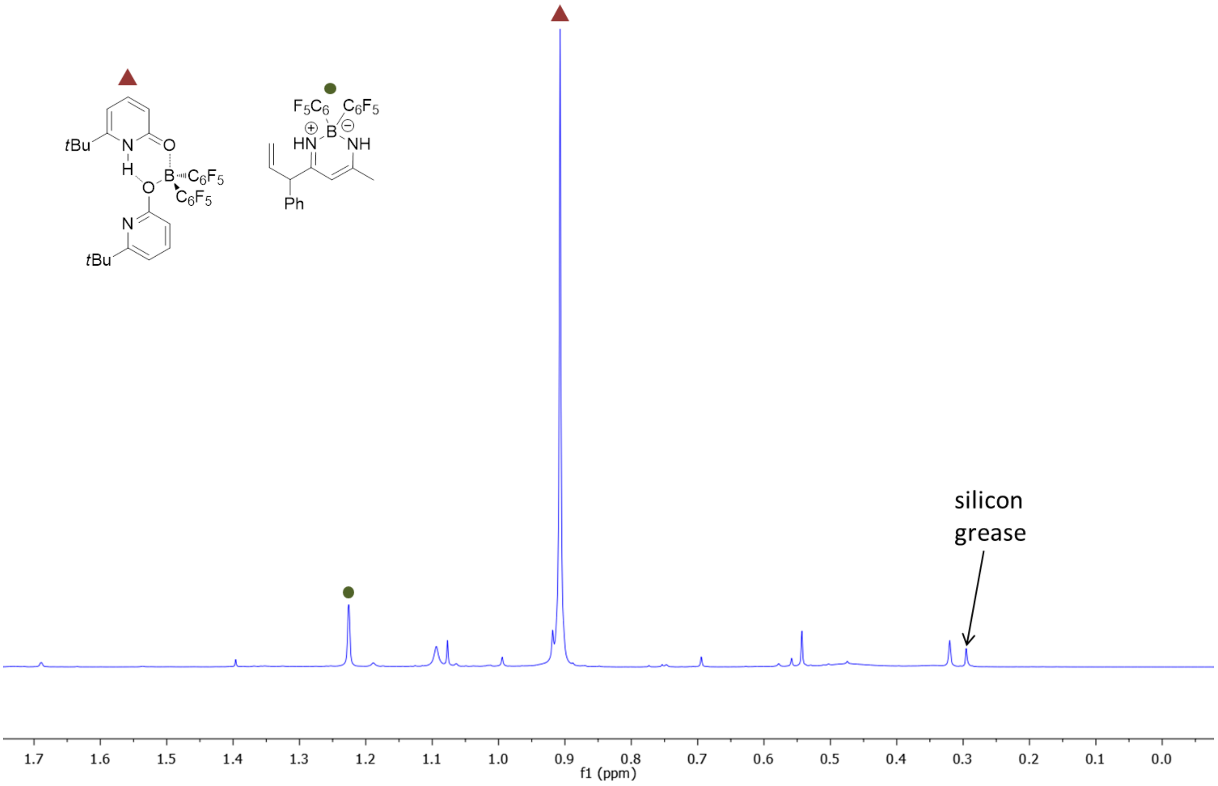

Figure SI 161: High field excerpt of the ^{1}H NMR spectrum after the reaction of pyridonate borane **3** with phenylallene and acetonitrile under H_2 pressure (400 MHz, benzene-d_6).

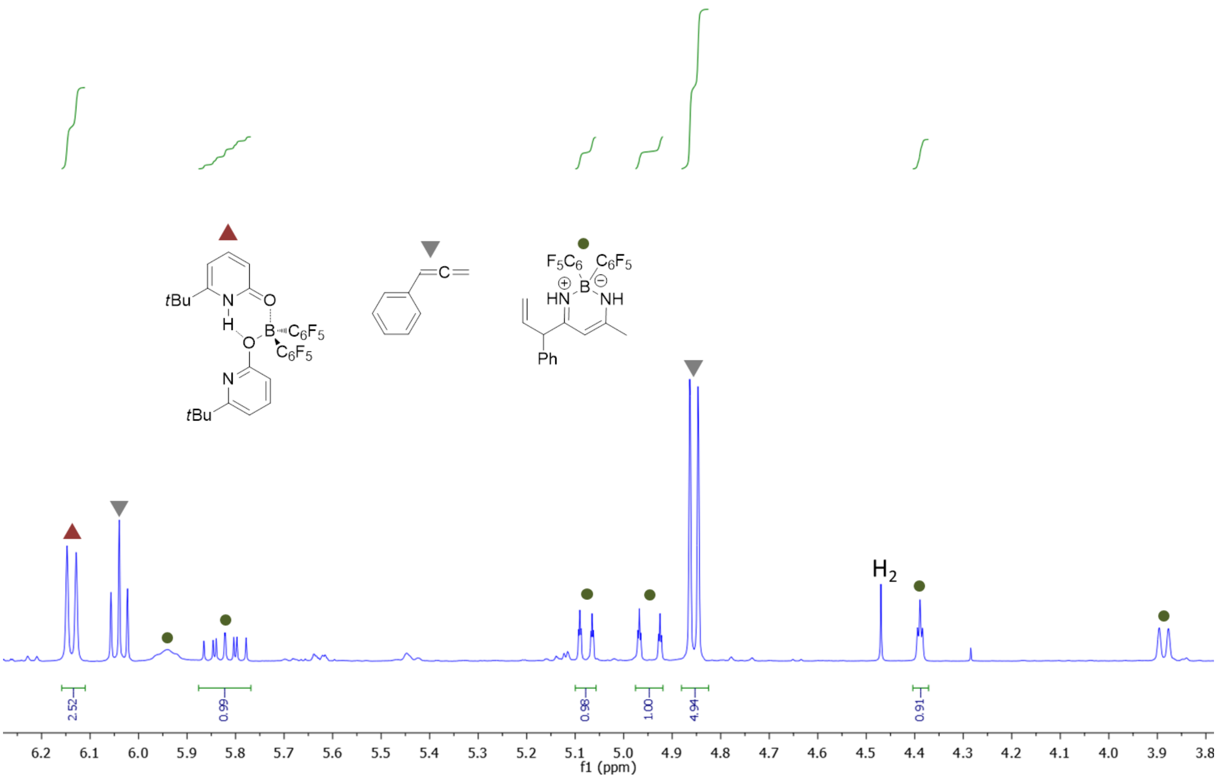

Figure SI 162: Low field excerpt of the ^{1}H NMR spectrum after the reaction of pyridonate borane **3** with phenylallene and acetonitrile under H_2 pressure (400 MHz, benzene-d_6).

The corresponding ^{11}B NMR spectrum shows the two main components β-diketiminate borane complex **9**, and bispyridone complex **10** (Figure SI 163).

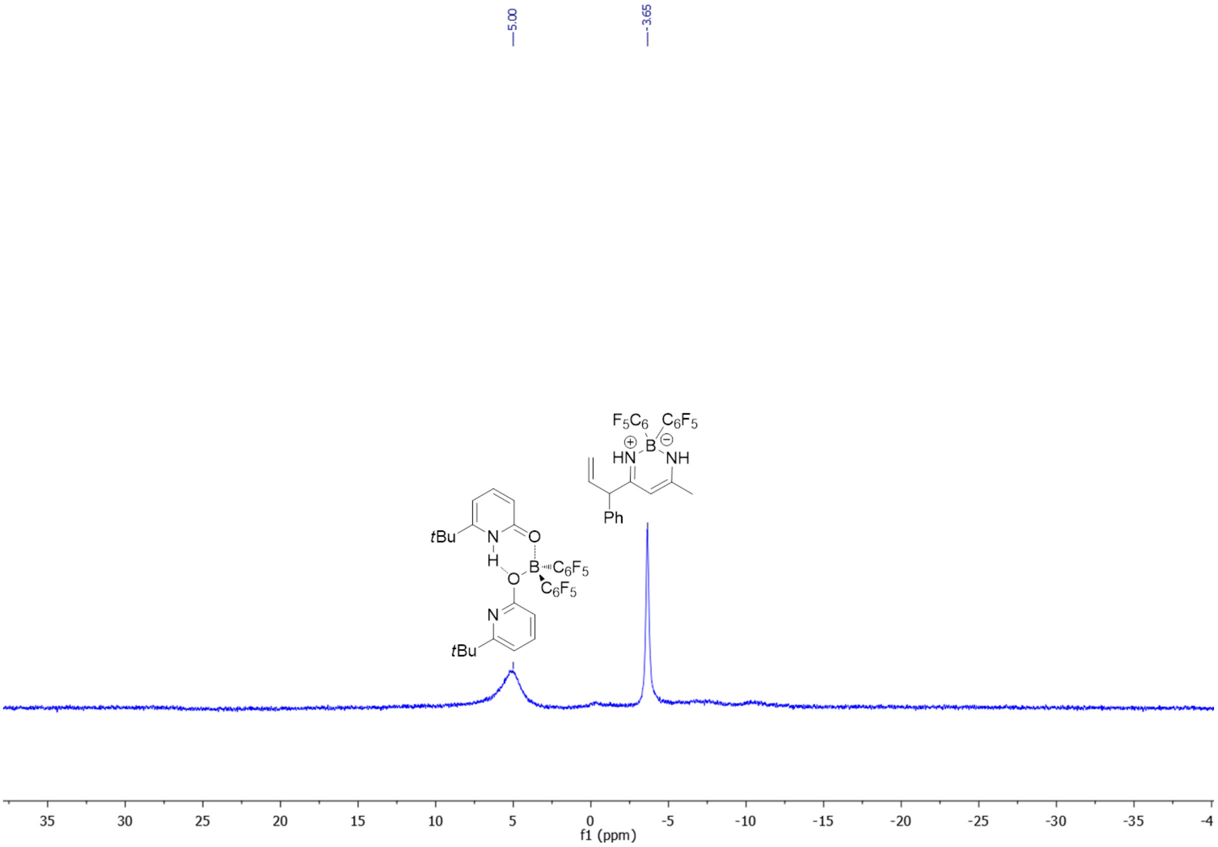

Figure SI 163: ^{11}B NMR spectrum after the reaction of pyridonate borane **3** with phenylallene and acetonitrile under H_2 pressure (128 MHz, benzene-d_6).

6.3 Stochiometric synthesis of β-diketiminate borane complex 9 from ketiminoborane pyridone complex 12, pyridonate borane 3 and acetonitrile

We envisioned that the formation of ß-diketiminate borane complex **9** is the result of the tautomerization ketiminoborane pyridone complex **12** and subsequent nucleophilic attack of the resulting enamine at acetonitrile.

Addition of one equivalent of pyridonate borane **3** should catch the free 6-*tert*-butyl-2-pyridone **5** in the bispyridone complex **10** providing a thermodynamic sink after dissociation (Scheme SI 8).

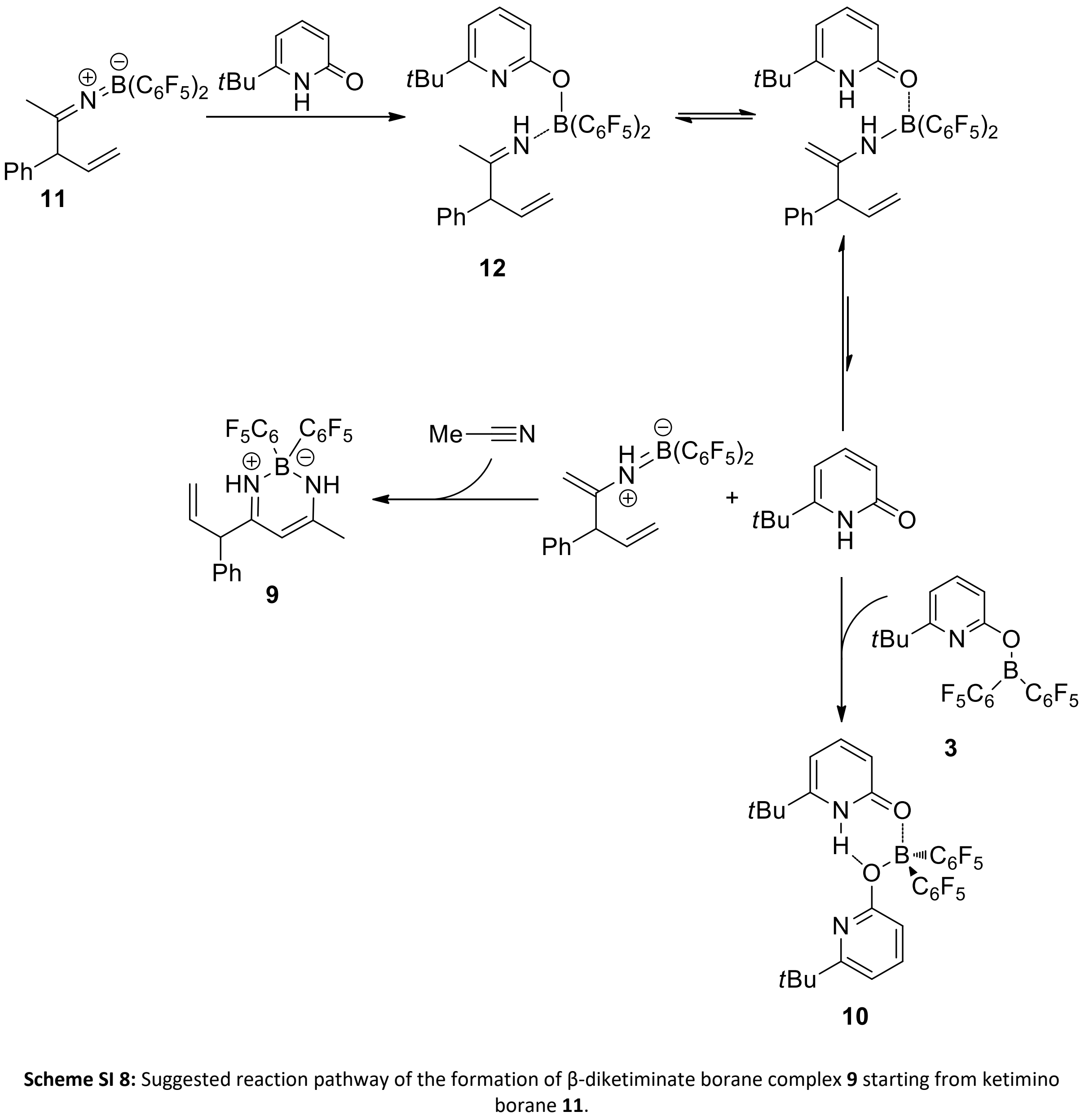

Scheme SI 8: Suggested reaction pathway of the formation of β-diketiminate borane complex **9** starting from ketimino borane **11**.

The ketiminoborane pyridone complex **12** was synthesized in two steps. First ketiminoborane **11** was synthesized as described in chapter 3.5. Afterwards pyridone **5** was added (4.5 mg, 0.03 mmol). The corresponding ^{1}H spectra show three main components: Bispyridone complex **10**, a slight excess of phenylallene, and ketimine borane pyridone complex **12** (Figure SI 164, Figure SI 165, Figure SI 166).

The respective ^{11}B NMR spectrum shows signals for bispyridone complex **10**, ketimine borane pyridone complex **12** and trace amounts of β-diketiminate borane complex **9** (Figure SI 167).

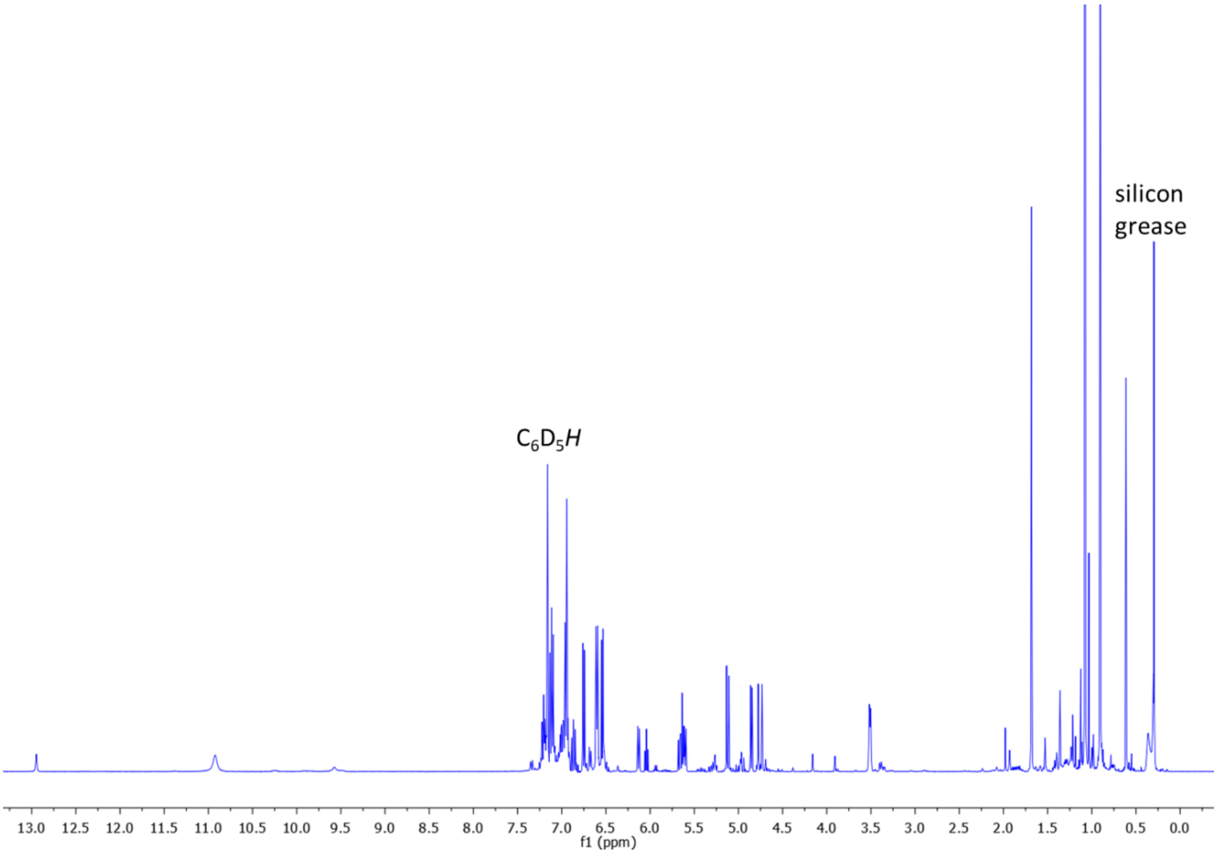

Figure SI 164: ^{1}H NMR spectrum of the synthesis of ketimine borane pyridone complex **12** (400 MHz, benzene-d_6).

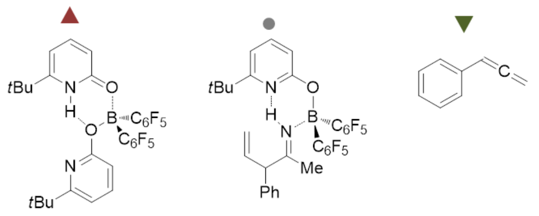

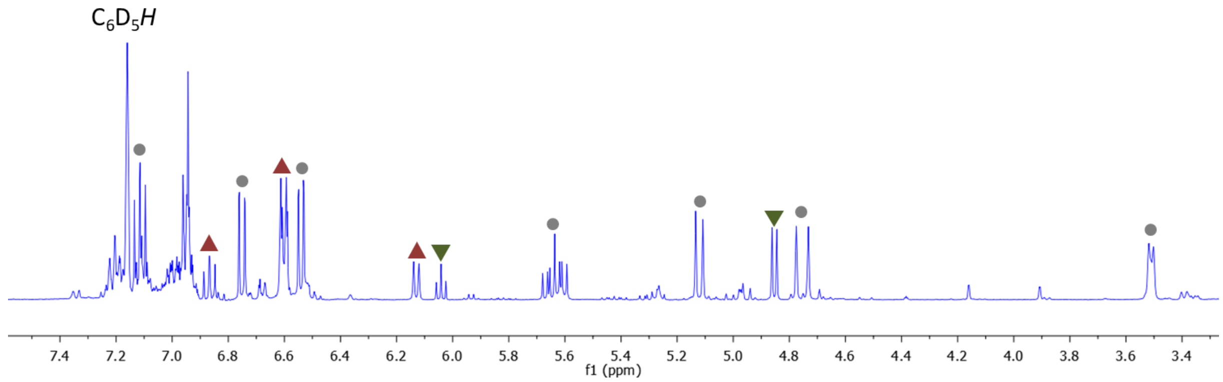

Figure SI 165: Low field excerpt of the ^{1}H NMR spectrum of the synthesis of ketimine borane pyridone complex **12** (400 MHz, benzene-d_6).

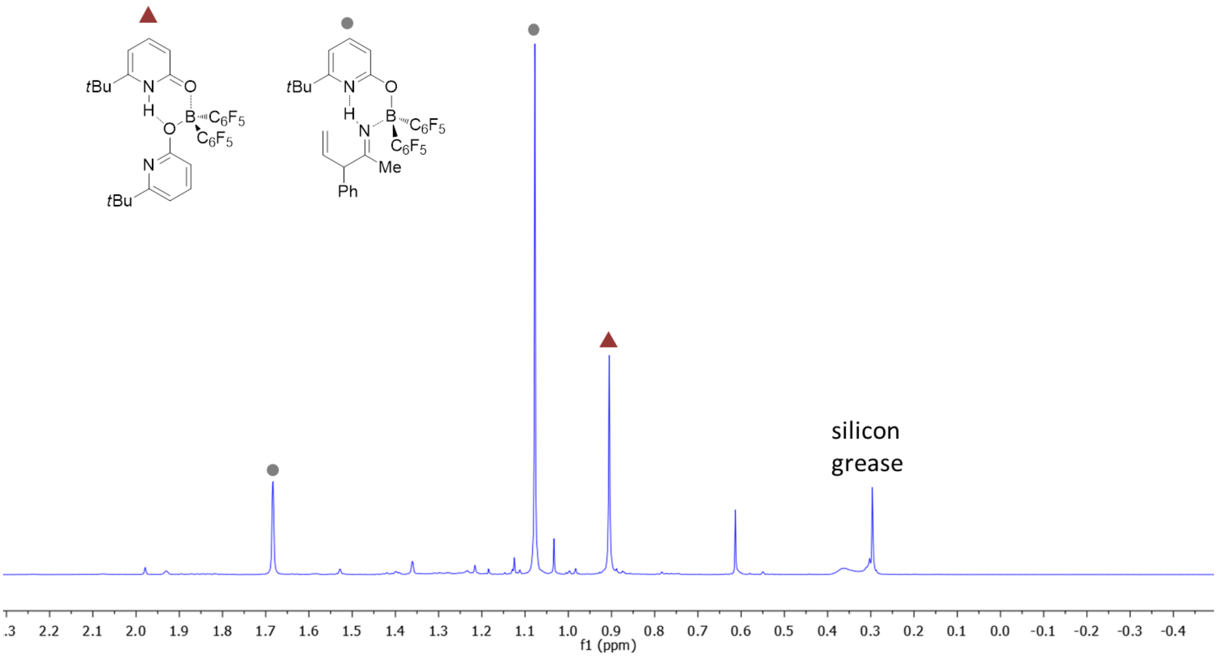

Figure SI 166: High field excerpt of the ^{1}H NMR spectrum of the synthesis of ketimine borane pyridone complex **12** (400 MHz, benzene-d_6).

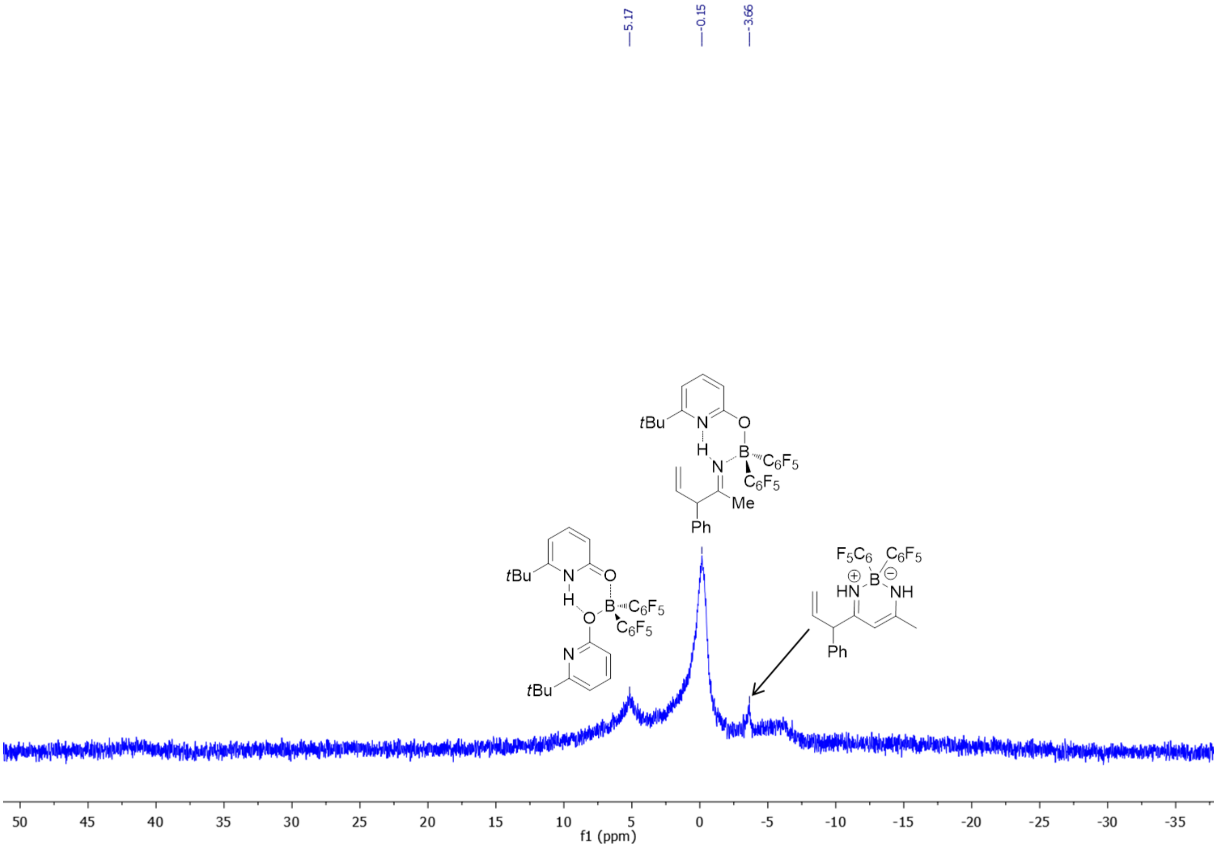

Figure SI 167: ^{11}B NMR spectrum of the synthesis of ketimine borane pyridone complex **12** (128 MHz, benzene-d_6).

Pyridonate borane **3** (0.03 mmol) was synthesized in an NMR tube with J Young valve in benzene–d_6 as described above. Pyridonate borane **3** and the reaction mixture containing **12** were transferred to a glass vial. Acetonitrile (1.6 µL, 0.03 mmol) was added and an aliquot was transferred to an NMR tube with J Young valve and kept at room temperature overnight.

The next day ^{1}H and ^{11}B NMR spectra were measured showing bispyridone complex **10** and β-diketiminate borane complex **11** were the main components (Figure SI 168, Figure SI 169, Figure SI 170).

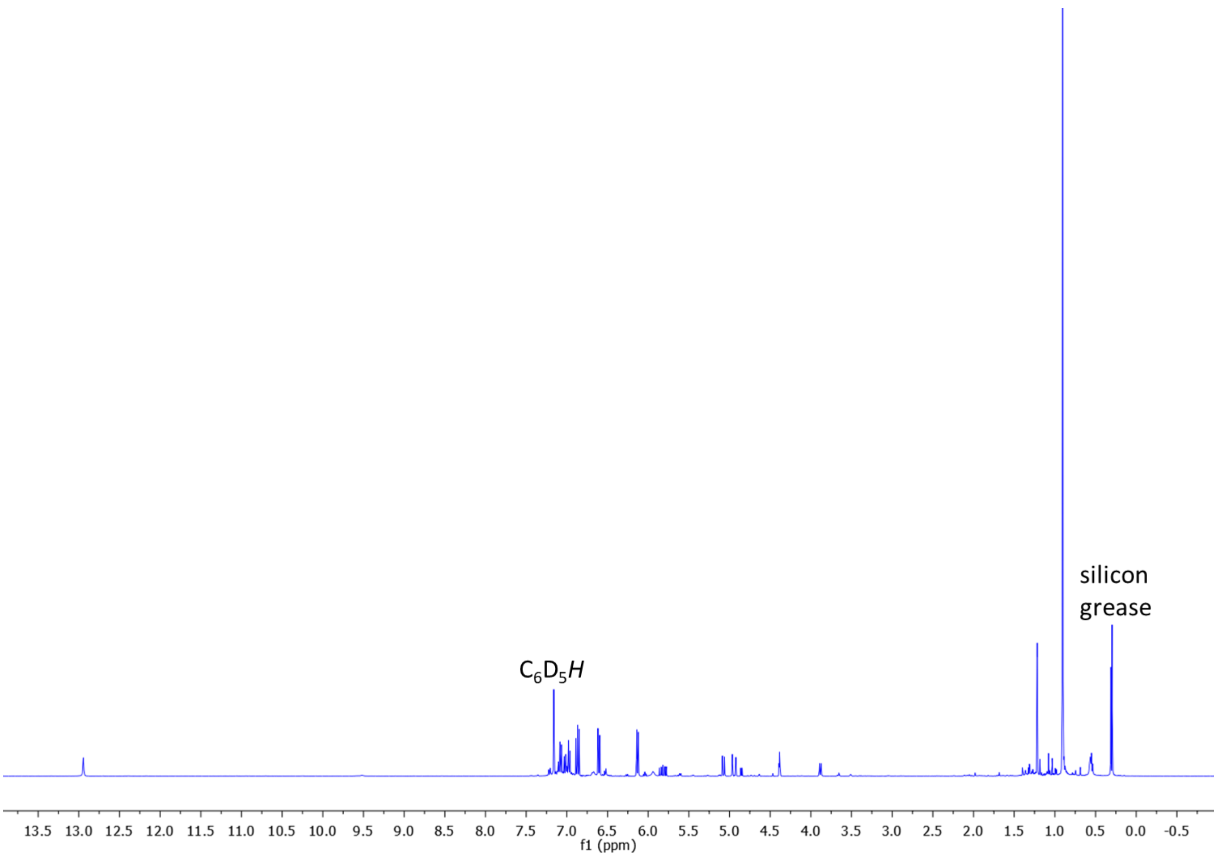

Figure SI 168: ^{1}H NMR spectrum of the reaction of ketimine borane pyridone complex **12** with acetonitrile and pyridonate borane **3** after overnight at room temperature (400 MHz, benzene-d_6).

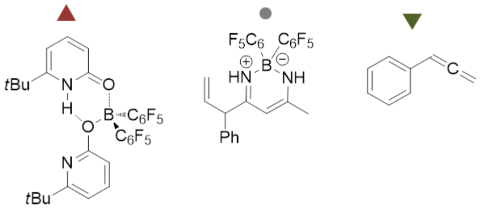

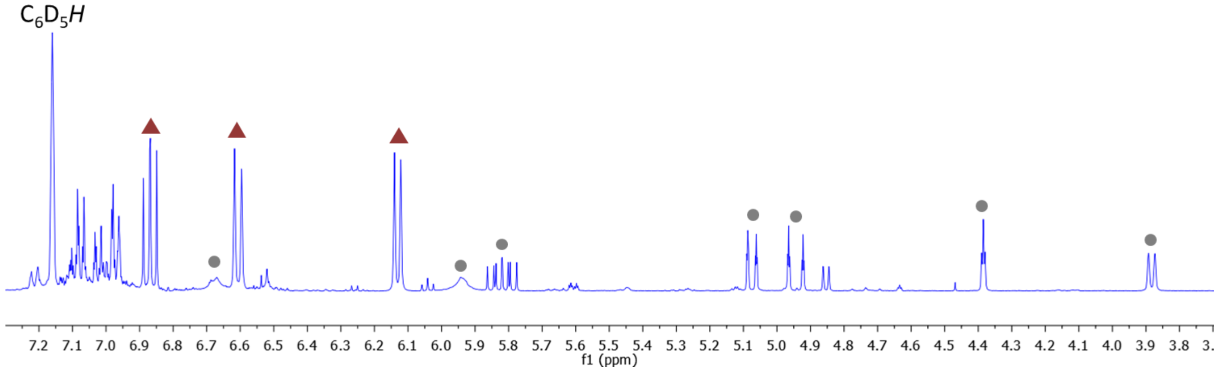

Figure SI 169: Low field excerpt of the ^1H NMR spectrum of the reaction of ketimine borane pyridone complex **12** with acetonitrile and pyridonate borane **3** after overnight at room temperature (400 MHz, benzene-d_6).

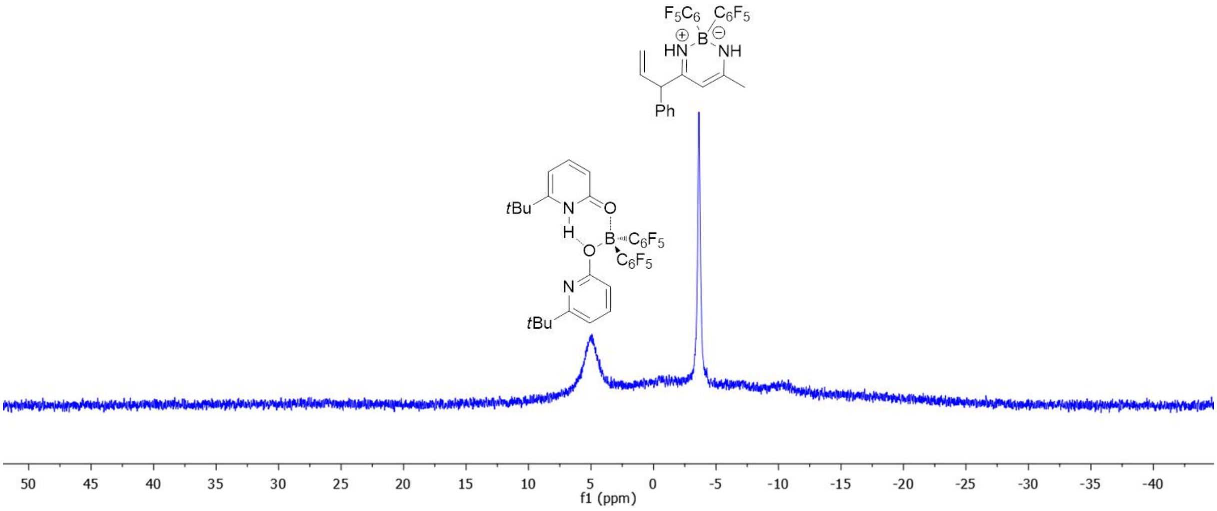

Figure SI 170: ^{11}B NMR spectrum of the reaction of ketimine borane pyridone complex **12** with acetonitrile and pyridonate borane **3** after overnight at room temperature (128 MHz, benzene-d_6).

The yield of β-diketiminate borane complex **9** was quantified in an identical experiment on a 0.05 mmol scale by adding 1,3,5-trimethoxybenzenze (50 µL of a 0.5 M stock solution in benzene-d_6) as an internal standard (see Figure SI 171). The yield of **9** was 71 % starting from phenylallene, Piers borane **6** and acetonitrile over four steps or 86 % starting from ketiminoborane **11** over two steps.

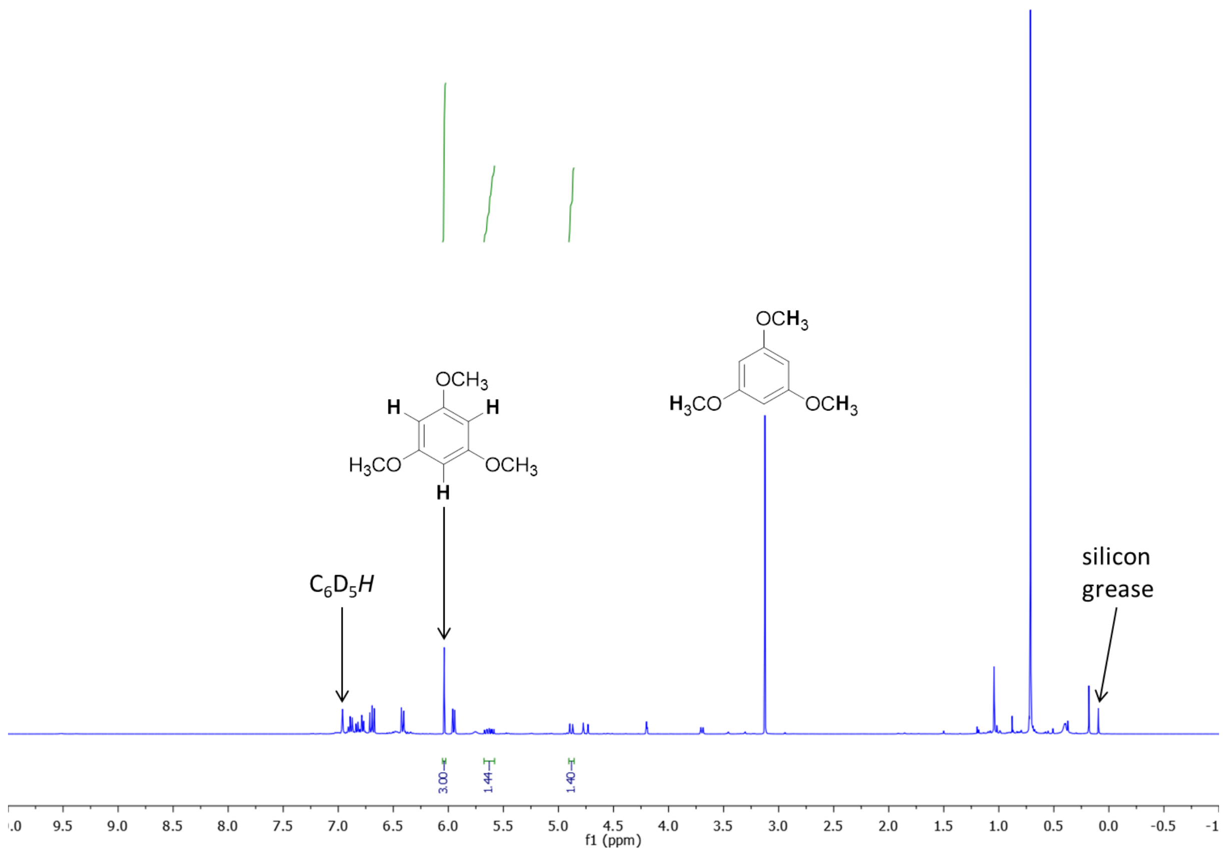

Figure SI 171: ^{1}H NMR spectrum of the reaction of ketiminoborane pyridone complex **12** with acetonitrile and pyridonate borane **3** after overnight at room temperature with 1,3,5-trimethoxybenzene (50 µL of a 0.5 M stock solution in benzene-d_6) (400 MHz, benzene-d_6).

6.4 Transformation of the kinetic to the thermodynamic product

According to the computations the allylimine complex **13** and an equivalent of acetonitrile is thermodynamically less stable then the corresponding β-diketiminate borane complex **9**. With one equivalent pyridonate borane **3** this transformation has a computed activation energy of 25.5 kcal/mol which should be thermally accessible (Scheme SI 9)

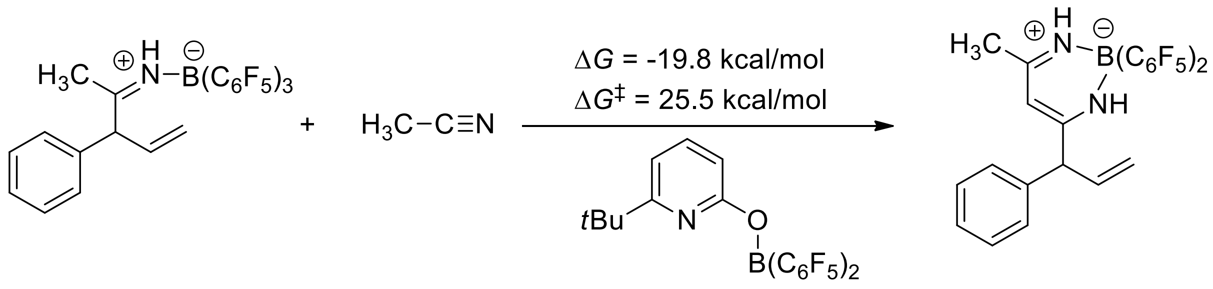

Scheme SI 9: Transformation of the allylimine borane complex **13** (kinetic product) to the thermodynamically more stable β-diketiminate borane complex **9** using pyridonate borane **3**. Energies were computed at revDSD-PBEP86-D4/def2-QZVPP//PBEh-3c. The SMD model for benzene was used to implicitly account for solvent effects.

A stock solution of pyridone **5** and Piers borane **6** (300 µL) (0.1 M pyridone **5** and 0.11 M Piers borane **6** in benzene-d_6) was transferred to an NMR tube with J. Young valve. Benzene-d_6 was added (0.2 mL) and the reaction mixture was subject to three freeze-pump-thaw cycles and heated overnight to 60 °C under passive vacuum. Allylimine borane complex **13** (20.1 mg, 0.03 mmol) and acetonitrile (1.58 µL, 0.03 mmol) were added. The reaction mixture was heated for 22 h to 80 °C and a ^{1}H and ^{11}B NMR spectrum were measured (Figure SI 172, Figure SI 173, Figure SI 174). Prolonged heating did not result in further change of the reaction mixture. 1,3,5-trimethoxybenzene (50 µL of a 0.3 M stock solution in benzene-d_6, 0.015 mmol) was added as an internal standard. The ^{1}H NMR spectrum shows an *in situ* yield of β-diketiminate borane complex **9** of 81% while 16% of allylimine borane complex **13** remained unchanged.

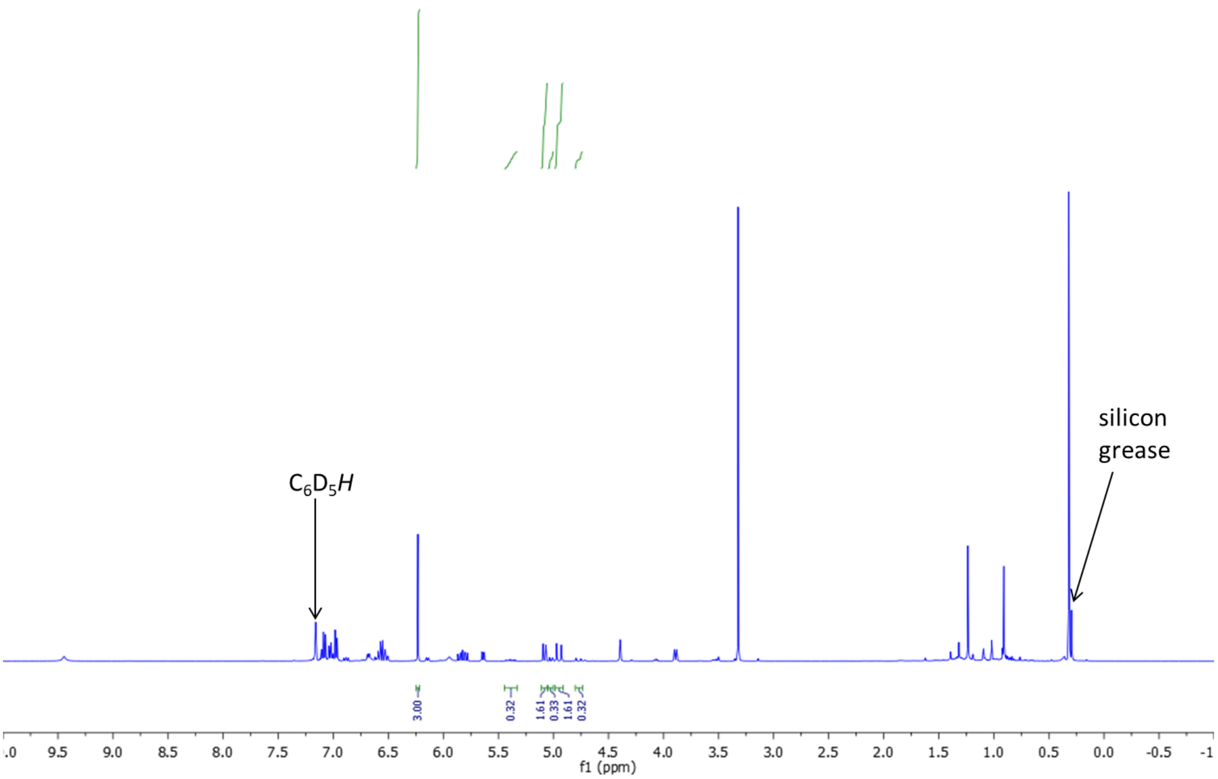

Figure SI 172: ^{1}H NMR spectrum of the reaction mixture after heating allylimine borane complex **13** with one equivalent of acetonitrile and pyridonate borane **3** after heating to 80 °C for 22 h (400 MHz, benzene-d_6).

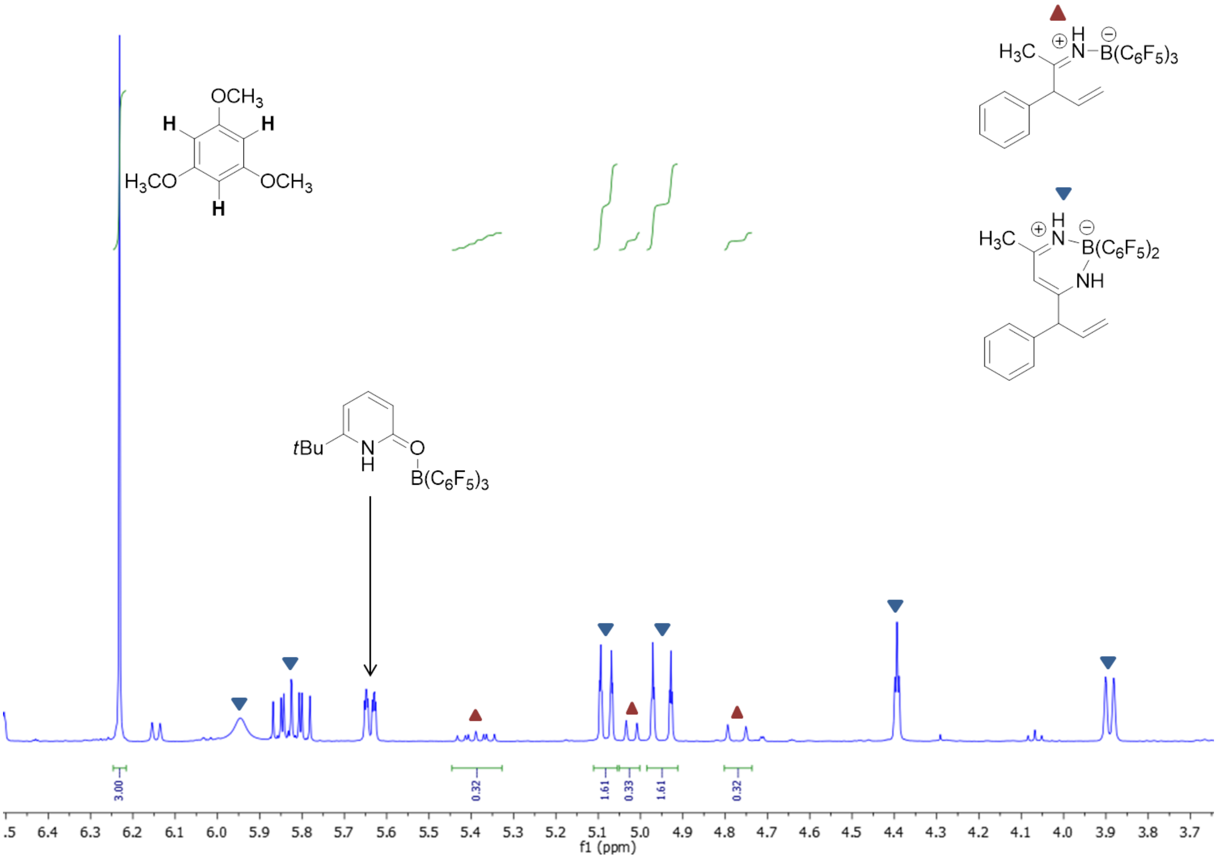

Figure SI 173: Low field excerpt of the ^{1}H NMR spectrum of the reaction mixture after heating allylimine borane complex **13** with one equivalent of acetonitrile and pyridonate borane **3** after heating to 80 °C for 22 h (400 MHz, benzene-d_6)..

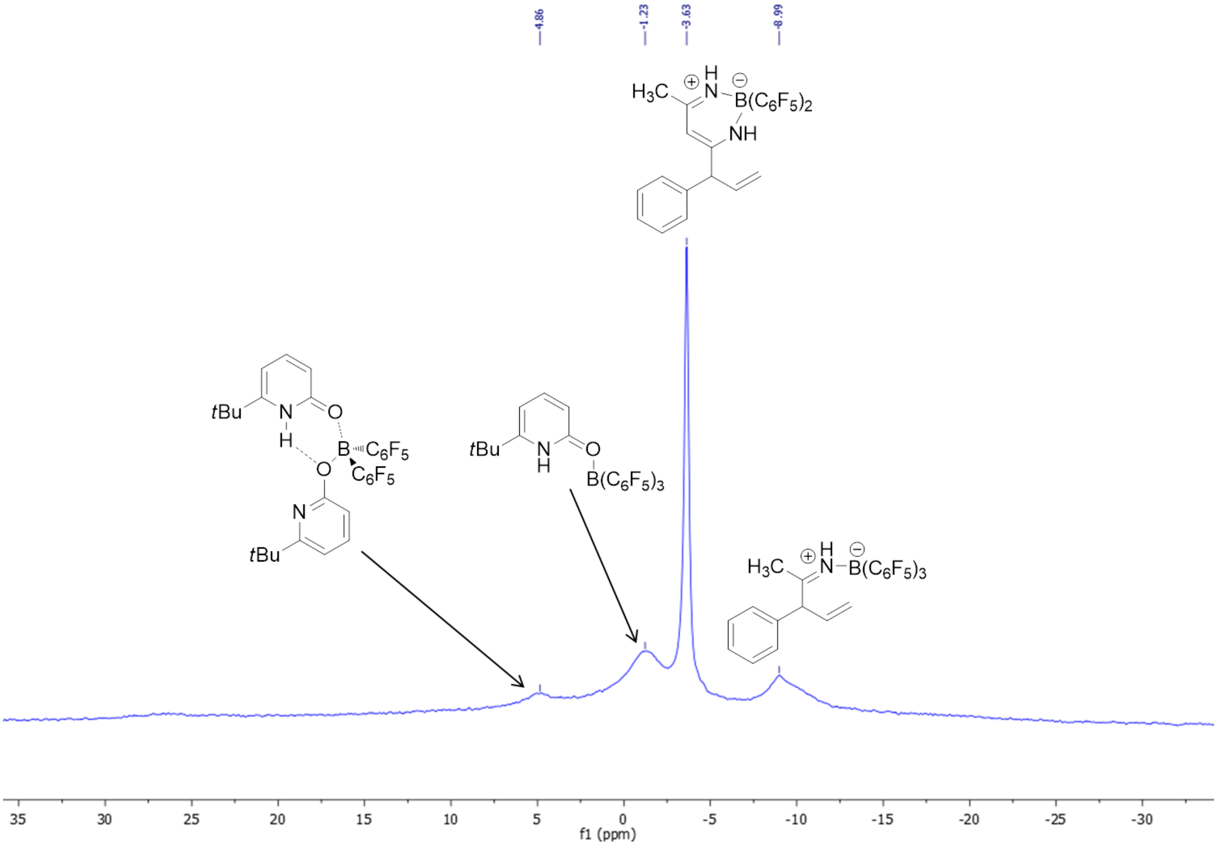

Figure SI 174: ^{11}B NMR spectrum of the reaction mixture after heating allylimine borane complex **13** with one equivalent of acetonitrile and pyridonate borane **3** after heating to 80 °C for about 22 h (128 MHz, benzene-d_6).

7 X-ray analysis

7.1 Crystallographic data collection and processing

All crystals for X-Ray analysis were obtained by concentrating a solution with the respective compound in pure *n*-hexane.

Diffraction data were collected at low temperatures (100 K) using ϕ- and ω-scans on a BRUKER D8 Venture system equipped with dual IµS microfocus sources, a PHOTON100 detector and an OXFORD CRYOSYSTEMS 700 low temperature system. Mo-Kα radiation with wavelength 0.71073 Å, Cu-Kα radiation with wavelength 1.54178 Å and a collimating Quazar multilayer mirror were used. Semi-empirical absorption correction from equivalents was applied using SADABS-2016/2[11] and the structures were solved by the dual space algorithm implemented in SHELXT2014/5.[12] Refinement was performed against F^2 on all data by full-matrix least squares using SHELXL2018/3.[13] All non-hydrogen atoms were refined anisotropically and C-H hydrogen atoms were positioned at geometrically calculated positions and refined using a riding model. N-H hydrogen atoms were located in the difference map and were set to ideal distances. The isotropic displacement parameters of all hydrogen atoms were fixed to 1.2x or 1.5x (CH_3 hydrogens) the U_{eq} value of the atoms they are linked to.

The crystallographic data have been deposited with the Cambridge Crystallographic Data Centre as CCDC No. 2022611 - 2022613 and can be obtained free of charge. (https://www.ccdc.cam.ac.uk/structures/)

7.2 Allylimin trisperfluorphenyl borane complex 13

The crystal structure of allylimin borane complex **13** was solved in the monoclinic space group *C*2/*c* and the asymmetric unit contains one full molecule. The molecule was found to be disordered in two positions. The disorder was refined with the help of same distance restraints, similarity restraints for anisotropic displacement parameters[14] and advanced rigid bond restraints.[15] The two nitrogen atoms with very similar positions were set to the same anisotropic displacement parameters. The two disorder rations were allowed to refine freely and their ratios converged to 0.603(3) and 0.51(3).

Table SI 1: Crystal data and structure refinement for allylimin borane complex **13**.

CCDC No	2022611	
Empirical formula	$C_{29}H_{13}BF_{15}N$	
Formula weight	671.21	
Temperature	100(2) K	
Wavelength	0.71073 Å	
Crystal system	Monoclinic	
Space group	*C*2/*c*	
Unit cell dimensions	a = 26.0032(15) Å	α= 90°.
	b = 15.1799(9) Å	β= 131.4615(16)°.
	c = 17.9257(11) Å	γ = 90°.

Volume	5302.6(6) Å^3
Z	8
Density (calculated)	1.682 Mg/m^3
Absorption coefficient	0.172 mm^{-1}
F(000)	2672
Crystal size	0.575 x 0.078 x 0.072 mm^3
Theta range for data collection	2.090 to 26.731°.
Index ranges	-32 ≤ h ≤ 30, -19 ≤ k ≤ 19, -22 ≤ l ≤ 22
Reflections collected	47957
Independent reflections	5626 [R(int) = 0.0902]
Completeness to theta = 25.242°	99.9 %
Absorption correction	Semi-empirical from equivalents
Refinement method	Full-matrix least-squares on F^2
Data / restraints / parameters	5626 / 1536 / 627
Goodness-of-fit on F^2	1.028
Final R indices [I>2σ(I)]	R1 = 0.0436, wR2 = 0.0960
R indices (all data)	R1 = 0.0686, wR2 = 0.1075
Extinction coefficient	0.00170(16)
Largest diff. peak and hole	0.419 and -0.219 e.Å^{-3}

Table SI 2: Atomic coordinates (x 104) and equivalent isotropic displacement parameters (Å2x 103) for allylimin borane complex **13**. U(eq) is defined as one third of the trace of the orthogonalized Uij tensor.

	x	y	z	U(eq)
N(1)	2382(5)	4578(12)	2772(11)	24(2)
C(1)	2261(7)	3268(11)	1894(10)	30(2)
C(2)	1985(5)	4063(11)	2017(11)	26(2)
C(3)	1218(4)	4250(5)	1199(6)	35(2)
C(4)	1056(2)	4583(3)	267(3)	41(1)
C(5)	1485(2)	4973(3)	223(3)	45(1)
C(6)	963(9)	4865(5)	1560(11)	32(2)
C(7)	740(3)	4519(3)	2019(3)	46(1)
C(8)	556(3)	5064(3)	2427(4)	58(1)
C(9)	598(3)	5965(4)	2393(4)	50(1)
C(10)	800(6)	6321(6)	1931(8)	49(2)
C(11)	983(2)	5781(3)	1508(4)	45(1)
N(1A)	2384(7)	4487(19)	2866(18)	24(2)
C(1A)	2384(10)	3191(15)	2063(15)	35(4)
C(2A)	2053(7)	3941(16)	2138(17)	28(4)
C(3A)	1278(6)	4052(7)	1362(9)	33(2)
C(4A)	934(3)	3252(4)	1370(5)	51(2)
C(5A)	483(4)	2766(5)	594(6)	65(2)
C(6A)	1026(14)	4903(8)	1462(18)	33(3)
C(7A)	965(3)	5000(4)	2166(5)	32(1)
C(8A)	794(4)	5823(5)	2290(6)	38(2)
C(9A)	671(9)	6525(8)	1709(11)	44(3)
C(10A)	703(4)	6427(4)	992(5)	53(2)
C(11A)	895(4)	5620(4)	873(5)	45(2)
B(1)	3187(1)	4584(2)	3747(2)	28(1)
F(21)	2381(1)	3027(1)	3665(1)	36(1)
F(22)	2942(1)	1737(1)	4986(1)	44(1)
F(23)	4314(1)	1680(1)	6546(1)	48(1)
F(24)	5131(1)	2990(1)	6780(1)	47(1)
F(25)	4582(1)	4303(1)	5460(1)	34(1)
C(21)	3462(1)	3719(1)	4472(1)	27(1)
C(22)	3071(1)	3048(1)	4407(2)	30(1)
C(23)	3348(1)	2368(1)	5090(2)	32(1)
C(24)	4042(1)	2341(1)	5884(2)	35(1)
C(25)	4450(1)	3001(1)	5995(2)	33(1)

C(26)	4154(1)	3663(1)	5302(2)	29(1)
F(31)	4145(1)	3393(1)	3761(1)	34(1)
F(32)	4623(1)	3749(1)	2874(1)	38(1)
F(33)	4277(1)	5269(1)	1833(1)	41(1)
F(34)	3443(1)	6448(1)	1724(1)	44(1)
F(35)	2933(1)	6106(1)	2588(1)	37(1)
C(31)	3534(1)	4748(1)	3261(1)	26(1)
C(32)	3963(1)	4178(1)	3285(1)	27(1)
C(33)	4216(1)	4339(1)	2822(1)	28(1)
C(34)	4042(1)	5102(2)	2294(1)	31(1)
C(35)	3613(1)	5696(1)	2234(2)	32(1)
C(36)	3369(1)	5502(1)	2697(2)	29(1)
F(41)	2362(9)	4725(11)	4323(15)	36(2)
F(42)	2379(7)	6032(11)	5342(11)	57(2)
F(43)	3309(7)	7356(8)	6125(9)	57(2)
F(44)	4211(4)	7380(7)	5838(9)	47(2)
F(45)	4195(7)	6112(10)	4790(11)	39(2)
C(41)	3256(11)	5378(12)	4458(17)	24(2)
C(42)	2823(9)	5377(10)	4663(13)	27(2)
C(43)	2826(8)	6037(10)	5200(12)	35(2)
C(44)	3281(8)	6721(9)	5571(11)	37(2)
C(45)	3747(7)	6730(8)	5444(10)	34(2)
C(46)	3730(8)	6053(9)	4907(12)	29(2)
F(41A)	2444(10)	4898(12)	4361(15)	37(2)
F(42A)	2577(9)	6187(12)	5459(10)	60(3)
F(43A)	3496(10)	7511(8)	6104(8)	66(3)
F(44A)	4298(7)	7476(6)	5625(8)	57(3)
F(45A)	4210(8)	6161(10)	4582(11)	39(2)
C(41A)	3302(12)	5465(13)	4366(18)	27(3)
C(42A)	2907(9)	5534(11)	4628(13)	31(2)
C(43A)	2966(9)	6208(10)	5211(11)	37(2)
C(44A)	3450(9)	6843(9)	5570(10)	42(3)
C(45A)	3843(8)	6826(7)	5319(10)	36(2)
C(46A)	3771(8)	6141(8)	4743(11)	28(2)

Table SI 3: Bond lengths [Å] and angles [°] for allylimin borane complex **13**.

N(1)-C(2)	1.288(8)	C(4A)-C(5A)	1.301(8)
N(1)-B(1)	1.609(8)	C(4A)-H(4A)	0.9500
N(1)-H(1)	0.873(19)	C(5A)-H(5AA)	0.9500
C(1)-C(2)	1.493(8)	C(5A)-H(5AB)	0.9500
C(1)-H(1A)	0.9800	C(6A)-C(7A)	1.380(11)
C(1)-H(1B)	0.9800	C(6A)-C(11A)	1.391(12)
C(1)-H(1C)	0.9800	C(7A)-C(8A)	1.394(9)
C(2)-C(3)	1.528(8)	C(7A)-H(7A)	0.9500
C(3)-C(4)	1.514(9)	C(8A)-C(9A)	1.372(12)
C(3)-C(6)	1.518(9)	C(8A)-H(8A)	0.9500
C(3)-H(3)	1.0000	C(9A)-C(10A)	1.350(16)
C(4)-C(5)	1.310(5)	C(9A)-H(9A)	0.9500
C(4)-H(4)	0.9500	C(10A)-C(11A)	1.391(8)
C(5)-H(5A)	0.9500	C(10A)-H(10A)	0.9500
C(5)-H(5B)	0.9500	C(11A)-H(11A)	0.9500
C(6)-C(7)	1.386(9)	B(1)-C(41A)	1.635(14)
C(6)-C(11)	1.397(9)	B(1)-C(31)	1.636(3)
C(7)-C(8)	1.382(6)	B(1)-C(21)	1.641(3)
C(7)-H(7)	0.9500	B(1)-C(41)	1.674(13)
C(8)-C(9)	1.377(7)	F(21)-C(22)	1.352(2)
C(8)-H(8)	0.9500	F(22)-C(23)	1.343(2)
C(9)-C(10)	1.353(12)	F(23)-C(24)	1.342(2)
C(9)-H(9)	0.9500	F(24)-C(25)	1.348(2)
C(10)-C(11)	1.396(11)	F(25)-C(26)	1.358(2)
C(10)-H(10)	0.9500	C(21)-C(26)	1.383(3)
C(11)-H(11)	0.9500	C(21)-C(22)	1.389(3)
N(1A)-C(2A)	1.281(12)	C(22)-C(23)	1.384(3)
N(1A)-B(1)	1.584(12)	C(23)-C(24)	1.371(3)
N(1A)-H(1NA)	0.88(2)	C(24)-C(25)	1.375(3)
C(1A)-C(2A)	1.487(12)	C(25)-C(26)	1.371(3)
C(1A)-H(1AA)	0.9800	F(31)-C(32)	1.355(2)
C(1A)-H(1AB)	0.9800	F(32)-C(33)	1.343(2)
C(1A)-H(1AC)	0.9800	F(33)-C(34)	1.339(2)
C(2A)-C(3A)	1.520(11)	F(34)-C(35)	1.341(2)
C(3A)-C(6A)	1.512(11)	F(35)-C(36)	1.368(2)
C(3A)-C(4A)	1.513(13)	C(31)-C(32)	1.390(3)
C(3A)-H(3A)	1.0000	C(31)-C(36)	1.393(3)

C(32)-C(33)	1.379(3)	C(4)-C(3)-C(6)	112.6(6)
C(33)-C(34)	1.370(3)	C(4)-C(3)-C(2)	110.7(10)
C(34)-C(35)	1.380(3)	C(6)-C(3)-C(2)	111.5(9)
C(35)-C(36)	1.371(3)	C(4)-C(3)-H(3)	107.2
F(41)-C(42)	1.351(9)	C(6)-C(3)-H(3)	107.2
F(42)-C(43)	1.345(9)	C(2)-C(3)-H(3)	107.2
F(43)-C(44)	1.351(9)	C(5)-C(4)-C(3)	126.2(4)
F(44)-C(45)	1.341(8)	C(5)-C(4)-H(4)	116.9
F(45)-C(46)	1.360(8)	C(3)-C(4)-H(4)	116.9
C(41)-C(46)	1.380(9)	C(4)-C(5)-H(5A)	120.0
C(41)-C(42)	1.397(9)	C(4)-C(5)-H(5B)	120.0
C(42)-C(43)	1.385(9)	H(5A)-C(5)-H(5B)	120.0
C(43)-C(44)	1.369(9)	C(7)-C(6)-C(11)	117.7(6)
C(44)-C(45)	1.374(9)	C(7)-C(6)-C(3)	119.5(6)
C(45)-C(46)	1.387(9)	C(11)-C(6)-C(3)	122.6(7)
F(41A)-C(42A)	1.360(9)	C(8)-C(7)-C(6)	120.9(5)
F(42A)-C(43A)	1.347(9)	C(8)-C(7)-H(7)	119.5
F(43A)-C(44A)	1.344(9)	C(6)-C(7)-H(7)	119.5
F(44A)-C(45A)	1.347(9)	C(9)-C(8)-C(7)	120.5(5)
F(45A)-C(46A)	1.352(9)	C(9)-C(8)-H(8)	119.8
C(41A)-C(46A)	1.380(10)	C(7)-C(8)-H(8)	119.8
C(41A)-C(42A)	1.387(10)	C(10)-C(9)-C(8)	119.9(7)
C(42A)-C(43A)	1.396(9)	C(10)-C(9)-H(9)	120.1
C(43A)-C(44A)	1.363(9)	C(8)-C(9)-H(9)	120.1
C(44A)-C(45A)	1.362(9)	C(9)-C(10)-C(11)	120.5(7)
C(45A)-C(46A)	1.388(9)	C(9)-C(10)-H(10)	119.8
		C(11)-C(10)-H(10)	119.8
C(2)-N(1)-B(1)	134.9(11)	C(10)-C(11)-C(6)	120.5(6)
C(2)-N(1)-H(1)	111(3)	C(10)-C(11)-H(11)	119.7
B(1)-N(1)-H(1)	114(3)	C(6)-C(11)-H(11)	119.7
C(2)-C(1)-H(1A)	109.5	C(2A)-N(1A)-B(1)	128.9(17)
C(2)-C(1)-H(1B)	109.5	C(2A)-N(1A)-H(1NA)	119(5)
H(1A)-C(1)-H(1B)	109.5	B(1)-N(1A)-H(1NA)	112(5)
C(2)-C(1)-H(1C)	109.5	C(2A)-C(1A)-H(1AA)	109.5
H(1A)-C(1)-H(1C)	109.5	C(2A)-C(1A)-H(1AB)	109.5
H(1B)-C(1)-H(1C)	109.5	H(1AA)-C(1A)-H(1AB)	109.5
N(1)-C(2)-C(1)	121.1(9)	C(2A)-C(1A)-H(1AC)	109.5
N(1)-C(2)-C(3)	121.7(8)	H(1AA)-C(1A)-H(1AC)	109.5
C(1)-C(2)-C(3)	117.2(8)	H(1AB)-C(1A)-H(1AC)	109.5

N(1A)-C(2A)-C(1A)	123.3(13)
N(1A)-C(2A)-C(3A)	116.8(12)
C(1A)-C(2A)-C(3A)	119.8(12)
C(6A)-C(3A)-C(4A)	112.3(10)
C(6A)-C(3A)-C(2A)	113.7(13)
C(4A)-C(3A)-C(2A)	109.6(14)
C(6A)-C(3A)-H(3A)	106.9
C(4A)-C(3A)-H(3A)	106.9
C(2A)-C(3A)-H(3A)	106.9
C(5A)-C(4A)-C(3A)	124.4(8)
C(5A)-C(4A)-H(4A)	117.8
C(3A)-C(4A)-H(4A)	117.8
C(4A)-C(5A)-H(5AA)	120.0
C(4A)-C(5A)-H(5AB)	120.0
H(5AA)-C(5A)-H(5AB)	120.0
C(7A)-C(6A)-C(11A)	119.5(9)
C(7A)-C(6A)-C(3A)	121.3(10)
C(11A)-C(6A)-C(3A)	119.1(9)
C(6A)-C(7A)-C(8A)	119.0(8)
C(6A)-C(7A)-H(7A)	120.5
C(8A)-C(7A)-H(7A)	120.5
C(9A)-C(8A)-C(7A)	120.8(9)
C(9A)-C(8A)-H(8A)	119.6
C(7A)-C(8A)-H(8A)	119.6
C(10A)-C(9A)-C(8A)	120.6(11)
C(10A)-C(9A)-H(9A)	119.7
C(8A)-C(9A)-H(9A)	119.7
C(9A)-C(10A)-C(11A)	119.8(7)
C(9A)-C(10A)-H(10A)	120.1
C(11A)-C(10A)-H(10A)	120.1
C(10A)-C(11A)-C(6A)	120.3(7)
C(10A)-C(11A)-H(11A)	119.9
C(6A)-C(11A)-H(11A)	119.9
N(1A)-B(1)-C(41A)	106.3(12)
N(1A)-B(1)-C(31)	108.1(14)
N(1)-B(1)-C(31)	101.7(8)
C(41A)-B(1)-C(31)	108.6(6)
N(1A)-B(1)-C(21)	108.2(13)
N(1)-B(1)-C(21)	115.3(8)
C(41A)-B(1)-C(21)	109.2(10)
C(31)-B(1)-C(21)	115.97(17)
N(1)-B(1)-C(41)	103.7(10)
C(31)-B(1)-C(41)	118.5(5)
C(21)-B(1)-C(41)	101.7(9)
C(26)-C(21)-C(22)	113.84(18)
C(26)-C(21)-B(1)	118.40(17)
C(22)-C(21)-B(1)	127.52(18)
F(21)-C(22)-C(23)	115.29(17)
F(21)-C(22)-C(21)	121.34(18)
C(23)-C(22)-C(21)	123.36(19)
F(22)-C(23)-C(24)	119.68(19)
F(22)-C(23)-C(22)	120.56(18)
C(24)-C(23)-C(22)	119.76(19)
F(23)-C(24)-C(23)	120.07(19)
F(23)-C(24)-C(25)	120.77(19)
C(23)-C(24)-C(25)	119.16(19)
F(24)-C(25)-C(26)	121.02(19)
F(24)-C(25)-C(24)	119.80(19)
C(26)-C(25)-C(24)	119.18(19)
F(25)-C(26)-C(25)	116.45(18)
F(25)-C(26)-C(21)	118.90(18)
C(25)-C(26)-C(21)	124.66(19)
C(32)-C(31)-C(36)	112.90(18)
C(32)-C(31)-B(1)	127.10(17)
C(36)-C(31)-B(1)	119.86(17)
F(31)-C(32)-C(33)	115.32(17)
F(31)-C(32)-C(31)	120.38(17)
C(33)-C(32)-C(31)	124.27(18)
F(32)-C(33)-C(34)	119.79(18)
F(32)-C(33)-C(32)	120.55(17)
C(34)-C(33)-C(32)	119.65(18)
F(33)-C(34)-C(33)	120.63(19)
F(33)-C(34)-C(35)	120.22(19)
C(33)-C(34)-C(35)	119.14(19)
F(34)-C(35)-C(36)	121.32(19)
F(34)-C(35)-C(34)	119.63(19)
C(36)-C(35)-C(34)	119.05(18)
F(35)-C(36)-C(35)	116.24(17)

F(35)-C(36)-C(31)	118.81(18)	C(41)-C(46)-C(45)	124.2(9)
C(35)-C(36)-C(31)	124.95(19)	C(46A)-C(41A)-C(42A)	113.0(10)
C(46)-C(41)-C(42)	114.2(9)	C(46A)-C(41A)-B(1)	130.2(10)
C(46)-C(41)-B(1)	125.7(8)	C(42A)-C(41A)-B(1)	116.6(9)
C(42)-C(41)-B(1)	120.1(8)	F(41A)-C(42A)-C(41A)	120.6(11)
F(41)-C(42)-C(43)	116.1(10)	F(41A)-C(42A)-C(43A)	114.6(10)
F(41)-C(42)-C(41)	120.7(11)	C(41A)-C(42A)-C(43A)	124.7(9)
C(43)-C(42)-C(41)	123.2(9)	F(42A)-C(43A)-C(44A)	121.5(8)
F(42)-C(43)-C(44)	119.3(8)	F(42A)-C(43A)-C(42A)	119.6(9)
F(42)-C(43)-C(42)	121.2(9)	C(44A)-C(43A)-C(42A)	118.8(8)
C(44)-C(43)-C(42)	119.5(8)	F(43A)-C(44A)-C(45A)	121.2(8)
F(43)-C(44)-C(43)	120.4(8)	F(43A)-C(44A)-C(43A)	119.2(9)
F(43)-C(44)-C(45)	119.5(8)	C(45A)-C(44A)-C(43A)	119.4(8)
C(43)-C(44)-C(45)	120.0(8)	F(44A)-C(45A)-C(44A)	120.0(8)
F(44)-C(45)-C(44)	120.3(8)	F(44A)-C(45A)-C(46A)	120.1(8)
F(44)-C(45)-C(46)	121.0(8)	C(44A)-C(45A)-C(46A)	119.9(8)
C(44)-C(45)-C(46)	118.7(8)	F(45A)-C(46A)-C(41A)	120.6(10)
F(45)-C(46)-C(41)	120.0(9)	F(45A)-C(46A)-C(45A)	115.2(9)
F(45)-C(46)-C(45)	115.7(9)	C(41A)-C(46A)-C(45A)	124.2(9)

Table SI 4: Anisotropic displacement parameters (Å2x 103) for allylimin borane complex **13**. The anisotropic displacement factor exponent takes the form: -2p2[h2 a*2U11 + ... + 2 h k a* b* U12]

	U^{11}	U^{22}	U^{33}	U^{23}	U^{13}	U^{12}
N(1)	32(1)	22(3)	26(3)	1(3)	22(1)	-3(1)
C(1)	28(3)	28(4)	23(3)	1(2)	13(2)	-1(2)
C(2)	35(3)	21(4)	23(3)	3(3)	19(2)	-2(2)
C(3)	26(2)	34(3)	30(3)	-1(2)	12(2)	-8(2)
C(4)	32(2)	47(2)	24(2)	5(2)	10(2)	-2(2)
C(5)	44(2)	51(2)	29(2)	10(2)	19(2)	-4(2)
C(6)	26(4)	24(3)	27(4)	1(2)	10(2)	-2(2)
C(7)	63(3)	28(2)	50(2)	5(2)	39(2)	-1(2)
C(8)	93(4)	43(2)	65(3)	13(2)	63(3)	10(2)
C(9)	60(4)	39(3)	52(3)	1(2)	37(3)	3(2)
C(10)	56(5)	25(4)	61(5)	2(3)	37(5)	1(3)
C(11)	46(2)	32(2)	59(3)	6(2)	35(2)	-5(2)
N(1A)	32(1)	22(3)	26(3)	1(3)	22(1)	-3(1)
C(1A)	60(10)	23(6)	45(8)	-11(6)	44(8)	-11(6)
C(2A)	33(4)	27(7)	31(6)	-2(5)	24(4)	-10(4)
C(3A)	42(4)	36(5)	31(4)	-10(4)	28(4)	-6(3)
C(4A)	41(3)	34(3)	54(4)	-1(3)	22(3)	1(3)
C(5A)	62(4)	70(5)	71(5)	-32(4)	47(4)	-28(4)
C(6A)	30(5)	31(4)	31(5)	-5(3)	17(4)	-6(3)
C(7A)	28(3)	28(3)	34(3)	-3(2)	18(2)	-3(2)
C(8A)	34(4)	40(4)	43(4)	-3(3)	27(3)	1(3)
C(9A)	45(6)	30(6)	53(6)	-1(4)	31(6)	-1(4)
C(10A)	81(5)	25(3)	47(4)	3(3)	40(4)	0(3)
C(11A)	69(4)	35(3)	41(3)	-4(3)	40(3)	-5(3)
B(1)	30(1)	22(1)	32(1)	-5(1)	21(1)	-3(1)
F(21)	31(1)	38(1)	36(1)	1(1)	22(1)	-6(1)
F(22)	46(1)	38(1)	42(1)	3(1)	28(1)	-11(1)
F(23)	53(1)	35(1)	33(1)	7(1)	19(1)	-5(1)
F(24)	38(1)	40(1)	34(1)	4(1)	11(1)	-6(1)
F(25)	33(1)	28(1)	34(1)	-3(1)	19(1)	-7(1)
C(21)	32(1)	26(1)	29(1)	-6(1)	23(1)	-2(1)
C(22)	32(1)	31(1)	28(1)	-4(1)	21(1)	-3(1)
C(23)	40(1)	28(1)	33(1)	-6(1)	25(1)	-10(1)
C(24)	46(1)	26(1)	28(1)	0(1)	22(1)	-2(1)

C(25)	32(1)	32(1)	24(1)	-5(1)	13(1)	-4(1)
C(26)	35(1)	23(1)	31(1)	-7(1)	23(1)	-6(1)
F(31)	49(1)	24(1)	41(1)	7(1)	34(1)	9(1)
F(32)	38(1)	44(1)	39(1)	5(1)	29(1)	9(1)
F(33)	34(1)	57(1)	35(1)	11(1)	23(1)	-2(1)
F(34)	34(1)	39(1)	44(1)	20(1)	20(1)	3(1)
F(35)	35(1)	25(1)	46(1)	3(1)	25(1)	3(1)
C(31)	26(1)	22(1)	28(1)	-4(1)	16(1)	-3(1)
C(32)	29(1)	22(1)	28(1)	2(1)	18(1)	0(1)
C(33)	24(1)	32(1)	26(1)	1(1)	15(1)	2(1)
C(34)	21(1)	42(1)	25(1)	2(1)	13(1)	-6(1)
C(35)	22(1)	30(1)	31(1)	7(1)	11(1)	-3(1)
C(36)	22(1)	23(1)	33(1)	1(1)	15(1)	1(1)
F(41)	38(3)	35(5)	41(3)	-3(3)	30(3)	-6(3)
F(42)	60(5)	76(4)	56(4)	-5(3)	48(4)	14(4)
F(43)	70(4)	48(4)	47(2)	-12(2)	35(3)	17(3)
F(44)	48(2)	26(3)	47(4)	-16(2)	23(2)	-7(2)
F(45)	39(2)	32(3)	47(5)	-15(3)	29(3)	-11(2)
C(41)	29(4)	21(4)	24(4)	0(4)	19(3)	1(3)
C(42)	36(4)	25(5)	31(3)	6(3)	27(3)	7(3)
C(43)	38(5)	38(4)	38(3)	5(3)	28(3)	10(3)
C(44)	38(5)	34(5)	33(3)	-6(3)	21(4)	12(3)
C(45)	37(4)	29(4)	25(4)	-8(3)	16(3)	-5(3)
C(46)	33(3)	28(4)	28(5)	2(3)	20(3)	4(3)
F(41A)	45(4)	35(6)	48(3)	-6(3)	38(3)	-9(3)
F(42A)	65(6)	78(6)	51(3)	5(3)	44(4)	30(4)
F(43A)	89(7)	41(4)	37(2)	-9(2)	28(4)	26(4)
F(44A)	64(4)	26(2)	44(3)	-7(2)	19(2)	-10(2)
F(45A)	36(2)	36(2)	40(4)	-7(3)	23(2)	-9(2)
C(41A)	33(4)	25(4)	24(4)	3(3)	20(3)	5(3)
C(42A)	38(5)	26(4)	23(3)	3(3)	18(3)	0(3)
C(43A)	44(6)	39(5)	32(3)	1(4)	27(4)	15(3)
C(44A)	52(7)	29(4)	26(3)	-6(2)	18(4)	9(4)
C(45A)	41(4)	21(3)	29(4)	4(3)	16(3)	6(3)
C(46A)	38(4)	20(3)	20(4)	1(3)	17(3)	-1(2)

Table SI 5: Hydrogen coordinates (x 104) and isotropic displacement parameters (Å2x 10 3) for allylimin borane complex **13**.

	x	y	z	U(eq)
H(1)	2160(20)	5030(20)	2730(30)	29
H(1A)	2073	2736	1948	44
H(1B)	2762	3262	2415	44
H(1C)	2128	3284	1239	44
H(3)	972	3676	1026	42
H(4)	600	4501	-345	49
H(5A)	1947	5070	815	54
H(5B)	1334	5160	-400	54
H(7)	714	3898	2054	55
H(8)	401	4815	2733	70
H(9)	484	6336	2692	60
H(10)	818	6944	1894	58
H(11)	1122	6039	1183	54
H(1NA)	2150(30)	4860(30)	2920(50)	29
H(1AA)	2084	2674	1799	53
H(1AB)	2824	3055	2725	53
H(1AC)	2464	3350	1617	53
H(3A)	1148	4065	698	40
H(4A)	1056	3093	1982	61
H(5AA)	349	2906	-30	78
H(5AB)	287	2272	652	78
H(7A)	1039	4513	2560	39
H(8A)	761	5900	2784	46
H(9A)	562	7084	1811	53
H(10A)	596	6908	571	63
H(11A)	937	5557	388	55

Table SI 6: Hydrogen bonds for allylimin borane complex **13** [Å and °].

D-H...A	d(D-H)	d(H...A)	d(D...A)	<(DHA)
N(1A^b)-H(1NA^b)...F(41A^b)	0.88(2)	2.16(6)	2.65(4)	115(5)

7.3 β-diketiminate borane complex 9

The crystal structure of β-diketiminate **9** was solved in the orthorhombic space group Aba2. The asymmetric unit contains one molecule of β-diketiminate **9**, exhibiting full molecule disorder. The disorder was modelled using same distance restraints, strong similarity restraints on anisotropic displacement parameters and advanced rigid bond restraints. Some of the atoms were set to the same anisotropic displacement parameters to allow for anisotropic refinement of all non-hydrogen atoms. The disorder ratio was refined and converged to 0.57(2). The data showed indication for inversion twinning, however, data quality did not allow for reliable determination of the twin ratios.

Table SI 7: Crystal data and structure refinement for β-diketiminate borane complex **9**.

CCDC No	2022612	
Empirical formula	$C_{25} H_{15} B F_{10} N_2$	
Formula weight	544.20	
Temperature	100(2) K	
Wavelength	1.54178 Å	
Crystal system	Orthorhombic	
Space group	*Aba*2	
Unit cell dimensions	a = 14.5842(3) Å	α= 90°.
	b = 16.3724(4) Å	β= 90°.
	c = 18.7547(5) Å	γ = 90°.
Volume	4478.22(19) $Å^3$	
Z	8	
Density (calculated)	1.614 Mg/m^3	
Absorption coefficient	1.362 mm^{-1}	
F(000)	2192	
Crystal size	0.212 x 0.190 x 0.081 mm^3	
Theta range for data collection	4.695 to 74.415°.	
Index ranges	-18 ≤ h ≤ 18, -20 ≤ k ≤ 20, -23 ≤ l ≤ 22	
Reflections collected	23809	
Independent reflections	4466 [R(int) = 0.0346]	
Completeness to theta = 67.679°	100.0 %	
Absorption correction	Semi-empirical from equivalents	
Refinement method	Full-matrix least-squares on F^2	
Data / restraints / parameters	4466 / 1969 / 659	
Goodness-of-fit on F^2	1.143	
Final R indices [I>2σ(I)]	R1 = 0.0764, wR2 = 0.1880	
R indices (all data)	R1 = 0.0767, wR2 = 0.1881	
Absolute structure parameter	0.3(4)	
Largest diff. peak and hole	0.446 and -0.434 e.$Å^{-3}$	

Table SI 8: Atomic coordinates (x 104) and equivalent isotropic displacement parameters (Å2x 103) for β-diketiminate borane complex **9**. U(eq) is defined as one third of the trace of the orthogonalized Uij tensor.

	x	y	z	U(eq)
F(21)	1310(30)	5930(20)	4530(12)	30(5)
F(22)	0(14)	6927(14)	4988(8)	30(4)
F(23)	-476(15)	6976(12)	6392(9)	33(4)
F(24)	358(13)	5912(11)	7327(8)	22(3)
F(25)	1560(20)	4827(16)	6868(14)	22(4)
F(31)	3396(17)	5441(13)	6390(10)	20(4)
F(32)	4991(15)	6222(13)	6219(9)	30(4)
F(33)	5680(12)	6496(11)	4889(8)	23(2)
F(34)	4765(15)	5900(12)	3737(8)	29(4)
F(35)	3208(19)	5069(14)	3892(11)	23(4)
N(1)	2540(15)	3989(11)	5868(11)	17(3)
N(2)	1900(20)	4209(11)	4678(11)	19(3)
C(1)	2710(30)	2624(17)	6357(12)	23(5)
C(2)	2460(20)	3186(12)	5758(9)	16(3)
C(3)	2172(13)	2876(11)	5097(9)	16(3)
C(4)	1907(14)	3417(10)	4564(8)	16(3)
C(5)	1563(10)	3075(10)	3863(8)	19(1)
C(6)	1074(10)	3718(10)	3410(8)	21(3)
C(7)	1402(14)	4080(11)	2847(10)	32(4)
C(8)	2323(11)	2625(10)	3471(9)	19(1)
C(9)	3190(13)	2911(12)	3396(12)	21(2)
C(10)	3850(14)	2441(13)	3037(16)	21(3)
C(11)	3630(15)	1693(14)	2754(16)	19(3)
C(12)	2731(14)	1398(13)	2836(14)	21(3)
C(13)	2082(12)	1896(12)	3148(14)	19(1)
C(21)	1500(20)	5297(19)	5657(12)	18(2)
C(22)	1050(20)	5850(20)	5227(12)	21(3)
C(23)	392(18)	6417(15)	5457(10)	21(3)
C(24)	159(16)	6442(14)	6152(10)	20(3)
C(25)	560(18)	5880(15)	6631(10)	20(3)
C(26)	1230(20)	5350(20)	6377(12)	19(3)
C(31)	3212(18)	5220(20)	5152(10)	15(2)
C(32)	3722(16)	5540(16)	5726(10)	16(3)
C(33)	4537(14)	5981(13)	5641(10)	19(3)

C(34)	4876(14)	6102(14)	4974(11)	23(2)
C(35)	4409(15)	5787(16)	4403(10)	21(3)
C(36)	3590(18)	5380(20)	4499(10)	17(3)
B(1)	2278(14)	4657(11)	5337(11)	18(2)
F(21A)	1340(40)	5800(30)	4402(15)	24(5)
F(22A)	117(19)	6928(18)	4750(11)	27(4)
F(23A)	-460(20)	7091(17)	6145(12)	30(5)
F(24A)	239(18)	6093(15)	7159(10)	20(4)
F(25A)	1540(30)	5010(20)	6834(18)	20(5)
F(31A)	3410(20)	5243(18)	6509(14)	23(5)
F(32A)	5010(20)	6077(18)	6424(10)	29(5)
F(33A)	5683(15)	6511(15)	5140(10)	25(4)
F(34A)	4790(20)	6092(18)	3919(11)	31(5)
F(35A)	3210(20)	5270(20)	3989(15)	24(5)
N(1A)	2380(20)	3959(15)	5846(15)	17(3)
N(2A)	1950(30)	4320(14)	4626(15)	19(3)
C(1A)	2700(40)	2560(20)	6166(16)	21(6)
C(2A)	2480(30)	3189(16)	5614(13)	16(3)
C(3A)	2299(19)	2963(15)	4910(12)	16(3)
C(4A)	1990(20)	3555(14)	4430(11)	17(3)
C(5A)	1709(14)	3299(13)	3686(10)	20(3)
C(6A)	1515(15)	4055(14)	3223(14)	27(4)
C(7A)	722(15)	4399(15)	3146(13)	34(5)
C(8A)	2438(15)	2763(12)	3342(12)	19(1)
C(9A)	3306(17)	3047(16)	3288(16)	21(2)
C(10A)	3988(19)	2577(18)	2990(20)	21(3)
C(11A)	3820(20)	1818(19)	2730(20)	20(4)
C(12A)	2920(20)	1507(17)	2790(20)	20(4)
C(13A)	2248(15)	1944(16)	3171(18)	19(1)
C(21A)	1480(30)	5350(30)	5593(15)	19(3)
C(22A)	1100(30)	5870(30)	5106(16)	20(3)
C(23A)	440(20)	6460(20)	5275(13)	21(3)
C(24A)	150(20)	6517(19)	5951(13)	20(3)
C(25A)	520(20)	6017(18)	6490(13)	18(3)
C(26A)	1160(30)	5430(30)	6291(15)	19(3)
C(31A)	3240(20)	5210(30)	5251(13)	16(3)
C(32A)	3720(20)	5450(20)	5862(14)	18(3)
C(33A)	4548(18)	5876(18)	5830(12)	17(3)
C(34A)	4893(18)	6104(18)	5191(12)	19(3)

C(35A)	4440(20)	5890(20)	4568(12)	19(3)
C(36A)	3640(20)	5440(30)	4625(14)	20(3)
B(1A)	2270(17)	4677(14)	5341(14)	18(2)

Table SI 9: Bond lengths [Å] and angles [°] for β-diketiminate borane complex **9**.

F(21)-C(22)	1.367(18)	C(11)-C(12)	1.41(2)
F(22)-C(23)	1.342(17)	C(11)-H(11)	0.9500
F(23)-C(24)	1.352(15)	C(12)-C(13)	1.380(18)
F(24)-C(25)	1.338(17)	C(12)-H(12)	0.9500
F(25)-C(26)	1.346(17)	C(13)-H(13)	0.9500
F(31)-C(32)	1.344(17)	C(21)-C(22)	1.374(17)
F(32)-C(33)	1.330(17)	C(21)-C(26)	1.410(17)
F(33)-C(34)	1.347(16)	C(21)-B(1)	1.659(17)
F(34)-C(35)	1.365(17)	C(22)-C(23)	1.408(17)
F(35)-C(36)	1.366(17)	C(23)-C(24)	1.35(2)
N(1)-C(2)	1.336(17)	C(24)-C(25)	1.41(2)
N(1)-B(1)	1.528(17)	C(25)-C(26)	1.390(17)
N(1)-H(1)	0.89(3)	C(31)-C(36)	1.370(17)
N(2)-C(4)	1.315(17)	C(31)-C(32)	1.411(17)
N(2)-B(1)	1.539(17)	C(31)-B(1)	1.677(17)
N(2)-H(2)	0.88(3)	C(32)-C(33)	1.399(17)
C(1)-C(2)	1.500(19)	C(33)-C(34)	1.36(2)
C(1)-H(1A)	0.9800	C(34)-C(35)	1.37(2)
C(1)-H(1B)	0.9800	C(35)-C(36)	1.380(17)
C(1)-H(1C)	0.9800	F(21A)-C(22A)	1.37(2)
C(2)-C(3)	1.403(19)	F(22A)-C(23A)	1.34(2)
C(3)-C(4)	1.390(19)	F(23A)-C(24A)	1.34(2)
C(3)-H(3)	0.9500	F(24A)-C(25A)	1.33(2)
C(4)-C(5)	1.515(17)	F(25A)-C(26A)	1.35(2)
C(5)-C(8)	1.520(17)	F(31A)-C(32A)	1.34(2)
C(5)-C(6)	1.53(2)	F(32A)-C(33A)	1.35(2)
C(5)-H(5)	1.0000	F(33A)-C(34A)	1.334(18)
C(6)-C(7)	1.30(2)	F(34A)-C(35A)	1.36(2)
C(6)-H(6)	0.9500	F(35A)-C(36A)	1.38(2)
C(7)-H(7A)	0.9500	N(1A)-C(2A)	1.34(2)
C(7)-H(7B)	0.9500	N(1A)-B(1A)	1.52(2)
C(8)-C(9)	1.356(17)	N(1A)-H(1AN)	0.88(3)
C(8)-C(13)	1.384(18)	N(2A)-C(4A)	1.31(2)
C(9)-C(10)	1.405(18)	N(2A)-B(1A)	1.53(2)
C(9)-H(9)	0.9500	N(2A)-H(2A)	0.89(3)
C(10)-C(11)	1.374(19)	C(1A)-C(2A)	1.50(2)
C(10)-H(10)	0.9500	C(1A)-H(1AA)	0.9800

C(1A)-H(1AB)	0.9800	C(2)-N(1)-B(1)	125.5(15)
C(1A)-H(1AC)	0.9800	C(2)-N(1)-H(1)	115(7)
C(2A)-C(3A)	1.39(2)	B(1)-N(1)-H(1)	119(7)
C(3A)-C(4A)	1.40(2)	C(4)-N(2)-B(1)	126.7(15)
C(3A)-H(3A)	0.9500	C(4)-N(2)-H(2)	108(9)
C(4A)-C(5A)	1.51(2)	B(1)-N(2)-H(2)	122(7)
C(5A)-C(8A)	1.52(2)	C(2)-C(1)-H(1A)	109.5
C(5A)-C(6A)	1.54(3)	C(2)-C(1)-H(1B)	109.5
C(5A)-H(5A)	1.0000	H(1A)-C(1)-H(1B)	109.5
C(6A)-C(7A)	1.29(2)	C(2)-C(1)-H(1C)	109.5
C(6A)-H(6A)	0.9500	H(1A)-C(1)-H(1C)	109.5
C(7A)-H(7AA)	0.9500	H(1B)-C(1)-H(1C)	109.5
C(7A)-H(7AB)	0.9500	N(1)-C(2)-C(3)	121.3(15)
C(8A)-C(9A)	1.35(2)	N(1)-C(2)-C(1)	117.7(17)
C(8A)-C(13A)	1.41(2)	C(3)-C(2)-C(1)	120.9(15)
C(9A)-C(10A)	1.38(2)	C(4)-C(3)-C(2)	119.2(14)
C(9A)-H(9A)	0.9500	C(4)-C(3)-H(3)	120.4
C(10A)-C(11A)	1.36(2)	C(2)-C(3)-H(3)	120.4
C(10A)-H(10A)	0.9500	N(2)-C(4)-C(3)	120.9(15)
C(11A)-C(12A)	1.41(2)	N(2)-C(4)-C(5)	120.2(14)
C(11A)-H(11A)	0.9500	C(3)-C(4)-C(5)	118.8(13)
C(12A)-C(13A)	1.41(2)	C(4)-C(5)-C(8)	111.0(12)
C(12A)-H(12A)	0.9500	C(4)-C(5)-C(6)	112.5(13)
C(13A)-H(13A)	0.9500	C(8)-C(5)-C(6)	113.9(12)
C(21A)-C(22A)	1.37(2)	C(4)-C(5)-H(5)	106.3
C(21A)-C(26A)	1.40(2)	C(8)-C(5)-H(5)	106.3
C(21A)-B(1A)	1.65(2)	C(6)-C(5)-H(5)	106.3
C(22A)-C(23A)	1.40(2)	C(7)-C(6)-C(5)	126.3(15)
C(23A)-C(24A)	1.34(2)	C(7)-C(6)-H(6)	116.8
C(24A)-C(25A)	1.41(2)	C(5)-C(6)-H(6)	116.8
C(25A)-C(26A)	1.39(2)	C(6)-C(7)-H(7A)	120.0
C(31A)-C(36A)	1.36(2)	C(6)-C(7)-H(7B)	120.0
C(31A)-C(32A)	1.40(2)	H(7A)-C(7)-H(7B)	120.0
C(31A)-B(1A)	1.67(2)	C(9)-C(8)-C(13)	119.3(14)
C(32A)-C(33A)	1.39(2)	C(9)-C(8)-C(5)	124.2(14)
C(33A)-C(34A)	1.35(2)	C(13)-C(8)-C(5)	116.4(12)
C(34A)-C(35A)	1.39(2)	C(8)-C(9)-C(10)	120.0(15)
C(35A)-C(36A)	1.38(2)	C(8)-C(9)-H(9)	120.0
		C(10)-C(9)-H(9)	120.0

C(11)-C(10)-C(9)	120.9(14)	F(33)-C(34)-C(33)	119.7(15)
C(11)-C(10)-H(10)	119.5	F(33)-C(34)-C(35)	121.3(15)
C(9)-C(10)-H(10)	119.5	C(33)-C(34)-C(35)	118.9(14)
C(10)-C(11)-C(12)	118.8(15)	F(34)-C(35)-C(34)	118.3(14)
C(10)-C(11)-H(11)	120.6	F(34)-C(35)-C(36)	121.0(15)
C(12)-C(11)-H(11)	120.6	C(34)-C(35)-C(36)	120.6(15)
C(13)-C(12)-C(11)	118.9(15)	F(35)-C(36)-C(31)	120.6(15)
C(13)-C(12)-H(12)	120.6	F(35)-C(36)-C(35)	115.0(16)
C(11)-C(12)-H(12)	120.6	C(31)-C(36)-C(35)	124.1(15)
C(12)-C(13)-C(8)	121.4(14)	N(1)-B(1)-N(2)	105.8(13)
C(12)-C(13)-H(13)	119.3	N(1)-B(1)-C(21)	112.8(14)
C(8)-C(13)-H(13)	119.3	N(2)-B(1)-C(21)	110.2(16)
C(22)-C(21)-C(26)	113.0(14)	N(1)-B(1)-C(31)	108.8(15)
C(22)-C(21)-B(1)	121.8(14)	N(2)-B(1)-C(31)	112.7(15)
C(26)-C(21)-B(1)	125.2(14)	C(21)-B(1)-C(31)	106.6(15)
F(21)-C(22)-C(21)	120.0(17)	C(2A)-N(1A)-B(1A)	122(2)
F(21)-C(22)-C(23)	114.3(16)	C(2A)-N(1A)-H(1AN)	115(8)
C(21)-C(22)-C(23)	125.3(15)	B(1A)-N(1A)-H(1AN)	122(8)
F(22)-C(23)-C(24)	120.6(14)	C(4A)-N(2A)-B(1A)	127(2)
F(22)-C(23)-C(22)	120.1(15)	C(4A)-N(2A)-H(2A)	111(9)
C(24)-C(23)-C(22)	119.3(15)	B(1A)-N(2A)-H(2A)	121(7)
C(23)-C(24)-F(23)	120.9(15)	C(2A)-C(1A)-H(1AA)	109.5
C(23)-C(24)-C(25)	119.5(13)	C(2A)-C(1A)-H(1AB)	109.5
F(23)-C(24)-C(25)	119.5(15)	H(1AA)-C(1A)-H(1AB)	109.5
F(24)-C(25)-C(26)	120.8(15)	C(2A)-C(1A)-H(1AC)	109.5
F(24)-C(25)-C(24)	120.3(14)	H(1AA)-C(1A)-H(1AC)	109.5
C(26)-C(25)-C(24)	118.6(15)	H(1AB)-C(1A)-H(1AC)	109.5
F(25)-C(26)-C(25)	114.5(16)	N(1A)-C(2A)-C(3A)	122(2)
F(25)-C(26)-C(21)	121.1(16)	N(1A)-C(2A)-C(1A)	116(2)
C(25)-C(26)-C(21)	124.2(15)	C(3A)-C(2A)-C(1A)	120.9(19)
C(36)-C(31)-C(32)	113.3(13)	C(2A)-C(3A)-C(4A)	119.1(18)
C(36)-C(31)-B(1)	128.2(14)	C(2A)-C(3A)-H(3A)	120.5
C(32)-C(31)-B(1)	118.4(13)	C(4A)-C(3A)-H(3A)	120.5
F(31)-C(32)-C(33)	117.9(15)	N(2A)-C(4A)-C(3A)	119.7(18)
F(31)-C(32)-C(31)	118.4(15)	N(2A)-C(4A)-C(5A)	121.0(19)
C(33)-C(32)-C(31)	123.7(15)	C(3A)-C(4A)-C(5A)	119.3(17)
F(32)-C(33)-C(34)	121.7(14)	C(4A)-C(5A)-C(8A)	111.2(16)
F(32)-C(33)-C(32)	118.9(15)	C(4A)-C(5A)-C(6A)	110.3(16)
C(34)-C(33)-C(32)	119.2(14)	C(8A)-C(5A)-C(6A)	110.7(17)

C(4A)-C(5A)-H(5A)	108.2	C(24A)-C(23A)-C(22A)	118.6(18)
C(8A)-C(5A)-H(5A)	108.2	C(23A)-C(24A)-F(23A)	121.0(19)
C(6A)-C(5A)-H(5A)	108.2	C(23A)-C(24A)-C(25A)	121.2(17)
C(7A)-C(6A)-C(5A)	125(2)	F(23A)-C(24A)-C(25A)	117.7(19)
C(7A)-C(6A)-H(6A)	117.4	F(24A)-C(25A)-C(26A)	122.0(19)
C(5A)-C(6A)-H(6A)	117.4	F(24A)-C(25A)-C(24A)	120.4(17)
C(6A)-C(7A)-H(7AA)	120.0	C(26A)-C(25A)-C(24A)	117.7(18)
C(6A)-C(7A)-H(7AB)	120.0	F(25A)-C(26A)-C(25A)	115(2)
H(7AA)-C(7A)-H(7AB)	120.0	F(25A)-C(26A)-C(21A)	121(2)
C(9A)-C(8A)-C(13A)	119.7(17)	C(25A)-C(26A)-C(21A)	123.3(18)
C(9A)-C(8A)-C(5A)	119.2(17)	C(36A)-C(31A)-C(32A)	114.2(17)
C(13A)-C(8A)-C(5A)	120.5(16)	C(36A)-C(31A)-B(1A)	126.4(17)
C(8A)-C(9A)-C(10A)	120.9(19)	C(32A)-C(31A)-B(1A)	119.3(17)
C(8A)-C(9A)-H(9A)	119.5	F(31A)-C(32A)-C(33A)	117(2)
C(10A)-C(9A)-H(9A)	119.5	F(31A)-C(32A)-C(31A)	120(2)
C(11A)-C(10A)-C(9A)	121(2)	C(33A)-C(32A)-C(31A)	122.7(19)
C(11A)-C(10A)-H(10A)	119.3	F(32A)-C(33A)-C(34A)	118.5(17)
C(9A)-C(10A)-H(10A)	119.3	F(32A)-C(33A)-C(32A)	121.6(19)
C(10A)-C(11A)-C(12A)	119(2)	C(34A)-C(33A)-C(32A)	119.9(18)
C(10A)-C(11A)-H(11A)	120.7	F(33A)-C(34A)-C(33A)	121.5(17)
C(12A)-C(11A)-H(11A)	120.7	F(33A)-C(34A)-C(35A)	118.5(17)
C(11A)-C(12A)-C(13A)	119.9(19)	C(33A)-C(34A)-C(35A)	119.9(17)
C(11A)-C(12A)-H(12A)	120.0	F(34A)-C(35A)-C(36A)	120.9(19)
C(13A)-C(12A)-H(12A)	120.0	F(34A)-C(35A)-C(34A)	120.9(18)
C(8A)-C(13A)-C(12A)	117.6(18)	C(36A)-C(35A)-C(34A)	118.1(18)
C(8A)-C(13A)-H(13A)	121.2	C(31A)-C(36A)-F(35A)	119.6(19)
C(12A)-C(13A)-H(13A)	121.2	C(31A)-C(36A)-C(35A)	125.0(19)
C(22A)-C(21A)-C(26A)	115.0(18)	F(35A)-C(36A)-C(35A)	115.3(19)
C(22A)-C(21A)-B(1A)	120.4(18)	N(1A)-B(1A)-N(2A)	106.3(17)
C(26A)-C(21A)-B(1A)	124.6(18)	N(1A)-B(1A)-C(21A)	113.9(18)
C(21A)-C(22A)-F(21A)	119(2)	N(2A)-B(1A)-C(21A)	107.0(19)
C(21A)-C(22A)-C(23A)	124.1(19)	N(1A)-B(1A)-C(31A)	112(2)
F(21A)-C(22A)-C(23A)	117(2)	N(2A)-B(1A)-C(31A)	111.5(19)
F(22A)-C(23A)-C(24A)	122.9(18)	C(21A)-B(1A)-C(31A)	106(2)
F(22A)-C(23A)-C(22A)	118.4(19)		

Table SI 10: Anisotropic displacement parameters (Å2x 103) for β-diketiminate borane complex **9**. The anisotropic displacement factor exponent takes the form: -2p2[h2 a*2U11 + ... + 2 h k a* b* U12]

	U^{11}	U^{22}	U^{33}	U^{23}	U^{13}	U^{12}
F(21)	33(7)	34(12)	22(8)	7(7)	4(7)	4(8)
F(22)	22(6)	38(6)	32(9)	11(7)	-2(7)	9(5)
F(23)	25(6)	32(8)	43(10)	-10(8)	-4(8)	14(5)
F(24)	24(7)	21(8)	21(6)	-2(5)	-5(5)	9(5)
F(25)	36(7)	4(9)	24(6)	3(5)	9(5)	4(7)
F(31)	30(6)	15(8)	14(7)	-1(5)	0(5)	-2(6)
F(32)	28(5)	35(8)	26(8)	-1(6)	-13(7)	-5(5)
F(33)	21(4)	23(4)	25(6)	12(5)	-4(5)	-3(3)
F(34)	22(5)	41(10)	25(8)	21(7)	1(6)	-9(6)
F(35)	30(6)	24(10)	15(6)	-6(5)	-4(5)	-5(6)
N(1)	20(7)	19(4)	12(5)	-1(4)	0(5)	0(4)
N(2)	27(5)	21(5)	8(5)	8(4)	0(5)	-3(5)
C(1)	27(8)	24(8)	17(10)	-1(8)	4(9)	6(7)
C(2)	16(5)	16(4)	15(6)	1(5)	4(6)	0(4)
C(3)	18(6)	19(5)	11(6)	6(5)	8(5)	0(5)
C(4)	15(5)	20(5)	12(5)	3(4)	4(5)	-4(4)
C(5)	18(3)	25(3)	12(2)	2(2)	6(3)	-10(3)
C(6)	19(6)	30(6)	14(5)	-2(5)	2(5)	-2(5)
C(7)	46(9)	27(8)	23(8)	2(7)	6(7)	-1(7)
C(8)	18(3)	25(3)	12(2)	2(2)	6(3)	-10(3)
C(9)	22(4)	24(5)	18(5)	4(4)	5(4)	-14(4)
C(10)	17(5)	27(6)	20(4)	8(5)	5(4)	-13(4)
C(11)	19(7)	25(7)	14(5)	2(6)	9(6)	-3(6)
C(12)	24(7)	23(6)	15(6)	5(5)	5(6)	-8(5)
C(13)	18(3)	25(3)	12(2)	2(2)	6(3)	-10(3)
C(21)	16(4)	17(5)	22(5)	1(4)	1(4)	-3(4)
C(22)	20(5)	23(5)	19(6)	1(5)	1(5)	-2(4)
C(23)	19(5)	19(5)	24(7)	3(6)	-3(6)	-3(4)
C(24)	17(5)	20(6)	24(7)	-2(6)	-4(6)	3(4)
C(25)	19(5)	18(7)	22(6)	-2(5)	-3(5)	2(5)
C(26)	18(5)	16(6)	24(6)	0(5)	1(5)	0(5)
C(31)	18(4)	13(4)	14(5)	2(5)	-4(4)	6(4)
C(32)	19(5)	14(6)	14(6)	3(5)	-6(5)	5(4)
C(33)	20(5)	19(6)	18(6)	3(6)	-9(5)	1(4)

C(34)	21(4)	23(4)	25(6)	12(5)	-4(5)	-3(3)
C(35)	19(5)	23(6)	19(6)	9(6)	-4(5)	0(4)
C(36)	20(5)	18(6)	14(6)	5(6)	-5(5)	1(4)
B(1)	23(4)	17(4)	15(4)	-1(4)	0(4)	1(4)
F(21A)	34(9)	18(10)	22(8)	4(8)	-2(8)	3(7)
F(22A)	27(8)	29(7)	26(10)	17(9)	4(8)	3(6)
F(23A)	24(7)	34(8)	32(11)	3(8)	-2(9)	11(6)
F(24A)	26(8)	15(9)	19(8)	-3(6)	-8(7)	15(6)
F(25A)	35(8)	9(13)	15(7)	-5(7)	1(6)	3(9)
F(31A)	34(8)	23(13)	13(8)	-2(7)	-2(6)	3(9)
F(32A)	30(7)	42(11)	14(9)	-9(8)	-2(7)	3(8)
F(33A)	20(6)	29(6)	25(9)	0(8)	-8(8)	-7(5)
F(34A)	33(8)	46(11)	13(8)	1(7)	-2(7)	-13(8)
F(35A)	27(8)	27(14)	19(8)	2(8)	-5(7)	-15(9)
N(1A)	21(7)	18(5)	13(5)	-1(5)	1(6)	-2(5)
N(2A)	25(6)	21(6)	10(6)	6(5)	0(5)	-1(6)
C(1A)	33(11)	18(9)	12(12)	2(9)	7(12)	-2(9)
C(2A)	17(6)	17(5)	14(6)	1(5)	4(7)	-1(5)
C(3A)	17(7)	18(6)	14(7)	-1(5)	3(6)	3(5)
C(4A)	18(6)	22(6)	12(5)	1(5)	4(5)	2(5)
C(5A)	21(5)	25(6)	13(5)	-1(5)	4(5)	-5(5)
C(6A)	29(7)	27(8)	26(8)	-1(7)	-5(7)	-2(6)
C(7A)	34(9)	34(10)	33(11)	-2(9)	-4(8)	5(8)
C(8A)	18(3)	25(3)	12(2)	2(2)	6(3)	-10(3)
C(9A)	22(4)	24(5)	18(5)	4(4)	5(4)	-14(4)
C(10A)	17(5)	27(6)	20(4)	8(5)	5(4)	-13(4)
C(11A)	20(7)	24(7)	16(6)	7(6)	7(7)	-9(6)
C(12A)	23(7)	20(7)	16(6)	-2(6)	9(6)	-11(6)
C(13A)	18(3)	25(3)	12(2)	2(2)	6(3)	-10(3)
C(21A)	18(5)	18(5)	20(5)	-1(5)	0(5)	-3(5)
C(22A)	17(6)	21(6)	22(7)	1(6)	-2(6)	-3(5)
C(23A)	20(6)	22(6)	20(7)	3(6)	-2(6)	-1(5)
C(24A)	17(5)	20(6)	21(7)	4(6)	-3(6)	-1(5)
C(25A)	20(6)	15(7)	20(7)	-2(6)	-2(6)	0(5)
C(26A)	20(6)	15(7)	22(6)	0(5)	0(5)	-1(5)
C(31A)	18(5)	15(5)	14(6)	4(5)	-5(5)	6(4)
C(32A)	21(5)	19(7)	14(7)	5(6)	-6(5)	5(5)
C(33A)	21(5)	19(6)	12(7)	3(6)	-9(6)	4(5)
C(34A)	18(5)	23(6)	16(7)	6(6)	-9(6)	2(5)

C(35A)	20(5)	25(7)	11(7)	5(6)	-5(6)	1(5)
C(36A)	21(5)	21(6)	17(6)	2(6)	-6(5)	0(5)
B(1A)	23(5)	17(5)	15(5)	0(4)	-1(4)	1(4)

Table SI 11: Hydrogen coordinates (x 104) and isotropic displacement parameters (Å2x 10 3) for β-diketiminate complex **9**.

	x	y	z	U(eq)
H(1)	2740(110)	4130(60)	6300(40)	21
H(2)	1800(200)	4460(70)	4260(50)	22
H(1A)	2657	2056	6198	34
H(1B)	2304	2718	6763	34
H(1C)	3349	2731	6502	34
H(3)	2160	2304	5015	19
H(5)	1091	2656	3986	22
H(6)	473	3868	3553	25
H(7A)	2001	3951	2682	38
H(7B)	1043	4473	2600	38
H(9)	3351	3429	3586	25
H(10)	4457	2644	2989	26
H(11)	4077	1380	2507	23
H(12)	2572	864	2679	25
H(13)	1456	1736	3140	22
H(1AN)	2330(160)	4010(80)	6310(20)	21
H(2A)	1800(300)	4640(60)	4260(80)	22
H(1AA)	2320	2074	6087	31
H(1AB)	2571	2783	6641	31
H(1AC)	3348	2412	6132	31
H(3A)	2388	2415	4760	20
H(5A)	1130	2974	3723	24
H(6A)	2018	4289	2974	33
H(7AA)	202	4183	3386	40
H(7AB)	660	4866	2849	40
H(9A)	3447	3578	3460	25
H(10A)	4594	2789	2962	26
H(11A)	4289	1504	2516	24
H(12A)	2765	1003	2565	23
H(13A)	1688	1694	3311	22

Table SI 12: Hydrogen bonds for β-diketiminate complex **9** [Å and °].

D-H...A	d(D-H)	d(H...A)	d(D...A)	<(DHA)
N(1)-H(1)...F(31)	0.89(3)	2.36(12)	2.86(3)	116(9)
N(2)-H(2)...F(35)	0.88(3)	2.3(3)	2.79(4)	108(20)
N(1A)-H(1AN)...F(25A)	0.88(3)	2.23(13)	2.81(5)	123(12)
N(2A)-H(2A)...F(21A)	0.89(3)	2.0(2)	2.62(5)	122(18)

7.4 Vinylimin borane complex 13'

The crystal structure of vinylimin borane complex **13'** was solved in the monoclinic space group *C*2/*c*. The asymmetric unit contains one molecule. The molecule was found to be disordered in two positions, the disorder was modelled with the help of same distance restraints, restraints to a common plane, similarity restraints on anisotropic displacement parameters and advanced rigid bond restraints. Two disordered atoms in close proximity were set to the same anisotropic displacement parameters. The disorder ratio was refined freely and converged to 0.52(3) and 0.45(3).

Table SI 13: Crystal data and structure refinement for vinylimin borane complex **13'**.

CCDC No	2022613	
Empirical formula	$C_{29} H_{13} B F_{15} N$	
Formula weight	671.21	
Temperature	100(2) K	
Wavelength	0.71073 Å	
Crystal system	Monoclinic	
Space group	*C*2/*c*	
Unit cell dimensions	a = 26.1703(9) Å	α= 90°.
	b = 14.6744(5) Å	β= 127.017(2)°.
	c = 17.9119(6) Å	γ = 90°.
Volume	5492.4(3) $Å^3$	
Z	8	
Density (calculated)	1.623 Mg/m^3	
Absorption coefficient	0.166 mm^{-1}	
F(000)	2672	
Crystal size	0.783 x 0.377 x 0.373 mm^3	
Theta range for data collection	1.949 to 29.129°.	
Index ranges	-35 ≤ h ≤ 35, -20 ≤ k ≤ 20, -24 ≤ l ≤ 24	
Reflections collected	159930	
Independent reflections	7396 [R(int) = 0.0579]	
Completeness to theta = 25.242°	100.0 %	
Absorption correction	Semi-empirical from equivalents	
Refinement method	Full-matrix least-squares on F^2	
Data / restraints / parameters	7396 / 862 / 569	
Goodness-of-fit on F^2	1.019	
Final R indices [I>2σ(I)]	R1 = 0.0391, wR2 = 0.1003	
R indices (all data)	R1 = 0.0502, wR2 = 0.1079	
Largest diff. peak and hole	0.409 and -0.401 e.$Å^{-3}$	

Table SI 14: Atomic coordinates (x 104) and equivalent isotropic displacement parameters (Å2x 103) for vinylimin borane complex **13'**. U(eq) is defined as one third of the trace of the orthogonalized Uij tensor.

	x	y	z	U(eq)
N(1)	2607(1)	5702(1)	3193(1)	22(1)
C(1)	2296(1)	6252(1)	2487(1)	21(1)
C(2)	2636(1)	6981(1)	2366(1)	25(1)
C(3)	1598(1)	6132(1)	1806(1)	23(1)
C(4)	1242(1)	6730(1)	1109(1)	31(1)
C(5)	532(1)	6664(1)	386(1)	41(1)
C(6)	1328(12)	5288(8)	1903(16)	25(2)
C(7)	1283(4)	4506(5)	1433(6)	24(1)
C(8)	1017(4)	3730(5)	1515(7)	35(1)
C(9)	812(4)	3720(7)	2065(7)	37(2)
C(10)	864(5)	4488(9)	2537(7)	41(2)
C(11)	1099(7)	5286(8)	2424(10)	39(2)
C(6A)	1298(14)	5355(8)	1969(18)	22(2)
C(7A)	1253(6)	4493(7)	1617(9)	39(2)
C(8A)	1002(5)	3756(5)	1781(11)	43(2)
C(9A)	782(4)	3878(6)	2300(10)	41(2)
C(10A)	823(5)	4721(7)	2662(9)	40(2)
C(11A)	1107(7)	5453(7)	2537(10)	28(2)
F(21)	4216(1)	3900(1)	4871(1)	27(1)
F(22)	4182(1)	2646(1)	5915(1)	35(1)
F(23)	3337(1)	2782(1)	6317(1)	47(1)
F(24)	2533(1)	4244(1)	5684(1)	49(1)
F(25)	2574(1)	5530(1)	4669(1)	37(1)
C(21)	3376(1)	4768(1)	4681(1)	23(1)
C(22)	3785(1)	4021(1)	5042(1)	23(1)
C(23)	3780(1)	3356(1)	5588(1)	27(1)
C(24)	3351(1)	3423(1)	5793(1)	33(1)
C(25)	2944(1)	4157(1)	5471(1)	34(1)
C(26)	2970(1)	4809(1)	4938(1)	28(1)
F(31)	4710(6)	5790(9)	5650(9)	22(1)
F(32)	5261(4)	7183(7)	6708(7)	57(3)
F(33)	4599(4)	8739(6)	6530(5)	34(1)
F(34)	3277(5)	8705(8)	5156(7)	79(3)
F(35)	2718(6)	7300(13)	4093(10)	34(2)

C(31)	3687(6)	6456(8)	4775(9)	18(2)
C(32)	4337(6)	6511(9)	5502(9)	21(2)
C(33)	4632(5)	7256(8)	6075(7)	26(2)
C(34)	4303(4)	8030(6)	5984(6)	21(2)
C(35)	3654(5)	8006(8)	5291(9)	34(1)
C(36)	3372(6)	7237(8)	4726(9)	31(2)
F(31A)	4613(5)	5737(7)	5513(8)	24(1)
F(32A)	5245(3)	7157(4)	6779(4)	27(1)
F(33A)	4556(4)	8642(7)	6533(5)	66(2)
F(34A)	3314(3)	8783(4)	5147(4)	36(1)
F(35A)	2676(5)	7399(9)	3952(8)	29(1)
C(31A)	3626(4)	6524(6)	4651(8)	18(2)
C(32A)	4271(5)	6485(7)	5398(8)	22(2)
C(33A)	4605(4)	7162(7)	6049(6)	30(2)
C(34A)	4260(4)	7929(7)	5930(7)	44(2)
C(35A)	3621(4)	8016(6)	5228(7)	34(1)
C(36A)	3306(4)	7293(5)	4612(7)	21(1)
F(41)	3012(1)	4004(1)	2903(1)	30(1)
F(42)	3428(1)	3492(1)	1921(1)	39(1)
F(43)	4296(1)	4533(1)	1936(1)	37(1)
F(44)	4736(1)	6098(1)	2962(1)	32(1)
F(45)	4343(1)	6613(1)	3945(1)	24(1)
C(41)	3670(1)	5317(1)	3506(1)	20(1)
C(42)	3454(1)	4538(1)	2951(1)	23(1)
C(43)	3655(1)	4257(1)	2431(1)	27(1)
C(44)	4094(1)	4783(1)	2437(1)	26(1)
C(45)	4320(1)	5568(1)	2962(1)	23(1)
C(46)	4107(1)	5816(1)	3477(1)	20(1)
B(1)	3349(1)	5590(1)	4033(1)	21(1)

Table SI 15: Bond lengths [Å] and angles [°] for vinylimin borane complex **13'.**

N(1)-C(1)	1.2927(16)	C(11A)-H(11A)	0.9500
N(1)-B(1)	1.5955(17)	F(21)-C(22)	1.3472(14)
N(1)-H(1)	0.873(13)	F(22)-C(23)	1.3398(15)
C(1)-C(3)	1.4744(17)	F(23)-C(24)	1.3438(16)
C(1)-C(2)	1.4875(18)	F(24)-C(25)	1.3448(16)
C(2)-H(2A)	0.9800	F(25)-C(26)	1.3508(15)
C(2)-H(2B)	0.9800	C(21)-C(22)	1.3897(17)
C(2)-H(2C)	0.9800	C(21)-C(26)	1.3919(18)
C(3)-C(4)	1.3408(18)	C(21)-B(1)	1.6454(19)
C(3)-C(6)	1.487(8)	C(22)-C(23)	1.3869(18)
C(3)-C(6A)	1.510(9)	C(23)-C(24)	1.379(2)
C(4)-C(5)	1.497(2)	C(24)-C(25)	1.374(2)
C(4)-H(4)	0.9500	C(25)-C(26)	1.3821(19)
C(5)-H(5A)	0.9800	F(31)-C(32)	1.355(10)
C(5)-H(5B)	0.9800	F(32)-C(33)	1.327(10)
C(5)-H(5C)	0.9800	F(33)-C(34)	1.315(9)
C(6)-C(11)	1.381(9)	F(34)-C(35)	1.340(10)
C(6)-C(7)	1.385(9)	F(35)-C(36)	1.373(10)
C(7)-C(8)	1.387(7)	C(31)-C(36)	1.383(10)
C(7)-H(7)	0.9500	C(31)-C(32)	1.389(9)
C(8)-C(9)	1.379(7)	C(31)-B(1)	1.657(9)
C(8)-H(8)	0.9500	C(32)-C(33)	1.375(9)
C(9)-C(10)	1.366(7)	C(33)-C(34)	1.374(9)
C(9)-H(9)	0.9500	C(34)-C(35)	1.374(9)
C(10)-C(11)	1.393(9)	C(35)-C(36)	1.394(10)
C(10)-H(10)	0.9500	F(31A)-C(32A)	1.352(8)
C(11)-H(11)	0.9500	F(32A)-C(33A)	1.370(7)
C(6A)-C(7A)	1.386(9)	F(33A)-C(34A)	1.361(8)
C(6A)-C(11A)	1.386(9)	F(34A)-C(35A)	1.339(8)
C(7A)-C(8A)	1.386(9)	F(35A)-C(36A)	1.338(8)
C(7A)-H(7A)	0.9500	C(31A)-C(36A)	1.384(7)
C(8A)-C(9A)	1.367(8)	C(31A)-C(32A)	1.386(8)
C(8A)-H(8A)	0.9500	C(31A)-B(1)	1.632(8)
C(9A)-C(10A)	1.371(7)	C(32A)-C(33A)	1.371(8)
C(9A)-H(9A)	0.9500	C(33A)-C(34A)	1.376(8)
C(10A)-C(11A)	1.400(9)	C(34A)-C(35A)	1.365(8)
C(10A)-H(10A)	0.9500	C(35A)-C(36A)	1.388(8)

F(41)-C(42)	1.3561(14)
F(42)-C(43)	1.3395(15)
F(43)-C(44)	1.3375(15)
F(44)-C(45)	1.3405(15)
F(45)-C(46)	1.3520(14)
C(41)-C(46)	1.3851(17)
C(41)-C(42)	1.3916(17)
C(41)-B(1)	1.6462(18)
C(42)-C(43)	1.3804(19)
C(43)-C(44)	1.380(2)
C(44)-C(45)	1.3759(19)
C(45)-C(46)	1.3829(17)
C(1)-N(1)-B(1)	132.64(11)
C(1)-N(1)-H(1)	111.9(11)
B(1)-N(1)-H(1)	115.3(11)
N(1)-C(1)-C(3)	118.07(11)
N(1)-C(1)-C(2)	120.87(11)
C(3)-C(1)-C(2)	121.06(11)
C(1)-C(2)-H(2A)	109.5
C(1)-C(2)-H(2B)	109.5
H(2A)-C(2)-H(2B)	109.5
C(1)-C(2)-H(2C)	109.5
H(2A)-C(2)-H(2C)	109.5
H(2B)-C(2)-H(2C)	109.5
C(4)-C(3)-C(1)	120.90(12)
C(4)-C(3)-C(6)	122.7(11)
C(1)-C(3)-C(6)	116.2(11)
C(4)-C(3)-C(6A)	121.6(12)
C(1)-C(3)-C(6A)	117.4(12)
C(3)-C(4)-C(5)	124.67(14)
C(3)-C(4)-H(4)	117.7
C(5)-C(4)-H(4)	117.7
C(4)-C(5)-H(5A)	109.5
C(4)-C(5)-H(5B)	109.5
H(5A)-C(5)-H(5B)	109.5
C(4)-C(5)-H(5C)	109.5
H(5A)-C(5)-H(5C)	109.5
H(5B)-C(5)-H(5C)	109.5
C(11)-C(6)-C(7)	119.7(7)
C(11)-C(6)-C(3)	120.7(8)
C(7)-C(6)-C(3)	119.5(8)
C(6)-C(7)-C(8)	118.7(6)
C(6)-C(7)-H(7)	120.6
C(8)-C(7)-H(7)	120.6
C(9)-C(8)-C(7)	121.4(6)
C(9)-C(8)-H(8)	119.3
C(7)-C(8)-H(8)	119.3
C(10)-C(9)-C(8)	119.8(6)
C(10)-C(9)-H(9)	120.1
C(8)-C(9)-H(9)	120.1
C(9)-C(10)-C(11)	119.4(6)
C(9)-C(10)-H(10)	120.3
C(11)-C(10)-H(10)	120.3
C(6)-C(11)-C(10)	120.7(7)
C(6)-C(11)-H(11)	119.7
C(10)-C(11)-H(11)	119.7
C(7A)-C(6A)-C(11A)	117.4(7)
C(7A)-C(6A)-C(3)	120.9(9)
C(11A)-C(6A)-C(3)	121.5(8)
C(6A)-C(7A)-C(8A)	122.5(7)
C(6A)-C(7A)-H(7A)	118.8
C(8A)-C(7A)-H(7A)	118.8
C(9A)-C(8A)-C(7A)	119.2(7)
C(9A)-C(8A)-H(8A)	120.4
C(7A)-C(8A)-H(8A)	120.4
C(8A)-C(9A)-C(10A)	119.8(7)
C(8A)-C(9A)-H(9A)	120.1
C(10A)-C(9A)-H(9A)	120.1
C(9A)-C(10A)-C(11A)	120.8(7)
C(9A)-C(10A)-H(10A)	119.6
C(11A)-C(10A)-H(10A)	119.6
C(6A)-C(11A)-C(10A)	120.0(7)
C(6A)-C(11A)-H(11A)	120.0
C(10A)-C(11A)-H(11A)	120.0
C(22)-C(21)-C(26)	113.99(11)
C(22)-C(21)-B(1)	127.02(11)
C(26)-C(21)-B(1)	118.96(11)

F(21)-C(22)-C(23)	115.70(11)
F(21)-C(22)-C(21)	120.81(11)
C(23)-C(22)-C(21)	123.49(12)
F(22)-C(23)-C(24)	119.40(12)
F(22)-C(23)-C(22)	121.05(12)
C(24)-C(23)-C(22)	119.55(12)
F(23)-C(24)-C(25)	120.18(13)
F(23)-C(24)-C(23)	120.24(13)
C(25)-C(24)-C(23)	119.57(12)
F(24)-C(25)-C(24)	120.35(13)
F(24)-C(25)-C(26)	120.69(13)
C(24)-C(25)-C(26)	118.96(13)
F(25)-C(26)-C(25)	116.22(12)
F(25)-C(26)-C(21)	119.39(12)
C(25)-C(26)-C(21)	124.39(13)
C(36)-C(31)-C(32)	110.9(9)
C(36)-C(31)-B(1)	125.1(8)
C(32)-C(31)-B(1)	123.9(8)
F(31)-C(32)-C(33)	117.4(9)
F(31)-C(32)-C(31)	118.0(9)
C(33)-C(32)-C(31)	124.5(9)
F(32)-C(33)-C(34)	122.3(9)
F(32)-C(33)-C(32)	115.1(9)
C(34)-C(33)-C(32)	122.6(9)
F(33)-C(34)-C(33)	121.2(7)
F(33)-C(34)-C(35)	123.2(7)
C(33)-C(34)-C(35)	115.5(8)
F(34)-C(35)-C(34)	121.2(9)
F(34)-C(35)-C(36)	118.6(9)
C(34)-C(35)-C(36)	120.2(9)
F(35)-C(36)-C(31)	120.4(11)
F(35)-C(36)-C(35)	113.4(10)
C(31)-C(36)-C(35)	126.2(9)
C(36A)-C(31A)-C(32A)	115.5(7)
C(36A)-C(31A)-B(1)	130.0(6)
C(32A)-C(31A)-B(1)	114.0(7)
F(31A)-C(32A)-C(33A)	115.8(8)
F(31A)-C(32A)-C(31A)	119.2(7)
C(33A)-C(32A)-C(31A)	124.9(7)
F(32A)-C(33A)-C(32A)	126.4(7)
F(32A)-C(33A)-C(34A)	117.5(7)
C(32A)-C(33A)-C(34A)	116.0(7)
F(33A)-C(34A)-C(35A)	117.0(8)
F(33A)-C(34A)-C(33A)	120.0(8)
C(35A)-C(34A)-C(33A)	123.0(8)
F(34A)-C(35A)-C(34A)	120.1(7)
F(34A)-C(35A)-C(36A)	121.7(7)
C(34A)-C(35A)-C(36A)	118.2(7)
F(35A)-C(36A)-C(31A)	121.7(8)
F(35A)-C(36A)-C(35A)	116.1(8)
C(31A)-C(36A)-C(35A)	122.1(7)
C(46)-C(41)-C(42)	113.44(11)
C(46)-C(41)-B(1)	127.53(11)
C(42)-C(41)-B(1)	118.79(11)
F(41)-C(42)-C(43)	116.21(11)
F(41)-C(42)-C(41)	118.97(11)
C(43)-C(42)-C(41)	124.83(12)
F(42)-C(43)-C(44)	119.97(12)
F(42)-C(43)-C(42)	121.15(12)
C(44)-C(43)-C(42)	118.88(12)
F(43)-C(44)-C(45)	120.58(12)
F(43)-C(44)-C(43)	120.40(12)
C(45)-C(44)-C(43)	119.02(12)
F(44)-C(45)-C(44)	119.94(11)
F(44)-C(45)-C(46)	120.15(11)
C(44)-C(45)-C(46)	119.90(12)
F(45)-C(46)-C(45)	115.07(11)
F(45)-C(46)-C(41)	120.98(11)
C(45)-C(46)-C(41)	123.93(11)
N(1)-B(1)-C(31A)	110.2(3)
N(1)-B(1)-C(21)	104.59(10)
C(31A)-B(1)-C(21)	108.9(5)
N(1)-B(1)-C(41)	103.67(10)
C(31A)-B(1)-C(41)	115.0(5)
C(21)-B(1)-C(41)	113.87(10)
N(1)-B(1)-C(31)	115.8(4)
C(21)-B(1)-C(31)	102.8(6)
C(41)-B(1)-C(31)	115.9(5)

Table SI 16: Anisotropic displacement parameters (Å2x 103) for vinylimin borane complex **13'**. The anisotropic displacement factor exponent takes the form: -2p2[h2 a*2U11 + ... + 2 h k a* b* U12]

	U^{11}	U^{22}	U^{33}	U^{23}	U^{13}	U^{12}
N(1)	18(1)	24(1)	22(1)	3(1)	11(1)	1(1)
C(1)	20(1)	20(1)	21(1)	-1(1)	12(1)	3(1)
C(2)	24(1)	23(1)	28(1)	4(1)	16(1)	3(1)
C(3)	21(1)	25(1)	21(1)	0(1)	11(1)	2(1)
C(4)	26(1)	34(1)	26(1)	6(1)	12(1)	2(1)
C(5)	25(1)	49(1)	32(1)	15(1)	8(1)	3(1)
C(6)	14(2)	28(3)	25(4)	3(2)	8(2)	4(3)
C(7)	21(2)	19(2)	29(3)	-4(1)	14(2)	-2(1)
C(8)	26(2)	25(2)	38(3)	1(2)	11(2)	-2(1)
C(9)	24(2)	32(3)	40(3)	8(2)	11(2)	-5(2)
C(10)	41(2)	43(4)	46(3)	6(3)	30(2)	-4(3)
C(11)	42(2)	34(4)	38(3)	-10(3)	23(2)	-9(3)
C(6A)	18(4)	18(2)	23(3)	-1(2)	8(2)	2(2)
C(7A)	35(2)	44(3)	41(4)	-9(2)	26(3)	4(2)
C(8A)	34(2)	23(2)	55(5)	-8(3)	18(3)	-3(2)
C(9A)	30(2)	22(3)	56(5)	6(3)	18(3)	1(2)
C(10A)	45(3)	24(3)	59(4)	2(2)	35(2)	-1(2)
C(11A)	38(3)	19(3)	36(3)	-4(2)	27(3)	-8(2)
F(21)	25(1)	27(1)	30(1)	6(1)	18(1)	7(1)
F(22)	41(1)	27(1)	36(1)	11(1)	23(1)	10(1)
F(23)	58(1)	45(1)	52(1)	24(1)	41(1)	10(1)
F(24)	48(1)	63(1)	58(1)	25(1)	44(1)	16(1)
F(25)	34(1)	46(1)	41(1)	18(1)	29(1)	19(1)
C(21)	20(1)	25(1)	21(1)	4(1)	12(1)	3(1)
C(22)	22(1)	25(1)	23(1)	2(1)	13(1)	1(1)
C(23)	29(1)	24(1)	27(1)	5(1)	15(1)	4(1)
C(24)	36(1)	34(1)	31(1)	11(1)	22(1)	2(1)
C(25)	31(1)	44(1)	34(1)	11(1)	24(1)	5(1)
C(26)	24(1)	34(1)	28(1)	9(1)	16(1)	7(1)
F(31)	9(3)	22(2)	16(3)	-3(2)	-2(2)	0(2)
F(32)	35(3)	63(4)	40(4)	-6(3)	5(2)	-3(3)
F(33)	31(2)	24(2)	29(2)	-12(2)	10(2)	-4(1)
F(34)	70(5)	70(5)	73(5)	-29(3)	30(4)	30(3)
F(35)	25(2)	43(4)	23(3)	3(2)	9(2)	18(2)

C(31)	22(3)	19(2)	13(3)	5(2)	10(2)	7(2)
C(32)	12(2)	26(3)	15(3)	5(2)	3(2)	4(2)
C(33)	26(3)	24(3)	20(3)	-11(2)	11(2)	-9(2)
C(34)	21(2)	21(3)	19(3)	-7(2)	10(2)	0(2)
C(35)	37(1)	32(1)	30(1)	-4(1)	19(1)	11(1)
C(36)	20(3)	46(4)	17(3)	6(2)	5(2)	7(2)
F(31A)	12(3)	25(2)	16(2)	-1(2)	-2(2)	2(2)
F(32A)	21(2)	25(2)	19(2)	-5(1)	3(1)	1(1)
F(33A)	69(4)	47(3)	56(3)	-32(2)	23(2)	-4(2)
F(34A)	35(2)	28(2)	37(2)	-8(1)	18(2)	10(1)
F(35A)	22(2)	35(2)	20(2)	1(2)	7(2)	15(1)
C(31A)	15(2)	24(2)	10(2)	7(2)	4(2)	6(2)
C(32A)	27(3)	20(2)	17(2)	-2(2)	11(2)	2(2)
C(33A)	24(3)	30(3)	22(3)	5(2)	7(2)	6(2)
C(34A)	54(3)	32(3)	30(3)	-17(2)	17(2)	-7(2)
C(35A)	37(1)	32(1)	30(1)	-4(1)	19(1)	11(1)
C(36A)	19(2)	23(2)	15(2)	2(2)	8(2)	13(1)
F(41)	28(1)	27(1)	35(1)	-6(1)	19(1)	-9(1)
F(42)	37(1)	36(1)	37(1)	-19(1)	19(1)	-7(1)
F(43)	36(1)	52(1)	31(1)	-11(1)	24(1)	1(1)
F(44)	30(1)	39(1)	34(1)	-1(1)	24(1)	-5(1)
F(45)	27(1)	19(1)	30(1)	-3(1)	18(1)	-2(1)
C(41)	18(1)	19(1)	19(1)	2(1)	10(1)	3(1)
C(42)	20(1)	22(1)	23(1)	0(1)	11(1)	-2(1)
C(43)	25(1)	26(1)	23(1)	-6(1)	11(1)	0(1)
C(44)	24(1)	34(1)	21(1)	-2(1)	13(1)	4(1)
C(45)	19(1)	28(1)	21(1)	2(1)	12(1)	2(1)
C(46)	19(1)	18(1)	18(1)	2(1)	9(1)	3(1)
B(1)	17(1)	22(1)	21(1)	3(1)	11(1)	4(1)

Table SI 17: Hydrogen coordinates (x 104) and isotropic displacement parameters (Å2x 10 3) for vinylimin borane complex **13'**.

	x	y	z	U(eq)
H(1)	2355(7)	5296(10)	3174(11)	26
H(2A)	2572	6890	1773	38
H(2B)	3094	6957	2884	38
H(2C)	2464	7577	2360	38
H(4)	1455	7231	1070	37
H(5A)	436	6463	-209	62
H(5B)	338	7263	302	62
H(5C)	358	6224	590	62
H(7)	1431	4502	1062	29
H(8)	976	3195	1185	42
H(9)	635	3180	2116	45
H(10)	740	4478	2939	49
H(11)	1102	5834	2709	47
H(7A)	1401	4404	1250	46
H(8A)	982	3172	1535	52
H(9A)	603	3381	2409	49
H(10A)	655	4809	3003	48
H(11A)	1169	6017	2842	34

8 Computational details

All structures were fully optimized with the composite method PBEh-3c.[16] Solvent effects were considered implicitly using the SMD (Solvation Model Based on Density) model for benzene.[17] Thermodynamic properties were obtained at the same level of theory from a frequency computation. The computed free energies were corrected regarding the standard state by adding RT ln(c0s/c0g) (i.e., about 1.89 kcal mol^{-1} for a concentration of 1 M in solution [c0s] and 1 bar in the gas phase [c0g] at 298.15 K) to energies of all structures.[17] For final single point energy computations, the revDSD-PBEP86 functional with Grimmes D4 dispersion correction and the def2-QZVPP basis set was used in combination with the RIJK approximation.[18]

All computations were performed with the ORCA program package, Version 4.2.1.[19]

3

C	-0.313552372	-3.585068978	2.708395561
C	0.538437628	-3.240687978	3.743782561
C	1.280495628	-2.078628978	3.642185561
C	1.194729628	-1.239737978	2.545922561
C	0.333349628	-1.616447978	1.530948561
C	-0.419520372	-2.772631978	1.591866561
F	-1.233406372	-3.112771978	0.601607561
F	0.231628628	-0.852679978	0.445100561
B	2.061458628	0.066660022	2.460837561
O	1.551993628	1.269314022	2.125930561
C	0.234382628	1.601111022	2.115720561
N	-0.512108372	1.128016022	3.077379561
C	-1.808003372	1.442298022	3.120445561
C	-2.367076372	2.277256022	2.162413561
C	-1.555690372	2.784014022	1.154463561
C	-0.218007372	2.449991022	1.114769561
H	0.448224628	2.815840022	0.346890561
H	-1.972523372	3.438353022	0.400308561
H	-3.414193372	2.537777022	2.192276561
C	-2.587669372	0.827243022	4.270212561
C	-4.058780372	1.236094022	4.254786561
H	-4.186762372	2.315459022	4.354860561
H	-4.566211372	0.914064022	3.343616561
H	-4.578173372	0.771386022	5.093558561
C	-2.491647372	-0.699219978	4.157907561
H	-2.928599372	-1.058822978	3.224400561
H	-3.027712372	-1.174467978	4.981334561
H	-1.454343372	-1.028872978	4.198264561
C	-1.954722372	1.280988022	5.590749561
H	-0.907166372	0.988693022	5.648162561
H	-2.480228372	0.830390022	6.434687561
H	-2.009281372	2.364895022	5.705395561
C	3.609674628	0.021829022	2.697936561
C	4.266999628	1.003482022	3.427046561
C	5.630794628	0.965075022	3.651350561

C	6.373666628	-0.076826978	3.122977561
C	5.754485628	-1.071953978	2.386306561
C	4.387595628	-1.009897978	2.193594561
F	3.819022628	-1.969366978	1.468313561
F	6.473256628	-2.061682978	1.875047561
F	7.679312628	-0.121361978	3.322496561
F	6.231735628	1.907591022	4.364196561
F	3.580655628	2.001784022	3.967080561
F	2.085243628	-1.753049978	4.651571561
F	0.630235628	-4.020554978	4.812115561
F	-1.029216372	-4.694867978	2.785182561

	Hatree	kcal mol^{-1}
E PBEh-3c	-1956.742349	-1227874.413
G	0.25391806	159.3359949
E SMD PBEh-3c	-1956.753408	-1227881.353
E revDSD-PBEP86-D4/def2-QZVPP	-1958.672422	-1229085.552

$TS_{3/4}$

B	1.418405499	0.162229625	0.217453149
C	1.030403499	-1.385359375	0.359725149
C	0.576872499	-2.163386375	-0.692151851
C	0.236374499	-3.497471375	-0.541082851
C	0.352873499	-4.092191375	0.700542149
C	0.809632499	-3.352089375	1.777448149
C	1.139634499	-2.025129375	1.585419149
F	1.576754499	-1.339738375	2.643586149
F	0.924730499	-3.920654375	2.970818149
F	0.030853499	-5.366362375	0.860401149
F	-0.197243501	-4.207458375	-1.575371851
F	0.455712499	-1.648376375	-1.914336851
C	2.869734499	0.654156625	0.652296149
C	3.097785499	1.972652625	1.018065149
C	4.346595499	2.447378625	1.371006149
C	5.426465499	1.582276625	1.348303149
C	5.245572499	0.263641625	0.974822149
C	3.978312499	-0.176887375	0.634234149
F	3.860579499	-1.447471375	0.255179149
F	6.284070499	-0.561564375	0.939624149
F	6.631428499	2.018634625	1.679287149
F	4.520588499	3.714003625	1.726857149
F	2.081132499	2.833088625	1.052527149
O	0.952316499	0.859812625	-0.928992851
C	-0.349373501	1.009214625	-1.145542851
N	-1.152089501	0.758326625	-0.131596851
C	-2.480558501	0.890737625	-0.230231851
C	-3.030770501	1.305374625	-1.427579851
C	-2.185120501	1.588371625	-2.501856851
C	-0.822929501	1.447021625	-2.380664851
H	-0.139495501	1.652690625	-3.191471851
H	-2.608706501	1.917903625	-3.441506851
H	-4.098593501	1.408161625	-1.543207851

C	-3.275290501	0.542947625	1.014551149
C	-4.778390501	0.684166625	0.784716149
H	-5.315266501	0.420975625	1.696444149
H	-5.061358501	1.706472625	0.528100149
H	-5.137067501	0.020794625	-0.004018851
C	-2.964798501	-0.907992375	1.405792149
H	-3.517733501	-1.180282375	2.306196149
H	-1.904390501	-1.055870375	1.610879149
H	-3.251316501	-1.603148375	0.615113149
C	-2.858511501	1.483661625	2.152936149
H	-1.803028501	1.382584625	2.405961149
H	-3.042596501	2.527647625	1.895561149
H	-3.431310501	1.256445625	3.053411149
H	-0.109284501	0.708064625	1.023055149
H	0.658845499	0.711994625	1.433233149

	Hatree	kcal mol^{-1}
E PBEh-3c	-1957.882575	-1228589.915
G	0.27022651	169.5697022
E SMD PBEh-3c	-1957.894933	-1228597.67
E revDSD-PBEP86-D4/def2-QZVPP	-1959.825438	-1229809.08

4

B	-6.130756205	4.302685420	2.624464156
C	-7.735008205	4.408232420	2.586246156
C	-8.495426205	3.897530420	1.547550156
C	-9.876528205	3.998344420	1.509931156
C	-10.538828205	4.627142420	2.547287156
C	-9.818594205	5.145653420	3.608170156
C	-8.440948205	5.026193420	3.605610156
F	-7.783716205	5.532887420	4.650569156
F	-10.454482205	5.745711420	4.610058156
F	-11.861587205	4.729284420	2.528325156
F	-10.570596205	3.494864420	0.493438156
F	-7.912409205	3.271266420	0.525887156
C	-5.409060205	5.747638420	2.880067156
C	-5.431566205	6.664027420	1.837310156
C	-4.846680205	7.915642420	1.906059156
C	-4.196936205	8.291743420	3.068066156
C	-4.150070205	7.415459420	4.134881156
C	-4.759048205	6.175619420	4.026383156
F	-4.668154205	5.379739420	5.098184156
F	-3.522703205	7.768363420	5.252855156
F	-3.621867205	9.483866420	3.156183156
F	-4.896883205	8.752804420	0.875929156
F	-6.043272205	6.340756420	0.697474156
O	-5.786142205	3.301236420	3.732476156
C	-4.608990205	2.859475420	3.902296156
N	-3.611255205	3.218832420	3.064980156
C	-2.308641205	2.848151420	3.170941156
C	-1.974771205	2.012018420	4.195714156
C	-2.977962205	1.577595420	5.083264156

C	-4.273563205	1.983609420	4.953854156
H	-5.051623205	1.673455420	5.635176156
H	-2.709299205	0.912281420	5.893099156
H	-0.955315205	1.689289420	4.330968156
C	-1.369868205	3.427626420	2.132976156
C	-1.413765205	4.962181420	2.215561156
H	-0.705574205	5.388024420	1.504425156
H	-2.393373205	5.376806420	1.969362156
H	-1.144730205	5.317694420	3.210621156
C	0.062595795	2.959813420	2.378815156
H	0.719404795	3.388468420	1.622256156
H	0.157366795	1.875102420	2.311467156
H	0.437180795	3.280889420	3.351629156
C	-1.809269205	2.966538420	0.734705156
H	-1.804834205	1.879142420	0.654110156
H	-2.805953205	3.317491420	0.460422156
H	-1.121433205	3.360318420	-0.013930844
H	-3.888868205	3.811432420	2.288785156
H	-5.697814205	3.851887420	1.576115156

	Hatree	kcal mol^{-1}
E PBEh-3c	-1957.922269	-1228614.824
G	0.27703568	173.842521
E SMD PBEh-3c	-1957.939439	-1228625.598
E revDSD-PBEP86-D4/def2-QZVPP	-1959.861336	-1229831.607

5

C	-2.705975000	-2.875273000	2.429203000
C	-1.485239000	-2.291739000	2.382829000
C	-3.632752000	-2.767794000	1.358996000
C	-1.074699000	-1.527800000	1.227868000
C	-3.281083000	-2.056731000	0.255290000
H	-4.596556000	-3.244817000	1.427756000
H	-2.990688000	-3.442899000	3.306592000
H	-0.781691000	-2.374981000	3.198558000
N	-2.049880000	-1.479409000	0.231039000
O	-0.011983000	-0.960672000	1.069861000
H	-1.772150000	-0.947261000	-0.580685000
C	-4.149794000	-1.846557000	-0.971300000
C	-5.503454000	-2.536072000	-0.812344000
H	-6.063099000	-2.143116000	0.037202000
H	-6.105630000	-2.367828000	-1.705692000
H	-5.400073000	-3.614453000	-0.686954000
C	-3.447328000	-2.434648000	-2.203875000
H	-2.495846000	-1.948110000	-2.424615000
H	-3.251015000	-3.499497000	-2.075090000
H	-4.078168000	-2.313638000	-3.085695000
C	-4.392803000	-0.344145000	-1.177029000
H	-4.880688000	0.098336000	-0.307968000
H	-3.474042000	0.215249000	-1.360640000
H	-5.039061000	-0.185938000	-2.041583000

	Hatree	kcal mol^{-1}
E PBEh-3c	-479.7423749	-301042.8978
G	0.17810151	111.7603895
E SMD PBEh-3c	-479.7577074	-301052.5191
E revDSD-PBEP86-D4/def2-QZVPP	-480.1510918	-301299.3715

6

B	0.686072000	0.009062000	-0.668148000
C	2.188736000	-0.309035000	-0.451348000
C	3.190417000	0.657415000	-0.489098000
C	2.606134000	-1.628402000	-0.293666000
C	4.529371000	0.342878000	-0.370219000
C	3.935420000	-1.976532000	-0.151413000
C	4.897394000	-0.981417000	-0.194554000
C	-0.003746000	1.333871000	-0.247410000
C	-1.081838000	1.819555000	-0.982293000
C	0.343263000	2.052282000	0.893205000
C	-1.763877000	2.968859000	-0.632202000
C	-0.329749000	3.192410000	1.284414000
C	-1.383314000	3.652146000	0.510359000
F	1.706506000	-2.601245000	-0.247124000
F	2.876458000	1.931707000	-0.686584000
F	5.461129000	1.283212000	-0.426374000
F	6.172769000	-1.295787000	-0.069621000
F	4.298938000	-3.239111000	0.019218000
F	1.329092000	1.623662000	1.672015000
F	0.013406000	3.847156000	2.384150000
F	-2.028546000	4.747064000	0.865837000
F	-2.770424000	3.418909000	-1.366882000
F	-1.465419000	1.185477000	-2.082129000
H	0.017781000	-0.817670000	-1.197033000

	Hatree	kcal mol^{-1}
E PBEh-3c	-1478.122986	-927536.2158
G	0.07221061	45.31284378
E SMD PBEh-3c	-1478.125977	-927538.0926
E revDSD-PBEP86-D4/def2-QZVPP	-1479.656391	-928498.4418

MeCN

N	-0.431709000	1.906684000	0.500314000
C	-0.868862000	2.745813000	1.152540000
C	-1.420498000	3.805815000	1.976266000
H	-1.249461000	3.593880000	3.030068000
H	-0.951238000	4.757188000	1.732827000
H	-2.492438000	3.894961000	1.810469000

	Hatree	kcal mol^{-1}
E PBEh-3c	-132.4540093	-83116.14913
G	0.02288454	14.36026625
E SMD PBEh-3c	-132.4598187	-83119.79461
E revDSD-PBEP86-D4/def2-QZVPP	-132.5819154	-83196.41142

1,2-butadien

C	-0.999226000	4.035286000	-1.044577000
C	-0.803414000	2.886987000	-1.626836000
C	-0.611423000	1.737325000	-2.205656000
H	-1.050892000	1.509710000	-3.169054000
H	-0.300064000	4.839493000	-1.255654000
H	-0.002900000	0.968263000	-1.745907000
C	-2.125436000	4.332055000	-0.099913000
H	-2.746590000	5.145359000	-0.478383000
H	-2.760423000	3.461622000	0.049756000
H	-1.743276000	4.645949000	0.872790000

	Hatree	kcal mol^{-1}
E PBEh-3c	-155.6184211	-97652.03764
G	0.05970552	37.465781
E SMD PBEh-3c	-155.6238618	-97655.45168
E revDSD-PBEP86-D4/def2-QZVPP	-155.7430553	-97730.24678

$B(C_6F_5)_3$

B	0.612554000	-0.078282000	-0.731228000
C	2.140521000	-0.313851000	-0.546327000
C	3.066370000	0.695802000	-0.775690000
C	2.644384000	-1.543409000	-0.142148000
C	4.425477000	0.502150000	-0.625862000
C	3.995025000	-1.766590000	0.039533000
C	4.886186000	-0.736236000	-0.210494000
C	-0.036518000	1.242528000	-0.223323000
C	-0.985747000	1.924891000	-0.973313000
C	0.307385000	1.797089000	1.002857000
C	-1.560755000	3.104569000	-0.543206000
C	-0.263391000	2.962645000	1.474717000
C	-1.197853000	3.618636000	0.690650000
F	1.808715000	-2.542799000	0.121369000
F	2.650999000	1.887995000	-1.191403000
F	5.286472000	1.478149000	-0.872140000
F	6.181267000	-0.935604000	-0.051844000
F	4.444439000	-2.944170000	0.446891000
F	1.188506000	1.177671000	1.781585000
F	0.069020000	3.456220000	2.658056000
F	-1.745559000	4.739443000	1.121852000
F	-2.447742000	3.745060000	-1.289893000
F	-1.338408000	1.461368000	-2.167987000
C	-0.264102000	-1.163141000	-1.422357000
C	0.175553000	-1.845095000	-2.549892000
C	-1.523382000	-1.498591000	-0.941417000
C	-0.591510000	-2.803869000	-3.181765000
C	-2.308481000	-2.467760000	-1.534273000
C	-1.837228000	-3.116726000	-2.663491000
F	-1.991382000	-0.903387000	0.150829000

F	-3.496620000	-2.781837000	-1.040221000
F	-2.579124000	-4.038118000	-3.248783000
F	-0.154087000	-3.422299000	-4.268315000
F	1.359600000	-1.551208000	-3.077138000

	Hatree	kcal mol^{-1}
E PBEh-3c	-2203.915859	-1382978.139
G	0.1052714	66.05880358
E SMD PBEh-3c	-2203.919238	-1382980.259
E revDSD-PBEP86-D4/def2-QZVPP	-2206.203178	-1384413.453

20

B	0.594079000	-0.593961000	-0.544363000
C	2.187841000	-0.437865000	-0.283678000
C	2.864065000	0.769964000	-0.334875000
C	2.973876000	-1.549358000	-0.023809000
C	4.228617000	0.875851000	-0.121103000
C	4.335412000	-1.488046000	0.199733000
C	4.967187000	-0.259036000	0.153135000
C	-0.253278000	0.767745000	-0.298944000
C	-0.864585000	1.490867000	-1.309841000
C	-0.375543000	1.303686000	0.973365000
C	-1.570429000	2.659642000	-1.075532000
C	-1.072673000	2.462925000	1.251630000
C	-1.679173000	3.145354000	0.213141000
F	2.410641000	-2.765888000	0.003306000
F	2.220414000	1.899401000	-0.623774000
F	4.832166000	2.056038000	-0.179530000
F	6.271886000	-0.172571000	0.364156000
F	5.035693000	-2.587805000	0.453983000
F	0.219673000	0.692465000	2.008084000
F	-1.162171000	2.924599000	2.493926000
F	-2.353337000	4.259931000	0.452814000
F	-2.140463000	3.316853000	-2.077443000
F	-0.773919000	1.098265000	-2.579071000
C	0.220671000	-1.369392000	-1.919301000
C	1.072346000	-1.467438000	-3.007115000
C	-1.023023000	-1.959001000	-2.082020000
C	0.724804000	-2.125918000	-4.175333000
C	-1.408508000	-2.628251000	-3.226906000
C	-0.520487000	-2.714093000	-4.283435000
F	-1.927207000	-1.876174000	-1.095307000
F	-2.612476000	-3.181191000	-3.322425000
F	-0.864593000	-3.350646000	-5.392797000
F	1.575862000	-2.194532000	-5.191063000
F	2.275868000	-0.897910000	-2.983787000

N	0.104701000	-1.570312000	0.601575000
C	-0.237427000	-2.266955000	1.432444000
C	-0.671227000	-3.150505000	2.485698000
H	-1.604210000	-3.632372000	2.198855000
H	0.087452000	-3.911207000	2.660994000
H	-0.827044000	-2.580787000	3.400099000

	Hatree	kcal mol^{-1}
E PBEh-3c	-2336.402438	-1466114.726
G	0.15196543	95.359751
E SMD PBEh-3c	-2336.415592	-1466122.98
E revDSD-PBEP86-D4/def2-QZVPP	-2338.814688	-1467628.435

21

C	-2.690544000	-2.739940000	2.603657000
C	-1.412592000	-2.384094000	2.287950000
C	-3.759592000	-2.483594000	1.727839000
C	-1.163648000	-1.742611000	1.059800000
B	0.485652000	-0.608585000	-0.481721000
C	2.106816000	-0.537925000	-0.325302000
C	2.884878000	0.602939000	-0.444620000
C	2.813902000	-1.715072000	-0.119838000
C	4.266073000	0.590807000	-0.340108000
C	4.190680000	-1.769225000	-0.005074000
C	4.923011000	-0.602301000	-0.113551000
C	-0.266965000	0.838828000	-0.296567000
C	-1.175570000	1.425961000	-1.159392000
C	-0.020873000	1.563140000	0.864934000
C	-1.775489000	2.653097000	-0.928067000
C	-0.592636000	2.790975000	1.137759000
C	-1.476473000	3.343996000	0.228194000
F	2.163938000	-2.878647000	-0.042135000
F	2.329242000	1.793410000	-0.675549000
F	4.962772000	1.716423000	-0.454883000
F	6.244550000	-0.629672000	-0.008110000
F	4.812808000	-2.925980000	0.200021000
F	0.813709000	1.077246000	1.782734000
F	-0.309753000	3.440998000	2.259697000
F	-2.044998000	4.514670000	0.474776000
F	-2.656540000	3.148283000	-1.791300000
F	-1.582248000	0.785696000	-2.268132000
C	-3.518410000	-1.851845000	0.542633000
H	-4.758321000	-2.791448000	1.990239000
H	-2.886890000	-3.238983000	3.543283000
H	-0.574623000	-2.584695000	2.938530000
N	-2.234133000	-1.489137000	0.272916000
O	0.018210000	-1.432723000	0.717059000
H	-2.053987000	-1.046786000	-0.619815000
C	-4.557994000	-1.507083000	-0.506208000

C	-5.889689000	-2.182778000	-0.181351000
H	-6.311190000	-1.833172000	0.761728000
H	-6.613540000	-1.943496000	-0.959827000
H	-5.799381000	-3.268978000	-0.140402000
C	-4.089943000	-1.989638000	-1.888738000
H	-3.222689000	-1.446301000	-2.267595000
H	-3.849252000	-3.053169000	-1.886164000
H	-4.885889000	-1.829229000	-2.615776000
C	-4.768865000	0.014270000	-0.524553000
H	-5.094673000	0.383133000	0.448414000
H	-3.871385000	0.562513000	-0.807824000
H	-5.537914000	0.268906000	-1.254487000
C	0.208103000	-1.387202000	-1.900797000
C	0.622855000	-0.757469000	-3.068642000
C	-0.252139000	-2.682780000	-2.076345000
C	0.560045000	-1.333707000	-4.320781000
C	-0.341504000	-3.298916000	-3.315968000
C	0.066960000	-2.621335000	-4.445278000
F	-0.659228000	-3.426622000	-1.038240000
F	-0.815123000	-4.536934000	-3.418680000
F	-0.010624000	-3.195413000	-5.636972000
F	0.963409000	-0.673576000	-5.398814000
F	1.117663000	0.477681000	-2.996505000

	Hatree	kcal mol^{-1}
E PBEh-3c	-2683.707604	-1684052.016
G	0.3128808	196.3356744
E SMD PBEh-3c	-2683.724099	-1684062.367
E revDSD-PBEP86-D4/def2-QZVPP	-2686.404891	-1685744.59

TS$_{6/22}$

C	-1.329165000	4.106821000	-1.141203000
C	-0.953824000	2.948133000	-1.593874000
C	-0.524096000	1.853465000	-2.211610000
H	0.527217000	1.747762000	-2.443108000
H	-1.170633000	2.113922000	0.419282000
H	-1.539943000	4.215882000	-0.085063000
H	-1.204114000	1.209682000	-2.754125000
B	-0.654818000	1.263073000	-0.245936000
C	-1.637566000	0.000336000	-0.349363000
C	-1.337661000	-1.243752000	0.187128000
C	-2.922251000	0.126921000	-0.860639000
C	-2.228248000	-2.305417000	0.174460000
C	-3.837257000	-0.907201000	-0.899142000
C	-3.482763000	-2.138508000	-0.378291000
C	0.895174000	1.085030000	0.076764000
C	1.520244000	1.843675000	1.054638000
C	1.703187000	0.171470000	-0.586209000
C	2.862329000	1.701563000	1.369305000
C	3.043396000	0.000282000	-0.305293000

C	3.624483000	0.773865000	0.685756000
F	-3.319694000	1.305854000	-1.351612000
F	-5.046047000	-0.728562000	-1.418758000
F	-4.343176000	-3.145210000	-0.401426000
F	-1.887754000	-3.477148000	0.695924000
F	-0.165571000	-1.465214000	0.779719000
F	0.836363000	2.755844000	1.742518000
F	3.420790000	2.447000000	2.315317000
F	4.908782000	0.627486000	0.972141000
F	3.776284000	-0.885575000	-0.968462000
F	1.178685000	-0.577932000	-1.559098000
C	-1.490357000	5.303936000	-2.027468000
H	-0.843272000	6.112514000	-1.686775000
H	-1.248886000	5.084102000	-3.064643000
H	-2.517490000	5.666740000	-1.983264000

	Hatree	kcal mol^{-1}
E PBEh-3c	-1633.749135	-1025193.103
G	0.15661909	98.27996686
E SMD PBEh-3c	-1633.75794	-1025198.628
E DSD-BLYP-D3BJ/def2-QZVPP	-1635.407364	-1026233.657

22

C	-1.172378000	3.629472000	-0.868122000
C	-1.431336000	2.427322000	-1.373954000
C	-0.454267000	1.575798000	-2.140728000
H	-0.940932000	1.198767000	-3.045495000
H	-2.416756000	1.996467000	-1.216286000
H	-0.191148000	4.067371000	-1.023627000
H	0.407625000	2.158413000	-2.463539000
B	-0.085522000	0.377795000	-1.197016000
C	-1.226573000	-0.592655000	-0.711732000
C	-1.464442000	-0.764460000	0.641926000
C	-2.045643000	-1.291204000	-1.582557000
C	-2.470460000	-1.581431000	1.122630000
C	-3.050160000	-2.131863000	-1.141044000
C	-3.263241000	-2.269705000	0.220232000
C	1.368140000	0.117144000	-0.675364000
C	2.213641000	1.134544000	-0.253305000
C	1.884384000	-1.172490000	-0.626750000
C	3.495773000	0.895935000	0.203600000
C	3.168684000	-1.447082000	-0.197206000
C	3.973755000	-0.403132000	0.224796000
F	-1.846538000	-1.191680000	-2.896031000
F	-3.805152000	-2.803164000	-1.999320000
F	-4.226368000	-3.062402000	0.659228000
F	-2.683874000	-1.711068000	2.424420000
F	-0.716928000	-0.101209000	1.522204000
F	1.782143000	2.391161000	-0.240610000
F	4.267824000	1.890184000	0.618649000

F	5.201549000	-0.646480000	0.647039000
F	3.634375000	-2.687736000	-0.185471000
F	1.142311000	-2.195724000	-1.040987000
C	-2.149936000	4.455831000	-0.101390000
H	-2.325743000	5.415327000	-0.591733000
H	-3.110743000	3.953274000	0.005975000
H	-1.774786000	4.680476000	0.899180000

	Hatree	kcal mol^{-1}
E PBEh-3c	-1633.819086	-1025236.998
G	0.15905692	99.80972834
E SMD PBEh-3c	-1633.826144	-1025241.427
E revDSD-PBEP86-D4/def2-QZVPP	-1635.468896	-1026272.269

23

C	-1.530522000	3.919695000	-1.359480000
C	-1.483651000	2.612027000	-1.609928000
C	-0.244153000	1.784229000	-1.595799000
H	-0.178030000	1.174405000	-2.499278000
H	-2.419799000	2.098319000	-1.810623000
H	-0.597342000	4.452359000	-1.184452000
H	0.628967000	2.444453000	-1.616683000
B	-0.077048000	0.803626000	-0.293181000
C	-1.177593000	-0.371771000	-0.033578000
C	-1.208567000	-0.996299000	1.204906000
C	-2.083280000	-0.859501000	-0.960590000
C	-2.075147000	-2.023423000	1.527808000
C	-2.970007000	-1.886823000	-0.680871000
C	-2.967089000	-2.470896000	0.571048000
C	1.436709000	0.221451000	-0.219432000
C	2.487044000	0.816626000	0.459120000
C	1.767749000	-0.921659000	-0.931108000
C	3.773889000	0.307860000	0.467827000
C	3.040566000	-1.463563000	-0.952712000
C	4.051612000	-0.844674000	-0.241501000
F	-2.136266000	-0.360643000	-2.195878000
F	-3.820916000	-2.316562000	-1.605772000
F	-3.811499000	-3.452946000	0.852864000
F	-2.060741000	-2.577239000	2.735515000
F	-0.358466000	-0.599818000	2.160434000
F	2.293796000	1.951112000	1.146463000
F	4.740538000	0.918882000	1.145497000
F	5.278789000	-1.346267000	-0.248450000
F	3.300372000	-2.563324000	-1.649862000
F	0.836628000	-1.544036000	-1.656613000
N	-0.322538000	1.795115000	0.897531000
C	-0.648206000	2.631065000	1.597895000
C	-1.053548000	3.684082000	2.496224000
H	-1.493906000	3.258370000	3.396035000
H	-0.188159000	4.284392000	2.771100000

H	-1.786168000	4.317570000	1.999063000
C	-2.775749000	4.747405000	-1.348288000
H	-2.918410000	5.261504000	-0.393410000
H	-2.742319000	5.526870000	-2.112302000
H	-3.662798000	4.141557000	-1.532780000

	Hatree	kcal mol^{-1}
E PBEh-3c	-1766.306502	-1108374.11
G	0.20538053	128.8782337
E SMD PBEh-3c	-1766.321869	-1108383.753
E revDSD-PBEP86-D4/def2-QZVPP	-1768.081476	-1109487.923

$TS_{23/24}$

C	-1.297099000	3.784421000	-0.906020000
C	-1.339166000	2.600469000	-1.615169000
C	-0.213064000	1.741486000	-1.714688000
H	-0.261100000	1.002079000	-2.508708000
H	-2.311862000	2.220733000	-1.913686000
H	-0.330028000	4.283866000	-0.854921000
H	0.735130000	2.276444000	-1.740492000
B	-0.089120000	0.796495000	-0.218864000
C	-1.176066000	-0.396180000	-0.009192000
C	-1.132140000	-1.080840000	1.198279000
C	-2.148615000	-0.826270000	-0.894115000
C	-1.987018000	-2.118520000	1.520539000
C	-3.026607000	-1.860006000	-0.611763000
C	-2.944969000	-2.510339000	0.603650000
C	1.424791000	0.236789000	-0.201810000
C	2.454200000	0.801948000	0.533600000
C	1.773245000	-0.861851000	-0.971698000
C	3.743289000	0.297134000	0.535266000
C	3.048715000	-1.395778000	-1.000659000
C	4.040562000	-0.811219000	-0.235074000
F	-2.291859000	-0.252418000	-2.094841000
F	-3.944186000	-2.229628000	-1.499019000
F	-3.778528000	-3.500482000	0.889514000
F	-1.900310000	-2.736510000	2.692745000
F	-0.223718000	-0.740185000	2.113320000
F	2.236053000	1.886360000	1.279907000
F	4.695529000	0.869770000	1.263912000
F	5.271117000	-1.304064000	-0.247913000
F	3.329766000	-2.451907000	-1.755095000
F	0.854117000	-1.444647000	-1.745986000
N	-0.384840000	1.830427000	0.819586000
C	-0.936694000	2.877936000	0.899086000
C	-1.422242000	3.809851000	1.932632000
H	-1.142502000	3.426060000	2.911816000
H	-0.989463000	4.799604000	1.799053000
H	-2.505637000	3.903658000	1.884594000
C	-2.494226000	4.694928000	-0.841191000
H	-2.439428000	5.396890000	-0.010471000
H	-2.562916000	5.296900000	-1.748690000

H	-3.424555000	4.134471000	-0.746832000

	Hatree	kcal mol^{-1}
E PBEh-3c	-1766.288981	-1108363.115
G	0.20771286	130.3417929
E SMD PBEh-3c	-1766.301151	-1108370.752
E revDSD-PBEP86-D4/def2-QZVPP	-1768.061184	-1109475.189

24

B	1.304252000	0.247015000	0.863941000
C	1.662118000	1.688033000	0.331097000
C	2.945956000	2.001197000	-0.084771000
C	0.720990000	2.701974000	0.256485000
C	3.291390000	3.253657000	-0.557703000
C	1.026307000	3.964330000	-0.217467000
C	2.320654000	4.237667000	-0.624299000
C	1.834581000	-1.047776000	0.136266000
C	2.435228000	-2.086587000	0.826126000
C	1.716088000	-1.194840000	-1.234712000
C	2.909900000	-3.219567000	0.190980000
C	2.171501000	-2.315086000	-1.904707000
C	2.773383000	-3.330461000	-1.181959000
F	-0.537622000	2.473234000	0.629971000
F	3.901993000	1.077679000	-0.013381000
F	4.533000000	3.522190000	-0.939070000
F	2.631006000	5.442757000	-1.075112000
F	0.098530000	4.910578000	-0.287247000
F	1.126813000	-0.234974000	-1.945865000
F	2.034362000	-2.429695000	-3.219165000
F	3.217709000	-4.410952000	-1.804440000
F	3.492326000	-4.194612000	0.877452000
F	2.593748000	-2.003078000	2.145874000
N	0.527180000	0.120549000	1.957846000
C	-0.204096000	0.029951000	2.960083000
C	-1.717450000	-0.034502000	2.815669000
H	-1.933311000	0.131286000	1.756648000
C	-2.164619000	-1.429888000	3.158179000
H	-1.766406000	-2.207918000	2.513692000
C	-2.975683000	-1.773120000	4.149632000
H	-3.413030000	-1.050082000	4.825434000
H	-3.244616000	-2.807336000	4.315299000
C	0.370992000	-0.014363000	4.346450000
H	1.407065000	-0.341840000	4.322990000
H	0.340506000	0.983357000	4.789590000
H	-0.205202000	-0.677146000	4.990986000
C	-2.390218000	1.065671000	3.627812000
H	-3.469514000	1.036746000	3.480445000
H	-2.198831000	0.972663000	4.697236000

	Hatree	kcal mol^{-1}

E PBEh-3c	-1766.337055	-1108393.282
G	0.20691522	129.8412662
E SMD PBEh-3c	-1766.346021	-1108398.908
E revDSD-PBEP86-D4/def2-QZVPP	-1768.100464	-1109499.838

25

C	-2.885135000	-0.293745000	-3.926060000
C	-1.611908000	-0.051474000	-3.498644000
C	-3.924825000	-0.552567000	-3.017104000
C	-1.350226000	-0.069012000	-2.113095000
B	0.392663000	0.086605000	-0.223369000
C	1.086228000	1.556135000	-0.028006000
C	2.381424000	1.806683000	0.392192000
C	0.313281000	2.685556000	-0.254595000
C	2.892201000	3.083767000	0.559579000
C	0.782171000	3.977450000	-0.105040000
C	2.088035000	4.176878000	0.302807000
C	1.410312000	-1.188594000	-0.338634000
C	1.276758000	-2.395260000	0.327696000
C	2.444597000	-1.143489000	-1.266200000
C	2.124092000	-3.474793000	0.124105000
C	3.310015000	-2.193886000	-1.500077000
C	3.148246000	-3.372456000	-0.793681000
F	-0.966882000	2.556702000	-0.626660000
F	3.208783000	0.801534000	0.690548000
F	4.143501000	3.266529000	0.968128000
F	2.559831000	5.407342000	0.456040000
F	-0.005160000	5.022717000	-0.342756000
F	2.640648000	-0.038883000	-1.986612000
F	4.288202000	-2.089152000	-2.393703000
F	3.966568000	-4.395020000	-1.003214000
F	1.949193000	-4.608125000	0.798421000
F	0.297259000	-2.596072000	1.216622000
C	-3.651623000	-0.557302000	-1.676756000
H	-4.923150000	-0.741629000	-3.377081000
H	-3.100959000	-0.287119000	-4.986500000
H	-0.797882000	0.148762000	-4.179450000
N	-2.379179000	-0.317766000	-1.281555000
O	-0.178179000	0.139903000	-1.663922000
H	-2.061716000	-0.287211000	-0.265902000
N	-0.749961000	-0.075540000	0.770645000
C	-0.648815000	-0.004216000	2.028731000
C	-4.661957000	-0.792100000	-0.573339000
C	-6.039307000	-1.126119000	-1.142592000
H	-6.738647000	-1.293825000	-0.323254000
H	-6.442597000	-0.313626000	-1.748465000
H	-6.024278000	-2.034211000	-1.747053000
C	-4.777159000	0.491392000	0.263003000
H	-3.821428000	0.812480000	0.677762000
H	-5.166086000	1.315470000	-0.336378000
H	-5.460655000	0.331574000	1.098031000

C	-4.199038000	-1.968261000	0.298929000
H	-4.139139000	-2.888631000	-0.283251000
H	-3.223260000	-1.805302000	0.757013000
H	-4.911320000	-2.130070000	1.108831000
C	-1.896243000	-0.109562000	2.893518000
H	-2.739696000	-0.206795000	2.204458000
C	-1.853524000	-1.369232000	3.712498000
H	-1.740098000	-2.284305000	3.139063000
C	-1.941427000	-1.454276000	5.033249000
H	-2.053620000	-0.586299000	5.670003000
H	-1.906557000	-2.412710000	5.532784000
C	0.631180000	0.204801000	2.790831000
H	1.493835000	-0.229380000	2.293126000
H	0.824140000	1.273736000	2.906212000
H	0.567365000	-0.223935000	3.790490000
C	-2.102689000	1.163786000	3.706752000
H	-3.044354000	1.122934000	4.254548000
H	-1.305131000	1.327730000	4.432018000
H	-2.138499000	2.033802000	3.050461000

	Hatree	kcal mol^{-1}
E PBEh-3c	-2246.113253	-1409457.405
G	0.41334256	259.3763832
E SMD PBEh-3c	-2246.131897	-1409469.104
E revDSD-PBEP86-D4/def2-QZVPP	-2248.285449	-1410820.478

TS$_{25/26}$

C	-2.859717000	-0.303202000	-3.888461000
C	-1.634724000	0.131180000	-3.464965000
C	-3.828878000	-0.725385000	-2.969765000
C	-1.375673000	0.138402000	-2.083433000
B	0.356922000	0.287484000	-0.260672000
C	1.202832000	1.647156000	0.057038000
C	2.530329000	1.721212000	0.442341000
C	0.549166000	2.866629000	-0.049121000
C	3.184828000	2.917732000	0.684588000
C	1.164029000	4.083955000	0.180859000
C	2.497421000	4.107779000	0.545323000
C	1.229036000	-1.085130000	-0.449506000
C	1.011043000	-2.295539000	0.186014000
C	2.223295000	-1.105126000	-1.421189000
C	1.734869000	-3.445050000	-0.095332000
C	2.967295000	-2.225829000	-1.732573000
C	2.719459000	-3.409546000	-1.060037000
F	-0.748915000	2.904392000	-0.365488000
F	3.245416000	0.610582000	0.640261000
F	4.460902000	2.931811000	1.055304000
F	3.107031000	5.263488000	0.773032000
F	0.489978000	5.223921000	0.062527000
F	2.502696000	0.005409000	-2.103084000
F	3.911142000	-2.183066000	-2.666597000
F	3.418769000	-4.499566000	-1.345264000

F	1.479897000	-4.579400000	0.550957000
F	0.064919000	-2.432220000	1.124969000
C	-3.540989000	-0.688536000	-1.627604000
H	-4.787492000	-1.073166000	-3.319952000
H	-3.083806000	-0.327831000	-4.946983000
H	-0.862300000	0.453757000	-4.147559000
N	-2.328115000	-0.253209000	-1.225231000
O	-0.230910000	0.529271000	-1.641086000
H	-1.875505000	-0.118771000	-0.110224000
N	-0.816852000	0.136452000	0.732959000
C	-0.750200000	0.182785000	1.997205000
C	-4.515782000	-1.084611000	-0.536039000
C	-5.802061000	-1.676591000	-1.111100000
H	-6.463491000	-1.967679000	-0.294389000
H	-6.348966000	-0.960594000	-1.726252000
H	-5.611197000	-2.569344000	-1.708499000
C	-4.883574000	0.176020000	0.259570000
H	-4.011240000	0.690094000	0.660900000
H	-5.415123000	0.890474000	-0.370293000
H	-5.533859000	-0.080156000	1.097550000
C	-3.868510000	-2.143477000	0.366772000
H	-3.678259000	-3.063020000	-0.188256000
H	-2.918956000	-1.823752000	0.792095000
H	-4.534618000	-2.386575000	1.195749000
C	-2.011152000	0.095100000	2.837192000
H	-2.848291000	-0.021557000	2.145422000
C	-1.981715000	-1.136932000	3.698850000
H	-1.856953000	-2.071700000	3.160879000
C	-2.101277000	-1.175425000	5.019038000
H	-2.227848000	-0.286439000	5.623106000
H	-2.080383000	-2.116399000	5.551281000
C	0.524937000	0.347191000	2.772829000
H	1.368506000	-0.146655000	2.297925000
H	0.773517000	1.406004000	2.866796000
H	0.421014000	-0.055673000	3.779306000
C	-2.219950000	1.397656000	3.604223000
H	-3.169498000	1.378191000	4.138913000
H	-1.431367000	1.580428000	4.334652000
H	-2.242882000	2.244917000	2.918674000

	Hatree	kcal mol^{-1}
E PBEh-3c	-2246.11129	-1409456.173
G	0.41084322	257.8080236
E SMD PBEh-3c	-2246.129164	-1409467.389
E revDSD-PBEP86-D4/def2-QZVPP	-2248.283148	-1410819.034

26

C	-2.700097000	-0.100983000	-3.920930000
C	-1.660356000	0.575540000	-3.329998000
C	-3.565829000	-0.870967000	-3.146352000
C	-1.520130000	0.458792000	-1.942515000

B	0.190139000	0.610836000	-0.178750000
C	1.308319000	1.743736000	0.164547000
C	2.670067000	1.526243000	0.288287000
C	0.900376000	3.062383000	0.314208000
C	3.580992000	2.544640000	0.519626000
C	1.775536000	4.107348000	0.548338000
C	3.130096000	3.844154000	0.643494000
C	0.756987000	-0.889334000	-0.498576000
C	0.579329000	-2.031795000	0.259964000
C	1.431322000	-1.083555000	-1.698253000
C	1.010283000	-3.287768000	-0.135023000
C	1.879480000	-2.316388000	-2.131627000
C	1.662333000	-3.431227000	-1.342206000
F	-0.398591000	3.368704000	0.273280000
F	3.173041000	0.292076000	0.227187000
F	4.878420000	2.282729000	0.630884000
F	3.986624000	4.830585000	0.867383000
F	1.332078000	5.352218000	0.689356000
F	1.692188000	-0.042155000	-2.487948000
F	2.517743000	-2.442627000	-3.289331000
F	2.082523000	-4.624560000	-1.739041000
F	0.799946000	-4.349653000	0.637466000
F	-0.029504000	-1.984090000	1.456345000
C	-3.359877000	-0.927162000	-1.779530000
H	-4.375468000	-1.408308000	-3.615520000
H	-2.842324000	-0.044332000	-4.992516000
H	-0.959941000	1.170739000	-3.897896000
N	-2.353564000	-0.258593000	-1.207503000
O	-0.533050000	1.110282000	-1.349075000
H	-1.801433000	0.360318000	0.608551000
N	-0.875480000	0.552739000	1.006352000
C	-0.800396000	0.660851000	2.270760000
C	-4.239246000	-1.725957000	-0.828271000
C	-5.331425000	-2.500811000	-1.563879000
H	-5.929204000	-3.065976000	-0.847578000
H	-6.012520000	-1.840709000	-2.103488000
H	-4.916859000	-3.217470000	-2.274593000
C	-4.909614000	-0.760188000	0.155720000
H	-4.173969000	-0.196129000	0.729702000
H	-5.543647000	-0.040076000	-0.363659000
H	-5.534336000	-1.307426000	0.864109000
C	-3.365952000	-2.723419000	-0.058757000
H	-2.880772000	-3.428185000	-0.735911000
H	-2.583113000	-2.223432000	0.509272000
H	-3.974866000	-3.300009000	0.640431000
C	-2.063048000	0.620396000	3.099393000

H	-2.910375000	0.571680000	2.405644000
C	-2.083129000	-0.646898000	3.913173000
H	-2.065821000	-1.565120000	3.335188000
C	-2.131048000	-0.718988000	5.235787000
H	-2.156880000	0.155886000	5.872114000
H	-2.156419000	-1.676602000	5.736743000
C	0.494389000	0.822777000	2.989733000
H	1.306967000	0.297070000	2.497541000
H	0.765337000	1.878628000	3.043598000
H	0.406581000	0.448809000	4.008278000
C	-2.195781000	1.905331000	3.911531000
H	-3.142166000	1.909864000	4.450745000
H	-1.395167000	2.020551000	4.642026000
H	-2.181474000	2.777306000	3.258118000

	Hatree	kcal mol^{-1}
E PBEh-3c	-2246.129142	-1409467.375
G	0.41463253	260.1858516
E SMD PBEh-3c	-2246.148889	-1409479.766
E revDSD-PBEP86-D4/def2-QZVPP	-2248.299352	-1410829.202

27

N	-0.506414000	0.201305000	0.828270000
C	-0.718150000	-0.347217000	1.947347000
C	-1.924902000	-0.114631000	2.840694000
H	-2.189987000	-1.086020000	3.275803000
C	-1.594474000	0.807797000	3.986903000
H	-2.290614000	0.769592000	4.819686000
C	-0.585859000	1.667251000	4.036234000
H	0.138700000	1.762073000	3.236685000
H	-0.449682000	2.316058000	4.890549000
C	0.294673000	-1.315177000	2.484501000
H	1.176756000	-1.336666000	1.849532000
H	0.589362000	-1.054225000	3.502034000
H	-0.129363000	-2.321330000	2.525443000
C	-3.155220000	0.407139000	2.099632000
H	-4.002773000	0.490867000	2.779735000
H	-2.983441000	1.402560000	1.687731000
H	-3.446025000	-0.260238000	1.287756000
H	-1.265842000	0.825587000	0.567043000

	Hatree	kcal mol^{-1}
E PBEh-3c	-289.3357115	-181560.9077
G	0.13112181	82.28018143
E SMD PBEh-3c	-289.3439196	-181566.0583
E revDSD-PBEP86-D4/def2-QZVPP	-289.5786067	-181713.3267

28

B	0.348274000	0.163827000	-0.443512000
C	0.792848000	1.726267000	-0.226939000
C	1.491332000	2.079431000	0.919934000
C	0.435111000	2.793244000	-1.037310000
C	1.856187000	3.370578000	1.239509000
C	0.785287000	4.106773000	-0.759384000
C	1.504388000	4.397132000	0.381756000
C	1.537298000	-0.947849000	-0.402370000
C	1.191672000	-2.292653000	-0.407937000
C	2.893640000	-0.689879000	-0.523049000
C	2.105658000	-3.323614000	-0.491931000
C	3.845927000	-1.693894000	-0.615468000
C	3.450881000	-3.016840000	-0.597229000
F	-0.302774000	2.614511000	-2.132684000
F	1.835065000	1.128491000	1.798718000
F	2.527559000	3.636080000	2.354841000
F	1.840205000	5.647091000	0.663726000
F	0.420468000	5.087778000	-1.576103000
F	3.353804000	0.557238000	-0.573071000
F	5.133835000	-1.393571000	-0.726146000
F	4.350300000	-3.985713000	-0.680338000
F	1.711673000	-4.592106000	-0.468676000
F	-0.098540000	-2.634500000	-0.296568000
N	-0.685456000	0.024012000	0.766935000
C	-0.706615000	-0.464440000	1.945024000
C	-1.833437000	-0.079634000	2.887279000
H	-1.895280000	-0.878165000	3.634461000
C	-1.362407000	1.168941000	3.596758000
H	-0.444910000	1.067949000	4.167868000
C	-1.958523000	2.351702000	3.556920000
H	-2.877087000	2.533831000	3.015513000
H	-1.541700000	3.195415000	4.088569000
C	0.320386000	-1.366010000	2.524625000
H	1.266088000	-1.375311000	2.000429000
H	0.497567000	-1.090900000	3.564334000
H	-0.087275000	-2.379798000	2.537290000
C	-3.200471000	0.043630000	2.229554000
H	-3.952266000	0.239107000	2.992357000
H	-3.268276000	0.859477000	1.508176000
H	-3.473712000	-0.877117000	1.717599000
H	-1.486781000	0.619215000	0.585640000
C	-0.430553000	-0.115965000	-1.855358000
C	0.307898000	0.007186000	-3.025437000
C	-1.726650000	-0.563202000	-2.034328000

C	-0.184510000	-0.286986000	-4.280508000
C	-2.266134000	-0.868182000	-3.274053000
C	-1.489030000	-0.731923000	-4.405392000
F	1.564961000	0.449195000	-2.957048000
F	0.572162000	-0.145015000	-5.361425000
F	-1.986255000	-1.019748000	-5.599449000
F	-3.521377000	-1.292377000	-3.377073000
F	-2.554954000	-0.721835000	-0.988581000

	Hatree	kcal mol^{-1}
E PBEh-3c	-2493.304706	-1564572.389
G	0.26578856	166.7848464
E SMD PBEh-3c	-2493.318685	-1564581.161
E revDSD-PBEP86-D4/def2-QZVPP	-2495.835403	-1566160.426

TS$_{26/29}$

C	2.022385000	4.425624000	1.490488000
C	1.641605000	3.542347000	0.511699000
C	1.085143000	4.927909000	2.392080000
C	0.299752000	3.154219000	0.456873000
B	-0.418226000	0.908554000	-0.375265000
C	1.007027000	0.149533000	-0.067281000
C	1.776533000	-0.454304000	-1.051588000
C	1.588161000	0.174100000	1.193524000
C	3.015457000	-1.023665000	-0.805222000
C	2.822982000	-0.378481000	1.482874000
C	3.540013000	-0.989545000	0.472491000
C	-1.155654000	0.427491000	-1.747155000
C	-1.634130000	-0.872178000	-1.812758000
C	-1.395127000	1.200771000	-2.871239000
C	-2.307207000	-1.392396000	-2.899604000
C	-2.070220000	0.718600000	-3.983468000
C	-2.528912000	-0.582808000	-3.998570000
F	0.961495000	0.775824000	2.216178000
F	1.356817000	-0.496940000	-2.314710000
F	3.706613000	-1.595066000	-1.784552000
F	4.725254000	-1.527164000	0.724438000
F	3.324512000	-0.322070000	2.712916000
F	-0.982593000	2.465242000	-2.947854000
F	-2.281775000	1.502544000	-5.036345000
F	-3.175971000	-1.054561000	-5.056444000
F	-2.739979000	-2.649819000	-2.901305000
F	-1.427926000	-1.704623000	-0.780161000
C	-0.230958000	4.527494000	2.294787000
H	1.394138000	5.625034000	3.154555000

H	3.055476000	4.739742000	1.561991000
H	2.337799000	3.140225000	-0.209853000
N	-0.578008000	3.650865000	1.334805000
O	-0.141430000	2.363413000	-0.478386000
H	-1.203002000	-0.212225000	1.343671000
N	-1.395300000	0.612278000	0.796913000
C	-2.501628000	1.243172000	1.125098000
C	-1.334448000	5.025910000	3.212446000
C	-0.801806000	6.029628000	4.234740000
H	-1.620947000	6.365379000	4.870917000
H	-0.045694000	5.591892000	4.888302000
H	-0.377850000	6.916341000	3.760948000
C	-1.936659000	3.837455000	3.972395000
H	-2.404177000	3.118100000	3.302440000
H	-1.177770000	3.313968000	4.556008000
H	-2.705655000	4.187857000	4.662886000
C	-2.417116000	5.717502000	2.372306000
H	-2.005285000	6.558425000	1.812878000
H	-2.888571000	5.039372000	1.662022000
H	-3.201324000	6.105188000	3.024596000
C	-3.350460000	0.638132000	2.228121000
H	-3.943768000	1.449049000	2.657552000
C	-4.293463000	-0.342510000	1.583802000
H	-3.828216000	-1.185866000	1.080386000
C	-5.613725000	-0.236679000	1.601446000
H	-6.114910000	0.591926000	2.086645000
H	-6.245277000	-0.976091000	1.129070000
C	-2.892499000	2.474358000	0.580364000
H	-2.644091000	2.692817000	-0.453141000
H	-3.877967000	2.820795000	0.869614000
H	-1.752083000	3.232829000	1.126667000
C	-2.556695000	-0.027003000	3.353577000
H	-3.231370000	-0.287818000	4.167282000
H	-2.080209000	-0.958435000	3.041048000
H	-1.783326000	0.631964000	3.748893000

	Hatree	kcal mol^{-1}
E PBEh-3c	-2246.088712	-1409442.004
G	0.41051451	257.6017549
E SMD PBEh-3c	-2246.107517	-1409453.805
E revDSD-PBEP86-D4/def2-QZVPP	-2248.262215	-1410805.899

29

C	1.649110000	4.874548000	2.057027000
C	1.825367000	3.681163000	1.430207000
C	0.361817000	5.333089000	2.381804000

C	0.702068000	2.885938000	1.106913000
B	0.062975000	0.585943000	-0.013016000
C	1.235395000	-0.558366000	-0.095539000
C	1.734743000	-1.118854000	-1.261228000
C	1.858869000	-0.991428000	1.068519000
C	2.752687000	-2.060698000	-1.276843000
C	2.876276000	-1.926106000	1.098461000
C	3.324594000	-2.470739000	-0.090016000
C	-0.593700000	0.966156000	-1.455365000
C	-1.448658000	0.043681000	-2.044480000
C	-0.405164000	2.134794000	-2.171235000
C	-2.093154000	0.259009000	-3.245709000
C	-1.034565000	2.392954000	-3.379918000
C	-1.885581000	1.450176000	-3.918626000
F	1.487513000	-0.489733000	2.255757000
F	1.269722000	-0.759835000	-2.457739000
F	3.187775000	-2.565348000	-2.426404000
F	4.299787000	-3.369385000	-0.088671000
F	3.426758000	-2.300211000	2.249676000
F	0.398086000	3.107348000	-1.719299000
F	-0.825002000	3.539726000	-4.020830000
F	-2.496569000	1.680906000	-5.073247000
F	-2.904487000	-0.657718000	-3.762111000
F	-1.664166000	-1.130412000	-1.449956000
C	-0.724958000	4.568345000	2.073147000
H	0.238080000	6.286856000	2.866698000
H	2.508414000	5.484432000	2.302710000
H	2.800827000	3.307260000	1.157482000
N	-0.516446000	3.362390000	1.461242000
O	0.889810000	1.788428000	0.508965000
H	-0.748735000	-0.640130000	1.548872000
N	-0.981698000	0.164150000	0.988946000
C	-2.269175000	0.551315000	1.164361000
C	-2.160593000	4.982284000	2.342680000
C	-2.213539000	6.332394000	3.057833000
H	-3.254941000	6.598462000	3.238347000
H	-1.714250000	6.306002000	4.027567000
H	-1.775990000	7.135174000	2.462841000
C	-2.849325000	3.939579000	3.232972000
H	-2.932638000	2.969818000	2.743575000
H	-2.317150000	3.809048000	4.176438000
H	-3.862814000	4.269447000	3.464786000
C	-2.904733000	5.115488000	1.005429000
H	-2.423149000	5.847416000	0.355976000
H	-2.968137000	4.171097000	0.466670000
H	-3.926621000	5.450619000	1.187408000
C	-2.997218000	-0.212268000	2.263025000
H	-4.022721000	0.168938000	2.284517000
C	-3.069404000	-1.663092000	1.869494000
H	-3.528245000	-1.848257000	0.903318000
C	-2.612511000	-2.692342000	2.570944000
H	-2.138907000	-2.581053000	3.537733000
H	-2.702931000	-3.702717000	2.196738000

C	-2.923148000	1.535835000	0.502104000
H	-2.530398000	2.031369000	-0.373720000
H	-3.960214000	1.729629000	0.733566000
H	-1.345251000	2.786720000	1.247896000
C	-2.378601000	0.023638000	3.638413000
H	-2.955667000	-0.483166000	4.411826000
H	-1.350583000	-0.333615000	3.704307000
H	-2.369091000	1.085971000	3.878867000

	Hatree	kcal mol^{-1}
E PBEh-3c	-2246.09765	-1409447.613
G	0.41679979	261.5458278
E SMD PBEh-3c	-2246.116224	-1409459.268
E revDSD-PBEP86-D4/def2-QZVPP	-2248.273587	-1410813.034

30

B	-0.277527000	-0.064545000	-0.160785000
C	1.146135000	-0.740994000	-0.138011000
C	1.483457000	-1.713228000	-1.065261000
C	2.128476000	-0.376674000	0.768409000
C	2.730405000	-2.308508000	-1.099369000
C	3.390664000	-0.942384000	0.760217000
C	3.687144000	-1.914694000	-0.179196000
C	-0.803244000	0.555486000	-1.507621000
C	-1.952146000	0.104580000	-2.132769000
C	-0.092481000	1.554125000	-2.146803000
C	-2.389912000	0.624704000	-3.335600000
C	-0.499709000	2.104670000	-3.348586000
C	-1.657008000	1.631748000	-3.941992000
F	1.874251000	0.564620000	1.675582000
F	0.572824000	-2.112282000	-1.950046000
F	3.016487000	-3.247254000	-1.990829000
F	4.886975000	-2.471128000	-0.196284000
F	4.313417000	-0.565271000	1.634859000
F	1.017000000	2.024867000	-1.577588000
F	0.197391000	3.071076000	-3.930747000
F	-2.064716000	2.141375000	-5.093286000
F	-3.493447000	0.171164000	-3.915070000
F	-2.663729000	-0.871401000	-1.575272000
H	-0.671276000	-0.630975000	1.767517000
N	-1.029065000	-0.068595000	1.007786000
C	-2.294634000	0.480204000	1.281246000
C	-3.136161000	-0.330946000	2.244916000
H	-4.146124000	0.088653000	2.197561000
C	-3.222546000	-1.762695000	1.792903000
H	-3.607393000	-1.901836000	0.788145000

C	-2.870514000	-2.831574000	2.494929000
H	-2.481685000	-2.772154000	3.503373000
H	-2.971168000	-3.825229000	2.080607000
C	-2.707773000	1.638157000	0.775891000
H	-2.079642000	2.267927000	0.163342000
H	-3.707301000	1.993194000	0.980422000
C	-2.637410000	-0.158203000	3.679340000
H	-3.288587000	-0.679247000	4.380997000
H	-1.627699000	-0.547314000	3.826858000
H	-2.625153000	0.896346000	3.949710000

	Hatree	kcal mol^{-1}
E PBEh-3c	-1766.330153	-1108388.951
G	0.21001917	131.7890244
E SMD PBEh-3c	-1766.338878	-1108394.426
E revDSD-PBEP86-D4/def2-QZVPP	-1768.098643	-1109498.695

10

C	1.625630000	0.214672000	1.467576000
C	2.209614000	0.060962000	2.740485000
C	3.568899000	0.000336000	2.840361000
C	4.392350000	0.084128000	1.704534000
C	3.818155000	0.231757000	0.473838000
N	2.464525000	0.306550000	0.412746000
H	4.025029000	-0.121571000	3.813985000
H	1.558312000	-0.010930000	3.598779000
H	5.463490000	0.024041000	1.806798000
H	2.006396000	0.324840000	-0.498864000
N	0.814803000	-2.230857000	-0.295683000
C	0.222772000	-1.511527000	-1.224085000
C	0.849746000	-3.559762000	-0.399860000
C	-0.379959000	-2.068187000	-2.350839000
C	0.276232000	-4.206052000	-1.485250000
C	-0.343947000	-3.441332000	-2.467462000
H	-0.857685000	-1.436948000	-3.086641000
H	0.301386000	-5.281556000	-1.575543000
H	-0.801984000	-3.924787000	-3.320736000
O	0.246473000	-0.174785000	-1.086819000
O	0.364920000	0.271987000	1.329995000
B	-0.428380000	0.466372000	0.041497000
C	1.547325000	-4.280084000	0.744017000
C	1.593105000	-5.792191000	0.536065000
H	2.109052000	-6.264812000	1.372969000
H	0.594789000	-6.230174000	0.486302000
H	2.131291000	-6.067216000	-0.373086000

C	2.982093000	-3.751902000	0.857781000	
H	2.991548000	-2.678110000	1.040596000	
H	3.502074000	-4.239167000	1.684843000	
H	3.551451000	-3.947989000	-0.053606000	
C	0.790509000	-3.975740000	2.042210000	
H	0.743812000	-2.904739000	2.231536000	
H	-0.233715000	-4.349206000	2.000494000	
H	1.285358000	-4.453361000	2.890280000	
C	4.555741000	0.327898000	-0.846491000	
C	4.026143000	-0.740987000	-1.815020000	
H	2.987681000	-0.580540000	-2.107702000	
H	4.613747000	-0.721026000	-2.733256000	
H	4.100701000	-1.741799000	-1.389114000	
C	6.053647000	0.106715000	-0.646031000	
H	6.270522000	-0.876300000	-0.225530000	
H	6.558714000	0.167137000	-1.609857000	
H	6.500551000	0.865695000	-0.002451000	
C	4.334892000	1.726869000	-1.441722000	
H	4.680818000	2.507315000	-0.763085000	
H	4.895237000	1.821972000	-2.372394000	
H	3.289641000	1.924104000	-1.678482000	
C	-0.487222000	2.082485000	-0.188081000	
C	-1.144323000	2.859510000	0.755982000	
C	0.065752000	2.782446000	-1.248055000	
C	-1.267228000	4.233564000	0.662632000	
C	-0.028296000	4.159119000	-1.375780000	
C	-0.702495000	4.888140000	-0.416515000	
C	-1.901139000	-0.187958000	0.252530000	
C	-2.831080000	-0.007147000	-0.760521000	
C	-2.325121000	-0.964562000	1.318880000	
C	-4.105420000	-0.541125000	-0.734250000	
C	-3.594218000	-1.518480000	1.382354000	
C	-4.488642000	-1.304599000	0.352461000	
F	0.749078000	2.156989000	-2.212485000	
F	0.528841000	4.782979000	-2.409866000	
F	-0.800529000	6.206258000	-0.524436000	
F	-1.912169000	4.927880000	1.593797000	
F	-1.697678000	2.277669000	1.820555000	
F	-2.499596000	0.710159000	-1.838210000	
F	-1.525460000	-1.228367000	2.351187000	
F	-3.957731000	-2.258918000	2.425906000	
F	-5.705238000	-1.831281000	0.403047000	
F	-4.956140000	-0.335717000	-1.735004000	
			Hatree	kcal mol^{-1}
E PBEh-3c			-2436.529561	-1528945.447
G			0.46069616	289.091217

E SMD PBEh-3c	-2436.551631	-1528959.295
E revDSD-PBEP86-D4/def2-QZVPP	-2438.868041	-1530412.865

$TS_{30/31}$

B	-0.400801000	-0.077557000	0.351731000
C	-0.190239000	1.419513000	-0.231211000
C	-1.041418000	2.006603000	-1.153742000
C	0.898707000	2.189248000	0.150485000
C	-0.850632000	3.286956000	-1.647245000
C	1.124185000	3.471435000	-0.315234000
C	0.235935000	4.024945000	-1.219001000
C	-1.948768000	-0.563449000	0.378852000
C	-2.468989000	-1.552758000	-0.440398000
C	-2.855752000	0.058196000	1.222460000
C	-3.804406000	-1.921780000	-0.408896000
C	-4.194733000	-0.279179000	1.281913000
C	-4.669922000	-1.283498000	0.458466000
F	1.804955000	1.690487000	0.998238000
F	-2.091463000	1.338202000	-1.627595000
F	-1.695484000	3.807149000	-2.529420000
F	0.433906000	5.250938000	-1.680984000
F	2.181042000	4.168874000	0.087552000
F	-2.441022000	1.049046000	2.017052000
F	-5.022704000	0.346345000	2.111834000
F	-5.949475000	-1.628348000	0.498930000
F	-4.260792000	-2.881478000	-1.206613000
F	-1.699421000	-2.192112000	-1.317631000
N	0.594358000	-1.009729000	-0.287652000
C	1.050034000	-2.166886000	0.223777000
C	2.429684000	-2.600175000	-0.250435000
H	2.552812000	-3.646296000	0.045326000
C	2.490442000	-2.555072000	-1.751735000
H	1.773379000	-3.194654000	-2.257199000
C	3.305135000	-1.804024000	-2.481969000
H	4.042849000	-1.143274000	-2.045460000
H	3.269520000	-1.833675000	-3.562241000
C	0.418189000	-2.868641000	1.208031000
H	0.863377000	-3.786778000	1.565500000
C	0.435318000	-0.896441000	2.654710000
N	0.029964000	-0.108112000	1.905198000
H	1.197276000	-0.605391000	-0.990083000
C	0.887616000	-1.479880000	3.904981000
H	0.846981000	-0.726437000	4.692341000
H	0.251004000	-2.319390000	4.174391000
H	1.907742000	-1.840230000	3.799209000

C	3.513661000	-1.777926000	0.443333000
H	3.454450000	-1.911640000	1.523380000
H	4.507748000	-2.093422000	0.126378000
H	3.415863000	-0.711149000	0.239861000
H	-0.631661000	-2.735583000	1.432288000

	Hatree	kcal mol^{-1}
E PBEh-3c	-1898.785705	-1191506.068
G	0.25674835	161.1120287
E SMD PBEh-3c	-1898.799848	-1191514.943
E revDSD-PBEP86-D4/def2-QZVPP	-1900.678439	-1192693.777

31

B	-0.475796000	-0.063147000	0.545856000
C	-0.170175000	1.428717000	-0.044499000
C	-0.964746000	2.104312000	-0.955761000
C	0.971044000	2.103954000	0.363975000
C	-0.681948000	3.385251000	-1.402496000
C	1.291763000	3.381226000	-0.057419000
C	0.449918000	4.029625000	-0.942491000
C	-2.001418000	-0.587525000	0.329745000
C	-2.393990000	-1.711568000	-0.371641000
C	-3.022515000	0.102511000	0.969018000
C	-3.709083000	-2.143027000	-0.441306000
C	-4.346209000	-0.290840000	0.922881000
C	-4.691143000	-1.427691000	0.212720000
F	1.844820000	1.504894000	1.178185000
F	-2.050732000	1.525385000	-1.471221000
F	-1.480485000	3.993775000	-2.272643000
F	0.739272000	5.253702000	-1.361812000
F	2.398561000	3.983941000	0.366013000
F	-2.742021000	1.214153000	1.644155000
F	-5.288818000	0.406079000	1.547140000
F	-5.955775000	-1.824555000	0.159103000
F	-4.028346000	-3.236798000	-1.128381000
F	-1.497359000	-2.458418000	-1.039251000
N	0.560280000	-0.990939000	-0.225269000
C	1.227414000	-1.927098000	0.319752000
C	2.365935000	-2.608043000	-0.395082000
H	2.358907000	-3.655289000	-0.071517000
C	2.170363000	-2.582587000	-1.884451000
H	1.337448000	-3.175483000	-2.250141000
C	2.900972000	-1.898386000	-2.754988000
H	3.744897000	-1.287647000	-2.462468000
H	2.682313000	-1.937683000	-3.813024000

C	0.901371000	-2.311021000	1.714802000
H	1.755883000	-2.804827000	2.182721000
C	0.389935000	-1.126877000	2.537476000
N	-0.195672000	-0.150043000	2.020269000
H	0.812135000	-0.764257000	-1.183868000
C	0.625390000	-1.242244000	4.014204000
H	0.159963000	-0.407010000	4.530627000
H	0.214861000	-2.175374000	4.408308000
H	1.694114000	-1.244783000	4.242032000
C	3.671591000	-1.969519000	0.089874000
H	3.777182000	-2.056638000	1.170674000
H	4.525947000	-2.467423000	-0.366017000
H	3.715019000	-0.909125000	-0.157093000
H	0.107379000	-3.068683000	1.674782000

	Hatree	kcal mol^{-1}
E PBEh-3c	-1898.831636	-1191534.89
G	0.26132452	163.9836189
E SMD PBEh-3c	-1898.849458	-1191546.074
E revDSD-PBEP86-D4/def2-QZVPP	-1900.713554	-1192715.812

32

B	-0.469424000	-0.114712000	0.546736000
C	-0.156106000	1.428575000	0.078808000
C	-0.934962000	2.197503000	-0.771852000
C	1.015012000	2.035374000	0.514234000
C	-0.601985000	3.490529000	-1.143880000
C	1.383942000	3.322828000	0.170688000
C	0.561879000	4.058220000	-0.663196000
C	-1.999856000	-0.594228000	0.193066000
C	-2.353508000	-1.572915000	-0.717165000
C	-3.061301000	-0.028881000	0.881232000
C	-3.662813000	-1.978234000	-0.926966000
C	-4.380321000	-0.395722000	0.707586000
C	-4.682500000	-1.388515000	-0.207608000
F	1.867151000	1.361473000	1.294704000
F	-2.056091000	1.707374000	-1.303045000
F	-1.385338000	4.185496000	-1.961065000
F	0.896349000	5.293241000	-1.009261000
F	2.515006000	3.854779000	0.623703000
F	-2.819934000	0.954654000	1.765866000
F	-5.352615000	0.187258000	1.400253000
F	-5.940259000	-1.765461000	-0.393657000
F	-3.943030000	-2.928024000	-1.813462000
F	-1.427789000	-2.178420000	-1.471870000

N	0.610229000	-1.015269000	-0.086026000
C	1.453805000	-1.786291000	0.555477000
C	2.514643000	-2.516201000	-0.240947000
H	2.808545000	-3.387710000	0.352921000
C	1.961711000	-3.020505000	-1.543938000
H	1.161893000	-3.748914000	-1.455119000
C	2.350696000	-2.643945000	-2.755099000
H	3.141099000	-1.923218000	-2.919394000
H	1.889737000	-3.060042000	-3.640123000
C	1.441951000	-1.880665000	1.943741000
H	2.137112000	-2.533431000	2.447934000
C	0.577835000	-1.076003000	2.674646000
N	-0.273180000	-0.272026000	2.076414000
H	0.712502000	-1.005834000	-1.092066000
C	0.629082000	-1.068976000	4.169858000
H	-0.370520000	-1.040333000	4.604081000
H	1.143460000	-1.945964000	4.554527000
H	1.168456000	-0.185843000	4.516822000
C	3.735216000	-1.605516000	-0.380762000
H	4.099908000	-1.302308000	0.599201000
H	4.542684000	-2.123138000	-0.897647000
H	3.499876000	-0.697684000	-0.936625000
H	-0.846011000	0.291451000	2.685349000

	Hatree	kcal mol^{-1}
E PBEh-3c	-1898.868417	-1191557.971
G	0.26118282	163.8947008
E SMD PBEh-3c	-1898.882627	-1191566.888
E revDSD-PBEP86-D4/def2-QZVPP	-1900.751109	-1192739.378

H_2

H	0.000000000	0.000000000	0.371229000
H	0.000000000	0.000000000	-0.371229000

	Hatree	kcal mol^{-1}
E PBEh-3c	-1.168032918	-732.9517524
G	-0.0012677	-0.795493793
E SMD PBEh-3c	-1.167554181	-732.6513405
E revDSD-PBEP86-D4/def2-QZVPP	-1.171742422	-735.2795012

9 References

[1] L. Hintermann, T. T. Dang, A. Labonne, T. Kribber, L. Xiao, P. Naumov, *Chem. - A Eur. J.*, **2009**, *15*, 7167-7179.

[2] Y. Soltani, L. C. Wilkins, R. L. Melen, *Angew. Chem. Int. Ed.* **2017**, *56*, 11995-11999.

[3] L. E. Longobardi, T. C. Johnstone, R. L. Falconer, C. A. Russell, D. W. Stephan, *Chem. - A Eur. J.* **2016**, *22*, 12665-12669.

[4] X. Tao, C. G. Daniliuc, D. Dittrich, G. Kehr, G. Erker, *Angew. Chem. Int. Ed.* **2018**, *57*, 13922-13926.

[5] N. Hu, H. Jung, Y. Zheng, J. Lee, L. Zhang, Z. Ullah, X. Xie, K. Harms, M.-H. Baik, E. Meggers, *Angew. Chem. Int. Ed.* **2018**, *57*, 6242-6246.

[6] H. Deng, Z. Meng, S. Wang, Z. Zhang, Y. Zhang, Y. Shangguan, F. Yang, D. Yuan, H. Guo, C. Zhang, *Adv. Synth. Catal.* **2019**, *361*, 3582-3587.

[7] U. H. Brinker, T. Miebach, *J. Org. Chem.* **1999**, *64*, 8000-8003.

[8] J. Kuang, S. Ma, *J. Org. Chem.* **2009**, *74*, 1763-1765.

[9] a) H. Stetter, P. Goebel, *Chem. Ber.* **1962**, *95*, 1039-1042; b) M. W. Lee, Y. V. Sevryugina, A. Khan, S. Q. Ye, *J. Med. Chem.* **2012**, *55*, 7290-7294.

[10] a) R. Blieck, J. Bahri, M. Taillefer, F. Monnier, *Org. Let.* **2016**, *18*, 1482-1485; b)S. Kitagaki, M. Komizu, C. Mukai, *Synlett* **2011**, *2011*, 1129-1132.

[11] L. Krause, R. Herbst-Irmer, G. M. Sheldrick, D. Stalke, *J. Appl. Crystallogr.* **2015**, *48*, 3-10.

[12] G. Sheldrick, *Acta Crystallogr. A* **2015**, *71*, 3-8.

[13] G. Sheldrick, *Acta Crystallogr. C* **2015**, *71*, 3-8.

[14] P. Müller, *Crystallogr. Rev.* **2009**, *15*, 57-83.

[15] A. Thorn, B. Dittrich, G. M. Sheldrick, *Acta Crystallogr. A* **2012**, *68*, 448-451.

[16] a) F. Weigend, *Phys. Chem. Chem. Phys.* **2006**, *8*, 1057-1065; b) S. Grimme, J. Antony, S. Ehrlich, H. Krieg, *J. Chem. Phys.* **2010**, *132*, 154104; c) S. Grimme, S. Ehrlich, L. Goerigk, *J. Comput. Chem.* **2011**, *32*, 1456-1465; d) H. Kruse, S. Grimme, *J. Chem. Phys.* **2012**, *136*, 154101; e) S. Grimme, J. G. Brandenburg, C. Bannwarth, A. Hansen, *J. Chem. Phys.* **2015**, *143*, 054107.

[17] A. V. Marenich, C. J. Cramer, D. G. Truhlar, *J. Phys. Chem. B* **2009**, *113*, 6378-6396.

[18] a) G. Santra, N. Sylvetsky, J. M. L. Martin, *J. Phys. Chem. A* **2019**, *123*, 5129-5143; b) E. Caldeweyher, C. Bannwarth, S. Grimme, *J Chem. Phys.* **2017**, *147*, 034112; c) E. Caldeweyher, S. Ehlert, A. Hansen, H. Neugebauer, S. Spicher, C. Bannwarth, S. Grimme, *J. Chem. Phys.* **2019**, *150*, 154122; d) F. Weigend, R. Ahlrichs, *Phys. Chem. Chem. Phys.* **2005**, *7*, 3297-3305; e) A. Hellweg, C. Hättig, S. Höfener, W. Klopper, *Theor. Chem. Acc.* **2007**, *117*, 587-597; f) F. Weigend, *J. Comput. Chem.* **2008**, *29*, 167-175.

[19] a) F. Neese, *WIREs Comput. Mol. Sci.* **2012**, *2*, 73-78; b) F. Neese, *WIREs Comput. Mol. Sci.* **2018**, *8*, e1327.
